# Molecular interaction of nitrate transporter proteins with recombinant glycinebetaine results in efficient nitrate uptake in the cyanobacterium *Anabaena* PCC 7120

**DOI:** 10.1371/journal.pone.0257870

**Published:** 2021-11-18

**Authors:** Prashant Swapnil, Mukesh Meena, Ashwani K. Rai

**Affiliations:** 1 Centre of Advanced Study in Botany, Institute of Science, Banaras Hindu University, Varanasi, India; 2 Department of Botany, University of Delhi, New Delhi, India; 3 Laboratory of Phytopathology and Microbial Biotechnology, Department of Botany, Mohanlal Sukhadia University, Udaipur, Rajasthan, India; Babasaheb Bhimrao Ambedkar University (A Central University), INDIA

## Abstract

Nitrate transport in cyanobacteria is mediated by ABC-transporter, which consists of a highly conserved ATP binding cassette (ABC) and a less conserved transmembrane domain (TMD). Under salt stress, recombinant glycinebetaine (GB) not only protected the rate of nitrate transport in transgenic *Anabaena* PCC 7120, rather stimulated the rate by interacting with the ABC-transporter proteins. *In silico* analyses revealed that nrtA protein consisted of 427 amino acids, the majority of which were hydrophobic and contained a Tat (twin-arginine translocation) signal profile of 34 amino acids (1–34). The nrtC subunit of 657 amino acids contained two hydrophobic distinct domains; the N-terminal (5–228 amino acids), which was 59% identical to nrtD (the ATP-binding subunit) and the C-terminal (268–591), 28.2% identical to nrtA, suggesting C-terminal as a solute binding domain and N-terminal as ATP binding domain. Subunit nrtD consisted of 277 amino acids and its N-terminal (21–254) was an ATP binding motif. Phylogenetic analysis revealed that nitrate-ABC-transporter proteins are highly conserved among the cyanobacterial species, though variation existed in sequences resulting in several subclades. *Nostoc* PCC 7120 was very close to *Anabaena variabilis* ATCC 29413, *Anabaena* sp. 4–3 and *Anabaena* sp. CA = ATCC 33047. On the other, *Nostoc* spp. NIES-3756 and PCC 7524 were often found in the same subclade suggesting more work before referring it to *Anabaena* PCC 7120 or *Nostoc* PCC 7120. The molecular interaction of nitrate with nrtA was hydrophilic, while hydrophobic with nrtC and nrtD. GB interaction with nrtACD was hydrophobic and showed higher affinity compared to nitrate.

## 1. Introduction

**Fig 1:**
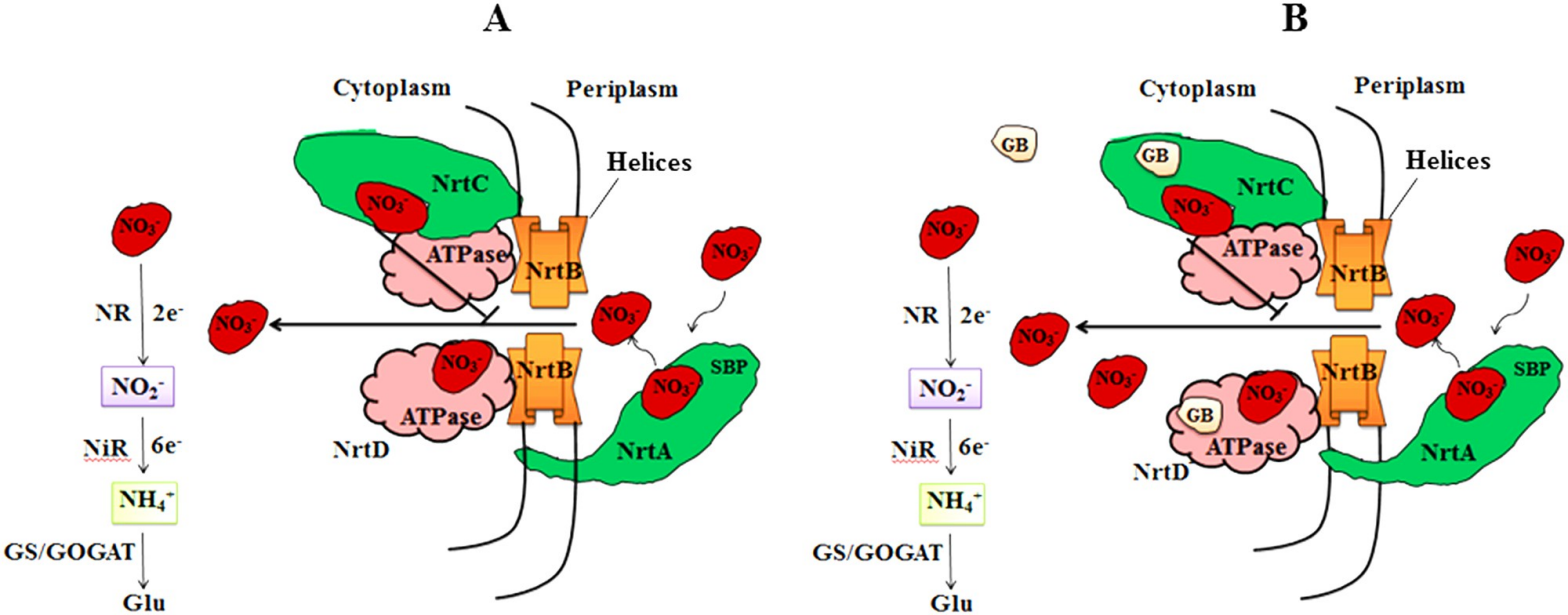
A schematic representation of nitrate transport. **(A)** The TMD proteins of the ABC-nitrate transporter, periplasmic subunit nrtA scavenges nitrate of the solution and transports across the lipid bilayer of the membrane through nrtB pore. When nitrate level rises in the cell, ABC protein, NrtC binds to nitrate and acts as negative regulator by inhibiting nitrate uptake, while nrtD binds and hydrolyzes ATP to induce conformational changes in TMDs (nrtAB). Extra cytoplasmic helices running parallel to the cytoplasmic membrane provide contact to TMDs proteins. **(B)** Interaction of GB with nrtACD proteins.

Nitrogen is a fundamental nutrient required by plants and microorganisms in high amounts and is often a limiting factor for their growth and yield. Except for N_2_-fixing prokaryotes and those living in association with them, the rest of the life forms depend upon combined forms of nitrogen viz., nitrate, nitrite, ammonium, and organic nitrogen. Nitrate is the most oxidized and stable form of combined-N, and is predominantly available as an immediate source of N for most of the plants and microorganisms in terrestrial and aquatic ecosystems. In the presence of nitrate, N_2_-fixing cyanobacteria cease the energy effective process of N_2_-fixation and switch over to utilizing available nitrate. However, nitrate concentration in natural environments is relatively low (< 0.01–50 μM) [1]. Hence, the initial step in nitrate assimilation, uptake of nitrate into the cell may become limiting. Cyanobacteria possess an active nitrate transport system to meet their N requirement [2]. The high-affinity ABC-nitrate transporter in cyanobacteria belongs to the ATP-binding cassette (ABC) super-family and comprises four domains (Fig 1); two ABC proteins: (1) a cytoplasmic ATPase (nrtD) and (2) ATPase/ nitrate binding fusion protein (nrtC), and two TMDs (transmembrane domains) proteins, (3) the periplasmic nitrate binding lipoprotein (nrtA) and (4) an integral membrane permease (nrtB) [3, 4]. The TMDs possess additional short extracytoplasmic binding proteins that provide critical contact to ABCs to function [5]. After entry into the cells, nitrate is reduced to nitrite and ammonium in two sequential enzymic reductions mediated by nitrate reductase and nitrite reductase, respectively, and then incorporated into carbon skeletons through GS/GOGAT (glutamine synthetase/ glutamine 2-oxoglutarate aminotransferase) pathway yielding amino acids.

In cyanobacteria, nitrate assimilation is directly linked to photosynthesis and is well documented [6]. In addition to the consumption of ATP during the uptake of nitrate, 8 e^-^ derived from photolysis of water are donated by ferredoxin to convert a molecule of nitrate into one ammonium molecule. However, most of the studies on nitrate assimilation are centered on the latter stages of nitrate assimilation, the first step being the transport of nitrate into the cell is the least studied [7]. Until recently, the mechanism of the ABC-nitrate transporter proteins in cyanobacteria has been overlooked. Salinity impairs nitrate uptake/ assimilation in cyanobacteria [6, 8]. Based on the kinetics and depressed transport of nitrate by an ionophore (monensin), sodium/nitrate symport has been proposed in *Anacystis nidulans* R2 [9, 10]. A mutant of *Anabaena* PCC 7120 defective in nitrate uptake could not take up Na^+^, indicated for NO_3_/ Na^+^ symport [11]. Conversely, no ABC-transporter driven by the electrochemical gradient of an ion has so far been reported. Furthermore, nitrate is noted to increase the adaptability of cyanobacterial cells to salinity by interacting with Na^+^ carrier and limiting Na^+^ influx [12–14]. To overcome the salt toxicity, halophilic and halotolerant forms synthesize/ accumulate low-molecular-weight organic compatible solutes. Glycinebetaine (*N*,*N*,*N*-trimethyl glycine), hereafter referred to as GB is a universal compatible solute synthesized by both prokaryotes and eukaryotes. In addition to maintaining cell osmolarity, GB is suggested to act as molecular chaperones protecting the native structure of proteins and membrane integrity [15].

In the present investigation, we used a freshwater diazotrophic cyanobacterium *Anabaena* PCC 7120 wild type (WT) and its transformant possessing N-methyltransferase genes (*ApGSMT-DMT*; glycine sarcosine methyltransferase and sarcosine dimethylglycine methyltransferase) from a halophilic cyanobacterium *Aphanothece halophytica* [6, 16]. The transformed cyanobacterium *de novo* synthesized and accumulated GB. We noted that salinity reduced the rate of nitrate transport in WT cells, while the rate increased in the *ApGSMT-DMT* transformed cells that synthesized recombinant GB. An attempt is made to unveil the mechanism(s) by which recombinant GB increases the efficiency of nitrate transporter system, and consequently its assimilation.

## 2. Materials and methods

Cyanobacterium *Anabaena* sp. PCC 7120 (hereafter referred to as *Anabaena* 7120; also known as *Nostoc* sp. PCC 7120) WT cells were grown and maintained in BG11_0_ medium [17]. The transformed *Anabaena* 7120 containing *ApGSMT-DMT* genes from *Aphanothece halophytica* (on the expression cassette pRL488 + ApGSMT-DMT) was grown in BG11_0_ + 0.1 M NaCl + Km^10^ (kanamycin 10 μg ml^-1^), since it was unable to grow without NaCl [6, 16]. The cultures were grown at 28 ± 1°C with an average illumination of 70 μE m^2^ s^1^ provided with daylight fluorescent lamps for 14 h a day.

### 2.1. Nitrate uptake

Exponential phase *Anabaena* 7120 WT and transgenic cells growing on N_2_ in the presence and absence of 0.1 M NaCl were harvested and suspended in their respective fresh media to get a cell density of 70–80 μg chl ml^-1^. To study the kinetics of nitrate uptake, the concentration of nitrate in the uptake mixture (nutrient solution with and without 0.1 M NaCl) was varied between 40–500 μM. The uptake experiments were performed under standard growth conditions (temperature 28 ± 1°C and light intensity 70 μE m^2^ s^1^), and samples (0.5 ml) were withdrawn at 10 min of incubation. Uptake of nitrate was determined by measuring the depletion of nitrate in the uptake mixture [18]. Since the uptake was linear up to 10 min, the initial uptake rate was determined as to nitrate taken up by the cells during the first 10 min. The assay was performed in triplicate with independent cultures.

### 2.2 Bioinformatics analysis

The amino acid sequences for the nitrate transporter proteins NrtA (accession no. BAB75032.1), NrtC (accession no. WP_010994786.1) and NrtD (accession no. WP_010994787) of *Nostoc* (*Anabaena*) 7120 were retrieved from National Centre for Biotechnology Information (NCBI) protein database, PDB (www.rcsb.org; http://www.ncbi.nlm.nih.gov/protein). The most suitable template for each protein was identified using BLASTP (protein BLAST, http://blast.ncbi.nlm.nih.gov/Blast.cgi) and selected for further bioinformatics approach. Protein domains, families and functional sites, patterns, and profile search were performed using PROSITE server [http://prosite.expasy.org/] [19–21]. The retrieved protein sequences were used for multiple sequence alignment using ClustalW (http://www.ebi.ac.uk/Tools/msa/clustalw2/). Sequences were aligned across the entire length or only in certain regions [22]. The top 100 sequences of each nrtA, nrtC and nrtD were taken for the phylogenetic analysis using PHYLIP (Phylogeny Inference Package) programs Seqboot, Protdist, Neighbour, and Consense to generate a phylogenetic tree [23–25]. Mega 5.0 and Tree view were used to view the phylograms [26].

### 2.3 Homology modeling

The nrtACD proteins of *Nostoc* PCC 7120 were used for template search on the NCBI PDB database for structure prediction [27, 28]. The selected templates were used for homology modeling employing Easy Modeller 4.0 [29]. It is a standalone tool with an intuitive interface that clearly defines the different steps of homology modeling for comparable protein structures based on MODELLER [30, 31]. Since no models were available for *Anabaena* 7120 nrtACD proteins in the PDB database, an approach was made for constructing their 3D structures using homology modeling. The validation of a generated model of nrtACD proteins was evaluated for stereochemical quality using assessment of Ramachandran Plot (RAMPAGE) server (http://mordred.boc.cam.ac.uk/~rapper/rampage.php), PROCHEK [32] and PDBSum [33–35]. The quality of the predicted model was calculated by the SAVES server (http://services.mbi.ucla.edu/SAVES/). Statistical analysis of non-bonded interactions among different atom types of the generated model was estimated using ERRAT server [36, 37], while compatibility of an atomic model (3D) with its own amino acid sequence (1D) was tested by Verify3D [37]. Potential errors in the 3D model and Z-score values were calculated and compared with a template by the ProSA-web server, which ensured overall quality as well as determined that the input structure is within the range of scores typically found for the native proteins of similar size [38]. After complete evaluation and visualization (Discovery Studio 3.0) sequence of the model is then deposited to PMDB (Protein Model Data Base) [39].

### 2.4 Molecular docking analysis

The chemical compounds GB (CID: 247) and nitrate (CID: 943) were retrieved using Pubchem compound database as SDF file [40] and converted to PDB using Discovery Studio Visualizer. The active sites and their residues for selected structures were identified using the MetaPocket server. The server identifies pockets on protein surfaces to predict ligand-binding sites [41, 42]. The retrieved PDB files for GB and nitrate were imported to the Patchdock server program (http://www.molegro.com/products.php) for docking with transporter proteins nrtACD models [43–45].

## 3. Results

**Fig 2:**
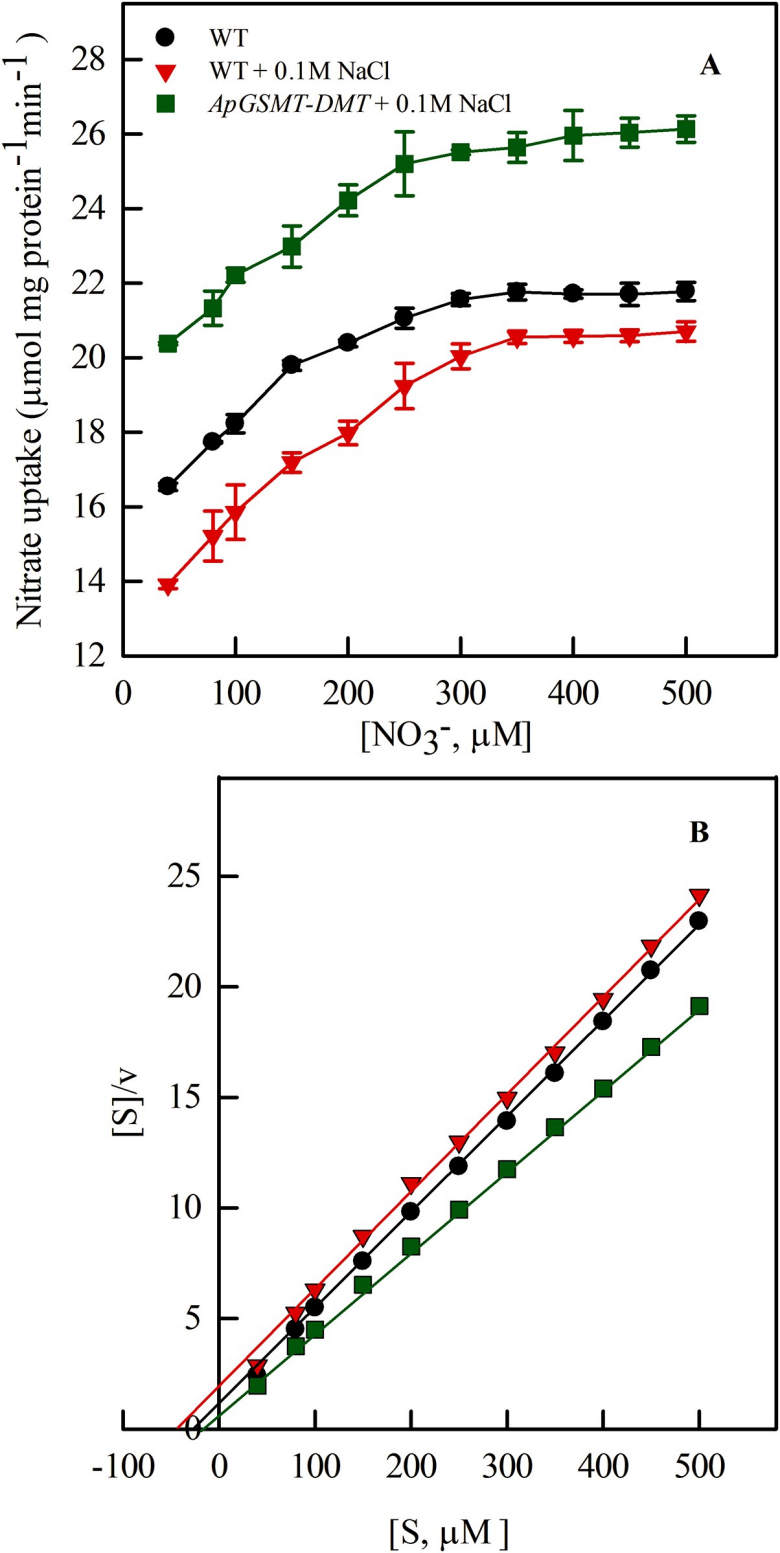
Nitrate uptake in *Anabaena* 7120, WT and *ApGSMT-DMT* transformant. Exponential phase cyanobacterial cells growing on N_2_ in the presence and absence of 0.1 M NaCl were collected and suspended in their specific media containing varied concentrations (40–500 μ) of nitrate. Uptake was performed under standard growth conditions in light (70 μE m^2^ s^1^). Samples were withdrawn at 10 min of incubation and measured nitrate depletion in the incubation mixture. Values represent means and SDs of triplicate experiments. **(A)** Initial rate of nitrate transport at different substrate concentrations; **(B)** Hofstee plot (v versus v/[S]) to determine the kinetics.

Salinity (0.1 M NaCl) reduced the rate of nitrate transport into the wild-type cells of *Anabaena* 7120. In contrast, under identical salinity, transport rate increased into the transgenic cells (transformed with *ApGSMT-DMT*) that synthesized and accumulated GB (Fig 2A). Determination of kinetics of the nitrate transport (Fig 2B) showed that salinity increased the *K*_*s*_ value (32.3μM) in WT, while the value was lowered (*Ks* 17.8 μM) in *ApGSMT-DMT* transformed cells compared to control (*K*_*s*_ = 19.5 μM), *i*.*e*., transport in absence of NaCl. This indicated the increased affinity of GB synthesizing cells towards nitrate [6]. The *V*_*max*_ value (26.8 μmol mg protein^-1^ min^-1^) was the highest for GB synthesizing cells, followed by control (22.6 μmol mg protein^-1^ min^-1^), and was the lowest (21.9 μmol mg protein^-1^ min^-1^) for NaCl-exposed cells. This revealed that salinization of the nutrient solution reduced the efficiency of the nitrate transport system, while GB stimulated it. Possibly, recombinant GB interacted positively with nitrate transporter proteins resulting in efficient nitrate transport. To unveil the mechanism of their interactions, we performed *in silico* analyses.

### 3.1. Sequence retrieval and analysis

Prosite analysis (S1 Fig) revealed that nrtA protein was 427 amino acids long and contained a Tat (twin-arginine translocation) signal profile (PS51318; score 8.195) of 34 amino acids (1–34) necessary to initiate the transport of folded proteins [46]. The Tat signal peptide consisted of three domains, the positively charged N-terminal domain, hydrophobic domain, and C-terminal domain. The majority of the amino acids were hydrophobic in nature.

Prosite results for nrtC protein revealed that it contains 657 amino acids (S2 Fig) and two functional motifs, ABC transporter 2 (5–239 amino acids) and ABC transporter 1 (139–153 amino acids) with Walker A (GHSGCGKS, 42–49) and Walker B (LLLD, 160–163), which is responsible for ATP binding and hydrolysis. Latter, it was preceded by an alpha-helical sub-domain and a signature motif (LSGGMKQR, 139–146). Sequence alignment results revealed that the nrtC subunit contained two distinct domains; one was an N-terminal domain (5–239 amino acids) mostly composed of hydrophobic residues, and the other C-terminal domain (268–591 amino acids) possessing mostly hydrophobic amino acids. N-terminal domain (5–228 amino acids) of nrtC showed 59% identity and 77% similarity with nrtD (the ATP-binding subunit), whereas the C-terminal domain (268–591 amino acids) was 28.2% identical and 50% similar to nrtA, suggesting that the C-terminal domain of nrtC was a solute binding domain and N-terminal as ATP binding.

NrtD protein was found 277 amino acids long and contained ATP binding motif (PS50893; score 26.484) located at N-terminal (21–254 amino acids) (S3 Fig). The majority of the amino acids were hydrophobic.

### 3.2. Sequence alignment and phylogenetic analysis

**Fig 3:**
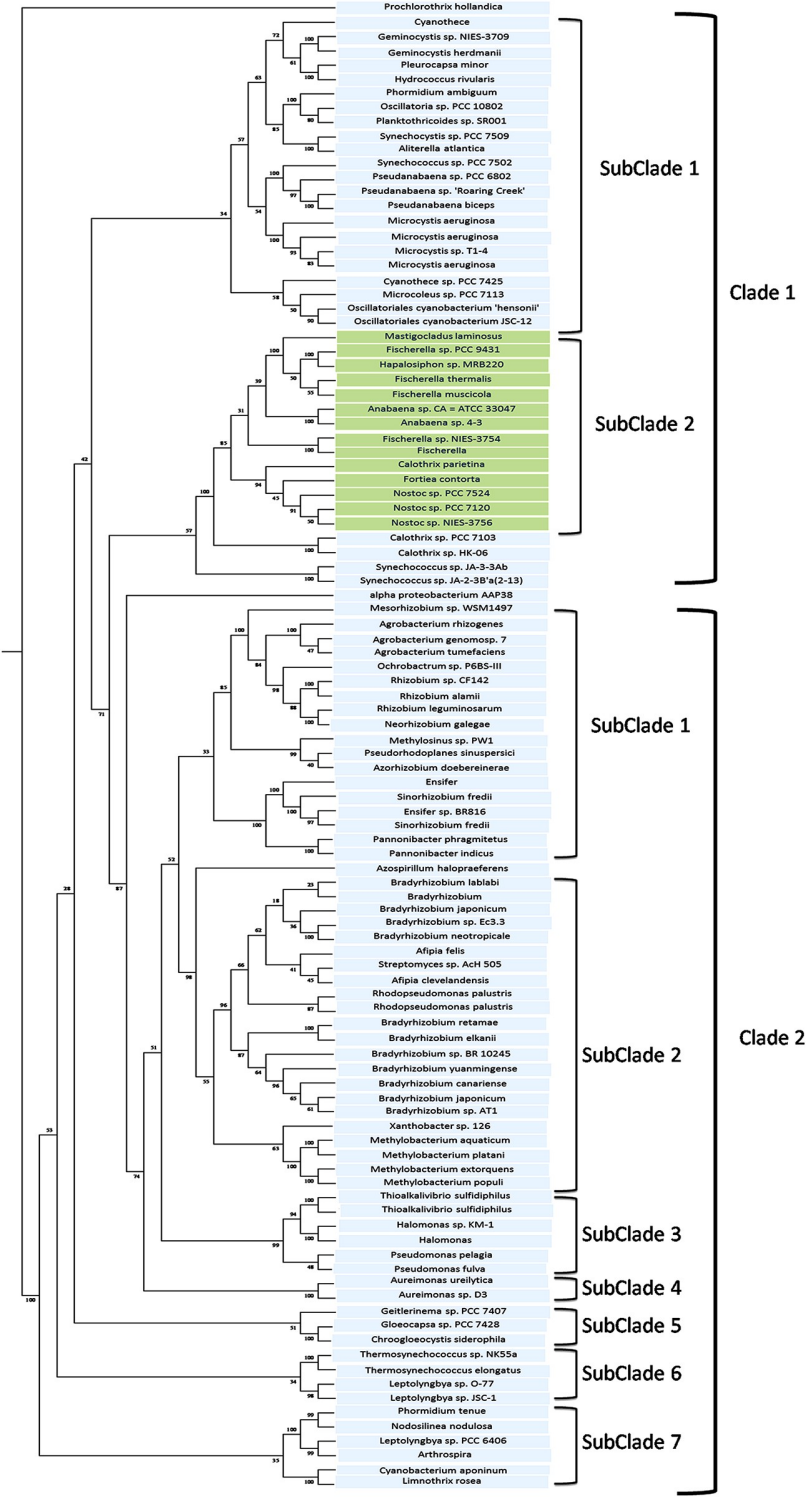
Phylogenetic tree of deduced nrtA protein. The tree was constructed by PHYLIP programs using Seqboot, Protdist, Neighbour and Consense methods. Bootstraping (1000 times) was performed to obtain support values for each branch. Values are shown at the nodes of each branch point.

**Fig 4:**
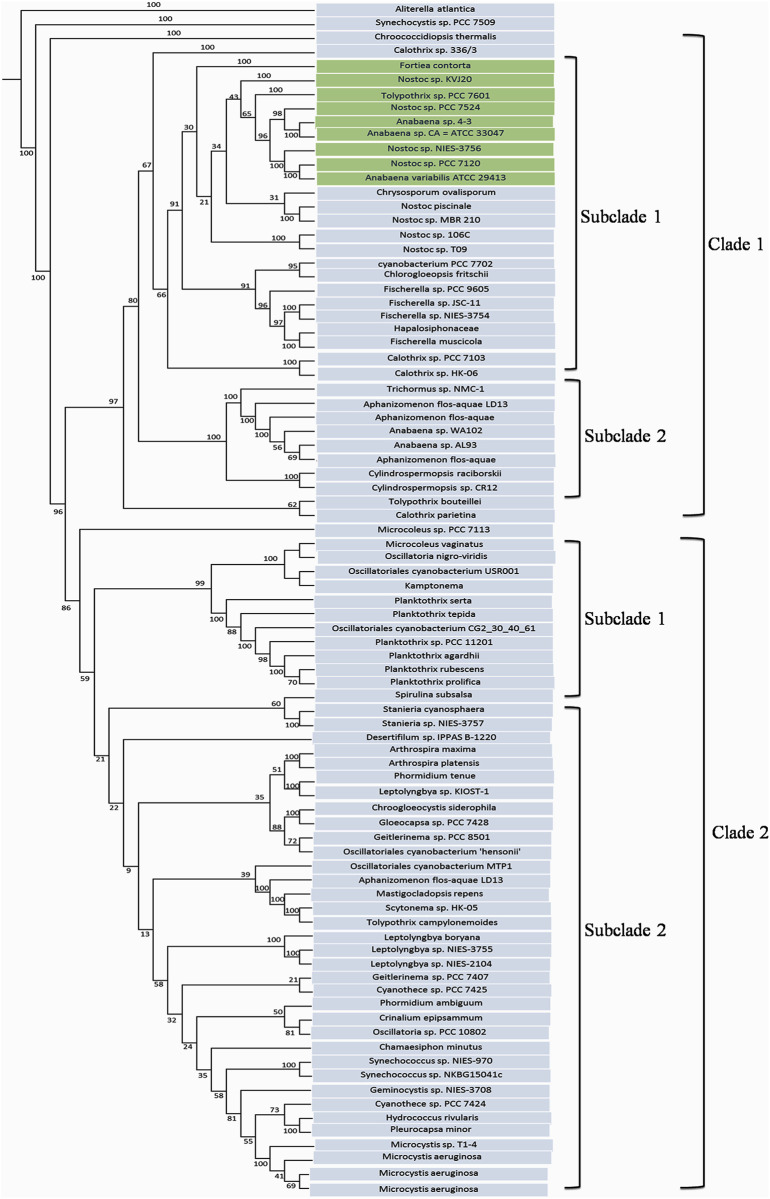
Phylogenetic tree of deduced nrtC protein. The tree was constructed by PHYLIP programs using Seqboot, Protdist, Neighbour and Consense methods. Bootstraping (1000 times) was performed to obtain support values for each branch. Values are shown at the nodes of each branch point.

**Fig 5:**
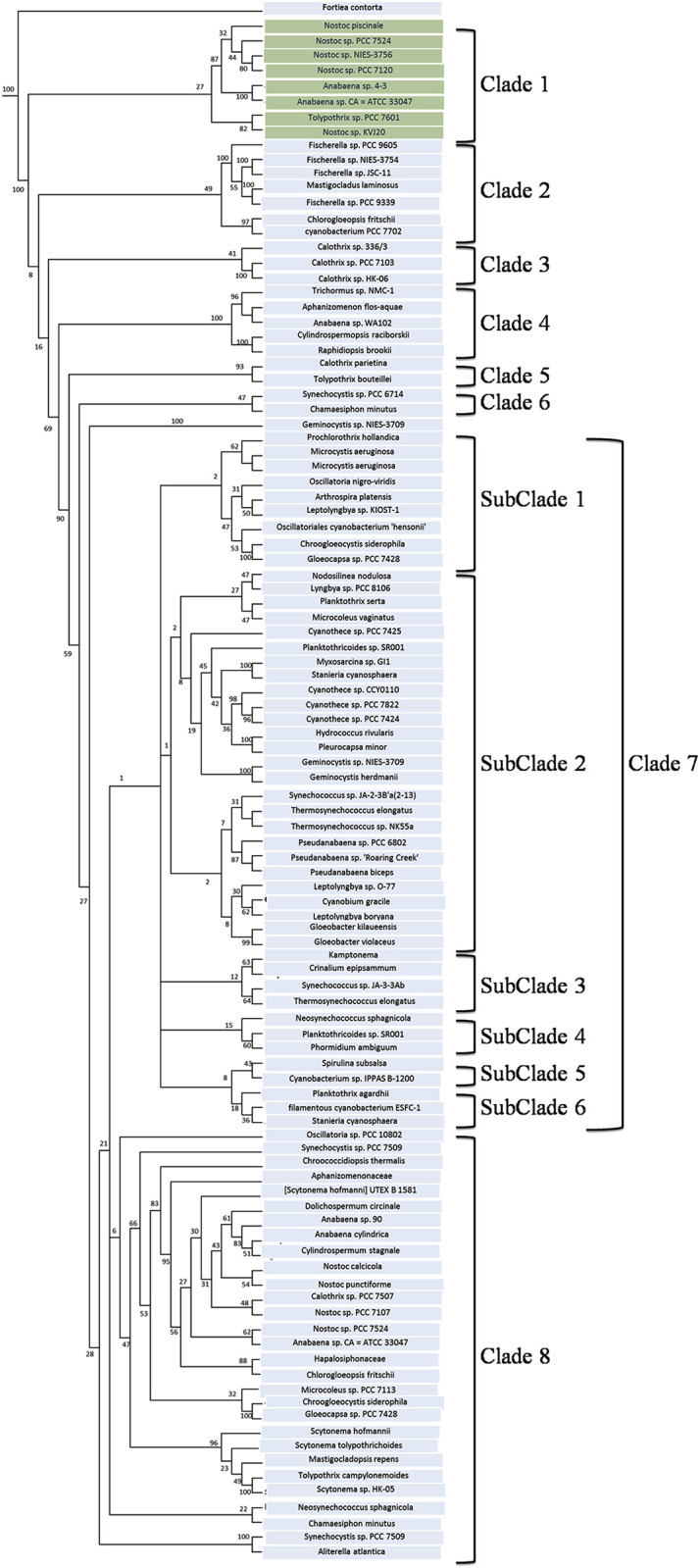
Phylogenetic tree of deduced nrtD protein. The tree was constructed by PHYLIP programs using Seqboot, Protdist, Neighbour and Consense methods. Bootstraping (1000 times) was performed to obtain support values for each branch. Values are shown at the nodes of each branch point.

The nrtACD protein sequences of *Nostoc* 7120 (target organism) were taken as input to find out the similar protein sequences in different cyanobacterial species using BLASTP. The searche against non-redundant data base and protein data bank resulted in 103, 86 and 106 homologs for nrtA, nrtC and nrtD, respectively (S1–S3 Tables), and were used for multiple sequence alignment (S4–S6 Figs). The phylogenetic analysis of nrtA, nrtC and nrtD revealed different clustering with respect to cyanobacterial nrt proteins. The nrtA cladogram (Fig 3) shows two clades in which clade 1 comprises two subclades and clade 2 comprises seven small subclades. The two subclades of clade 1 are differentiated with one having *Anabaena* and *Nostoc* species and the other containing the *Microcystis* and *Gemnocyctis* species. The subclades one, two, three and six of clad 2 showed *Agrobacterium* and *Rhizobium*, *Bradyrhizobium* species, *Thioalkalivibrio* and *Psuedomonas*, *Thermosynechococcus* and *Leptolyngbya* as dominating species in the phylogenetic tree, respectively. The nrtC phyllogram (Fig 4) also has two clades each having two subclades comprising of *Anabaena*, *Nostoc*, *Fischerella*, *Planktothrix*, *Leptolyngbya* and *Microcystis* species. Phylogeny of nrtD protein showed eight clades (Fig 5), where clade seven had six subclades. The phylogenetic tree was comprised of *Nostoc*, *Anabaena*, *Fischerella* and *Calothrix* species. Clade one was dominated by the presence of *Nostoc* and *Anabaena* species.

Species like *Hapalosiphon* sp., *Mastigocladus laminosus* and *Calothrix* sp. had close homologues of nrtA protein, *Tolipothrix* sp. PCC of both the nrtC and nrtD proteins, while *Fortiacontorta* species appeared to have a close homologue of all three nrtACD proteins of *Anabaena*. Overall phylogenetic results thus indicated that the nitrate-ABC-transporter proteins (nrtACD) homology is highly conserved among the cyanobacterial species.

### 3.3. Modeling of nrtACD proteins of Nostoc sp. PCC 7120 and validation of 3D homology

**Fig 6:**
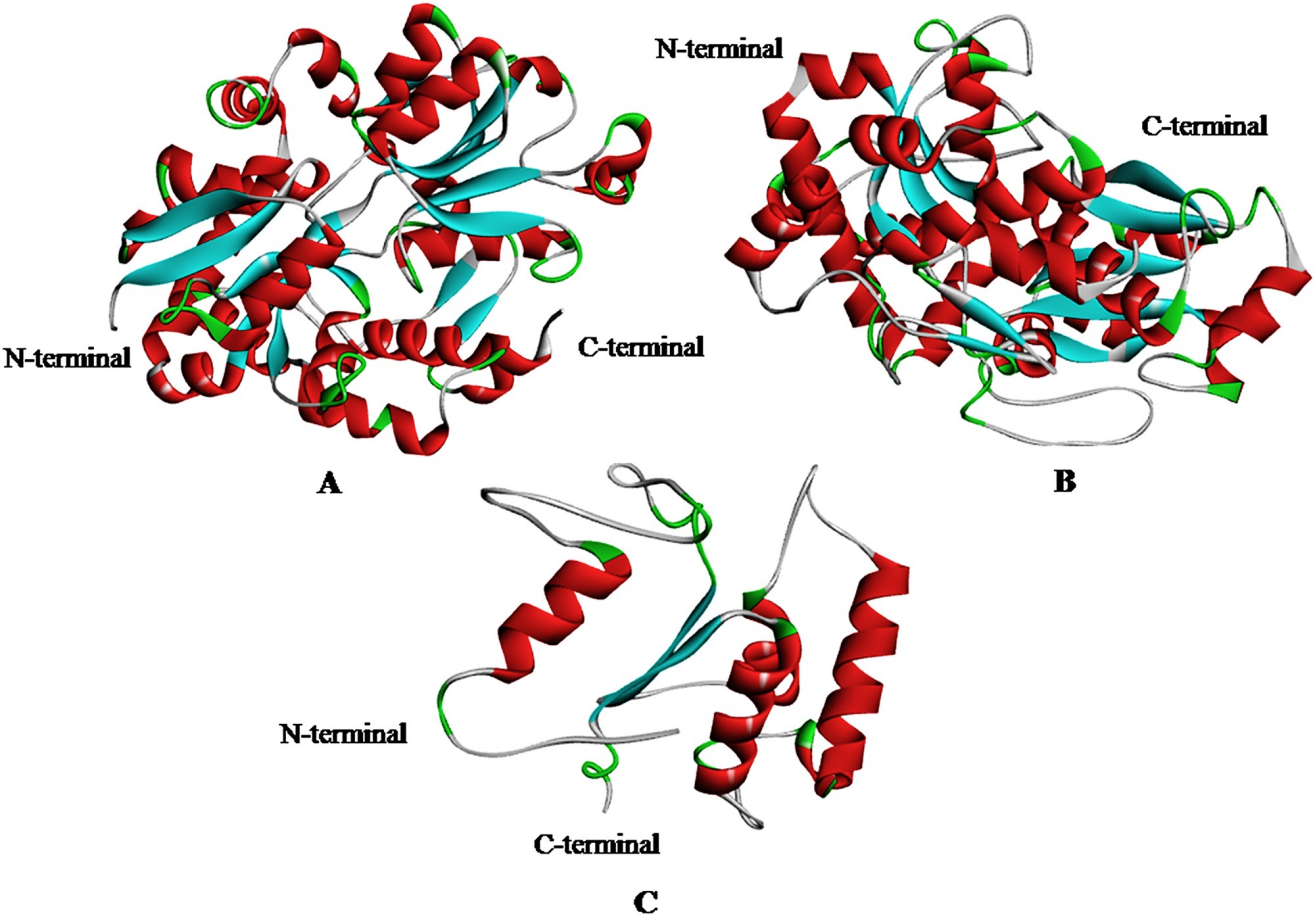
Homology model of nrtACD proteins. Models are presented as ribbon structure. Alpha helices are in red, beta sheets in cyan, turn in grey and coil in green color. **(A)** nrtA, **(B)** nrtC and **(C)** nrtD.

**Fig 7:**
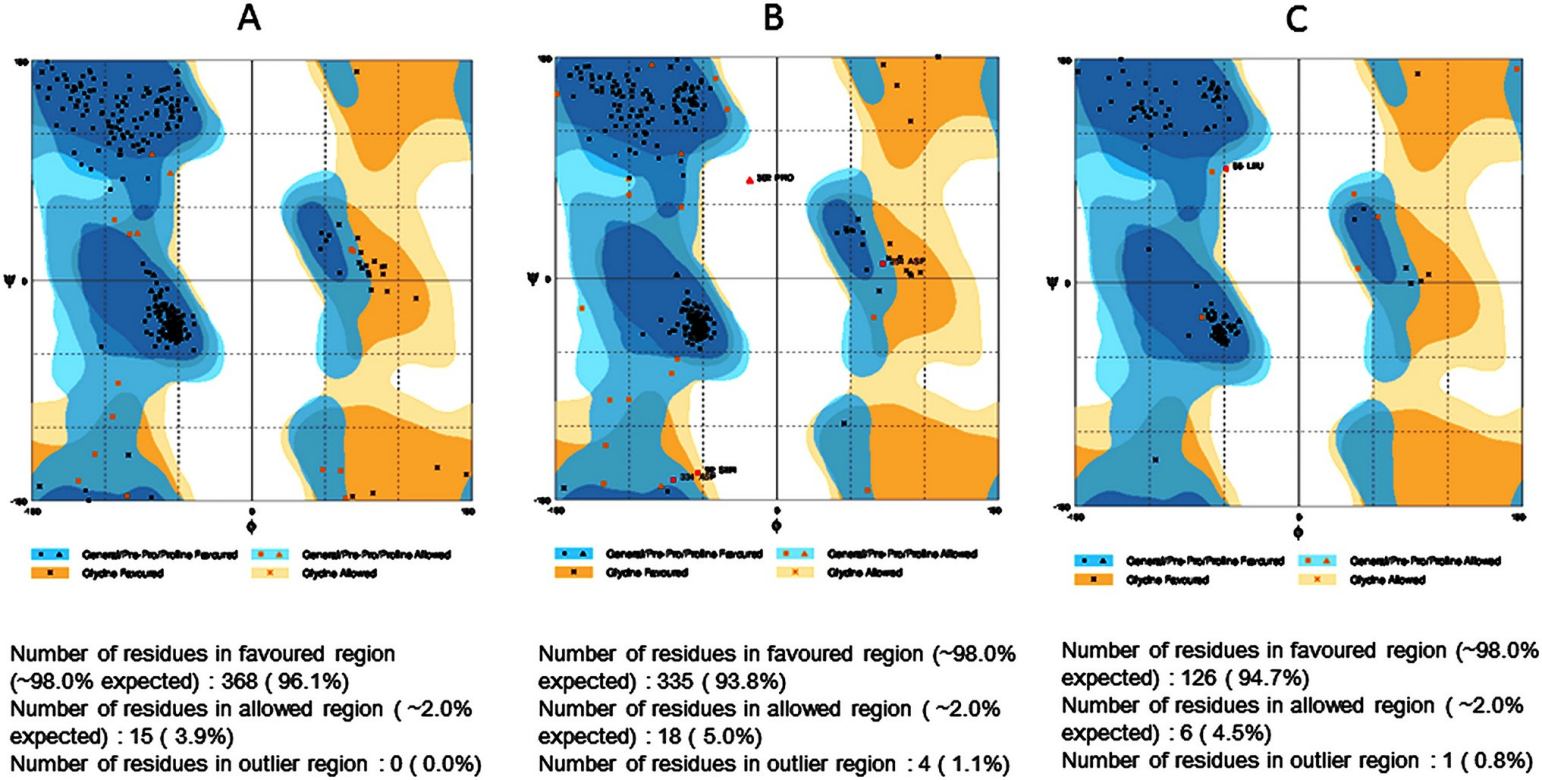
RAMPAGE analysis of nrtACD proteins. Values indicate number of the residues in favored, allowed, and outlier region. **(A)** nrtA, **(B)** nrtC and **(C)** nrtD.

**Fig 8:**
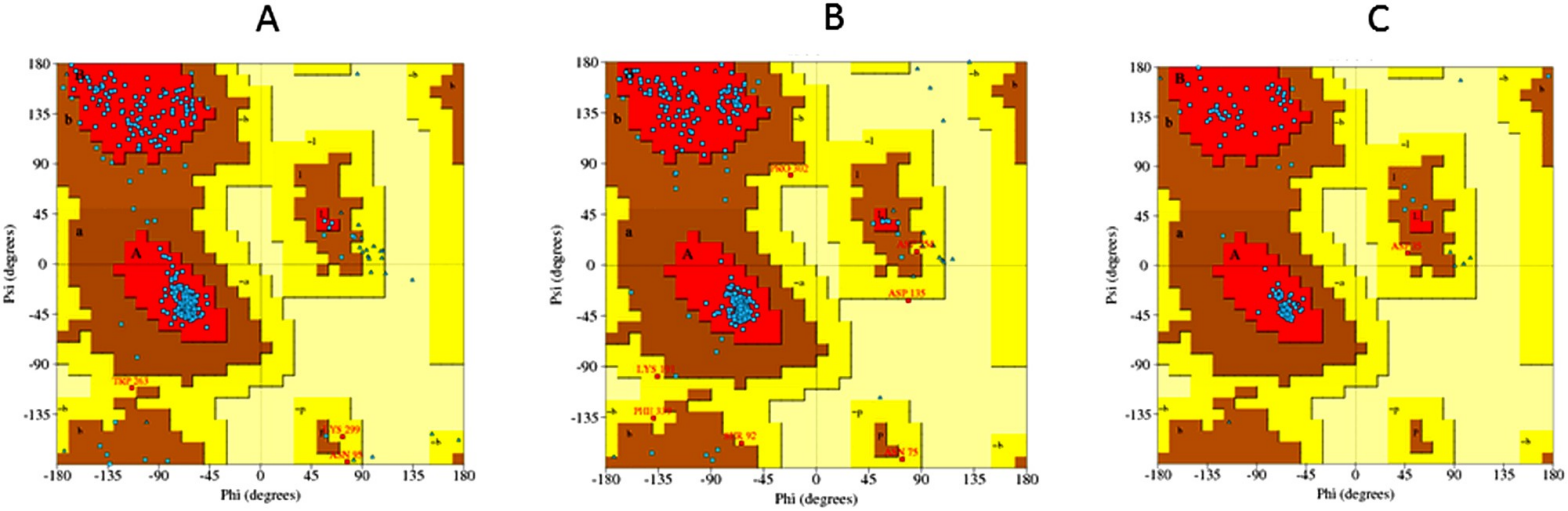
Ramachandran plot analysis of traget proteins nrtACD. Red regions in the graph indicate the most allowed regions and yellow the allowed region. **(A)** nrtA, **(B)** nrtC and **(C)** nrtD.

**Fig 9:**
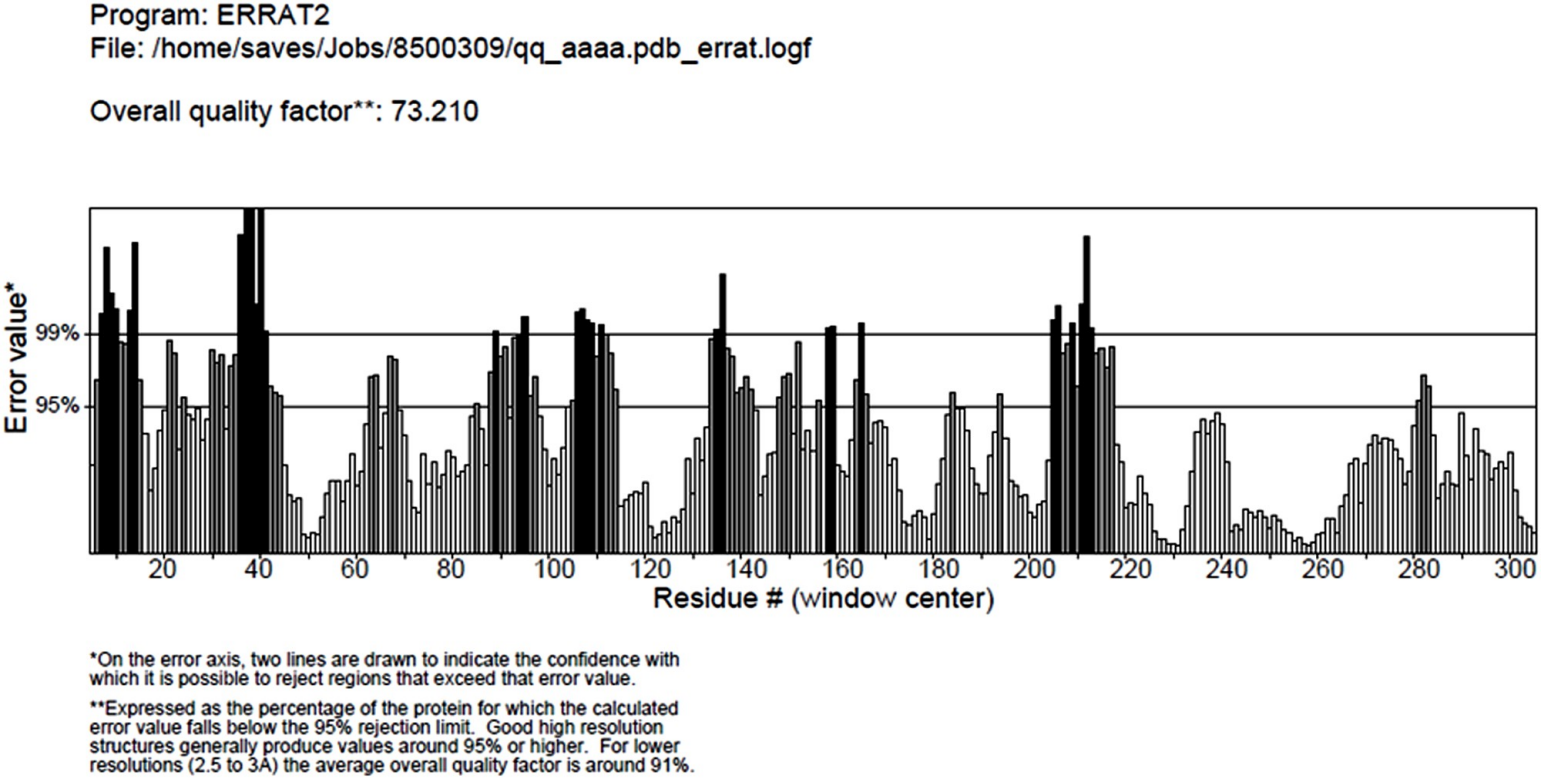
ERRAT measurement of nrtA protein. Values indicate over all structure quality of the traget protein.

**Fig 10:**
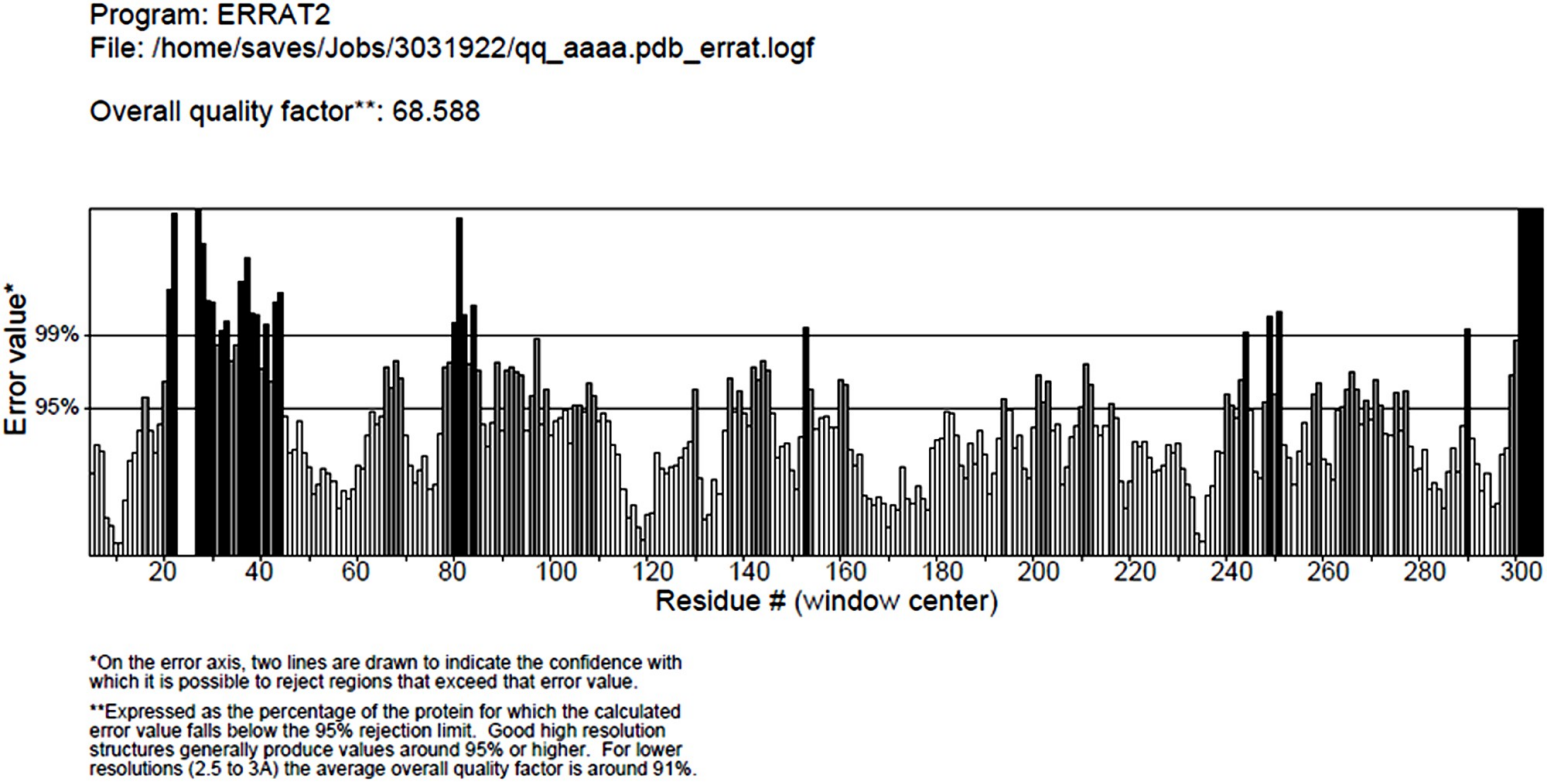
ERRAT measurement of nrtC protein. Values indicate over all structure quality of the traget protein.

**Fig 11:**
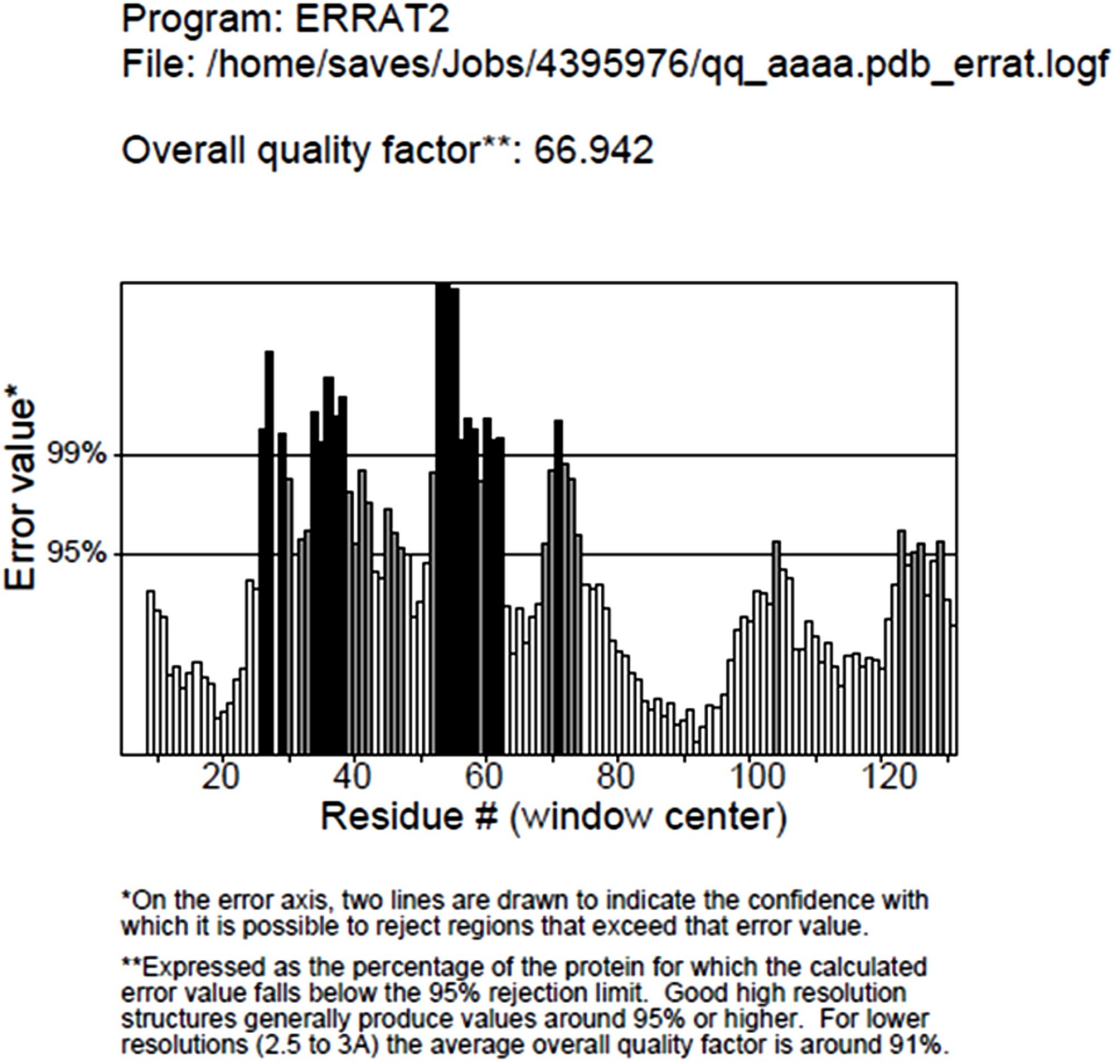
ERRAT measurement of nrtD protein. Values indicate over all structure quality of the traget protein.

**Fig 12:**
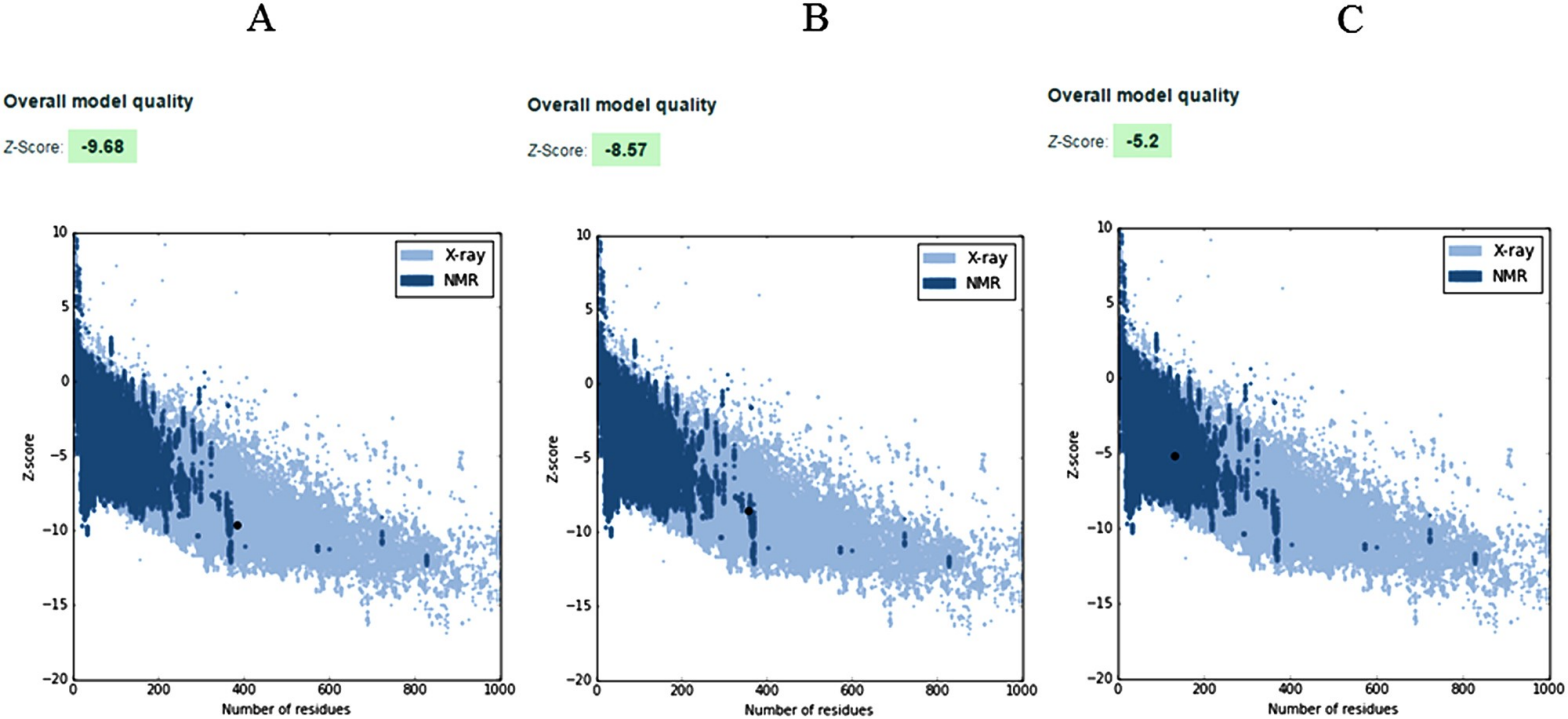
ProSA analysis for nrtA (A), nrtC (B), nrtD (C) proteins. Z-scores of the protein chains in PDB were determined by X-ray crystallography (light blue) and NMR spectroscopy (dark blue). The proSA web results indicated that protein structures had characteristic features of their native structures. The Z-score for all the three target proteins was highlighted as a large dot.

The target proteins (nrtACD) of *Nostoc* sp. PCC 7120 were used for 3D structure prediction using templates (Fig 6A–6C). Details of similarity for each selected template are presented (Table 1). The results obtained by RAMPAGE for stereochemical quality of nrtA showed that 368 residues (96.1%) lies in the favored region, 15 residues (3.9%) lies in the allowed and none in the outliner region (Fig 7A). For nrtC protein, 335 residues (93.8%) lies in the favored region, 18 residues (5.0%) lies in the allowed region and 4 residues (1.1%) lies in the outliner region (Fig 7B), and for nrtD, 126 residues (94.7%) lies in the favored region, 6 residues (4.5%) lies in the allowed region and 1 residue (0.8%) lies in the outliner region (Fig 7C). PROCHEK output in the form of Ramachandran plot for nrtA showed 304 residues (91.8%) lies in the most favored region, 24 residues (7.3%) lies in the additional allowed region, 3 residues (0.9%) lies in generously allowed regions and none in the disallowed region (Fig 8A). For nrtC protein, 281 residues (90.6%) lies in the most favored region, 23 residues (7.4%) lies in the additional allowed region, 5 residues (1.6%) lies in generously allowed regions and 1 residue (0.3%) lies in the disallowed region (Fig 8B), whereas for nrtD, the 102 residues (90.3%) lies in the most favored region, 10 residues (8.8%) lies in the additional allowed region, 1 residues (0.9%) lies in generously allowed regions and none in the disallowed region (Fig 8C). ERRAT web server was used to check the overall quality factor of nrtACD proteins which was found 73.21 in nrtA (Fig 9), 68.58 in nrtC (Fig 10) and 66.94% in nrtD (Fig 11) protein models, while the Verify 3D was used to determine the compatibility of atomic model (3D) with its own amino acid sequence (1D) by assigning a structural class which is based on its position and environment (alpha, beta, loop, polar and nonpolar), and the compatibility was found more than or equal to 0.2 averaged 3D-1D score for the 95.84, 95.26 and 89.92% amino acid residue of NrtA, NrtC and NrtD, respectively. Hence, more than 80% amino acid score with the template protein demonstrated the good quality of the model proteins. The overall model quality was checked by the ProSA-web tool [35] by determining their Z-score values. Z-score value for nrtA, NrtC and NrtD was found -9.68 (Fig 12A), -8.57 (Fig 12B) and -5.2 (Fig 12C), respectively. The results taken together suggested a close similarity between the template and the predicted structures and reinforced a good quality of the modeled protein.

**Table 1:** Similarity between template and target proteins nrtACD of ABC-transporter based on identities and positives of model proteins.

Target protein	Templates PDB ID	Identities	Positives
nrtA	2G29	50	70
	2I48	41	61
	2I49	41	61
nrtC	2G29	32	52
	2I48	31	51
	2I49	31	51
nrtD	1Z47	41	60
	2IT1	40	62
	1G29	37	58

### 3.4. Molecular docking

**Fig 13:**
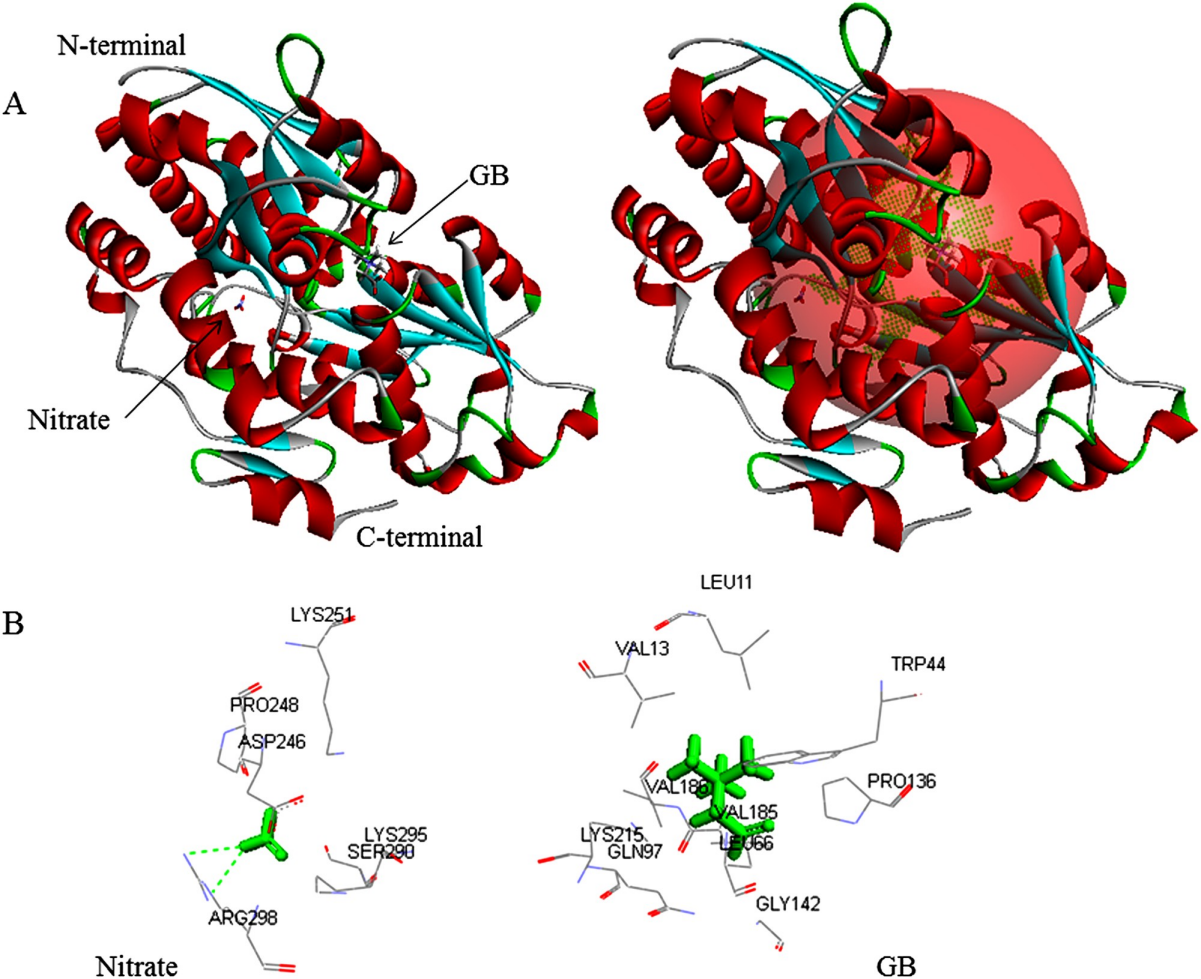
Docking of ligands GB and nitrate with nrtA protein. **(A)** Green color in the sphere indicates prominent active site with which the ligand interacted. **(B)** 3D level interaction.

**Fig 14:**
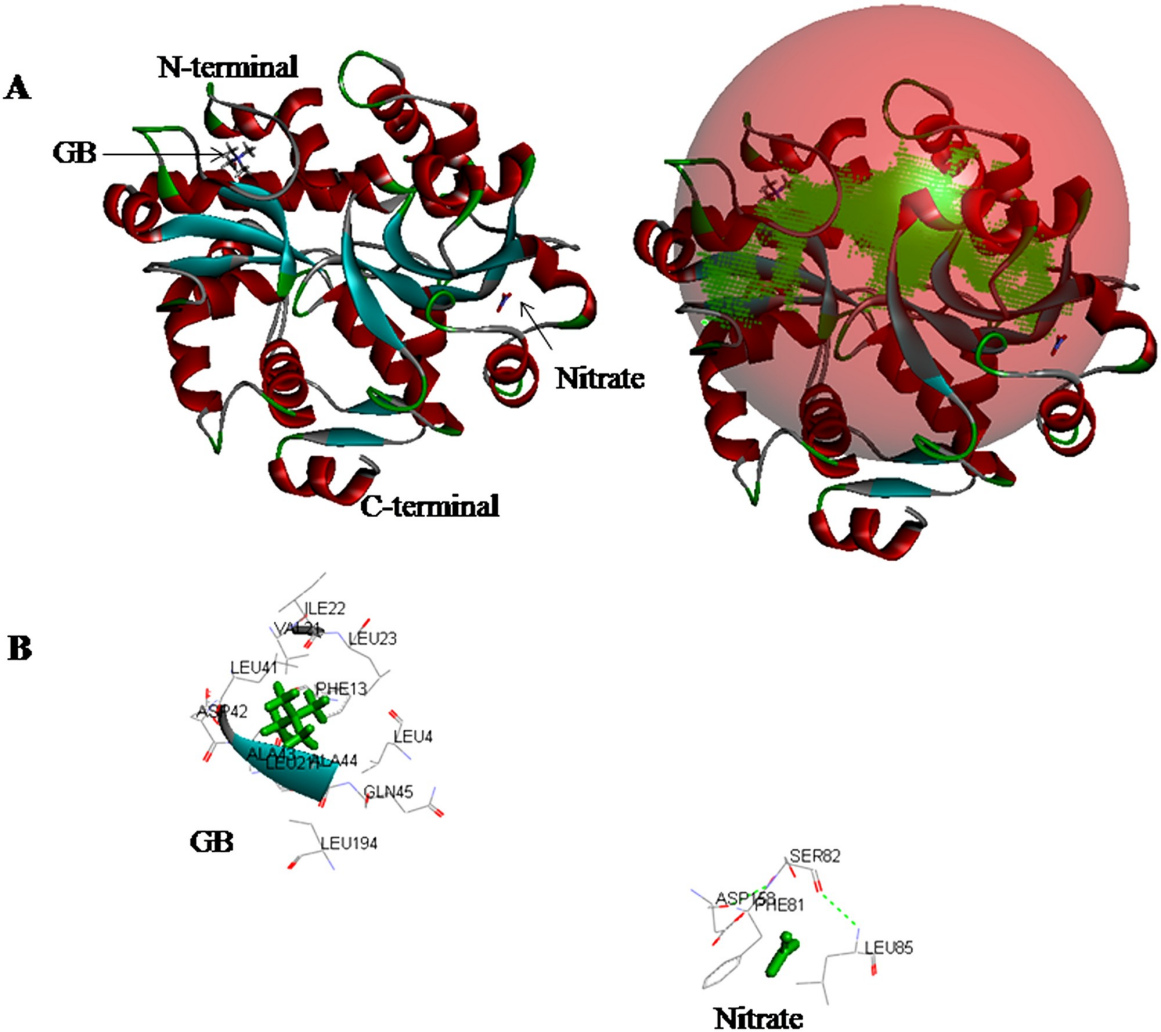
Docking of ligands GB and nitrate with target protein nrtC. **(A)** Poses of docked complexes; green color in the sphere indicates prominent active site on which the ligand interacted. **(B)** 3D level interaction.

**Fig 15:**
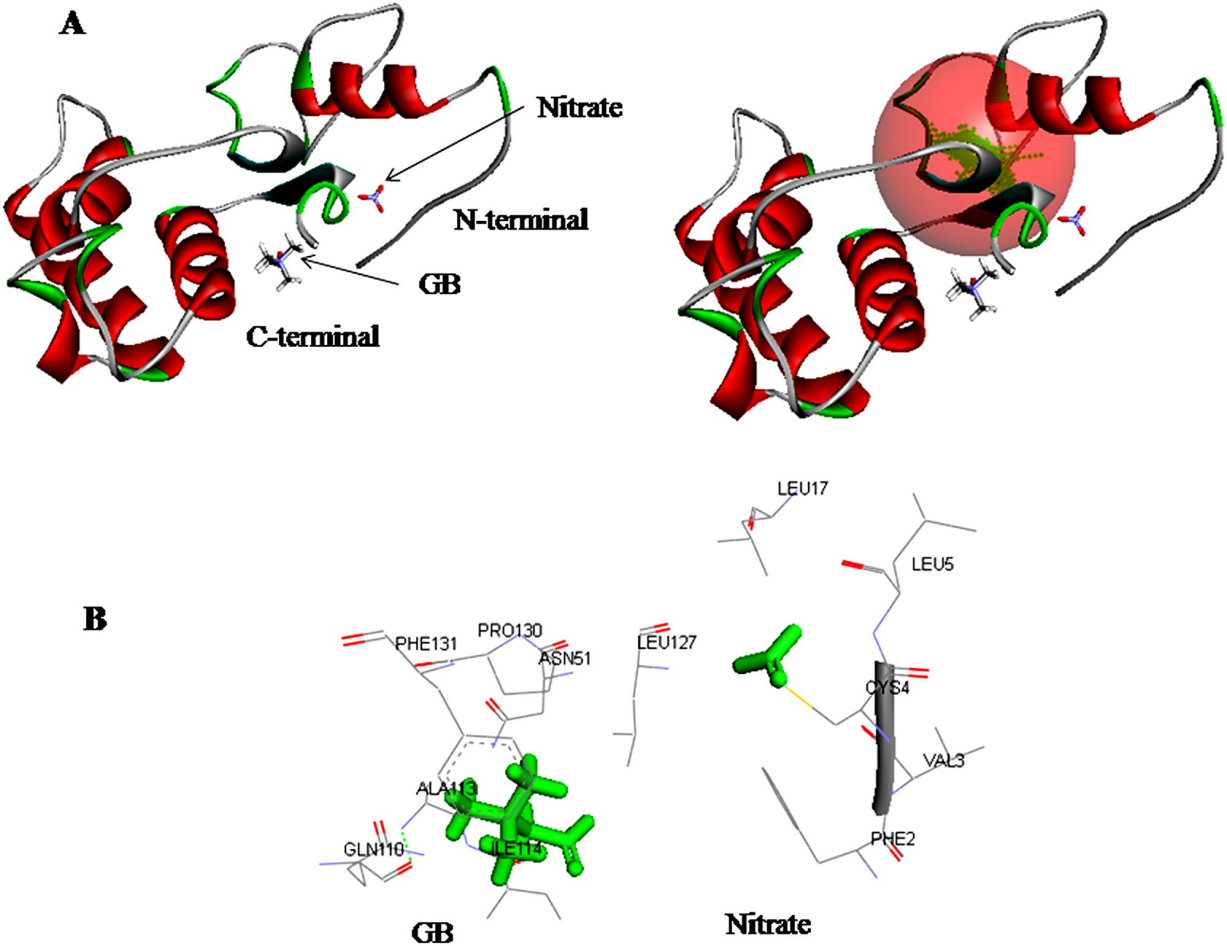
Docking of ligands GB and nitrate with target protein nrtD. **(A)** Poses of docked complexes; green color in sphere indicates prominent active site, on which the ligand interacted. **(B)** 3D level interaction.

Metapocket server predicted three possible active sites in nrtACD proteins (Table 2). Active site one in the nrtA protein (Fig 13A) and active site two in nrtC (Fig 14A) and nrtD proteins (Fig 15A) appeared as the prominent binding sites (Table 2). The polar hydrophilic and positively charged amino acids Lys^251^, Lys^295^and Arg^298^, neutral Ser^290^, and negatively charged Asp^246^, and hydrophobic neutral amino acid Pro^48^ of nrtA protein interacted in docking with nitrate (Fig 13B). The majority of the molecular interactions were hydrophilic with H-bonding between Arg298:NH1-O2, and Arg298:NH1-O2. On the other, the majority of the molecular interactions of nrtA with GB were hydrophobic. Five aliphatic hydrophobic neutral amino acid residues (Leu^11^, Leu^66^, Val^13^, Val1^85^ and Val^186^) along with hydrophobic neutral (Pro^136^), polar hydrophobic neutral (Gln^97^), polar hydrophilic positively charged (Lys^215^), aliphatic neutral (Gly^142^) and aromatic hydrophobic neutral (Trp^44^) amino acid residues interacted in the absence of H-bond (Fig 13B).

**Table 2:** The potential ligand binding catalytic active sites of target proteins (nrtACD) calculated with METAPOCKET server to perform molecular docking.

Target protein	Header binding site		
	Active site 1	Active site 2	Active site 3
**nrtA**	Gln^97^, Val^135^, Pro^136^, Gly^142^, Cys^184^, Val^185^, Trp^44^, Leu^66^, Val^186^, Thr^141^, Lys^215^, Leu^11^, Val^13^, Pro^168^, Trp^189^, His^64^, Ser^15^, Ser^43^, Pro^12^, Pro^169^, Pro^42^, Ala^167^, Lys^40^, Gln^41^, Pro^188^, Arg^192^, Ile^264^, Val^266^, Leu^270^, Thr^14^, Gly^265^, Val^165^, Ile^166^, Tyr^137^, Ala^45^, Lys^267^, Trp^263^, Phe^145^, Thr^271^, Val^258^, Ser^259^, Ile^20^, Asp^187^, Cys^17^, Leu^275^, Leu^255^, Arg^274^, Tyr^296^, Leu^254^, Ile^294^	Thr^14^, Gly^96^, Asp^187^, Ile^294^, Tyr^296^	Asp^325^, Phe^326^, Lys^329^, Pro^330^, Leu^310^, Ile^332^, Lys^306^, Asp^333^, Pro^373^, Ser^307^, Glu^374^, Thr^328^, Pro^376^, Thr^358^, Ser^359^, Asn^375^
**nrtC**	Tyr^204^, Thr^207^, Leu^18^, Trp^200^, Asn^210^, Asn^20^, Leu^211^, Val^21^, Asp^42^, Ile^22^, Leu^4^, Phe^13^, Leu^194^, Val^212^, Leu^215^, Val^36^, Ala^43^, Tyr^208^, Val^196^, Leu^23^, Ala^44^, Lys^8^, Asn^24^, Leu^41^, Lys^40^, Gln^45^, Ser^1^, Tyr^240^, Arg^25^, Ala^2^, Ile^231^, Leu^235^, Phe^252^, Phe^273^, Pro^3^, Ser^73^, Asn^271^, Leu^72^, Lys^192^, Val^248^, Ile^5^, Trp^29^, Ala^26^, Ala^31^, Ile^32^, Ala^76^, Asn^75^, Leu^241^, Lys^191^, Met^46^, Gly^251^, Tyr^270^, Leu^232^, Gln^30^, Tyr^256^, Glu^164^, Pro^165^, Asn^77^, Asn^28^, Met^50^, Leu^54^, Ala^33^, Gly^163^, Gln^119^, Asn^27^, Ser^60^, Thr^181^, His^114^, Gly^59^, Pro^255^, Trp^166^, Pro^145^, Thr^146^, Thr^62^, Asn^167^, Ala^162^, Val^243^, Val^113^, Ala^53^, Ala^58^, Asp^242^, Pro^144^, Gln^169^	Lys^303^,Asn^304^,Glu^307^,Ile^308^, Leu^56^, Pro^300^, Thr^299^, Ile^290^, Ala^58^, Trp^305^, Leu^298^, Glu^310^, Arg^311^, Gly^57^, Gln^61^, Pro^63^, Ser^60^, Thr^55^, Ala^53^, Met^293^, Val^306^, Pro^349^, Ala^294^, Pro^302^, Thr^291^, Phe^301^, Ile^350^, Leu^353^, Leu^287^, Arg^359^, Pro^346^, Ser^347^, Glu^348^	Ile^79^, Arg^91^, Trp^126^, Ala^180^, Tyr^125^, Glu^184^, Ile^185^, Phe^339^, Asp^340^, Ser^92^, Ala^129^, Leu^93^, Asn^77^, Ala^78^, Ile^122^, Cys^161^, Ala^182^, Thr^181^, Ala^94^, Gly^130^, Trp^186^, Ser^187^, Leu^338^, Leu^183^, Gly^341^, Arg^258^
**nrtD**	Ala^70^, Val^71^, Asp^69^, Lys^75^, Ile^83^, Arg^121^, His^87^, Ser^119^, Glu^79^, Ile^120^, Val^68^, Lys^80^, lu^86^, Ala^67^, Asp^44^, Arg^45^, Met^47^, Tyr^65^, Met^90^, Leu^118^, Pro^122^, Leu^66^, Val^48^, Met^46^, Pro^43^, Phe^49^, Asn^63^, Val^64^, Arg^116^, Gln^50^, Tyr^52^, Phe^23^, Ile^126^, Asn^51^, Ser^21^, Pro^56^, Ala^117^, Leu^125^, Pro^76^, Val^84^, Ala^115^, Leu^88^	Ile^20^, Val^124^, Leu^125^, Leu^17^, Ile^126^, Val^3^, Leu^5^, Leu^16^, Glu^1^, Phe^2^, Ser^21^, Met^46^, Val^48^, Lys^13^, Ser^14^, Leu^127^, Cys^4^, Glu^129^, Pro^130^, Phe^131^, Leu^33^, Asp^128^, Ile^6^, Gly^7^, Gln^123^, Ile^114^, Leu^118^, Ala^117^, Asn^51^, Gln^110^, Ala^113^, Phe^49^, Pro^101^, Ser^102^, Ile^104^, Lys^109^, Pro^56^, Trp^57^, Arg^111^, Met^108^, Ser^105^, Leu^55^, Gly^107^, Val^31^, Leu^32^, Tyr^52^, Arg^116^, Leu^134^, Ala^133^, Cys^53^, Gly^132^, Asp^135^	Met^108^, Lys^109^, Gln^110^, Leu^134^, Pro^101^, Ile^104^, Gly^107^, Leu^55^, Pro^56^, Trp^57^, Ala^133^, Ser^102^, Asp^135^, Asn^51^, Leu^127^, Pro^130^, Ile^114^, Ala^117^, Leu^125^, Ala^113^, Phe^131^, Tyr^52^, Cys^53^, Leu^118^, Arg^111^

Green color amino acid residues are located on the prominent active site.

In nrtC/nitrate docking, the majority of the amino acids involved were hydrophobic and interacted in the presence of H-bond (Fig 14B). Polar hydrophilic negatively charged (sp^158^, aromatic hydrophobic neutral Phe^81^, polar hydrophobic neutral Ser^82^ and aliphatic hydrophobic neutral Leu^85^ amino acids were buried in the center with H-bond between Ser^82^:N-Asp^158^:O, and Leu^85^:N-Ser^82^:O. In the interaction of GB with nrtC, the bulk of the amino acids were hydrophobic. Aliphatic hydrophobic neutral (Leu^23, 41, 4, 21, 194^, Val^21^, Ala^43^, Ala^44^ and Ile^22^), aromatic hydrophobic neutral (Phe^13^), and polar hydrophilic charged (Asp^42^) and polar hydrophilic neutral (Gln^45^) amino acid residues bind with GB (Fig 14B). H-bond was present between Ala^43^:N-Asp^42^:OD1, Ala^44^:N-Gly^195^:O and Leu^85^:N-Lys^83^:O amino acid residues.

The results of nrtD/nitrate docking showed that the major portion of the amino acid residues that interacted with nitrate was hydrophobic and contains aliphatic hydrophobic neutral Leu^17^, Leu^5^ and Val^3^, polar hydrophobic neutral Cys^4^ and aromatic hydrophobic neutral Phe^2^ with hydrogen bond in between Cys^4^: SG-O_2_ (Fig 15B). Similarly, the majority of nrtD amino acids interacting with GB were hydrophobic involved aliphatic hydrophobic neutral (Leu^127^, Ala^13^ and Ile^114^), polar hydrophobic neutral (Asn^51^), aromatic hydrophobic neutral (Phe^131^ and Pro^130^) and polar hydrophilic (Gln^110^) with H-bond between (Tyr^52^:N-:Asn^51^:OD1, Ala^113^:N-:GLN^110^:O and Ile^114^:N-:Arg^111^:O) (Fig 15B). PatchDock server docking calculation for the ligands GB and nitrate with the receptor proteins nrtACD presented a higher score solution, larger acquired docking area and lesser atomic contact energy (ACE) for GB compared to nitrate (Table 3), revealing a higher affinity of GB towards receptor proteins compared to nitrate.

**Table 3:** PatchDock server molecular docking results of GB and nitrate with ABC transporter proteins nrtACD.

S.No.	Docking molecule		Score	Area	Atomic contact energy (ACE)	Transformation
	Receptor proteins	Ligands compounds				
1.	nrtA	nitrate	1042	115.90	31.75	2.73, -0.19, -2.87, 4.75, 6.07, and 28.20.
2.	nrtA	GB	2700	281.80	-95.38	0.61, 0.05, -2.59, 11.47, -0.34, and -4.89.
3.	nrtC	nitrate	1116	130.10	-29.93	-0.61, 1.07, -1.56, -6.92, 3.67, and 52.41.
4.	nrtC	GB	2712	295.10	-100.64	1.11, 0.40, -2.62, -25.12, 26.77, and 39.55.
5.	nrtD	nitrate	1002	126.70	-46.14	3.02, -1.32, -0.72, 45.88, 32.36, and 12.95.
6.	nrtD	GB	2270	256.80	-70.10	0.72, -0.96, -2.23, 22.41, 35.32, and 24.79.

## 4. Discussion

Nitrate (NO_3_^−^) is the main nitrogen source for plants and their mobilization is regulated by various transporters in the plant body [47]. To transport nitrate into the cell, nrtA protein of ABC-transporter binds to nitrate and delivers it to the nrtB (Fig 1). NrtA has a high-affinity with the nitrate ABC transporter. The passage of nitrate transportation through the pore is linked to ATP binding and its consequent hydrolysis on the P-loop of the ABC domain. This brings conformational changes in the transmembrane domains and allows nitrate to cross the lipid bilayer of the membrane. Short cytoplasmic coupling helices provide critical contacts to TMD with ATP domain [4, 5, 48]. When nitrate concentration becomes high in the cell, nrtC binds to nitrate allosterically and inhibits nitrate transport [49, 50].

Salinity reduced the nitrate transport into WT cells (Fig 2), while the rate of transport was increased into the transgenic cells (synthesizing recombinant GB). This means that in addition to protecting nitrate transport from salt toxicity, recombinant GB increased the efficiency of nitrate transport system. This increased efficiency might be due to the interaction of GB with the nitrate transporter proteins. NaCl stress increases Na^+^ concentration in the cells, which disrupts the water structure and reduces hydrophobic interactions and hydrostatic forces within proteins [51]. Hence, NaCl-induced inhibition of nitrate transport is due to the disturbed hydrophobic–electrostatic balance necessary to maintain the protein structure [52, 53]. Compatible solute GB can interact with both the hydrophilic and hydrophobic regions of macromolecules [54] and can replace water at the surface of proteins [55] enabling the Nrt proteins to stabilize their native structure.

*In silico* analyses revealed that the deduced periplasmic protein nrtA in *Anabaena* 7120 that traps nitrate of the ambient solution was 427 amino acids long, the majority of which were hydrophobic as reported for *Synechococcus* sp. PCC7942 [56]. The molecular interaction of nrtA with nitrate (Fig 13B) was hydrophilic (involving Lys^251^, Lys^295^and Arg^298^, Ser^290^, Asp^246^, and Pro^48^ with H-bonding at Arg^298^). The protein contained a Tat signal profile of 34 amino acids (1–34) necessary to initiate the transport [46]. Tat protein transport system has been reported in most bacteria and archaea. In cyanobacteria, Tat consensus motifs have been reported in deduced protein PhoD of *Aphanothece halophytica* exhibiting alkaline phosphatase (assimilate phosphorous under salt stress) and phosphodiesterase activities [57]. The presence of Tat motifs also supports the capturing of nitrate by pushing nrtA off the membrane and passing to the nrtB [4]. Since one nrtA protein binds to one molecule of nitrate [58], nrtA becomes the most abundant protein in the nitrate-grown cells [59].

The N-terminal (5–239 amino acids) and C-terminal (268–591 amino acids) of nrtC protein of *Anabaena* 7120 contained 657 amino acids that are mostly hydrophobic. Therefore, the interaction of nrtC with nitrate was also found hydrophobic. The hydrophilic negatively charged amino acid residues (Asp^158^, Phe^81^, Ser^82^ and Leu^85^) were buried in the center with H-bond between Ser^82^:N-Asp^158^:O, and Leu^85^:N-Ser^82^:O (Fig 14B). The nrtC protein in S*ynechococcus* sp. PCC 7942, is reported to be 659 amino acids long with the deduced amino acid sequences 1–254 for the N-terminal domain, and 279–659 for the C-terminal domain [56]. In *Phormidium laminosum*, the N-terminal (1–262) contained mostly hydrophobic residues, while the C-terminal (281–626) was highly hydrophilic [60]. In *Anabaena* 7120, the N-terminal of nrtC contained two functional motifs, ABC transporter 2 (5–239 amino acids) and ABC transporter 1 (139–153 amino acids) consisting of Walker A (GHSGCGKS, 42–49) and Walker B (LLLD, 160–163) similar to that reported in *Phormidium laminosum* [58], and was responsible for ATP binding and hydrolysis.

The C-terminal domain (268–591 amino acids) of nrtC in *Anabaena* 7120 was 28.2% identical and 50% similar to nrtA. In *Synechocystis* sp. PCC 6803, C-terminal domain was >30% identical and 50% similar to nrtA [4], while in *Synechococcus* sp. PCC 7942, the identity of C-terminal domain of nrtC (amino acid residues 279–659) to nrtA was 30% [3, 56]. This further affirmed that the C-terminal domain of nrtC protein is a solute-binding domain. The deduced protein NrtD of *Anabaena* 7120 was of 277 amino acids, the majority of which were hydrophobic. The interaction of nrtD with nitrate was found hydrophobic and included Leu^17^, Leu^5^ Val^3^, Cys^4^, and Phe^2^ with hydrogen bond between Cys^4^: SG-O_2_ (Fig 15B). The N-terminal domain (5–228 amino acids) showed 59% identity and 77% similarity with nrtD (the ATP-binding subunit). At N-terminal 21–254 amino acid sequences were ATP binding motif. In *Synechococcus* sp. PCC7942, the nrtD protein consisted of 274 amino acids and showed 58% identity to the N-terminal domain of nrtC [3, 56]. This protein domain of the ABC transporter is responsible to bind and hydrolyze ATP to energize the nitrate transport system.

Ammonium is preferred over nitrate when available in the solution and inhibits nitrate uptake [49, 61]. When intracellular nitrate level goes up, the C-terminal domain of nrtC, which is structurally similar to nrtA, may bind to nitrate allosterically as an effector-binding domain. Hence, nrtC role is suggested as a negative regulator involved in the ammonium-promoted inhibition of nitrate transport activity [49, 62]. The interactions of GB with deduced nrtACD proteins of *Anabaena* PCC 7120 were hydrophobic and showed higher affinity towards GB compared to nitrate. Further, docking of GB was more efficient (with maximum docking score) with nrtC than that of nrtA and nrtD. Among the osmoprotectants, GB is by far the most effective and the most characterized osmolyte to date [63], allowing the cells to grow luxuriantly under salt stress. The level of GB in transgenic *Anabaena* 7120 was low (7.5 μmol g^−1^ dry weight) and insufficient to contribute significantly to cellular osmotic potential, yet GB conferred considerable tolerance to salt-stress [6]. Other than the osmoprotective role, the mechanisms put forth to explain the GB enhanced stress tolerance include: (1) stabilization of proteins native structure and maintenance of membrane integrity, and (2) a regulatory function in the expression of genes *i*.*e*., induction of specific genes that led to increased stress tolerance [64, 65], including photosynthetic and respiratory electron transport [6]. It suggested that both the roles of GB, stabilization of protein native structure and regulatory function in gene expression are responsible for the efficient nitrate transport in GB synthesizing and accumulating transgenic *Anabaena* PCC 7120.

Salinity reduces the thermodynamic activity of water, thereby displacing the water of hydration from the protein surfaces [66] causing protein unfolding. GB induces the folding of unfolded (inactive) proteins *in vitro* and *in vivo* in a chaperone-like manner by decreasing the exposure of protein surface to solvent by drastically decreasing the water activity [66]. Experimentation regarding *in situ* evidence of the GB interaction with the proteins is difficult, hence scanty [67]. However, the periplasmic protein ProX of the ATP-transport system and ProU of *Escherichia coli* is reported to bind GB with high affinity [68, 69]. The receptor protein ProX undergoes a conformational change to form a site/cavity to accommodate GB and has an evenly negative surface potential [70]. The two conserved cis-proline align the Trp65 and Trp188 residues in a way to form an almost rectangular box in the polypeptide backbone of tryptophan residues [70, 71]. Possibly, nitrate transporter proteins nrtACD bind with GB in a similar fashion resulting in efficient nitrate transport. To our knowledge, this is the first report of GB interaction with ABC-nitrate transporter proteins and may open the way for further experimentation.

## 5. Conclusion

Salinity is one of the major factors limiting plant metabolic activities, growth, biomass and yield. GB is a universal osmoprotectant synthesized by prokaryotes and eukaryotes, including some crop plants to counter various stresses including salinity, and is also beneficial to humans. Nitrogen is the most limiting nutrient for plant growth and is often available as nitrate-N in the natural ecosystems, though at μM concentrations. The cyanobacterium *Anabaena* 7120 transported nitrate into the cells through an active process mediated by ABC-transporter proteins nrtABCD. The *ApGSMT-DMT* transformed cells possessed Ń-methyltransferase activities and *de novo* synthesized and accumulated intracellular recombinant GB. Salinity reduced the transport of nitrate into the cells. The recombinant GB not only protected the nitrate transport into the cells but increased the rate of transport under salinity. The low amount of intracellular GB (7.5 μmol g^−1^ dry weight) was insufficient to act as a cellular osmoticum. It is noted that the increased rate of nitrate transport in GB accumulating cells was due to the interaction of GB with ABC-nitrate transporter proteins and also by the participation of GB in the regulation of other intracellular metabolic processes. Thus, the results provided important bearing on the mechanism of GB interaction with ABC-nitrate transporter proteins.

## Supporting information

S1 Fig Prosite analysis of nrtA-nitrate transporter protein. nrtA protein showing functional motif Tat signal profile (PS51318).
(TIF)

S2 Fig Prosite analysis of nrtC-nitrate transporter protein. nrtC protein showing two functional motifs, ABC transporter 2 (5–239 amino acids) and ABC transporter 1 (139–153 amino acids) consisting Walker A (GHSGCGKS, 42–49) and Walker B (LLLD, 160–163) responsible for ATP binding and hydrolysis.
(TIF)

S3 Fig Prosite analysis of nrtD-nitrate transporter protein. nrtD protein showing ATP binding motif (PS50893).
(TIF)

S4 Fig Multiple sequence alignment of target protein nrtA of *Nostoc* sp. PCC 7120. (PDF)

S5 Fig Multiple sequence alignment of target protein nrtC of *Nostoc* sp. PCC 7120. (PDF)

S6 Fig Multiple sequence alignment of target protein nrtD of *Nostoc* sp. PCC 7120. (PDF)

S1 Table Sequence retrieval details of nrtA protein for multiple sequence alignment and phylogenetic tree construction showing accession number, proteins, organisms and name proposed. (DOC)

S2 Table Sequence retrieval details of nrtC protein for multiple sequence alignment and phylogenetic tree construction showing accession number, proteins, organisms and name proposed. (DOC)

S3 Table Sequence retrieval details of nrtD protein for multiple sequence alignment and phylogenetic tree construction showing accession number, proteins, organisms and name proposed. (DOC)

## Abbreviations

ABC: ATP-binding cassette
GB: Glycinebetaine
MEGA: Molecular Evolutionary Genetics Analysis
N_2_: Dinitrogen
NaCl: Sodium Chloride
NCBI: National Centre for Biotechnology Information
Nrt: Nitrate transporter
pBLAST: Basic Local Alignment Search Tool
PDB: Protein Data Bank
PHYLIP: PHYLogeny Inference Package
PMDB: Protein Model Data Base
ProSA: Protein Structure Analysis
TMDs: Transmembrane domains
UPGMA: Unweighted Pair Group Method with Arithmetic Mean
WT: Wild type
