# Supplementary figures and images for: Molecular interaction of nitrate transporter proteins with recombinant glycinebetaine results in efficient nitrate uptake in the cyanobacterium Anabaena PCC 7120
Source: PLoS One. 2021 Nov 18;16(11):e0257870. doi: 10.1371/journal.pone.0257870 (PMC8601584; doi:10.1371/journal.pone.0257870)

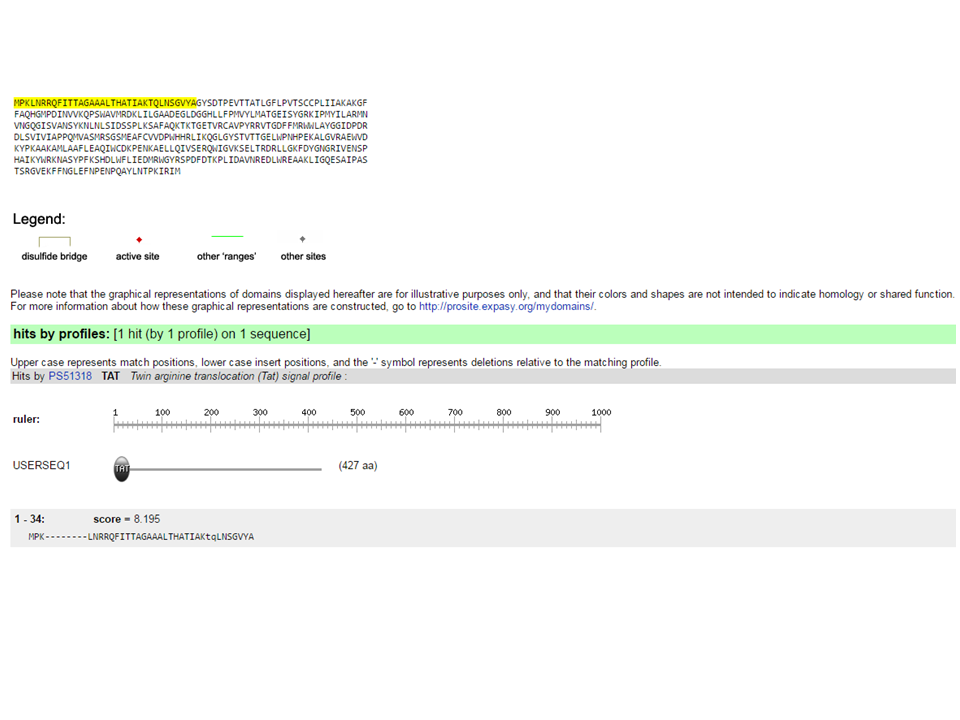

Supplement: S1 Fig — nrtA protein showing functional motif Tat signal profile (PS51318). (TIF) [file pone.0257870.s001.tif]

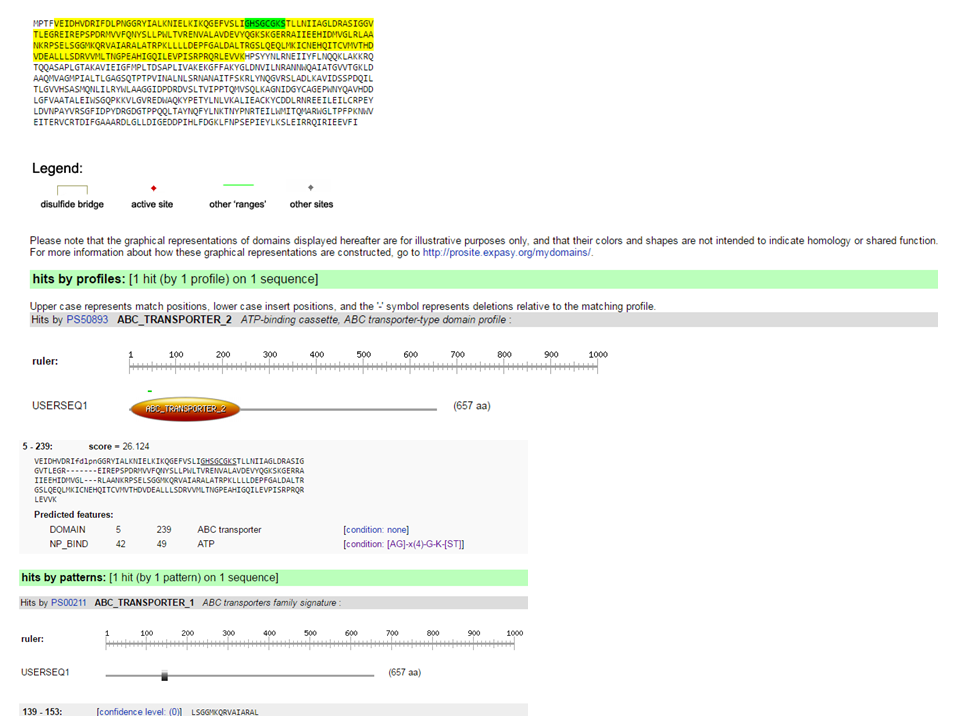

Supplement: S2 Fig — nrtC protein showing two functional motifs, ABC transporter 2 (5–239 amino acids) and ABC transporter 1 (139–153 amino acids) consisting Walker A (GHSGCGKS, 42–49) and Walker B (LLLD, 160–163) responsible for ATP binding and hydrolysis. (TIF) [file pone.0257870.s002.tif]

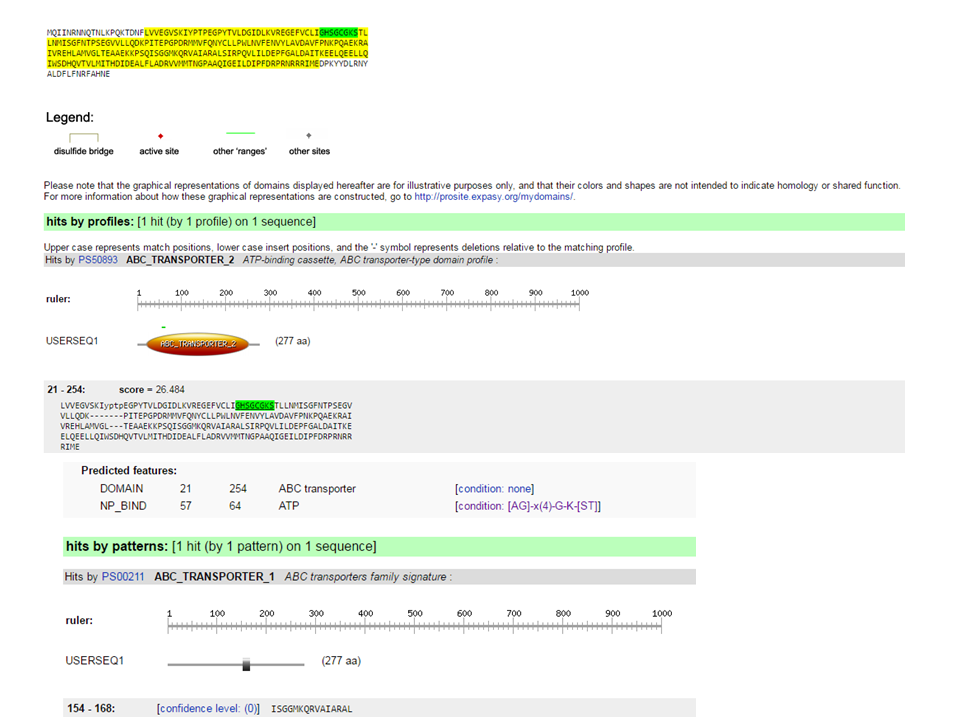

Supplement: S3 Fig — nrtD protein showing ATP binding motif (PS50893). (TIF) [file pone.0257870.s003.tif]
