# Supplementary material for: Molecular interaction of nitrate transporter proteins with recombinant glycinebetaine results in efficient nitrate uptake in the cyanobacterium Anabaena PCC 7120
Source: PLoS One. 2021 Nov 18;16(11):e0257870. doi: 10.1371/journal.pone.0257870 (PMC8601584; doi:10.1371/journal.pone.0257870)
Supplement: S4 Fig — (PDF) [file pone.0257870.s004.pdf]

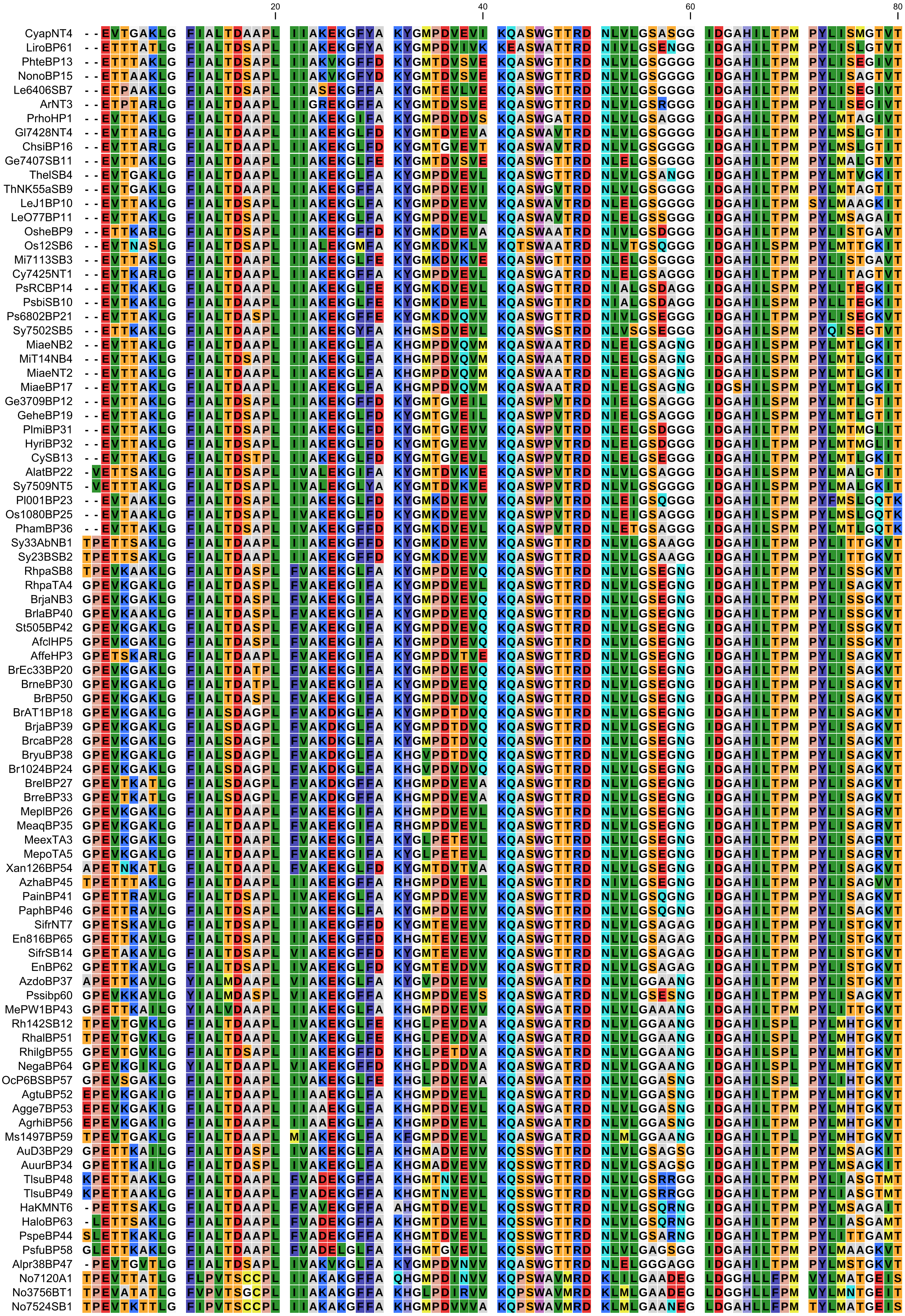
20
40
60
80
CyapNT4 --EVTGAKLG FIALTDAAPL IIAKEKGFYA KYGMPDVEVI KQASWGTTRD NLVLGSASGG IDGAHILTPM PYLISMGTVT
LiroBP61 --ETTTATLG FIALTDSAPL IIAKEKGFYA KYGMPDVIVK KEASWATTRD NIVLGSENGG IDGAHILTPM PYLISLGTVT
PhteBP13 --ETTTAKLG FIALTDSAPL IIAKVKGFFD KYGMTDVSVE KQASWGTTRD NLVLGSGGGG IDGAHILTPM PYLISEGIVT
NonoBP15 --ETTAAKLG FIALTDSAPL IIAKVKGFYD KYGMTDVSVE KQASWGTTRD NLVLGSGGGG IDGAHILTPM PYLISAGTVT
Le6406SB7 --ETPAAKLG FIALTDSAPL IIASEKGFFA KYGMTEVLVE KQASWGTTRD NLVLGSGGGG IDGAHILTPM PYLISEGIVT
ArNT3 --ETPTARLG FIALTDAAPL IIGREKGFFA KYGMTDVSVE KQASWGTTRD NLVLGSRGGG IDGAHILTPM PYLISEGIVT
PrhoHP1 --EVTTAKLG FIALTDAAPL IIAKEKGIFA KYGMPDVDVS KQASWGATRD NLVLGSAGGG IDGAHILTPM PYLMTAGIVT
Gl7428NT4 --EVTTARLG FIALTDAAPL IIAKEKGLFD KYGMTDVEVA KQASWAVTRD NLVLGSGGGG IDGAHILTPM PYLMSLGTIT
ChsiBP16 --EVTTARLG FIALTDAAPL IIAKEKGLFD KYGMTGVEVT KQASWAVTRD NLVLGSGGGG IDGAHILTPM PYLMSLGTIT
Ge7407SB11 --EVTTAKLG FIALTDAAPL IIAKEKGLFE KYGMTDVSVE KQASWGTTRD NLELGSGGGG IDGAHILTPM PYLMALGTVT
ThelSB4 --EVTGAKLG FIALTDAAPL IIAKEKGLFA KYGMPDVEVL KQASWGTTRD NLVLGSANGG IDGAHILTPM PYLMTVGKIT
ThNK55aSB9 --EVTGAKLG FIALTDAAPL IIAKEKGFFA KYGMPDVEVI KQASWGVTRD NLVLGSGGGG IDGAHILTPM PYLMTAGTIT
LeJ1BP10 --EVTTAKLG FIALTDSAPL IIAKEKGLFA KYGMPDVEVV KQASWAVTRD NLELGSGGGG IDGAHILTPM SYLMAAGKIT
LeO77BP11 --EVTTAKLG FIALTDSAPL IIAKEKGLFA KYGMPDVEVL KQASWAVTRD NLELGSSGGG IDGAHILTPM PYLMSAGAIT
OsheBP9 --ETTKARLG FIALTDSAPL IIAKEKGFFD KYGMKDVEVA KQASWAATRD NIVLGSDGGG IDGAHILTPM PYLISTGAVT
Os12SB6 --EVTNASLG FIALTDSAPL IIALEKGMFA KYGMKDVKLV KQTSWAATRD NLVTGSQGGG IDGAHILSPM PYLMTTGKIT
Mi7113SB3 --EVTTAKLG FIALTDAAPL IIAKEKGLFE KYGMKDVKVE KQASWGTTRD NLELGSGGGG IDGAHILTPM PYLISTGAVT
Cy7425NT1 --EVTKARLG FIALTDAAPL IIAKEKGFFA KYGMPDVEVL KQASWGATRD NLELGSAGGG IDGAHILTPM PYLITAGTVT
PsRCBP14 --EVTKAKLG FIALTDAAPL IIAKEKGLFE KYGMKDVEVL KQASWGTTRD NIALGSDAGG IDGAHILSPM PYLLTEGKIT
PsbiSB10 --EVTKAKLG FIALTDAAPL IIAKEKGLFE KYGMKDVEVL KQASWGTTRD NIALGSDAGG IDGAHILSPM PYLLTEGKIT
Ps6802BP21 --EVTTAKLG FIALTDASPL IIAKEKGFFE KYGMKDVQVV KQASWGTTRD NIVLGSEGGG IDGAHILTPM PYLISEGKVT
Sy7502SB5 --ETTKAKLG FIALTDAAPL IIAKEKGYFA KHGMSDVEVL KQASWGSTRD NLVSGSEGGG IDGAHILSPM PYQISEGTVT
MiaeNB2 --EVTTAKLG FIALTDAAPL IIAKEKGLFA KHGMPDVQVM KQASWAATRD NLELGSAGNG IDGAHILSPM PYLMTLGKIT
MiT14NB4 --EVTTAKLG FIALTDSAPL IIAKEKGLFA KHGMPDVQVM KQASWAATRD NLELGSAGNG IDGAHILSPM PYLMTLGKIT
MiaeNT2 --EVTTAKLG FIALTDAAPL IIAKEKGLFA KHGMPDVQVM KQASWAATRD NLELGSAGNG IDGAHILSPM PYLMTLGKIT
MiaeBP17 --EVTTAKLG FIALTDAAPL IIAKEKGLFA KHGMPDVQVM KQASWAATRD NLELGSAGNG IDGSHILSPM PYLMTLGKIT
Ge3709BP12 --EVTTAKLG FIALTDSAPL IIAKEKGFFD KYGMTGVEIL KQASWPVTRD NIELGSAGGG IDGAHILSPM PYLMTLGTIT
GeheBP19 --EVTTAKLG FIALTDSAPL IIAKEKGLFD KYGMTGVEIL KQASWPVTRD NIELGSAGGG IDGAHILSPM PYLMTLGTIT
PlmiBP31 --EVTTAKLG FIALTDSAPL IIAKEKGLFD KYGMTGVEVV KQASWPVTRD NLELGSDGGG IDGAHILTPM PYLMTMGLIT
HyriBP32 --EVTTAKLG FIALTDSAPL IIAKEKGLFD KYGMTGVEVV KQASWPVTRD NLELGSDGGG IDGAHILTPM PYLMTMGLIT
CySB13 --EVTTAKLG FIALTDSTPL IIAKEKGLFD KYGMTGVEVL KQASWPVTRD NLELGSEGGG IDGAHILTPM PYLMTLGKIT
AlatBP22 -VETTSAKLG FIALTDSAPL IVALEKGIFA KYGMTDVKVE KQASWPVTRD NLVLGSAGGG IDGAHILSPM PYLMALGTIT
Sy7509NT5 -VETTTAKLG FIALTDSAPL IVALEKGLYA KYGMTDVKVE KQASWPVTRD NLVLGSGGGG IDGAHILSPM PYLMALGKIT
Pl001BP23 --EVTAAKLG FIALTDSAPL IIAKEKGLFD KYGMKDVEVV KQASWPVTRD NLEIGSQGGG IDGAHILTPM PYFMSLGQTK
Os1080BP25 --EVTAAKLG FIALTDSAPL IVAKEKGLFD KYGMKDVEVV KQASWPVTRD NLEIGSAGGG IDGAHILSPM PYLMSLGQTK
PhamBP36 --EVTTAKLG FIALTDSAPL IVAKEKGFFD KYGMKDVEVL KQASWPVTRD NLETGSAGGG IDGAHILSPM PYLMTLGQTK
Sy33AbNB1 TPETTSAKLG FIALTDAAPL IIAKEKGFFD KYGMKDVEVV KQASWGTTRD NLVLGSAAGG IDGAHILTPM PYLITTGKVT
Sy23BSB2 TPETTSAKLG FIALTDAAPL IIAKEKGFFD KYGMKDVEVV KQASWGTTRD NLVLGSAAGG IDGAHILTPM PYLITTGKVT
RhpaSB8 TPEVKAAKLG FIALTDASPL FVAKEKGIFA KYGMPDVEVQ KQASWGTTRD NLVLGSEGNG IDGAHILTPM PYLISSGKVT
RhpaTA4 GPEVKGAKLG FIALTDASPL FVAKEKGIFA KYGMPDVEVL KQASWGTTRD NLVLGSEGNG IDGAHILTPM PYLISAGKVT
BrjaNB3 GPEVKGAKLG FIALTDASPL FVAKEKGIFA KYGMPDVEVQ KQASWGTTRD NLVLGSEGNG IDGAHILTPM PYLISSGKVT
BrlaBP40 GPEVKAAKLG FIALTDASPL FVAKEKGIFA KYGMPDVEVQ KQASWGTTRD NLVLGSEGNG IDGAHILTPM PYLISSGKVT
St505BP42 GPEVKGAKLG FIALTDASPL FVAKEKGIFA KYGMPDVEVQ KQASWGTTRD NLVLGSEGNG IDGAHILTPM PYLISSGKVT
AfclHP5 GPEVKGAKLG FIALTDASPL FVAKEKGIFA KYGMPDVEVQ KQASWGTTRD NLVLGSEGNG IDGAHILTPM PYLISSGKVT
AffeHP3 GPETSKARLG FIALTDAAPL FVAKEKGIFA KYGMPDVTVE KQASWGTTRD NLVLGSEGNG IDGAHILTPM PYLISAGKVT
BrEc33BP20 GPEVKGAKLG FIALTDATPL FVAKEKGIFA KYGMPDVEVQ KQASWGTTRD NLVLGSEGNG IDGAHILTPM PYLISAGKVT
BrneBP30 GPEVKGAKLG FIALTDATPL FVAKEKGIFA KYGMPDVEVQ KQASWGTTRD NLVLGSEGNG IDGAHILTPM PYLISAGKVT
BrBP50 GPEVKGAKLG FIALTDASPL FVAKEKGIFA KYGMPDVDVQ KQASWGTTRD NLVLGSEGNG IDGAHILTPM PYLISAGKVT
BrAT1BP18 GPEVKGAKLG FIALSDAGPL FVAKDKGLFA KYGMPDTDVQ KQASWGTTRD NLVLGSEGNG IDGAHILTPM PYLISAGKVT
BrjaBP39 GPEVKGAKLG FIALSDAGPL FVAKDKGLFA KYGMPDTDVQ KQASWGTTRD NLVLGSEGNG IDGAHILTPM PYLISAGKVT
BrcaBP28 GPEVKGAKLG FIALSDAGPL FVAKDKGLFA KYGMPDTDVQ KQASWGTTRD NLVLGSEGNG IDGAHILTPM PYLISAGKVT
BryuBP38 GPEVKGAKLG FIALSDAGPL FVAKDKGLFA KHGVPDTDVQ KQASWGTTRD NLVLGSEGNG IDGAHILTPM PYLISAGKVT
Br1024BP24 GPEVKGAKLG FIALSDAGPL FVAKDKGLFA KHGVPDVDVQ KQASWGTTRD NLVLGSEGNG IDGAHILTPM PYLISAGKVT
BrelBP27 GPEVTKATLG FIALSDAGPL FVAKDKGLFA KHGMPDVEVA KQASWGTTRD NLVLGSEGNG IDGAHILTPM PYLISAGKVT
BrreBP33 GPEVTKATLG FIALSDAGPL FVAKDKGFFA KHGMPDVEVA KQASWGTTRD NLVLGSEGNG IDGAHILTPM PYLISAGKVT
MeplBP26 GPEVKGAKLG FIALTDAAPL FVAKEKGIFA KHGMPDVEVL KQASWGTTRD NLVLGSEGNG IDGAHILTPM PYLISAGRVT
MeaqBP35 GPEVKGAKLG FIALTDAAPL FVAKEKGIFA RHGMPDVEVL KQASWGTTRD NLVLGSEGNG IDGAHILTPM PYLISAGRVT
MeexTA3 GPEVKGAKLG FIALTDAAPL FVAKEKGIFA KYGLPETEVL KQASWGTTRD NLVLGSEGNG IDGAHILTPM PYLISAGRVT
MepoTA5 GPEVKGAKLG FIALTDAAPL FVAKEKGIFA KYGLPETEVL KQASWGTTRD NLVLGSEGNG IDGAHILTPM PYLISAGRVT
Xan126BP54 APETNKATLG FIALTDAAPL FVAKEKGLFD KYGMTDVTVA KQASWGTTRD NLVLGSEGNG IDGAHILTPM PYLISAGKVT
AzhaBP45 TPETTTAKLG FIALTDAAPL IIAKEKGFFA RHGMPDVEVL KQASWGTTRD NIVLGSEGNG IDGAHILTPM PYLISAGVVT
PainBP41 GPETTRAVLG FIALTDSAPL IVAKEKGLFA KYGMPDVEVV KQASWGTTRD NLVLGSQGNG IDGAHILTPM PYLISAGKVT
PaphBP46 GPETTRAVLG FIALTDSAPL IVAKEKGLFA KYGMPDVEVV KQASWGTTRD NLVLGSQGNG IDGAHILTPM PYLISAGKVT
SifrNT7 GPETSKAVLG FIALTDSAPL IIAKEKGFFD KYGMTEVEVV KQASWGTTRD NLVLGSAGAG IDGAHILTPM PYLISTGKVT
En816BP65 GPETTKAVLG FIALTDSAPL IIAKEKGFFD KHGMTEVEVV KQASWGTTRD NLVLGSAGAG IDGAHILTPM PYLISTGKVT
SifrSB14 GPETAKAVLG FIALTDSAPL VIAKEKGFFD KYGMTEVEVV KQASWGTTRD NLVLGSAGAG IDGAHILTPM PYLISTGKVT
EnBP62 GPETTKAVLG FIALTDSAPL IVAKEKGLFD KYGMTEVDVV KQASWGTTRD NLVLGSAGAG IDGAHILTPM PYLISTGKVT
AzdoBP37 APETTKAVLG YIALMDAAPL IIAKEKGLFA KYGVPDVEVV KQASWGATRD NLVLGGAANG IDGAHILTPM PYLISTGKVT
Pssibp60 GPEVKKAVLG YIALMDASPL VIAKEKGFFA KHGMPDVEVS KQASWGATRD NLVLGSESNG IDGAHILTPM PYLISAGKVT
MePW1BP43 GPETTKAILG YIALVDAAPL IIAKEKGLFA KHGMPDVEVV KQASWGATRD NLVLGAAANG IDGAHILTPM PYLITTGKVT
Rh142SB12 TPEVTGVKLG FIALTDAAPL IVAKEKGLFE KHGLPEVDVA KQASWGATRD NLVLGGAANG IDGAHILSPL PYLMHTGKVT
RhalBP51 TPEVTGVKLG FIALTDAAPL IVAKEKGLFE KHGLPEVDVA KQASWGATRD NLVLGGAANG IDGAHILSPL PYLMHTGKVT
RhilgBP55 GPEVTGVKLG FIALTDSAPL IIAKEKGFFD KHGLPETDVA KQASWGATRD NLVLGGAANG IDGAHILSPL PYLMHTGKVT
NegaBP64 GPEVKGIKLG YIALTDAAPL VIAKEKGFFD KHGLPDVDVA KQASWGATRD NLVLGGAANG IDGAHILSPL PYLMHTGKVT
OcP6BSBP57 GPEVSGAKLG FIALTDAAPL IVAKEKGLFE KHGLPDVEVA KQASWGATRD NLVLGGASNG IDGAHILSPL PYLIHTGKVT
AgtuBP52 EPEVKGAKIG FIALTDAAPL IIAAEKGLFA KHGMPDVEVL KQASWGATRD NLVLGGASNG IDGAHILTPM PYLMHTGKVT
Agge7BP53 EPEVKGAKIG FIALTDAAPL IIAAEKGLFA KHGMPDVEVL KQASWGATRD NLVLGGASNG IDGAHILTPM PYLMHTGKVT
AgrhiBP56 EPEVKGAKIG FIALTDAAPL IIAAEKGLFA KHGMPDVEVL KQASWGATRD NLVLGGASNG IDGAHILTPM PYLMHTGKVT
Ms1497BP59 TPEVTGAKLG FIALTDAAPL MIAKEKGLFA KFGMPDVEVL KQASWGATRD NLMLGGAANG IDGAHILTPL PYLMHTGKVT
AuD3BP29 GPETTKAILG FIALTDASPL IVAKEKGLFA KHGMADVEVV KQSSWGTTRD NLVLGSAGSG IDGAHILTPM PYLMSAGKIT
AuurBP34 GPETTKAILG FIALTDASPL IVAKEKGLFA KHGMADVEVV KQSSWGTTRD NLVLGSAGSG IDGAHILTPM PYLMSAGKIT
TlsuBP48 KPETTAAKLG FIALTDAAPL FVADEKGFFA KHGMTNVEVL KQSSWGTTRD NLVLGSRRGG IDGAHILTPM PYLIASGTMT
TlsuBP49 KPETTAAKLG FIALTDAAPL FVADEKGFFA KHGMTNVEVL KQSSWGTTRD NLVLGSRRGG IDGAHILTPM PYLIASGTMT
HaKMNT6 -PETTSAKLG FIALTDAAPL FVAVEKGFFA AHGMTDVEVL KQSSWGTTRD NLVLGSQRNG IDGAHILTPM PYLMSAGAIT
HaloBP63 -LETTSAKLG FIALTDAAPL FVADEKGFFA KHGMTDVEVL KQSSWGTTRD NLVLGSQRNG IDGAHILTPM PYLIASGAMT
PspeBP44 SLETTKAKLG FIALTDAAPL FVADEKGFFA KHGMTDVEVL KQSSWGTTRD NLVLGSARNG IDGAHILTPM PYLITTGAMT
PsfuBP58 GLETTKAKLG FIALTDAAPL FVADELGLFA KHGMTGVEVL KQSSWGTTRD NLVLGAGSGG IDGAHILTPM PYLMAAGKVT
Alpr38BP47 -PEVTGVTLG FIALTDSAPL IVAKVKGLFA KYGMPDVNVV KQASWGTTRD NLELGGGAGG IDGAHILTPM PYLMSTGKVT
No7120A1 TPEVTTATLG FLPVTSCCPL IIAKAKGFFA QHGMPDINVV KQPSWAVMRD KLILGAADEG LDGGHLLFPM VYLMATGEIS
No3756BT1 TPEVATATLG FVPVTSGCPL IIAKAKGFFA KHGLPDIRVV KQPSWAVMRD KLMLGAADEG LDGGHLLFPM VYLMNTGEIT
No7524SB1 TPEVTKTTLG FIPVTSCCPL IIAKAKGFFA KHGMPDVVVA KQPSWAVMRD KLMLGAANEG LDGAHLLFPM TYLMATGEIS

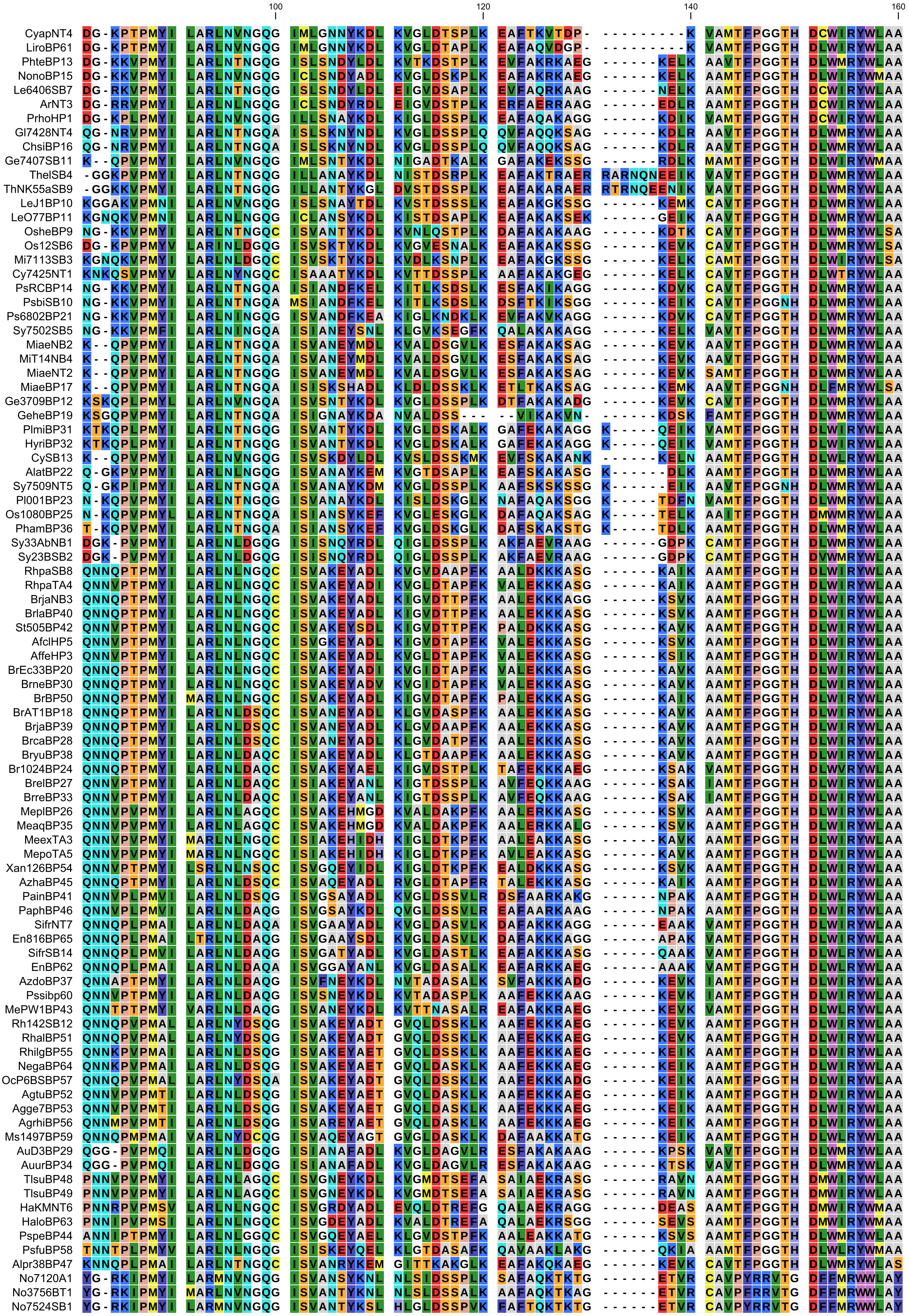
100
120
140
160
CyapNT4
LiroBP61
PhteBP13
NonoBP15
Le6406SB7
ArNT3
PrhoHP1
Gl7428NT4
ChsiBP16
Ge7407SB11
ThelSB4
ThNK55aSB9
LeJ1BP10
LeO77BP11
OsheBP9
Os12SB6
Mi7113SB3
Cy7425NT1
PsRCBP14
PsbiSB10
Ps6802BP21
Sy7502SB5
MiaeNB2
MiT14NB4
MiaeNT2
MiaeBP17
Ge3709BP12
GeheBP19
PlmiBP31
HyriBP32
CySB13
AlatBP22
Sy7509NT5
Pl001BP23
Os1080BP25
PhamBP36
Sy33AbNB1
Sy23BSB2
RhpaSB8
RhpaTA4
BrjaNB3
BrlaBP40
St505BP42
AfclHP5
AffeHP3
BrEc33BP20
BrneBP30
BrBP50
BrAT1BP18
BrjaBP39
BrcaBP28
BryuBP38
Br1024BP24
BrelBP27
BrreBP33
MeplBP26
MeaqBP35
MeexTA3
MepoTA5
Xan126BP54
AzhaBP45
PainBP41
PaphBP46
SifrNT7
En816BP65
SifrSB14
EnBP62
AzdoBP37
Pssibp60
MePW1BP43
Rh142SB12
RhalBP51
RhilgBP55
NegaBP64
OcP6BSBP57
AgtuBP52
Agge7BP53
AgrhiBP56
Ms1497BP59
AuD3BP29
AuurBP34
TlsuBP48
TlsuBP49
HaKMNT6
HaloBP63
PspeBP44
PsfuBP58
Alpr38BP47
No7120A1
No3756BT1
No7524SB1

```
                                        180         200         220         240
                                         |           |           |           |
CyapNT4      GGLEPGKDVS TIVVPPAQMV ANVKVNAMES FCVGEPWPLQ TVNQGVGYQA ITTGQLWKDH PEKAFGMRKD WVDANPKAAK
LiroBP61     GGLEPEKDFS TIVVPPAQMV ANVKVNAMES FCVGEPWPLQ TVNQEVGYQA ITTGELWKDH PEKAFGMRKE WVDKHPKATK
PhteBP13     GGIDPDQDIS TIVVPPPQMV ANIKVGNMDA FCVGEPWPLQ LVNQKIGYNA LTTGELWKDH PEKALGMRAD WVDANPKAAK
NonoBP15     GGIDPDKDVS TIVVPPPQMV ANVKVGNMDS FCVGEPWPLQ TVNQKVGYNA LTTGQLWKDH PEKALGMRAD WVDANPKAAK
Le6406SB7    GGIDPDQDIS TIVVPPPQMV ANIKVGNMDA FCVGEPWPLQ TVNQKIGYNA ITTGEFWKDH PEKALGMRAD WVDANPKATK
ArNT3        GGIDPDQEIS TIVVPPPQMV ANMRVGNMDT FCVGEPWPLQ LVNQDIGYNA LTTGELWQDH PEKALGMRAD WVDEHPKAAR
PrhoHP1      GGIDPDNDIS TIVVPPPQMV ANMKVDAMEA FCVGEPWPLQ TVNQAIGYGA LTTGELWKDH PEKAFGMRAD WVDANPKATQ
Gl7428NT4    GGINPNSDIS LIVVPPPQMV QNVRVGNMET FCVGEPWPAQ TVTQGIGYTA LTTGELWKDH PEKALAMRAD WVDKHPKATK
ChsiBP16     GGINPHSDIS LIVVPPPQMV QNVRVGNMET FCVGEPWPAQ TVAQGIGYTA LTTGELWQDH PEKALAMRAD WVDKHPKATK
Ge7407SB11   GGINPDQDVS TIVVPPPQMV ANIKVSNMEG FCVGEPWPLQ TVNQQIGYMA VTTGEIWKDH PEKAFAMRAE WVDKHPKAAM
ThelSB4      GGIDPEKDVS MIVVPPPQMV ANLKTGTMEA FCVGEPWPLQ TVNQKVGYGA ITTGELWQDH PEKSFALRAD WVDQHPKATK
ThNK55aSB9   GGIDPEKDVS VIVVPPPQMV ANLKTGTMEA FCVGEPWPLQ TVNQKVGWGA VTTGELWKDH PEKSLALRAD WVDKHPKATK
LeJ1BP10     GGIDPDKDVS TIVVPPPQMV ANIKVNNMEA FCVGEPWNAQ TVNQKIGYTA ITTGELWKDH PEKALAMRAD WVEQNPKAAK
LeO77BP11    GGIDPDKDIS TIVVPPPQMV ANIKVNNMEA FCVGEPWPLQ TVNQKLGYNA LTTGELWKDH PEKAFAMRAE WVEQNPKAAK
OsheBP9      GGIDPDKDAS TIVVPPPQMV ANMKVGNMEA FCVGEPWNAQ LVNQGLGYTA FTTGELWKDH PEKAFSMRAD YVDKHPKAAK
Os12SB6      GGITPGKDTS LIVIPPPQMV SNMKEGQMEA FCVGEPWNAQ LVNQGIGYTA ITTGEFWNGH PEKAFTLRAD WVDKNPKAAK
Mi7113SB3    GGIDPEKDVS TIVVPPAQMV ANMKVDNMEA FCVCEPWNKQ LISQGIGYTA LTTGELWNNH PEKAFGMRAD WVDKHPKATM
Cy7425NT1    GGIDPNNDVS LIVVPPPQMV ANMKVGTMET FCVCEPWNKQ AINQGIAFTA LTTGELWNDH PEKALGMRAD WVDKNPKAAK
PsRCBP14     NGVDPDKDVN TIVVPPPQMV ANMKAGNMQA FCVGEPWNAR LVAQNSGYSA LITGELWKDH PEKAFSLRAD WVDKNPKAAK
PsbiSB10     NGIDPDKDVS TIVVPPPQMV ANMKAGNMQA FCVGEPWNAR LVAQNSGYTA FITGEFWKDH PEKAFTMRAD WVDKNPKAAK
Ps6802BP21   NGINPDVDVS TIVVPPPQMV ANMKAGNMQA FCVGEPWNAR LVAQQSGYTA LVTGELWKDH PEKSLALRAD WVDKNPKAAQ
Sy7502SB5    GGINPDKDLS TIVIPPPQMV ANMKSGTMEA FCVGEPWNAR LVAQKHGYTA LVTGELWKNH PEKAFAMRAS WVNKHPKAAQ
MiaeNB2      GGIDPNKDLS LIVVPPAQMV ANMKVGTMQA FCVGEPWNAQ LVNKKLGYSA LITGELWKDH PEKAFALRAD WVDKNPKAAK
MiT14NB4     SGIDPNKDLS LIVVPPAQMV ANMKVGTMQA FCVGEPWNAQ LVNKKLGYSA LITGELWKDH PEKAFALRAD WVDKNPKAAK
MiaeNT2      GGIDPNKDLS LIVVPPAQMV ANMKVGTMQA FCVGEPWNAQ LVKQKLGYSA LITGELWKDH PEKAFALRAD WVDKNPKAAK
MiaeBP17     GGIDPNNDIS LIVVPPPQMV ANMKVGTMDA FCVGEPWNAQ IVSKGLGYTA LITGEFWKDH PEKAFAMRAD WVDKNPKAAK
Ge3709BP12   GGIDPNKDVS VIPVPPPQMV ANMKVGNMEA FCVGEPWNAQ LVNQDIGFSA LVTGELWKDH PEKALSLRKD WVDTNPKAAK
GeheBP19     GGINPDTDVS VIPVPPPQMV ANMKVGNMEA FCVGEPWNAQ LINQDIGYSA LVTGELWNDH PEKALGLRKD WVDANPKAAK
PlmiBP31     GGIDPNKDVS VIPVPPPQMV ANMRTGNMEA FCVGEPWNAQ LVNQQLGYTA IVTGELWKDH PEKAFTMRKD WVDKNPKAAK
HyriBP32     GGIDPNKDVS LIPVPPPQMV ANMRTGNMEA FCVGEPWNAQ LINQQLGYTA IVTGELWKDH PEKAFTMRKD WVDKNPKAAK
CySB13       GGIDPEKDIS VIPVPPPQMV ANMKIGAMET FCVGEPWNAQ LVNQKQGYTA LVTGELWKDH PEKSFALRAD WVDKNPKAAK
AlatBP22     GGVNPDSDIS VVPVPPPQMV ANMKVGNMEA FCVGEPWNGQ LVNQKQGYSA IVTGELWNDH PEKAFAMRAD WVDKNPNAAK
Sy7509NT5    GGIDPNAEIS VVPVPPPQMV ANMKVGNMEA FCVGEPWNAQ LVNQKVGYSA LVTGELWNDH PEKAFTMRAD WVDKNPNSAK
Pl001BP23    GGIVPEQDVS VVPIPPPQMV ANMRTGNMEA FCVGEPWNAQ LVNQGIGYTA LVTGELWQDH PEKALAMRSD WVDKHPKAAK
Os1080BP25   GGIIPDQDIS VVPIPPPQMV ANMKTGNMEA FCVGEPWNAQ LVNQGQGYTA LLTGELWKDH PEKAFAMRAD WVDKHPKAAK
PhamBP36     GGINPDQDIA VIPVPPAQMV ANMRSGSMET FCVGEPWNAQ LINQGLGYTA LVTGELWKDH PEKAFAMRQE WVDKNPKAAK
Sy33AbNB1    GGIDPTSDVS IIVVPPPQMV ANMKSRTMEA FCVGEPWNAQ LVNQKEGYSA LTTGELWNGH PEKAFALRAD WVDAHPKATL
Sy23BSB2     GGIDPTSDVS IIVVPPPQMV ANMKSRTMEA FCVGEPWNAQ LVNQKEGYSA LTTGELWNGH PEKAFALRAD WVDAHPKATL
RhpaSB8      GGIDPDKDIE TIVVPPPQMV ANMKVGTMDC FCVCEPWNLQ LIHQDIGYTA LTTGELWNKH PEKSFAMRAA YVDKYPKAAK
RhpaTA4      GGIDPDKDIE TIVVPPPQMV ANMKVGTMDC FCVCEPWNLQ LIHQNIGYTA ITTGELWNKH PEKSFGMRAA WVDKYPKAAK
BrjaNB3      GGIDPDKDIE TIVVPPAQMV ANMKVGTMDA FCVCEPWNLQ LIHQQIGYTA LTTGELWDKH PEKSFGMRAA YVDKYPKAAK
BrlaBP40     GGIDPDKDIE TIVVPPAQMV ANMKVGTMDA FCVCEPWNLQ LIHQEIGYTA LTTGELWDKH PEKSFGMRAA YVDKYPKAAK
St505BP42    GGIDPDKDIE TIVVPPAQMV ANMKVGTMDA FCVCEPWNLQ LIHQEIGYTA LTTGELWDKH PEKSFGLRAA YVDKYPKAAK
AfclHP5      GGIDPDKDIE TIVVPPAQMV ANMKVGTMDA FCVCEPWNLQ LIHQNIGYTA LTTGELWDKH PEKSFGMRAA YVDKYPKAAK
AffeHP3      GGIDPDKDIE TIVVPPAQMV ANMKVGTMDA FCVCEPWNLQ LIHQNIGYTA LTTGELWDKH PEKSFGMRAA YVDKYPKAAK
BrEc33BP20   GGIDPDKDIE TIVVPPPQMV ANMKVGTMDC FCVCEPWNLQ LIHQEIGYTA ITTGELWDKH PEKSFGMRAA FVDKYPKATK
BrneBP30     GGIDPDKDIE TIVVPPPQMV ANMKVGTMDC FCVCEPWNLQ LIHQEIGYTA ITTGELWDKH PEKSLGMRAA WVDKYPKAAK
BrBP50       GGIDPDKDIE TIVVPPAQMV ANMKVGTMDC FCVCEPWNLQ LIHQDIGYTA ITTGELWDKH PEKSFGLRAA WVDKNPKAAK
BrAT1BP18    GGIDPDKDIE TIVVPPPQMV ANMKVGTMDC FCVGEPWNLQ LIHQKIGYTA VTTGELWNKH PEKSFGMRAA FVDKYPKAAK
BrjaBP39     GGIDPDKDIE TIVVPPPQMV ANMKVGTMDC FCVGEPWNLQ LIHQKIGYTA VNTGELWNKH PEKSFGMRAA FVDKYPKAAK
BrcaBP28     GGIDPDKDIE TIVVPPPQMV ANMKVGTMDC FCVGEPWNLQ LIHQNIGYTA INTGELWNKH PEKSFGMRAA FVDKYPKASK
BryuBP38     GGIDPDKDIE TIVVPPPQMV ANMKVGTMDC FCVGEPWNLQ LIHQGIGYTA VNTGEIWNKH PEKSFGMRAA FVDKYPKASK
Br1024BP24   GGIDPDKDVE TITVPPPQMV ANMKVGTMDA FCVGEPWPGQ LVDQGIGYTA VNTGEIWSKH PEKSLGMRAA FVDKYPKATK
BrelBP27     GGIDPDKDVE TIVVPPPQMV ANMKVGTMDA FCVGEPWNLQ LINQGIGYTA VNTGELWNKH PEKSLGMRAA WVDKYPKAAK
BrreBP33     GGIDPDKDVE TIVVPPPQMV ANMKVGTMDA FCVGEPWNLQ LINQGIGYTA VNTGELWNKH PEKSLGMRAA WVDKYPKAAK
MeplBP26     GGIDPDKDIE TIVVPPPQMV ANMKVGTMDC FCVCEPWNEQ LITQGIGYTA LTTGELWKDH PEKALGMRAS WVDKYPNAAK
MeaqBP35     GGIDPDKDIE TIVVPPPQMV ANMKVGTMDC FCVCEPWNEQ LITQGIGYTA LTTGELWKDH PEKALGMRAS WVDKYPNAAK
MeexTA3      GGIDPDKDIE TIVVPPPQMV ANMKVGTMDC FCVCEPWNEQ LIHQGIGYTA ITTGELWKDH PEKAFAMRAA WVDKYPNAAK
MepoTA5      GGIDPDKDIE TIVVPPPQMV ANMKVGTMDC FCVCEPWNEQ LIHQGIGYTA ITTGELWKDH PEKAFAMRAA WVDKYPNAAK
Xan126BP54   GGIDPDKDIE TIVVPPAQMV ANMKVGTMDC FCVCEPWNEQ LIHQKIGYTA VTTGELWKDH PEKALGMRAA YVDKNPKAAM
AzhaBP45     GGIDPDRDIE TIVVPPAQMV ANMKVGTMDC FCVCEPWNEQ LKNQKIGYTA VNTGELWNRH PEKALGMRAA WVDKHPKAAK
PainBP41     GGIDPDKDVE TIVVPPPQMV ANMKVGTMDC FCVGEPWNEQ LVNQGIGYSA LTTGEIWNNH PEKSLGMRAD WVEKNPRAAQ
PaphBP46     GGIDPDTDVE TIVVPPPQMV ANMKVGTMDC FCVGEPWNEQ LVNQGIGFTA VNTAELWNRH PEKSLGMRAD WVEKNPRAAQ
SifrNT7      AGIDPDKDVE TIVVPPPQMV ANMKVGTMDC FCVGEPWNEQ LVNQKIGYTA VNTAEIWAKH PEKSFAMRAD WVEKNPRAAK
En816BP65    GGIDPDKDVE TIVVPPPQMV ANMKVGTMDC FCVGEPWNEQ LVNQKIGYTA VNTAELWAKH PEKSFAMRAD WVEKNPHAAK
SifrSB14     AGIDPDKDVE TIVVPPPQMV ANMKVGTMDC FCVGEPWNEQ LVNQKIGYSA LTTGEIWAKH PEKSFAMRAD WVEKNPRAAK
EnBP62       GGIDPDSDVE TIVVPPPQMV ANMKVGTMDC FCVGEPWNEQ LVNQKIGYTA VNTAEIWNEH PEKSFAMRAD WVEKNPRAAK
AzdoBP37     AGIDPDKDVS TIVVPPPQMV ANMKVGNMDA FCVGEPWNEQ LVNQGIGFTA CTTGEIWKNH PEKALGMRAD YVDKNPNATK
Pssibp60     GGVDPDKDVS TIVVPPPQMV ANMKVGNMDA FCVGEPWNEQ LVNQNIGYTA CTTGELWFKH PEKALGMRAA WVDKYPNAAK
MePW1BP43    AGIDPDKDVS TIVVPPPQMV ANMKVGNMDA FCVGEPWNEQ LANQGIGYTA CATGEIWSKH PEKALGLRAD WVDKNPRAAR
Rh142SB12    GGIDPDKDVS TIVVPPPQMV ANMKVGNMDV FCVGEPWNEQ LVNQGIGFTA ATTGELWKGH PEKALGMRAD WVEKNPNAAK
RhalBP51     GGIDPDKDVS TIVVPPPQMV ANMKVGNMDV FCVGEPWNEQ LVNQGIGFTA ATTGELWKGH PEKALGMRAD WVEKNPNAAK
RhilgBP55    GGIDPNKDVS TIVVPPPQMV ANMKVGNMDV FCVGEPWNEQ LVNQGIGFTA ATTGELWKGH PEKALGLRAE WIEKNPNAAK
NegaBP64     GGIDPNKDVS TIVVPPPQMV ANMKVGNMDV FCVGEPWNEQ LVNQGIGFTA ATTGELWKGH PEKALGLRAD WIEKNPNAAK
OcP6BSBP57   GGIDPDKDVS TIVVPPPQMV ANMKVGNMDL FCVGEPWNEQ LVNQGIGFTA ATTGEIWKHH PEKALGMRAD WIEKNPNAAK
AgtuBP52     GGIDPDKDVS TIVVPPPQMV ANMKVGNMDV FCVGEPWNEQ LVNQGIGFTA CTTGELWKGH PEKALGMRAD WVEKNPNATK
Agge7BP53    GGIDPDKDVS TIVVPPPQMV ANMKVGNMDV FCVGEPWNEQ LVNQGIGFTA CTTGELWKGH PEKALGMRAD WVEKNPNATK
AgrhiBP56    GGIDPDKDVS TIVVPPPQMV ANMKVGNMDV FCVGEPWNEQ LVNQGIGFTA CTTGELWKGH PEKALGMRAD WVEKNPNATK
Ms1497BP59   GGIDPDKDVS TIVVPPPQMV ANMKVGNMDV FCVGEPWNEQ LVHQGVGFTA ATTGELWKGH PEKALGLRAE FIEKYPKATK
AuD3BP29     AGIDPNTDVE TIVVPPPQMV ANLKVNTMDA FCVGEPWNEQ MVGQKIGFSA LTTGELWNNH PEKSLAMRAD WVAAHPNSAK
AuurBP34     AGIDPNTDVE TIVVPPPQMV ANLKVNTMDA FCVGEPWNEQ MVGQKIGFSA LTTGELWNNH PEKSLAMRAD WVAAHPNSAK
TlsuBP48     GGIDPERNIS TIVVPPPQMV ANMRVGSMDT FCVCEPWNQQ LINQGIGYTA NTTGEIWHNH PEKALGMRAD YVDANPNATR
TlsuBP49     GGIDPERNIS TIVVPPPQMV ANMRVGSMDT FCVCEPWNQQ LINQGIGYTA NTTGEIWHNH PEKALGMRAD YVDANPNATR
HaKMNT6      GGIDPNRDVS TITIPPAQMV ANMRVGSMDT FCVCEPWNHQ LINQGIGYTA NTTGEIWNDH PEKAFALRSD YVEANPNAAM
HaloBP63     GGIDPNRDIS TITIPPAQMV ANMRVGSMDT FCVCEPWNQQ LINQGIGYTA NTTGELWNDH PEKAFAMRSD YVEAHPNATK
PspeBP44     GGIDPNSDIS TIVVPPAQMV ANMKVGSMDT FCVCEPWNEQ LINQKIGYTA LTTSELWHNH PEKALGMRAD WVDANPNAAQ
PsfuBP58     GGIEPNKDLP TVVIPPPQMV ANMKVGSMDA FCVGEPWNAQ LINQGIGYSA VTTGELWANH PEKSLSLRAD YVDANPNATR
Alpr38BP47   NGIDPDKDVS TIVVPPPQMV ANMKVDTMEA FCVGEPWNAQ LVNQGIGFTA LTTGELWHDH PEKSFAMRAD WVEKNPNAAK
No7120A1     GGIDPDRDLS VIVIAPPQMV ASMRSGSMEA FCVVDPWHHR LIKQGLGYST VTTGELWPNH PEKALGVRAE WVDKYPKAAK
No3756BT1    GGIDPDRDLS IIVIAPPQMV ASMRSGSMEA FSVVDPWHHR LIKQGLGYSA VTTGELWNNH PEKAFTMRAE WVDKYPKAAK
No7524SB1    GGIDPDRDTS VIVIAPPQMV ASMRSGTMDA FCVVDPWHYR LIKQKLGYST VTSGELWHNH PEKALAVRAE WVDKYPKAAK
```

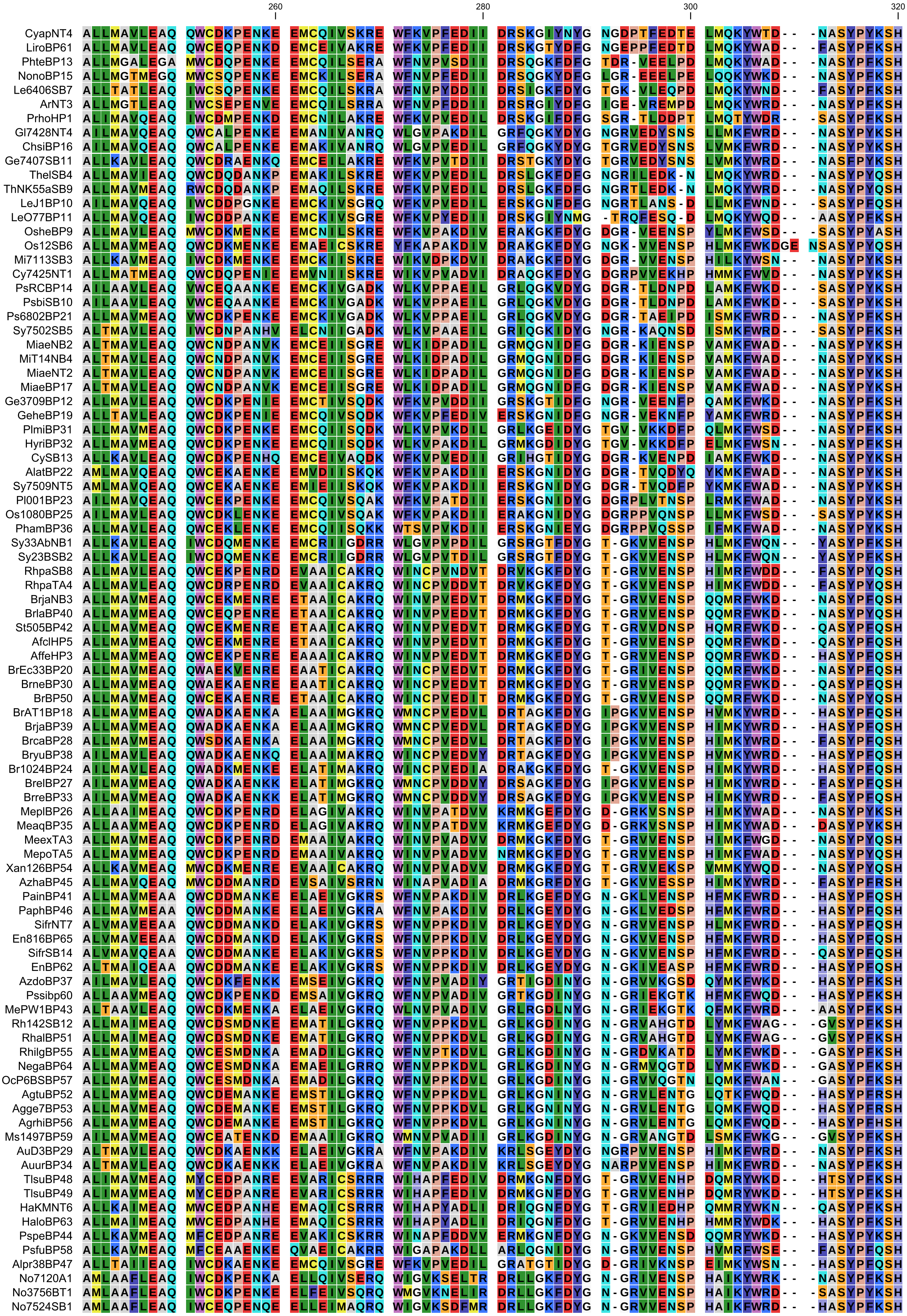
260
280
300
320
CyapNT4 ALLMAVLEAQ QWCDKPENKE EMCQIVSKRE WFKVPFEDII DRSKGIYNYG NGDPTFEDTE LMQKYWTD-- -NASYPYKSH
LiroBP61 ALLMAVLEAQ QWCEQPENKD EMCEIVAKRE WFKVPFEDII DRSKGTYDFG NGEPPFEDTD LMQKYWAD-- -FASYPYKSH
PhteBP13 ALLMGALEGA MWCDQPENKE EMCQILSERA WFNVPVSDII DRSQGKFDFG TDR-VEELPD LMQKYWAD-- -NASYPFKSH
NonoBP15 ALLMGTMEGQ MWCSQPENKE EMCKILSERA WFNVPFEDII DRSQGKYDFG LGR-EEELPE LQQKYWKD-- -NASYPFKSH
Le6406SB7 ALLTATLEAQ IWCSQPENKE EMCQILSKRA WFNVPYDDII DRSIGKFDYG TGK-VLEQPD LMQKYWND-- -FASYPFKSH
ArNT3 ALLMGTLEAQ IWCSEPENVE EMCQILSRRA WFNVPFDDII DRSRGIYDFG IGE-VREMPD LMQKYWRD-- -NASYPFKSH
PrhoHP1 AILMAVQEAQ IWCDMPENKD EMCNILAKRE WFKVPVEDIL DRSKGIFDFG SGR-TLDDPT LMQTYWDR-- -SASYPFKSH
Gl7428NT4 AILMAVQEAQ QWCALPENKE EMANIVANRQ WLGVPAKDIL GRFQGKYDYG NGRVEDYSNS LLMKFWRD-- -NASYPYKSH
ChsiBP16 AILMAVQEAQ QWCALPENKE EMAKIVANRQ WLGVPVEDIL GRFQGKYDYG TGRVEDYSNS LVMKFWRD-- -NASYPYKSH
Ge7407SB11 ALLKAVLEAQ QWCDRAENKQ EMCEILAKRE WFKVPVTDII DRSTGKYDYG TGRVEDYSNS LVMKYWRD-- -NASFPYKSH
ThelSB4 ALLMAVIEAQ QWCDQDANKP EMAKILSKRE WFKVPVEDIL DRSLGKFDFG NGRILEDK-N LMQKYWRD-- -NASYPYQSH
ThNK55aSB9 ALLMAVMEAQ RWCDQDANKP EMAQILSKRE WFKVPVEDIL DRSLGKFDFG NGRTLEDK-N LMQKYWRD-- -NASYPYQSH
LeJ1BP10 AILMAVQEAQ IWCDDPGNKE EMCKIVSGRQ WFKVPVEDIL ERSKGNFDFG NGRTLANS-D LLMKFWND-- -SASYPFQSH
LeO77BP11 ALLMAVQEAQ IWCDDPANKE EMCKIVSGRE WFKVPYEDII DRSKGIYNMG -TRQFESQ-D LMQKYWQD-- -AASYPFKSH
OsheBP9 ALLMAVLEAQ MWCDKMENKE EMCNILSKRE WFKVPAKDIV ERAKGKFDYG DGR-VEENSP YLMKFWSD-- -SASYPYASH
Os12SB6 ALLMAVMEAQ QWCDKMENKE EMAEICSKRE YFKAPAKDIV DRAKGKFDYG NGK-VVENSP HLMKFWKDGE NSASYPYQSH
Mi7113SB3 ALLKAVMEAQ IWCDKMENKE EMCKIISKRE WIKVDPKDVI DRAKGKFDYG DGR-VVENSP HILKYWSN-- -NASYPYKSH
Cy7425NT1 ALLMATMEAQ QWCDQPENIE EMVNIISKRE WIKVPVADVI DRAQGKFDYG DGRPVVEKHP HMMKFWVD-- -NASYPYKSH
PsRCBP14 AILAAVLEAQ VWCEQAANKE EMCKIVGADK WLKVPPAEIL GRLQGKVDYG DGR-TLDNPD LAMKFWKD-- -SASYPYKSH
PsbiSB10 AILAAVLEAQ VWCEQAANKE EMCKIVGADK WLKVPPAEIL GRLQGKVDYG DGR-TLDNPD LAMKFWKD-- -SASYPYKSH
Ps6802BP21 ALLMAVMEAQ VWCDKPENKE EMCKIVGADK WLKVPPAEIL GRLQGKVDYG DGR-TAEIPD ISMKFWRD-- -NASYPFKSH
Sy7502SB5 ALTMAVLEAQ IWCDNPANHV ELCNIIGADK WLKVPAAEIL GRIQGKIDYG NGR-KAQNSD ISMKFWRD-- -SASYPFKSH
MiaeNB2 ALTMAVLEAQ QWCNDPANVK EMCEIISGRE WLKIDPADIL GRMQGNIDFG DGR-KIENSP VAMKFWAD-- -NASYPYKSH
MiT14NB4 ALTMAVLEAQ QWCNDPANVK EMCEIISGRE WLKIDPADIL GRMQGNIDFG DGR-KIENSP VAMKFWAD-- -NASYPYKSH
MiaeNT2 ALTMAVLEAQ QWCNDPANVK EMCEIISGRE WLKIDPADIL GRMQGNIDFG DGR-KIENSP VAMKFWAD-- -NASYPYKSH
MiaeBP17 ALTMAVLEAQ QWCNDPANVK EMCEIISGRE WLKIDPADIL GRMQGNIDFG DGR-KIENSP VAMKFWAD-- -NASYPYKSH
Ge3709BP12 ALLMAVLEAQ QWCDKPENIE EMCTIVSQDK WFKVPVDDII GRSKGTIDFG NGR-VEENFP QAMKFWKD-- -NASYPFKSH
GeheBP19 ALLTAVLEAQ QWCDKPENIE EMCQIVSQDK WFKVPFEDII ERSKGNIDFG NGR-VEKNFP YAMKFWRD-- -NASYPFKSH
PlmiBP31 ALLMAVMEAQ QWCDKPENKE EMCQIISQDK WLKVPVKDIL GRLKGEIDYG TGV-VKKDFP QLMKFWSD-- -NASYPFKSH
HyriBP32 ALLMAVMEAQ QWCDKPENKE EMCQIISQDK WLKVPAKDIL GRMKGDIDYG TGV-VKKDFP ELMKFWSN-- -NASYPFKSH
CySB13 ALLKAVLEAQ QWCDKPENHQ EMCEIVAQDK WFKVPVEDII GRIHGTIDYG DGR-KVENPD IAMKFWKD-- -NASYPYKSH
AlatBP22 AMLMAVQEAQ QWCEKAENKE EMVDIISKQK WFKVPAKDII ERSKGNIDYG DGR-TVQDYQ YKMKFWAD-- -NASYPYKSH
Sy7509NT5 AMLMAVQEAQ QWCEKAENKE EMIEIISKQK WFKVPAKDII ERSKGNIDYG DGR-TVQDFP YKMKFWAD-- -NASYPYKSH
Pl001BP23 AILMAVQEAQ QWCEKPENKE EMCQIVSQAK WFKVPATDII ERSKGNIDYG DGRPLVTNSP LRMKFWAD-- -NASYPYKSH
Os1080BP25 AILMAVLEAQ QWCDKLENKE EMCQIVSQAK WFKVPAKDII ERAKGNIDYG DGRPPVQNSP LLMKFWSD-- -SASYPYKSH
PhamBP36 ALLMAVLEAQ QWCEKLENKE EMCQIISQKK WTSVPVKDII ERSKGNIEYG DGRPPVQSSP IFMKFWAD-- -NASYPYKSH
Sy33AbNB1 ALLKAVLEAQ IWCDQMENKE EMCRIIGDRR WLGVPVPDIL GRSRGTFDYG T-GKVVENSP HLMKFWQN-- -YASYPFKSH
Sy23BSB2 ALLKAVLEAQ IWCDQMENKE EMCRIIGDRR WLGVPVTDIL GRSRGTFDYG T-GKVVENSP HLMKFWQN-- -YASYPFKSH
RhpaSB8 ALLMAVLEAQ QWCEKPENRD EVAAICAKRQ WINCPVNDVT DRVKGKFDYG T-GRVVENSP HIMRFWDD-- -FASYPFQSH
RhpaTA4 ALLMAVLEAQ QWCDRPENRD EVAAICAKRQ WINCPVDDVT DRVKGKFDYG T-GKVVENSP HIMKFWDD-- -FASYPYQSH
BrjaNB3 ALLMAVMEAQ QWCEKMENRE ETAAICAKRQ WINVPVEDVT DRMKGKFDYG T-GRVVENSP QQMRFWKD-- -NASYPFQSH
BrlaBP40 ALLMAVMEAQ QWCEQPENRE ETAAICAKRQ WINVPVEDVT DRMKGKFDYG T-GRVVENSP QQMRFWKD-- -NASYPYQSH
St505BP42 ALLMAVMEAQ QWCEKMENRE ETAAICAKRQ WINVPVEDVT DRMKGKFDYG T-GRVVDNSP HQMRFWKD-- -QASYPFQSH
AfclHP5 ALLMAVMEAQ QWCEKMENRE ETAAICAKRQ WINVPVEDVT DRMKGKFDYG T-GRVVENSP QQMRFWKD-- -QASYPFQSH
AffeHP3 ALLMAVMEAQ QWCEKPENRE EAAAICAKRQ WINVPVEDVT DRMKGKFDYG T-GRVVENSP QQMRFWKD-- -HASYPFQSH
BrEc33BP20 ALLMAVMEAQ QWAEKVENRE EAATICAKRQ WINCPVEDVT DRMKGKFDYG T-GRIVENSP QQMRFWKD-- -NASYPFQSH
BrneBP30 ALLMAVMEAQ QWAEKAENRE EAATICAKRQ WINCPVEDVT DRMKGKFDYG T-GRVVENSP QQMRFWKD-- -QASYPFQSH
BrBP50 ALLMAVMEAQ QWCEKAENRE ETAAICAKRQ WINCPVEDIT DRMKGKFDYG T-GRVVENSP QQMRFWKD-- -NASYPYQSH
BrAT1BP18 ALLMAVMEAQ QWADKAENKA ELAAIMGKRQ WMNCPVEDVL DRTAGKFDYG IPGKVVENSP HVMKYWRD-- -HASYPFQSH
BrjaBP39 ALLMAVMEAQ QWADKAENKA ELAAIMGKRQ WMNCPVEDVL DRTAGKFDYG IPGKVVENSP HIMKYWRD-- -HASYPFQSH
BrcaBP28 ALLMAVMEAQ QWSDKAENKA ELAAIMGKRQ WMNCPVEDVL DRTAGKFDYG IPGKVVENSP HIMKYWRD-- -FASYPFQSH
BryuBP38 AILMAVLEAQ QWADKAENKQ ELAAIMAKRQ WINCPVEDVY DRTAGKFDYG IPGKVVENSP HVMKYWRD-- -HASYPFQSH
Br1024BP24 AILMAVLEAQ QWADKMENKE ELATIMAKRQ WINCPVEDIA DRAKGKFDYG T-GKVVENSP HIMKYWRD-- -HASYPFQSH
BrelBP27 AILMAVMEAQ QWADKAENKK ELATIMGKRQ WMNCPVDDVY DRSAGKFDYG IPGKVVENSP HIMKYWRD-- -FASYPFQSH
BrreBP33 AILMAVMEAQ QWADKAENKK ELATIMGKRQ WMNCPVDDVY DRSAGKFDYG IPGKVVENSP HIMKYWRD-- -FASYPFQSH
MeplBP26 ALLAAIMEAQ QWCDKPENRD ELAGIVAKRQ WINVPATDVV KRMKGEFDYG D-GRKVSNSP HIMKYWAD-- -NASYPYKSH
MeaqBP35 ALLAAVMEAQ QWCDKPENRD ELAGIVAKRQ WINVPATDVV KRMKGEFDYG D-GRKVSNSP HIMKYWAD-- -DASYPYKSH
MeexTA3 ALLMAVMEAQ QWCDKPENRD EMAAIVAKRQ WINVPVADVV DRMKGKFDYG T-GRVVENSP HIMKFWGD-- -NASYPYQSH
MepoTA5 ALLMAVMEAQ QWCDKPENRD EMAAIVAKRQ WINVPVADVV NRMKGKFDYG T-GRVVENSP HIMKFWAD-- -NASYPYQSH
Xan126BP54 ALLKAVMEAQ MWCDKMENRE EVAAICAKRQ WINVPVADVV DRMKGKFDYG T-GRVVEKSP VMMKYWQD-- -NASYPYQSH
AzhaBP45 ALLMAVQEAQ MWCDDMANRD EVSAIVSRRN WINAPVADIA DRMKGKFDYG T-GKVVESSP HIMKFWRD-- -FASYPFRSH
PainBP41 ALLMAVMEAA QWCDDMANKE ELAEIVGKRS WFNVPAKDIV DRLKGEFDYG N-GKLVENSP HFMKFWRD-- -HASYPFQSH
PaphBP46 ALLMAVMEAA QWCDDMANKE ELAEIVGKRA WFNVPAKDIV DRLKGEYDYG N-GKLVEDSP HFMKFWRD-- -HASYPFQSH
SifrNT7 ALVMAVEEAA QWCDDMANKD ELAKIVGKRS WFNVPPKDIV DRLKGEYDYG N-GRVVENSP HLMKFWRD-- -HASYPFQSH
En816BP65 ALVMAVMEAA QWCDDMANKD ELAKIVGKRS WFNVPPKDIV DRLKGEYDYG N-GKVVENSP HFMKFWRD-- -HASYPFQSH
SifrSB14 ALVMAVQEAA QWCDDMANKE ELAKIVGKRS WFNVPPKDIV DRLKGEYDYG N-GKVVENSP HFMKFWRD-- -QASYPFQSH
EnBP62 ALTMAIQEAA QWCDDMANKE ELAKIVGKRS WFNVPPKDIV DRLKGEYDYG N-GKIVEASP HFMKFWRD-- -HASYPFQSH
AzdoBP37 AILMAVLEAQ QWCDKFENKK EMSEIVGKRQ WFNVPVADIY GRTIGDINYG N-GRVVKGSD QYMKFWKD-- -HASYPFKSH
Pssibp60 ALLAAVMEAQ QWCDKPENKD EMSAIVGKRQ WFNVPVADIV GRTKGDLNYG N-GRIEKGTK HFMKFWQD-- -HASYPFKSH
MePW1BP43 ALTAAVLEAQ QWCDKMENKA ELAEIVGKRQ WLNVPVADIV GRLRGDLNYG N-GRIEKGTK QFMKFWRD-- -AASYPFKSH
Rh142SB12 ALLMAIMEAQ QWCDSMDNKE EMATILGKRQ WFNVPPKDVL GRLKGDINYG N-GRVAHGTD LYMKFWAG-- -GVSYPFKSH
RhalBP51 ALLMAIMEAQ QWCDSMDNKE EMATILGKRQ WFNVPPKDVL GRLKGDINYG N-GRVAHGTD LYMKFWAG-- -GVSYPFKSH
RhilgBP55 ALLMAVMEAQ QWCESMDNKA EMADILGKRQ WFNVPTKDVL GRLKGDINYG N-GRDVKATD LYMKFWKD-- -GASYPFKSH
NegaBP64 ALLMAVMEAQ QWCESMDNKA EMAEILGRRQ WFNVPPKDVL GRLKGDINYG N-GRMVQGTD LYMKFWKD-- -GASYPFKSH
OcP6BSBP57 ALLMAVMEAQ QWCESMDNKA EMADILGKRQ WFNVPPKDVL GRLKGDINYG N-GRVVQGTN LQMKFWAN-- -GASYPFKSH
AgtuBP52 ALLMAVMEAQ QWCDEMANKE EMSTILGKRQ WFNVPPKDVL GRLKGNINYG N-GRVLENTG LQTKFWQD-- -HASYPFRSH
Agge7BP53 ALLMAVMEAQ QWCDEMANKE EMSTILGKRQ WFNVPPKDVL GRLKGNINYG N-GRVLENTG LQMKFWQD-- -HASYPFRSH
AgrhiBP56 ALLMAVMEAQ QWCDEMANKE EMSTILGKRQ WFNVPPKDVL GRLKGNINYG N-GRVLENTG LQMKFWQD-- -HASYPFHSH
Ms1497BP59 AILMAVMEAQ QWCEATENKD EMAAILGKRQ WMNVPVADII GRLKGDINYG N-GRVANGTD LSMKFWKG-- -GVSYPFKSH
AuD3BP29 ALTMAVLEAQ QWCDKAENKK ELAEIVGKRA WFNVPAKDIV KRLSGEYDYG NGRPVVENSP HIMKFWRD-- -NASYPFKSH
AuurBP34 ALTMAVLEAQ QWCDKAENKK ELAEIVGKRA WFNVPAKDIV KRLSGEYDYG NARPVVENSP HIMKFWRD-- -NASYPFKSH
TlsuBP48 ALIMAVMEAQ MYCEDPANRE EVARICSRRR WIHAPFEDIV DRMKGNFDYG T-GRVVENHP DQMRYWKD-- -HTSYPFKSH
TlsuBP49 ALIMAVMEAQ MYCEDPANRE EVARICSRRR WIHAPFEDIV DRMKGNFDYG T-GRVVENHP DQMRYWKD-- -HTSYPFKSH
HaKMNT6 ALLKAIMEAQ MWCEDPANHE EMAQICSRRR WIHAPYADLI DRIQGNFDYG T-GRVIEDHP QMMRYWKN-- -QASYPFKSH
HaloBP63 ALLMAIMEAQ MWCEDPANHE EMAQICSRRR WIHAPYADLI DRIQGNFDYG T-GRVVENHP HMMRYWDK-- -HASYPFKSH
PspeBP44 ALLKAVMEAQ MFCEDPANRE EVAKICSKRR WINAPFDDVV ERMKGNFDYG N-GKVVENSD QQMRYWKD-- -HASYPYKSH
PsfuBP58 ALLKAVMEAQ MFCEAAENKE QVAEICAKRR WIGAPAKDLL ARLQGNIDYG N-GRVVENSP HVMRFWSE-- -FASYPFQSH
Alpr38BP47 ALLTAIIEAQ IWCDKAENKE EMCQIVSGRE WFKVPVEDIL GRATGTIDYG D-GRIVKNSP EIMKYWSN-- -HASYPFQSH
No7120A1 AMLAAFLEAQ IWCDKPENKA ELLQIVSERQ WIGVKSELTR DRLLGKFDYG N-GRIVENSP HAIKYWRK-- -NASYPFKSH
No3756BT1 AMLAAFLEAQ IWCDKPENKE ELFEIVSQRQ WMGVKNELIR DRLLGKFDYG N-GRVVENSP HAIKYWRD-- -SASYPYKSH
No7524SB1 AMLAAFLEAQ IWCEQPENQE ELLEIMAQRQ WIGVKSDFMR DRLLGKFDYG N-GRVVENSP HAIKFWRD-- -HASYPFKSH

```
                                      340                            360                            380                            400
                                        |                              |                              |                              |
CyapNT4     DLWFLTENIR WGYIPADTDT KKIVDEVNRE DLWREAATAL GVPESEIPTS PSRGVETFFD GITFDPENPQ AYLDSLKIKT IKA- 387
LiroBP61    DQWFLTENIR WGYLPASTDV NKIVDEVNRE DLWREAAAAI GVPEAEIPTS TSRGVETFFD GVEFDPENPQ AYLDSLTLKA IKA- 387
PhteBP13    DLWFLTENIR WGKLPADTDT KAWVDAVNRE DLWREAATAL GQ-EAMIPKS TSRGVETFFD GVTFDPENPQ AYLDSLKIKR V--- 387
NonoBP15    DTWFLTENIR WGYLPPDTDV KAMVDAVNRE DLWREAATAL GQ-EDMIPKS TSRGVETFFD GVTFDPEDPQ AYLDSLKIKK V--- 387
Le6406SB7   DLWFLTENIR WGYLPPETDI QAMVDAVNRE DLWKEAAIAL GQ-ESAIPDS TSRGVETFFD GITFDPEDPQ DYLNSLAIKR V--- 387
ArNT3       ELWFLTENIR WGYLPPDADI KGMVDRVNRE DLWREAAEAI GQ-GDMIPES TSRGPETFFD GKVFDPEDPQ DYLKSLEITR I--- 387
PrhoHP1     DLWFLTENIR WGYLKPDTDT KALVDQVNRE DLWQAAAKAL GQ-DAAIPAS SSRGIETFFD GVTFDPENPN AYLSSLKIKK V--- 387
Gl7428NT4   DLWFLTENIR WGYIPGDTDT KAVVDQVNRE DLWREAAQAL GVPAAEIPKS PSRGVETFFD GVKFDPENPT AYLNSLKIKK A--- 389
ChsiBP16    DLWFLTENIR WGYIPGDTDT KAVVDQVNRE DLWREAAQAL GVPAAEIPQS TSRGVETFFD GVQFDPENPT AYLNSLKIKK T--- 389
Ge7407SB11  DLWFLTEDIR WGYLPADTDT RKIVDQVNRE DLWREAAKAI GVAEAEIPTT SSRGVETFFD GVKFDPENPE AYLNGLAIKK V--- 388
ThelSB4     DLWFLTEDMR WGYLPPDTDA KALIQKVNRE DIWREAAKTI GVSAAEIPTT TSRGVETFFD GVAFDPQNPQ AYLNSLNLRK V--- 394
ThNK55aSB9  ELWFLTEDIR WGYLPAQTDT KALIQQVNRE DLWREAAKAI GVPAAEIPTT TSRGVETFFD GVAFDPQNPQ AYLSSLSLKK V--- 394
LeJ1BP10    DLWFLMEDMR WGYLPEDTDT KALIQAVNRE DLWREAAKAI G-QEAAIPKS TSRGIETFFD GVKFDPEKPE AYLKSLEIKK I--- 388
LeO77BP11   DLWFLTEDIR WGYLPADTDT KALIDAVNRE DLWREAAKAI G-QEAAIPAS TSRGVETFFD GVKFDPENPS AYLNALKIKK V--- 387
OsheBP9     DLWFLTEDIR WGYLPADTDS KALVKAVNRE DLWKDAAKAI GQEAA-IPKS TSRGIETFFD GVKFDPENPE AYLKSLKIKK VKA- 389
Os12SB6     DLWFITEEKR WGYLPGDLDA KALVKKVNRE DIWREAAKSL SVAAADIPKT TSRGVETFFD GVKFDPEDPE AYLKSLKIKD LKA- 393
Mi7113SB3   DMWFLTEDIR WGYLPADTDI KAVVDQVNRE DLWKEAAKAI GVPAAEIPTS TSRGVETFFD GVKFDPEKPE EYLKSLKIKK VKV- 391
Cy7425NT1   DLWFVTEDIR WGYLPADTDT KQLVDKVNRE DLWREAAKTL GVADADIPKS TSRGVETFFD GVKFDPENPG EYLQTLKIKK V--- 390
PsRCBP14    DIWFVTEDMR WGYLDAETDA KKLVDKVNRE DLWKEAAKAI GKEAD-IPKS TSRGVETFFD GVKFDPENPS EYLKGLKIKK A--- 387
PsbiSB10    DIWFVTEDMR WGYLDAETDA KKLVDKVNRE DLWKEAAKMI GKEAD-IPKS TSRGVETFFD GVKFDPENPS EYLKGLKIKK A--- 387
Ps6802BP21  DLWFVVEDMR WGYFPANTDA KKLVDAVNRE DIWQAAAKAL KLEAD-IPKS TSRGVETFFD GVKFDPEKPD EYLKGLKIKK A--- 387
Sy7502SB5   DIWFVTEDMR WGYFPASTNA KQLVDKVNRE DIWRAAAKAI GQSAA-IPKS TSRGVETFFD GVKFDPANPS AYLKSLKIKK V--- 387
MiaeNB2     DTWFVTEDIR WGYIPADTDI KALVDKVNRE DIWREAATAL NVPADQIPTS TSRGVETFFD GVTFDPENPQ AYLKSLKIKK V--- 387
MiT14NB4    DTWFVTEDIR WGYIPADTDI KALVDKVNRE DIWREAATAL NVPADQIPTS TSRGVETFFD GVKFDPENPT AYLKSLKIKK V--- 387
MiaeNT2     DTWFVTEDIR WGYIPADTDI KALVDKVNRE DIWREAATAL NVPADQIPTS TSRGVETFFD GVTFDPENPT AYLKSLKIKK V--- 387
MiaeBP17    DTWFVTEDIR WGYIPADTDI KALVDKVNRE DIWREAATAL NVPADQIPTS TSRGVETFFD GVKFDPENPQ AYLKSLKIKK V--- 387
Ge3709BP12  DLWFLTEDIR WGYIPADTDT KALVDQVNRE DLWKEAAKAL SVPEAEIPTS TSRGVETFFD GTKFDPENPQ AYLDGLKIKK I--- 389
GeheBP19    DLWFLTEDIR WGYIPADTDT KALVDQVNRE DLWKEAAKAL SVPEAEIPTS TSRGVETFFD GVTFDPENPQ GYLDSLKIKK V--- 383
PlmiBP31    DLWFLTENIR WGYIKPDTDT KKLVDKVNRE DLWKESAKAL KVADAEIPKS TSRGVETFFD GVQFDPEKPE EYLKSLAIKK V--- 390
HyriBP32    DLWFLTENIR WGYIKPDTDT KKLVDEVNRE DLWKEAAKAL KVADAEIPKS TSRGVETFFD GVQFDPEKPE EYLKSLAIKK V--- 390
CySB13      DLWFLTEDMR WGYIPADTDT KTLVDKVNRS DLWKEAAKAI KVADAEIPKS DSRGVETFFD GVKFDPANPK AYLDSLKIKK A--- 387
AlatBP22    DLWFLTENIR WGTLPADTDT KKIIDQVNRE DLWKEAAKAL GVPDAEIPKS TSRGVEKFFD GVTFDPENPQ AYLKSLKIKK A--- 389
Sy7509NT5   DLWFLTENIR WGNLPADTDT KKIIDQVNRE DLWKEAATAL GVPAAEIPTS TSRGVEKFFD GVTFDPENPA AYLKSLKIKK A--- 389
Pl001BP23   DLWFLTENIR WGNIPADTNI KEIVDKVNRE DLWREAAKTL GVAAAEIPTS TSRGIETFFD GVKFDPDKPE EYLKSLAIKK V--- 390
Os1080BP25  DLWFLTENIR WGYLPADTKT KEIVDRVNRE DLWREAAKTL GVPAAQIPAS TSRGVETFFD GIKFDPEKPE EYLKSLAIKK A--- 390
PhamBP36    DLWFLTEDIR WGYIPADTNT KQLIDKVNRE DLWKEAAKSL GVPEAEIPTS TSRGIETFFD GVKFDPENPQ EYLKSLAIKK V--- 390
Sy33AbNB1   DLWFLTENIR WGYLPADTDT KALVERVNRE ELWREAAKAI GQQA-AIPPS TSRGVETFFD GVKFDPENPA AYLEKLAIKK LA-- 390
Sy23BSB2    DLWFLTENIR WGYLPADTDT KALVERVNRE ELWREAAKAI GQEA-AIPPS PSRGVETFFD GVQFDPENPA AYLEKLAIKK LA-- 390
RhpaSB8     DLWFMTEDIR WGKYEPGFDS KALIAKVNRE DLWKDAAKTL GVT--EMPAS TSRGKETFFD GKVFDPEDPQ AYLKSLSIKR VEV- 391
RhpaTA4     DLWFMTEDIR WGKYEPGFDS KALIAKVNRE DLWKDAAKAL GVS--ALPAS TSRGQETFFD GKVFDPADPA AYLKSLSIKR VDA- 391
BrjaNB3     DLWFLTEDIR WGKYEAGFDT KALIAKVNRE DLWKDAAKTL GVAAADIPAS TSRGKETFFD GKVFDPENPA AYLKSLAIKR VEV- 393
BrlaBP40    DLWFITEDIR WGKYEAGYDA KALIAKVNRE DLWKEAAKEL GVAASDIPAS TSRGKEIFFD GKVFDPENPA AYLKSLSIKR VDV- 393
St505BP42   DLWFITEDIR WGKYEANYDA KALIAKVNRE DLWREAAKEL GVAASDIPTS TSRGKETFFD GKVFDPENPS AYLKSLTIKR VEA- 393
AfclHP5     DLWFITEDIR WGKYEAGFDA KSIIAKVNRE DLWKDAAKSL GVAVADIPTS KSRGKETFFD GKVFDPENPA AYLKSLAIKR VEA- 393
AffeHP3     DLWFITEDIR WGKYESGYDA KALIAKVNRE DIWKDAAKDL GIAAADIPSS TSRGKETFFD GKIFDPENPA AYLKSLAIKR VDA- 393
BrEc33BP20  DLWFLTEDIR WGKYEPSFDT KALIAKVNRE DLWKDAAKTL GVASSDIPAS TSRGKETFFD GKVFDPENPA AYLKSLAIKR VEV- 393
BrneBP30    DLWFMTEDIR WGKYEPGFDS KALIAKVNRE DLWKDAAKTL GVASADIPTS TSRGKETFFD GKVFDPENPA AYLKSLAIKR VEV- 393
BrBP50      DLWFLTEDIR WGKYEAGFDT KALIAKVNRE DLWKEAAKEL GVT--DIPAS TSRGKETFFD GKVFDPADPA AYLKSLSIKR VDV- 391
BrAT1BP18   DLWFLTEDIR WGKYEPNFDT KALIAKVNRE DMWRDAAKTL GVAASEIPTS TSRGKETFFD GKVFDPENPA AYLKSLAIKR VEV- 394
BrjaBP39    DLWFLTEDIR WGKYEPNFDT KALIAKVNRE DMWRDAAKTL GVAAADIPTS TSRGKETFFD GKVFDPENPA AYLKSLAIKR VEV- 394
BrcaBP28    DLWFLTEDIR WGKYEANFDT KALIAKVNRE DMWRDAAKTL GVASAEIPAS TSRGKETFFD GKVFDPENPA AYLKSLAIKR VEV- 394
BryuBP38    DLWFLTEDIR WGKYEPNFDT KALIAKVNRE DMWRDAAKTL GVAAADIPAT TSRGKETFFD GKVFDPENPA AYLKSLAIKR VEV- 394
Br1024BP24  DLWFMTEDIR WGKYEPGFDS KALIAKVNRE DLWKDAAKTL GVSASDIPAS TSRGKETFFD GKVFDPENPA AYLKSLAIKR VEV- 393
BrelBP27    DLWFMTEDIR WGKYEAGFDS KALVAKVNRE DLWKEAAKTL GVPAAEIPAS TSRGKETFFD GKVFDPENPA AYLKSLAIKR VEV- 394
BrreBP33    DLWFMTEDIR WGKYEAGFDS KALVAKVNRE DLWKEAAKTL GVPAAEIPTS TSRGKETFFD GKVFDPENPA AYLKSLSIKR VEV- 394
MeplBP26    DLWFLTEDIR WGKFDPQTDT KALIEKVNRE DIWREVAKSM GVTA---PAS TSRGKETFFD GKVFDPADPA AYLNSLGIKK VA-- 389
MeaqBP35    DLWFLTEDIR WGKFDPQTDT KALIEKVNRE DIWREVAKTM GVTA---PSS TSRGKETFFD GKVFDPADPS AYLNSLGIKK VA-- 389
MeexTA3     DLWFLTEDIR WGKFDAQTDT KALIAKVNRE DLWREAAKAL GVAAI--PTS TSRGVETLFD GKVFDPDNPA AYLNSLGIKK VA-- 390
MepoTA5     DLWFLTEDIR WGKFDAATDT KALIGKVNRE DLWREAAKAL GVSAI--PTS TSRGVETLFD GKVFDPENPA AYLNSLGIKK VA-- 390
Xan126BP54  DLWFITEDQR WGKFEPDYDA KALIAKVNRE DMWREAAKAL SVPAAEIPTA TSRGKETFFD GKVFDPENPS AYLSSLGIKR IS-- 392
AzhaBP45    DLWFLTENVR WGKFSADTDF AALIDKVNRE ELWRDAAATL GVPADQIPAS TSRGKETFFD GKVFDPEDPA AYLASLSITR IA-- 392
PainBP41    DAWFLTEDIR WGKFEPETDI KALIAQVNRE DLWREAAKTL GVT--DIPAS TSRGLETFFD GKVFDPENPE AYLKSLAITR IA-- 390
PaphBP46    DAWFLTEDIR WGKFEPETDI KALIAQVNRE DLWREAAKTL GVT--DIPAS TSRGVETFFD GKVFDPENPE AYLKSLAITR IA-- 390
SifrNT7     DAWFLTENIR WGKLAPDTDI KGLIAKVNRE DIWREAAKDL GAA--DIPAS TSRGPETFFD GKVFDPANPE AYLKSLAISR IA-- 390
En816BP65   DAWFLTENIR WGKLAPDTDI KGLIAKVNRE DIWREAAKDL GIA--DIPAS TSRGPETFFD GKVFDAANPE AYLKSLAISR IA-- 390
SifrSB14    DAWFLTENIR WGKLAPDTDI NGLIAKVNRE EIWREAAKDL GVA--DIPAS TSRGPETFFD GKVFDPANPE AYLKSLAISR IA-- 390
EnBP62      DSWFLTENIR WGKFAPDTDV KALVATVNRE DIWREAAKEL GVS--DIPSS TSRGVEKFFD GKVFDPADPQ AYLKSLSISR VA-- 390
AzdoBP37    DAWFVTEDIR WGKFEPSTDI KALVDKVNRE DLWRAAAKEL NVAAADIPAT TSRGVETFFD GKTFDPANPT AYLKSLGIKR VEV- 393
Pssibp60    DAWFVTEDIR WGKFEPTTDI KAMVDKVNRE DLWREAAKQL SVS--DLPAS TSRGKETFFD GKVFDPDKPG EYLKSLSIKR VEV- 391
MePW1BP43   DAWFVTEDIR WGKLEPTTNI KALVDKVNRE DIWREAAKSI NVPAKEIPAS TSRGKETFFD GKVFDPENPQ AYLKSLSIKR AQV- 393
Rh142SB12   DTWFMAENIR WGKLAPNTDI KALVDKVNRE DIWREAAKDL GVGAADIPAS TSRGKETFFD GKVFDPENPS AYLDSLSIKA AS-- 392
RhalBP51    DTWFMAENIR WGKLAPNTDI KALVDKVNRE DIWREAAKDL GVAAADIPAS TSRGKETFFD GKVFDPENPS AYLDSLSIKA AS-- 392
RhilgBP55   DSWFMTENIR WGNLPASTDI KALVNQVNRE DIWREAAKDL GVAAADIPAS SSRGKETFFD GKVFDPENPS AYLDSLSIKA VS-- 392
NegaBP64    DTWFMAENIR WGYLPANTDI KALVNQVNRE DIWRDAAKDL GVAAADIPAS SSRGKETFFD GKVFDPENPS AYLDSLSIKA AS-- 392
OcP6BSBP57  DIWFIAENIR WGKFTPDTDI KALVDQVNRE DIWRDAAKDL GVAAADIPAS TSRGKETFFD GKVFDPENPS AYLDSLAIKV AS-- 392
AgtuBP52    DSWFIAENIR WGKFAPDTDV KALVEKVNRE DIWRAAAKDL GV--ADLPAS TSRGKETFFD GKVFDPENPS AYLESLSIKA AS-- 390
Agge7BP53   DSWFIAENIR WGKFAPDTDV KALVEKVNRE DIWRAAAKDL GV--ADLPAS TSRGKETFFD GKVFDPENPS AYLESLSIKA AS-- 390
AgrhiBP56   DSWFIAENIR WGKFAPDTDV KALVEKVNRE DIWRAAAKDL GV--ADLPAS TSRGKETFFD GKVFDPENPS AYLESLSIKA AS-- 390
Ms1497BP59  DAWFLAENIR WGKFAPTTDI NALVDQVNRE DLWRAAAKDL GVAAADVPAS SSRGVETFFD GKIFDPANPS AYLDSLKIKA SA-- 392
AuD3BP29    DLWFLTEDQR WGYLPADADT KALVEAVNRE DIWRAAAKDL GVAQAEIPAS TSRGVETFFD GKSFDPANPS AYLESLSIKR VA-- 392
AuurBP34    DLWFLTEDQR WGYLPADADT KALVEAVNRE DIWRAAAKDL GVAQAEIPTS TSRGVETFFD GKSFDPANPS AYLESLSIKR VA-- 392
TlsuBP48    DLWFLTENIR WGYLPKDFEM KGIIDRVNRE DLWRDAAATL GVAAEDIPAS TSRGVETFFD GKVFDPENPQ AYLDSLAIKR I--- 391
TlsuBP49    DLWFLTENIR WGYLPKDFEM KGIIDRVNRE DLWRDAAATL GVAAEDIPAS TSRGVETFFD GKVFDPENPQ AYLDSLAIKR I--- 391
HaKMNT6     DLWFLTENKR WGYLPQDFDS QALIEQVNRE DLWREAAAAL GIDSADIPDS TSRGVETFFD GKTFDPEDPQ AYLDSLEIKR LA-- 391
HaloBP63    DLWFLTENKR WGYLPQDFDS AALIEQVNRE DLWREAAHAL GVASADIPDS TSRGVETLFD GKVFDPENPQ AYLDSLEIKA LA-- 391
PspeBP44    DKWFITEDIR WGYLPQDLDM DALVDQVNRE DLWRAAATSL GVPAAQIPTS TSRGIETFFD GKTFDPENPK AYLDSLSLKA MS-- 392
PsfuBP58    DLWFLTENKR WGYLPKDFDS QALVDTVNRE DIWRQAAAEL AVPAEQIPAS KSRGVETFFD GKTFDPQNPQ AYLDSLSLKA ---- 390
Alpr38BP47  DLWFLTEDQR WGYLPGDLDT KALIAKVNRQ DLWRDAAKVA GAT--DLPAG TSRGIEKFFD GRSFDPANPA AYLASHDIKR IA-- 389
No7120A1    DLWFLIEDMR WGYRSPDFDT KPLIDAVNRE DLWREAAKLI GQES-AIPAS TSRGVEKFFN GLEFNPENPQ AYLNTPKIRI M--- 389
No3756BT1   DLWFLTEDMR WGYRSPDFAT KPLIDAVNRE DVWREAAKLI GQES-AIPAS TSRGVEKFFN GLEFDPENPQ AYLNAPKIRI M--- 389
No7524SB1   DLWFLIEDMR WGYRSFDFAT KPLIDAVNRE DLWREAAKLI GQES-AIPVS TSRGVERFFN GLEFDPENPQ AYLNAPKVRM G--- 389
```

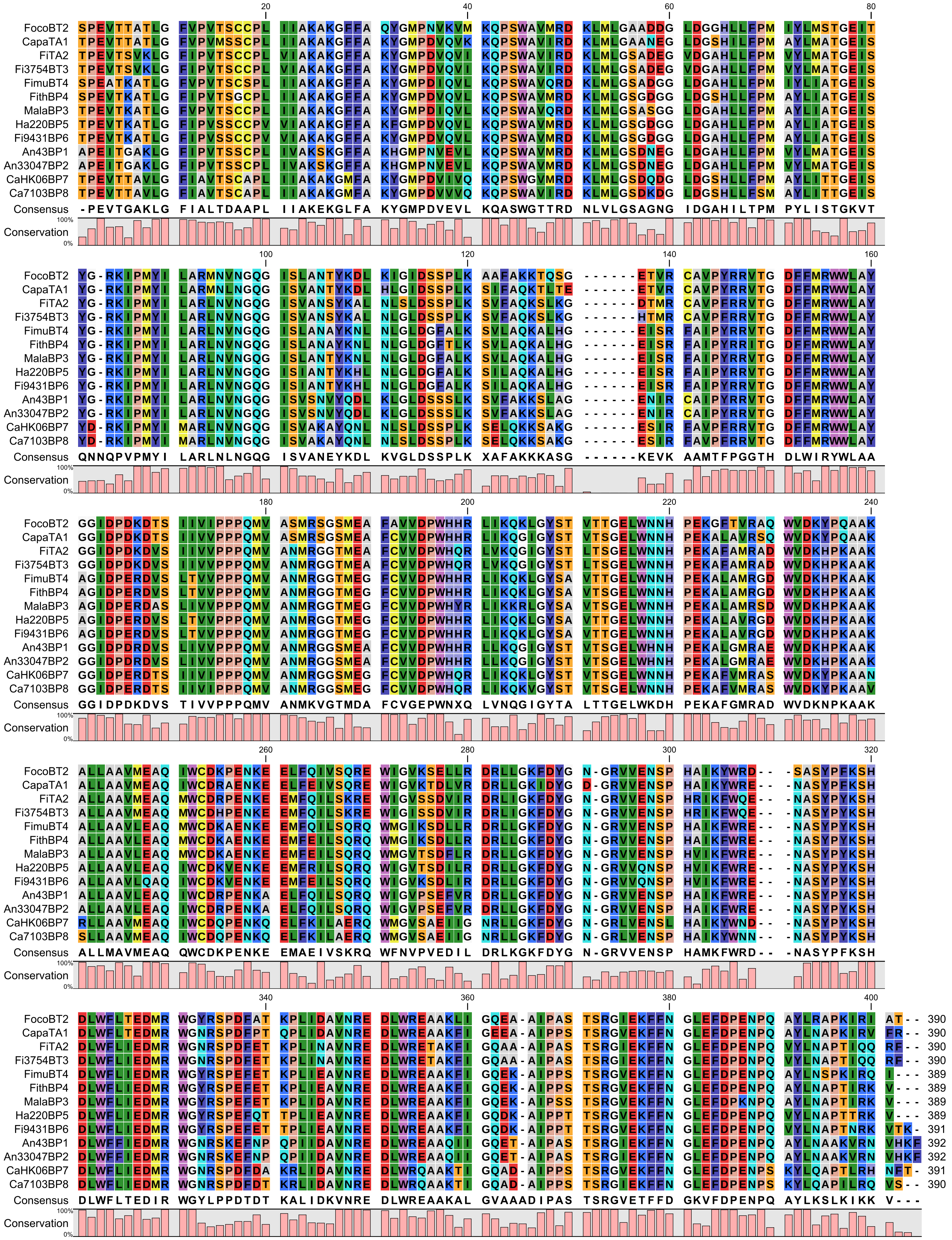
20 40 60 80
FocoBT2 SPEVTTATLG FVPVTSCCPL IIAKAKGFFA QYGMPNVKVM KQPSWAVMRD KLMLGAADDG LDGGHLLFPM IYLMSTGEIT
CapaTA1 TPEVTTATLG FVPVMSSCPL IIAKAKGFFA KYGMPDVQVK KQPSWAVIRD KLMLGAANEG LDGSHILFPM AYLMATGEIS
FiTA2 TPEVTSVKLG FIPVTSCCPL VIAKAKGFFA KYGMPDVQVI KQPSWAVIRD KLMLGSADEG VDGAHLLFPM VYLMATGEIS
Fi3754BT3 TPEVTSVKLG FIPVTSCCPL VIAKAKGFFA KYGMPDVQVI KQPSWAVIRD KLMLGSADEG VDGAHLLFPM VYLMATGEIS
FimuBT4 SPEATKATLG FVPVTSCSPL IIAKAKGFFA KYGMPDIQVL KQPSWAVQRD KLMLGSADGG LDGAHLLFPM IYLIATGEIS
FithBP4 SPEVTKATLG FIPVTSGCPL IIAKAKGFFA KYGMPDVQVL KQPSWAVMRD KLMLGSGDGG LDGSHLLFPM AYLIATGEIS
MalaBP3 TPEVTKATLG FVPVTSCCPL IIAKAKGFFA KYGMPDIQVL KQPSWAVQRD KLMLGSASGG LDGAHLLFPM AYLIATGEIS
Ha220BP5 TPEVTKATLG FIPVSSCCPV IIAKAKGFFA KFGMPDVQVL KQPSWAVMRD KLMLGSGDGG LDGAHLLFPM AYLIATGEIS
Fi9431BP6 TPEVTKATLG FIPVSSCCPL VIAKAKGFFA KYGMPDVQVL KQPSWAVMRD KLMLGSGDGG LDGAHLLFPM AYLIATGEIS
An43BP1 APEITGAKLG FIPVTSSCPL IVAKSKGFFA KHGMPNVEVL KQPSWAVMRD KLMLGSDNEG LDGAHLLFPM VYLMATGEIS
An33047BP2 APEITGAKLG FIPVTSSCPL IVAKSKGFFA KHGMPNVEVL KQPSWAVMRD KLMLGSDNEG LDGAHLLFPM VYLMATGEIS
CaHK06BP7 TPEVTTAVLG FIAVTSCAPL IIAKAKGMFA KYGMPDVIVQ KQPSWGVMRD KLMLGSDQDG LDGSHLLFPM AYLITTGEIS
Ca7103BP8 TPEVTTAVLG FIAVTSCAPL IIAKAKGMFA KYGMPDVVVQ KQPSWAVIRD KLMLGSDKDG LDGSHLLFSM AYLITTGEIS
Consensus -PEVTGAKLG FIALTDAAPL IIAKEKGLFA KYGMPDVEVL KQASWGTTRD NLVLGSAGNG IDGAHILTPM PYLISTGKVT
Conservation 100% 0%
100 120 140 160
FocoBT2 YG-RKIPMYI LARMNVNGQG ISLANTYKDL KIGIDSSPLK AAFAKKTQSG ------ETVR CAVPYRRVTG DFFMRWWLAY
CapaTA1 YG-RKIPMYI LARMNLNGQG ISVANTYKDL HLGIDSSPLK SIFAQKTLTE ------ETVR CAVPYRRVTG DFFMRWWLAY
FiTA2 YG-RKIPMYI LARLNVNGQG ISVANSYKAL NLSLDSSPLK SVFAQKSLKG ------DTMR CAVPFRRVTG DFFMRWWLAY
Fi3754BT3 YG-RKIPMYI LARLNVNGQG ISVANSYKAL NLGLDSSPLK SVFAQKSLKG ------HTMR CAVPFRRVTG DFFMRWWLAY
FimuBT4 YG-RKIPMYI LARLNVNGQG ISLANAYKNL NLGLDGFALK SVLAQKALHG ------EISR FAIPYRRVTG DFFMRWWLAY
FithBP4 YG-RKIPMYI LARLNVNGQG ISLANAYKNL NLGLDGFTLK SVLAQKALHG ------EISR FAIPYRRITG DFFMRWWLAY
MalaBP3 YG-RKIPMYI LARLNVNGQG ISLANTYKNL NLGLDGFALK SVLAQKALHG ------EISR FAIPYRRVTG DFFMRWWLAY
Ha220BP5 YG-RKIPMYI LARLNVNGQG ISIANTYKHL NLGLDGFALK SILAQKALHG ------EISR FAIPYRRVTG DFFMRWWLAY
Fi9431BP6 YG-RKIPMYI LARLNVNGQG ISIANTYKHL NLGLDGFALK SILAQKALHG ------EISR FAIPYRRVTG DFFMRWWLAY
An43BP1 YG-RKIPMYI LARLNVNGQG ISVSNVYQDL KLGLDSSSLK SVFAKKSLAG ------ENIR CAIPYRRVTG DFFMRWWLAY
An33047BP2 YG-RKIPMYI LARLNVNGQG ISVSNVYQDL KLGLDSSSLK SVFAKKSLAG ------ENIR CAIPYRRVTG DFFMRWWLAY
CaHK06BP7 YD-RKIPMYI MARLNVNGQG ISVAKAYQNL NLSLDSSPLK SELQKKSAKG ------ESIR FAVPFRRVTG DFFMRWWLAY
Ca7103BP8 YD-RKIPMYI MARLNVNGQG ISVAKAYQNL NLSLDSSPLK SELQKKSAKG ------ESIR FAVPFRRVTG DFFMRWWLAY
Consensus QNNQPVPMYI LARLNLNGQG ISVANEYKDL KVGLDSSPLK XAFAKKKASG ------KEVK AAMTFPGGTH DLWIRYWLAA
Conservation 100% 0%
180 200 220 240
FocoBT2 GGIDPDKDTS IIVIPPPQMV ASMRSGSMEA FAVVDPWHHR LIKQKLGYST VTTGELWNNH PEKGFTVRAQ WVDKYPQAAK
CapaTA1 GGIDPDKDTS IIVIPPPQMV ASMRSGSMEA FCVVDPWHHR LIKQGIGYST VTSGELWNNH PEKALAVRSQ WVDKYPQAAK
FiTA2 GGIDPDKDVS IIVVPPPQMV ANMRGGTMEA FCVVDPWHQR LVKQGIGYST LTSGELWNNH PEKAFAMRAD WVDKHPKAAK
Fi3754BT3 GGIDPDKDVS IIVVPPPQMV ANMRGGTMEA FCVVDPWHQR LVKQGIGYST LTSGELWNNH PEKAFAMRAD WVDKHPKAAK
FimuBT4 AGIDPERDVS LTVVPPPQMV ANMRGGTMEG FCVVDPWHHR LIKQKLGYSA VTTGELWNNH PEKALAMRGD WVDKHPKAAK
FithBP4 AGIDPERDVS LTVVPPPQMV ANMRGGTMEG FCVVDPWHHR LIKQKLGYSA VTSGELWNNH PEKALAMRGD WVDKHPKAAK
MalaBP3 AGIDPERDAS LIVVPPPQMV ANMRGGTMEG FCVVDPWHYR LIKKRLGYSA VTSGELWNNH PEKALAMRSD WVDKHPKAAK
Ha220BP5 AGIDPERDVS LTVVPPPQMV ANMRGGTMEG FCVVDPWHHR LIKQKLGYSA VTTGELWNNH PEKALAVRGD WVDKHPKAAK
Fi9431BP6 AGIDPERDVS LTVVPPPQMV ANMRGGTMEG FCVVDPWHHR LIKQKLGYSA VTTGELWNNH PEKALAVRGD WVDKHPKAAK
An43BP1 GGIDPDRDVS LIVVPPPQMV ANMRGGSMEA FCVVDPWHHR LLKQGIGYST VTSGELWHNH PEKALGMRAE WVDKHPKAAK
An33047BP2 GGIDPDRDVS LIVVPPPQMV ANMRGGSMEA FCVVDPWHHR LLKQGIGYST VTSGELWHNH PEKALGMRAE WVDKHPKAAK
CaHK06BP7 GGIDPERDTS IIVIPPPQMV ANMRGGSMEG FCVVDPWHQR LIKQKLGYST VTSGELWNNH PEKAFVMRAS WVDKYPKAAN
Ca7103BP8 GGIDPERDTS IIVIPPPQMV ANMRGGSMEG FCVVDPWHQR LIKQKVGYST VTSGELWNNH PEKAFVMRAS WVDKYPKAAV
Consensus GGIDPDKDVS TIVVPPPQMV ANMKVGTMDA FCVGEPWNXQ LVNQGIGYTA LTTGELWKDH PEKAFGMRAD WVDKNPKAAK
Conservation 100% 0%
260 280 300 320
FocoBT2 ALLAAVMEAQ IWCDKPENKE ELFQIVSQRE WIGVKSELLR DRLLGKFDYG N-GRVVENSP HAIKYWRD-- -SASYPFKSH
CapaTA1 ALLAAVMEAQ IWCDRAENKE ELFEIVSQRE WIGVKTDLVR DRLIGKIDYG D-GRVVENSP HAIKYWRE-- -NASYPFKSH
FiTA2 ALLAAVMEAQ MWCDRPENKE EMFQILSKRE WIGVSSDVIR DRLIGKFDYG N-GRVVENSP HRIKFWQE-- -NASYPYKSH
Fi3754BT3 ALLAAVMEAQ MWCDHPENKE EMFQILSKRE WIGISSDVIR DRLIGKFDYG N-GRVVENSP HRIKFWQE-- -NASYPYKSH
FimuBT4 ALLAAVLEAQ MWCDKAENKE EMFQILSQRQ WMGIKSDLLR DRLLGKFDYG N-GRVVENSP HAIKFWRE-- -NASYPYKSH
FithBP4 ALLAAVLEAQ MWCDKAENKE EMFEILSQRQ WMGIKSDLLR DRLLGKFDYG N-GRVVENSP HAIKFWRE-- -NASYPYKSH
MalaBP3 ALLAAVLEAQ MWCDKAENKE EMFEILSQRQ WMGVTSDFLR DRLLGKFDYG N-GRVVENSP HVIKFWRE-- -NASYPYKSH
Ha220BP5 ALLAAVLEAQ IWCDKVENKE EMFRILSQRQ WIGVTSDILR DRLLGKFDYG N-GRVVQNSP HVIKFWRE-- -NASYPYKSH
Fi9431BP6 ALLAAVLQAQ IWCDKVENKE EMFEILSQRQ WIGVKSDLIR DRLLGKFDYG N-GRVVQNSP HVIKFWRE-- -NASYPYKSH
An43BP1 ALLAAVLEAQ IWCDRPENKA ELFQILSQRQ WIGVPSEFVR DRLLGKFDYG N-GRVVENSP HAIKFWRE-- -NASYPYKSH
An33047BP2 ALLAAVLEAQ IWCDRPENKA ELFQILSQRQ WIGVPSEFVR DRLLGKFDYG N-GRVVENSP HAIKFWRE-- -NASYPYKSH
CaHK06BP7 RLLAAVMEAQ IWCDQPENKQ ELFKILAERQ WMGVSAEIIG NRLLGKFDYG N-GRLVENSL HAIKYWND-- -NASYPYKSH
Ca7103BP8 SLLAAVMEAQ IWCDQPENKQ ELFKILAERQ WMGVSAEIIG NRLLGKFDYG N-GRLVENSP HAIKYWNN-- -NASYPYKSH
Consensus ALLMAVMEAQ QWCDKPENKE EMAEIVSKRQ WFNVPVEDIL DRLKGKFDYG N-GRVVENSP HAMKFWRD-- -NASYPFKSH
Conservation 100% 0%
340 360 380 400
FocoBT2 DLWFLTEDMR WGYRSPDFAT KPLIDAVNRE DLWREAAKLI GQEA-AIPAS TSRGIEKFFN GLEFDPENPQ AYLRAPKIRI AT-- 390
CapaTA1 DLWFLTEDMR WGNRSPDFPT QPLIDAVNRE DLWREAAKFI GEEA-AIPPS TSRGVEKFFN GLEFDPENPQ AYLNAPKIRV FR-- 390
FiTA2 DLWFLIEDMR WGNRSPDFET KPLINAVNRE DLWRETAKFI GQAA-AIPAS TSRGIEKFFN GLEFDPENPQ VYLNAPTIQQ RF-- 390
Fi3754BT3 DLWFLIEDMR WGNRSPDFET KPLINAVNRE DLWRETAKFI GQAA-AIPAS TSRGIEKFFN GLEFDPDNPQ VYLNAPTIQQ RF-- 390
FimuBT4 DLWFLIEDMR WGYRSPEFET KPLIEAVNRE DLWREAAKFI GQEK-AIPPS TSRGVEKFFN GLEFDPENPQ AYLNSPKIRQ I--- 389
FithBP4 DLWFLIEDMR WGYRSPEFET KPLIEAVNRE DLWREAAKFI GQEK-AIPPS TSRGVEKFFN GLEFDPENPQ AYLNAPTIRK V--- 389
MalaBP3 DLWFLIEDMR WGYRSPEFET KPLIDAVNRE DLWREAAKFI GQEK-AIPSS TSRGVEKFFN GLEFDPKNPQ AYLNAPTIRK V--- 389
Ha220BP5 DLWFLIEDMR WGYRSPEFQT TPLIEAVNRE DLWREAAKFI GQDK-AIPPT TSRGVEKFFN GLEFDPENPQ VYLNAPTTRK V--- 389
Fi9431BP6 DLWFLIEDMR WGYRSPEFET TPLIEAVNRE DLWREAAKFI GQDK-AIPPT TSRGVEKFFN GLEFDPENPQ VYLNAPTNRK VTK- 391
An43BP1 DLWFFIEDMR WGNRSKEFNP QPIIDAVNRE DLWREAAQII GQET-AIPAS TSRGIEKFFN GLEFDPENPQ AYLNAAKVRN VHKF 392
An33047BP2 DLWFFIEDMR WGNRSKEFNP QPIIDAVNRE DLWREAAQII GQET-AIPAS TSRGIEKFFN GLEFDPENPQ AYLNAAKVRN VHKF 392
CaHK06BP7 DLWFLIEDMR WGNRSPDFDA KRLIDAVNRE DLWRQAAKTI GQAD-AIPPS TSRGVEKFFN GLEFDPENPS KYLQAPTLRH NFT- 391
Ca7103BP8 DLWFLIEDMR WGNRSPDFDT KRLIDAVNRE DLWRQAAKTI GQAD-AIPPS TSRGIEKFFN GLEFDPENPS KYLQAPILRQ VS-- 390
Consensus DLWFLTEDIR WGYLPPDTDT KALIDKVNRE DLWREAAKAL GVAAADIPAS TSRGVETFFD GKVFDPENPQ AYLKSLKIKK V---
Conservation 100% 0%
