# Supplementary material for: Molecular interaction of nitrate transporter proteins with recombinant glycinebetaine results in efficient nitrate uptake in the cyanobacterium Anabaena PCC 7120
Source: PLoS One. 2021 Nov 18;16(11):e0257870. doi: 10.1371/journal.pone.0257870 (PMC8601584; doi:10.1371/journal.pone.0257870)
Supplement: S5 Fig — (PDF) [file pone.0257870.s005.pdf]

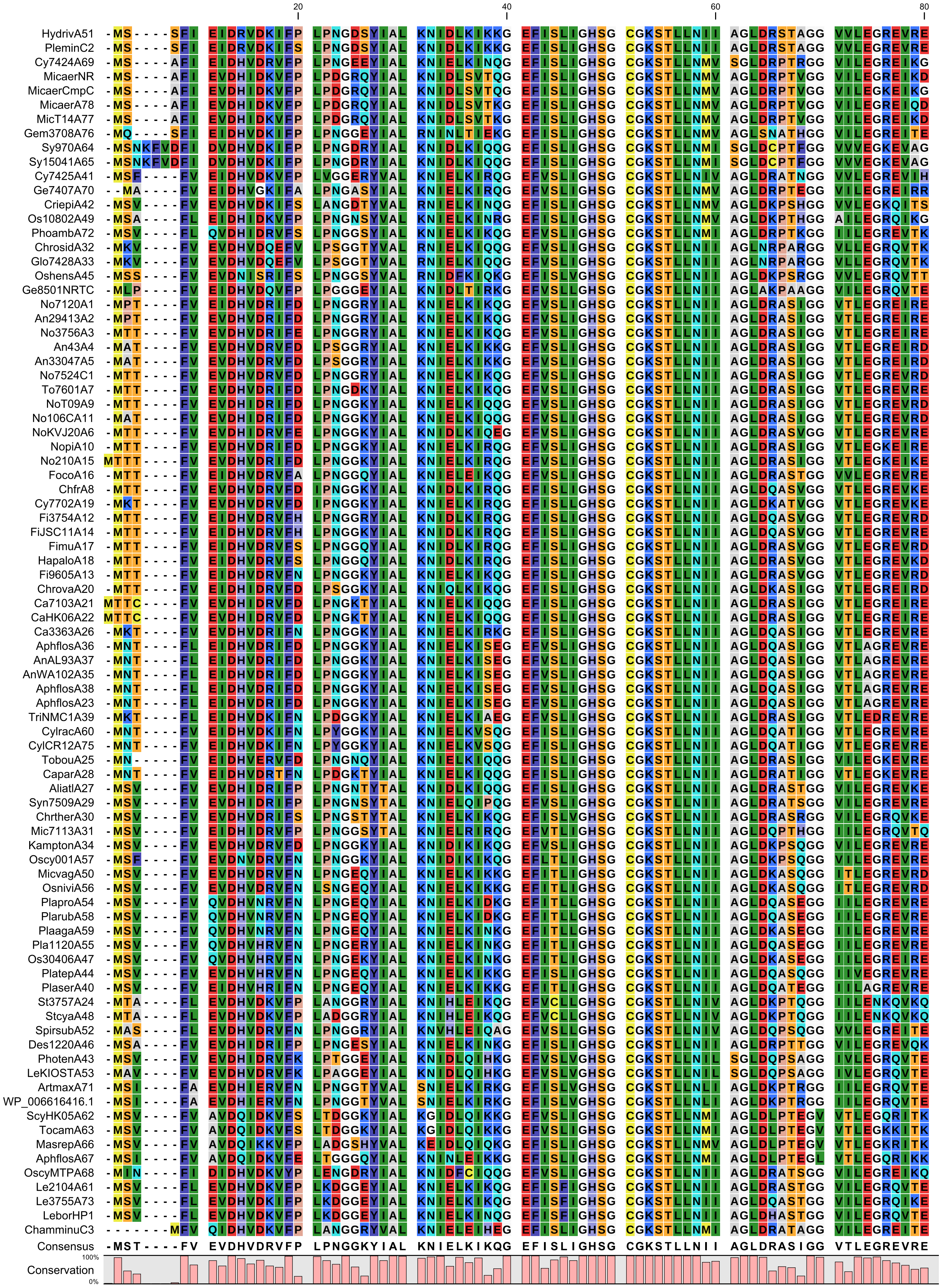
20 40 60 80
HydrivA51 -MS----SFI EIDRVDKIFP LPNGDSYIAL KNIDLKIKKG EFISLIGHSG CGKSTLLNII AGLDRSTAGG VVLEGREVRE
PleminC2 -MS----SFI EIDRVDKIFS LPNGDSYIAL KNIDLKIKKG EFISLIGHSG CGKSTLLNII AGLDRSTAGG VVLEGREVRE
Cy7424A69 -MS----AFI EIDHVDRVFP LPNGEEYIAL KNIELKINQG EFISLIGHSG CGKSTLLNMV SGLDRPTRGG VILEGREIKG
MicaerNR -MS----AFI EVDHIDKVFP LPDGRQYIAL KNIDLSVTQG EFISLIGHSG CGKSTLLNMV AGLDRPTVGG VILEGREIKD
MicaerCmpC -MS----AFI EVDHIDKVFP LPDGRQYIAL KNIDLSVTQG EFISLIGHSG CGKSTLLNMV AGLDRPTVGG VILEGKEIKG
MicaerA78 -MS----AFI EVDHIDKVFP LPDGRQYIAL KNIDLSVTKG EFISLIGHSG CGKSTLLNMV AGLDRPTVGG VILEGREIQD
MicT14A77 -MS----AFI EVDHIDKVFP LPDGRQYIAL KNIDLSVTKG EFISLIGHSG CGKSTLLNMV AGLDRPTVGG VILEGREIKD
Gem3708A76 -MQ----SFI EIDHVDKIFP LPNGGEYIAL RNINLTIEKG EFISLIGHSG CGKSTLLNMV AGLSNATHGG VILEGREITE
Sy970A64 -MSNKFVDFI DVDHVDKIFP LPNGDRYIAL KNIDLKIQQG EFISLIGHSG CGKSTLLNMI SGLDCPTFGG VVVEGKEVAG
Sy15041A65 -MSNKFVDFI DVDHVDKIFP LPNGDRYIAL KNIDLKIQQG EFISLIGHSG CGKSTLLNMI SGLDCPTFGG VVVEGKEVAG
Cy7425A41 -MSF----FV EIDHVDKVFP LVGGERYVAL KNIELKIRQG EFVSLIGHSG CGKSTLLNIV AGLDRATNGG VVLEGREVIH
Ge7407A70 --MA----FV EIDHVGKIFA LPNGASYIAL KNIELKIRQG EFVSLIGHSG CGKSTLLNMV AGLDRPTEGG VILEGREIRR
CriepiA42 -MSV----FV EVDHVDKIFS LANGDTYVAL RNIELKINEG EFISLIGHSG CGKSTLLNMV AGLDKPSHGG VVLEGKQITS
Os10802A49 -MSA----FL EIDHIDKVFP LPNGNSYVAL KNIELKINRG EFISLIGHSG CGKSTLLNMV AGLDKPTHGG AILEGRQIKG
PhoambA72 -MSV----FL QVDHIDRVFS LPNGGSYIAL KNIELKIKQG EFVSLIGHSG CGKSTLLNMI AGLDRPTKGG IILEGREVTK
ChrosidA32 -MKV----FV EVDHVDQEFV LPSGGTYVAL RNIELKIQQG EFVSLIGHSG CGKSTLLNII AGLNRPARGG VLLEGRQVTK
Glo7428A33 -MKV----FV EVDHVDQEFV LPSGGTYVAL RNIELKIQQG EFVSLIGHSG CGKSTLLNII AGLNRPARGG VLLEGRQVTK
OshensA45 -MSS----FV EVDNISRIFS LPNGGSYVAL RNIDFKIQKG EFISLVGHSG CGKSTLLNII AGLDKPSRGG VVLEGRQVTT
Ge8501NRTC -MLP----FV EIDHVDQVFP LPGGGEYIAL KNIDLTIRKG EFVSLLGHSG CGKSTLLNII AGLAKPAAGG VILEGRQVTE
No7120A1 -MPT----FV EIDHVDRIFD LPNGGRYIAL KNIELKIKQG EFVSLIGHSG CGKSTLLNII AGLDRASIGG VTLEGREIRE
An29413A2 -MPT----FV EVDHVDRIFD LPNGGRYIAL KNIELKIKQG EFVSLIGHSG CGKSTLLNII AGLDRASIGG VTLEGREIRE
No3756A3 -MTT----FV EVDHVDRIFE LPNGGRYIAL KNIELKIKQG EFVSLIGHSG CGKSTLLNII AGLDRASIGG VTLEGREIRE
An43A4 -MAT----FV EVDHVDRVFD LPSGGRYIAL KNIELKIKKG EFVSLIGHSG CGKSTLLNII AGLDRASIGG VTLEGREIRD
An33047A5 -MAT----FV EVDHVDRVFD LPSGGRYIAL KNIELKIKKG EFVSLIGHSG CGKSTLLNII AGLDRASIGG VTLEGREIRD
No7524C1 -MTT----FV EVDHVDRVFD LPNGGRYIAL KNIELKIKQG EFVSLIGHSG CGKSTLLNII AGLDRASIGG VTLEGREVRD
To7601A7 -MTT----FV EVDHVDRIFD LPNGDKYIAL KNIELKIKQG EFVSLIGHSG CGKSTLLNII AGLDRASIGG VTLEGREVRE
NoT09A9 -MTT----FV EVDHIDRIFD LPNGGKYIAL KNIELKIQQG EFVSLIGHSG CGKSTLLNII AGLDRASIGG VTLEGREVRD
No106CA11 -MAT----FV EVDHIDRIFD LPNGGKYIAL KNIELKIKQG EFVSLIGHSG CGKSTLLNII AGLDRASIGG VTLEGREVRD
NoKVJ20A6 -MTT----FV EVDHIDRVFE LPNGGKYIAL KNIDLKIQEG EFVSLIGHSG CGKSTLLNII AGLDRASVGG VTLEGREVRE
NopiA10 -MTT----FV EVDHVDRIFD LPNGGKYIAL KNIELKIRQG EFVSLIGHSG CGKSTLLNII AGLDRASIGG VTLEGKEIRE
No210A15 MTTT----FV EVDHVDRIFD LPNGGKYIAL KNIELKIRQG EFVSLIGHSG CGKSTLLNII AGLDRASIGG VTLEGKEIKE
FocoA16 -MTT----FV EIDHVDRVFA LPNGGQYIAL KNIELEIKQG EFVSLIGHSG CGKSTLLNII AGLDRASTGG VVLEGREVRE
ChfrA8 -MTT----FV EVDHVDRVFD IPNGGKYIAL KNIDLKIRQG EFISLIGHSG CGKSTLLNII AGLDQASVGG VTLEGREVKE
Cy7702A19 -MKT----FV EVDHVDRVFD IPNGGKYIAL KNIELKIRQG EFISLIGHSG CGKSTLLNII AGLDKATVGG VTLEGREVKE
Fi3754A12 -MTT----FV EIDHVDRVFH LPNGGRYIAL KNIDLKIRQG EFISLIGHSG CGKSTLLNII AGLDQASVGG VTLEGREVRD
FiJSC11A14 -MTT----FV EIDHVDRVFH LPNGGKYIAL KNIDLKIRQG EFISLIGHSG CGKSTLLNII AGLDQASVGG VTLEGREVRE
FimuA17 -MTT----FV EIDHVDRVFS LPNGGQYIAL KNIDLKIRQG EFISLIGHSG CGKSTLLNII AGLDRASVGG VTLEGREVRD
HapaloA18 -MTT----FV EIDHVDRVFS LPNGGQYIAL KNIDLKIRQG EFISLIGHSG CGKSTLLNII AGLDRASVGG VTLEGREVKD
Fi9605A13 -MTT----FV EIDHVDRVFN LPNGGKYIAL KNIELKIRQG EFISLIGHSG CGKSTLLNII AGLDRASVGG VTLEGREVRD
ChrovaA20 -MTT----FV EIDHIDRVFD LPSGGKYIAL KNIQLKIKQG EFISLIGHSG CGKSTLLNII AGLDKASIGG VTLEGREIRD
Ca7103A21 MTTC----FV EVDHIDRVFD LPNGKTYIAL KNIELKIQQG EFISLIGHSG CGKSTLLNII AGLDRASIGG VTLEGREIRE
CaHK06A22 MTTC----FV EVDHIDRVFD LPNGKTYIAL KNIELKIQQG EFISLIGHSG CGKSTLLNII AGLDRASIGG VTLEGREIRE
Ca3363A26 -MKT----FV EVDHVDRIFN LPNGGKYIAL KNIELKIRKG EFISLIGHSG CGKSTLLNII AGLDQASIGG VTLEGREVRE
AphflosA36 -MNT----FL EIDHVDRIFD LPNGGKYIAL KNIELKISEG EFVSLIGHSG CGKSTLLNII AGLDQASIGG VTLAGREVRE
AnAL93A37 -MNT----FL EIDHVDRIFD LPNGGKYIAL KNIELKISEG EFVSLIGHSG CGKSTLLNII AGLDQASIGG VTLAGREVRE
AnWA102A35 -MNT----FL EIDHVDRIFD LPNGGKYIAL KNIELKISEG EFVSLIGHSG CGKSTLLNII AGLDQASIGG VTLAGREVRE
AphflosA38 -MNT----FL EIDHVDRIFD LPNGGKYIAL KNIELKISEG EFVSLIGHSG CGKSTLLNII AGLDQASIGG VTLAGREVRE
AphflosA23 -MNT----FL EIDHVDRIFD LPNGGKYIAL KNIELKISEG EFVSLIGHSG CGKSTLLNII AGLDQASIGG VTLAGREVRE
TriNMC1A39 -MKT----FL EIDHVDKIFN LPDGGKYIAL KNIELKIAEG EFVSLIGHSG CGKSTLLNII AGLDRASIGG VTLEDREVRE
CylracA60 -MNT----FV EIDHVDKIFN LPYGGKYIAL KNIELKVSQG EFISLIGHSG CGKSTLLNII AGLDQATIGG VTLEGREVRE
CylCR12A75 -MNT----FV EIDHVDKIFN LPYGGKYIAL KNIELKVSQG EFISLIGHSG CGKSTLLNII AGLDQATIGG VTLEGREVRE
TobouA25 -MN-----FV EIDHVERVFD LPNGNQYIAL KNIELKIQQG EFISLIGHSG CGKSTLLNII AGLDRASIGG VILEGKEVRE
CaparA28 -MNT----FV EIDHVDRTFN LPDGKTYIAL KNIELKIQQG EFISLIGHSG CGKSTLLNII AGLDRATIGG VTLEGKEVRE
AliatlA27 -MSV----FV EVDHIDRIFP LPNGNTYTAL KNIDLKIQQG EFISLIGHSG CGKSTLLNII AGLDRASTGG VILEGKEVRE
Syn7509A29 -MSV----FV EVDHIDRIFP LPNGNSYTAL KNIELQIPQG EFVSLIGHSG CGKSTLLNII AGLDRATSGG VILEGREVRE
ChrtherA30 -MSV----FV EVDHVDRIFS LPNGSTYTAL KNIELKIKQG EFISLVGHSG CGKSTLLNII AGLDRASRGG VILEGRQVKE
Mic7113A31 -MSV----FV EIDHIDRVFP LPNGGSYTAL KNIELRIRQG EFVTLIGHSG CGKSTLLNII AGLDQPTHGG IILEGRQVTQ
KamptonA34 -MSV----FV EVDHVDRVFD LPNGGKYIAL KNIDIKIKQG EFVSLIGHSG CGKSTLLNII AGLDKPSQGG VILEGREVRE
Oscy001A57 -MSF----FV EVDNVDRVFN LPNGGKYIAL KNIDIKIKQG EFLTLIGHSG CGKSTLLNII AGLDKPSQGG VILEGREVRE
MicvagA50 -MSV----FV EVDHVDRVFN LPNGEQYIAL KNIELKIKKG EFITLIGHSG CGKSTLLNII AGLDKASQGG ITLEGREVRD
OsniviA56 -MSV----FV EVDHVDRVFN LSNGEQYIAL KNIELKIKKG EFITLIGHSG CGKSTLLNII AGLDKASQGG ITLEGREVRD
PlaproA54 -MSV----FV QVDHVNRVFN LPNGEQYIAL KNIELKIDKG EFITLLGHSG CGKSTLLNII AGLDQASEGG IILEGREVRE
PlarubA58 -MSV----FV QVDHVNRVFN LPNGEQYIAL KNIELKIDKG EFITLLGHSG CGKSTLLNII AGLDQASEGG IILEGREVRE
PlaagaA59 -MSV----FV QVDHVNRVFN LPNGEQYIAL KNIELKINKG EFITLIGHSG CGKSTLLNII AGLDKASEGG IILEGREVRE
Pla1120A55 -MSV----FV QVDHVHRVFN LPNGERYIAL KNIELKINKG EFITLIGHSG CGKSTLLNII AGLDQASEGG IILEGREVRE
Os30406A47 -MSV----FV QVDHVHRVFN LPNGEKYIAL KNIELKINKG EFITLIGHSG CGKSTLLNII AGLDKASEGG IILEGREIRE
PlatepA44 -MSV----FV EVDHVHRVFN LPNGEQYIAL KNIELKIKKG EFISLIGHSG CGKSTLLNII AGLDQASQGG IIVEGREVRE
PlaserA40 -MSV----FV EIDHVHRIFN LPNGEQYIAL KNIELKINKG EFITLIGHSG CGKSTLLNII AGLDQATEGG IILAGREVRE
St3757A24 -MTA----FL EVDHVDKVFP LANGGRYIAL KNIHLEIKQG EFVCLLGHSG CGKSTLLNIV AGLDKPTQGG IILENKQVKQ
StcyaA48 -MTA----FL EVDHVDKVFP LADGGRYIAL KNIHLEIKQG EFVCLLGHSG CGKSTLLNIV AGLDKPTQGG IILENKQVKQ
SpirsubA52 -MAS----FL EVDHVDRVFN LPNGGRYIAI KNVHLEIQAG EFVSLIGHSG CGKSTLLNIV AGLDQPSQGG VVLEGREITE
Des1220A46 -MSA----FV EVDHIDRIFP LPNGESYIAL KNIELKINKG EFISLIGHSG CGKSTLLNII AGLDRPTTGG VILEGREVQK
PhotenA43 -MSV----FV EVDHIDRVFK LPTGGEYIAL KNIDLQIHKG EFVSLVGHSG CGKSTLLNIL SGLDQPSAGG IVLEGRQVTE
LeKIOSTA53 -MAV----FV EVDHVDRVFK LPAGGEYIAL KNIDLQIHKG EFVSLVGHSG CGKSTLLNIL SGLDQPSAGG IVLEGRQVTE
ArtmaxA71 -MSI----FA EVDHIERVFN LPNGGTYVAL SNIELKIRKG EFISLVGHSG CGKSTLLNLI AGLDKPTRGG IILEGRQVTE
WP_006616416.1 -MSI----FA EVDHIERVFN LPNGGTYVAL SNIELKIRKG EFISLVGHSG CGKSTLLNLI AGLDKPTRGG IILEGRQVTE
ScyHK05A62 -MSV----FV AVDQIDKVFS LTDGGKYIAL KGIDLQIKKG EFVSLIGHSG CGKSTLLNMI AGLDLPTEGV VTLEGQRITK
TocamA63 -MSV----FV AVDQIDKVFS LTDGGKYIAL KGIDLQIKKG EFVSLIGHSG CGKSTLLNMI AGLDLPTEGV VTLEGKKITK
MasrepA66 -MSV----FV AVDQIKKVFP LADGSHYVAL KEIDLQIQKG EFISLIGHSG CGKSTLLNMV AGLDLPTEGV VTLEGKRITK
AphflosA67 -MSI----FV AVDQIDKVFE LTGGGQYIAL KNINLEIKKG EFISLIGHSG CGKSTLLNMI AGLDLPTEGL VTLEGQRIKK
OscyMTPA68 -MIN----FI DIDHVDKVYP LENGDRYIAL KNIDFCIQQG EFVSLIGHSG CGKSTLLNII AGLDRATSGG VILEGREIKQ
Le2104A61 -MSV----FL EVDHVDKVFP LKDGGEYIAL KNIELKIKQG EFISFIGHSG CGKSTLLNII AGLDRASTGG VILEGRQVTE
Le3755A73 -MSV----FL EVDHVDKIFP LKDGGEYIAL KNIELKIKQG EFISFIGHSG CGKSTLLNII AGLDQASTGG VILEGRQIKE
LeborHP1 -MSV----FL EVDHVDKVFP LKDGGEYIAL KNIELKINQG EFISFIGHSG CGKSTLLNII AGLDHASTGG VILEGRQVTE
ChamminuC3 -------MFV QIDHVDKVFP LANGGRYVAL KNIELEIHEG EFISLIGHSG CGKSTLLNMI AGLDRATAGG VILEGREITE
Consensus -MST----FV EVDHVDRVFP LPNGGKYIAL KNIELKIKQG EFISLIGHSG CGKSTLLNII AGLDRASIGG VTLEGREVRE
100%
Conservation
0%

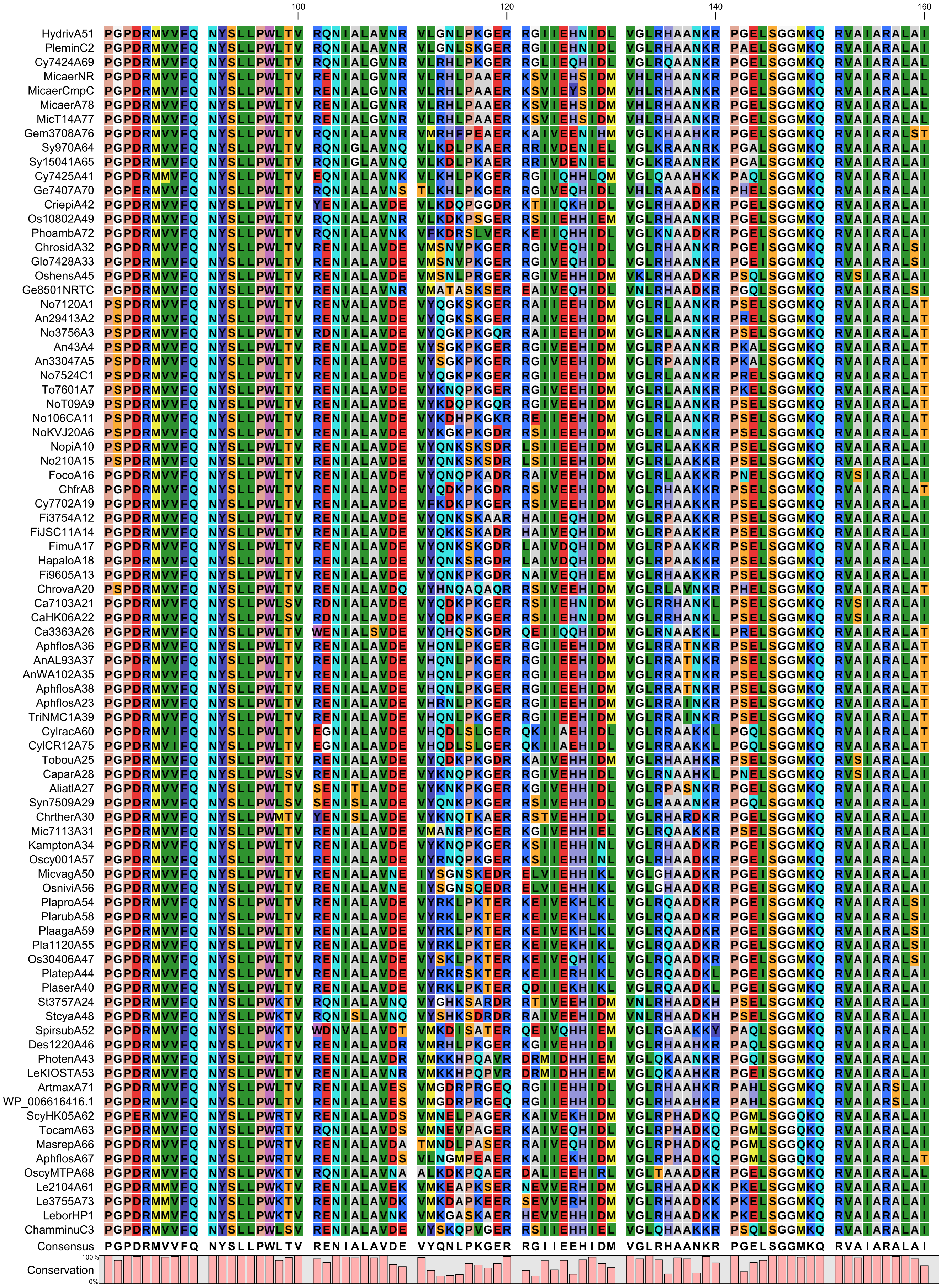
100
120
140
160
HydrivA51
PleminC2
Cy7424A69
MicaerNR
MicaerCmpC
MicaerA78
MicT14A77
Gem3708A76
Sy970A64
Sy15041A65
Cy7425A41
Ge7407A70
CriepiA42
Os10802A49
PhoambA72
ChrosidA32
Glo7428A33
OshensA45
Ge8501NRTC
No7120A1
An29413A2
No3756A3
An43A4
An33047A5
No7524C1
To7601A7
NoT09A9
No106CA11
NoKVJ20A6
NopiA10
No210A15
FocoA16
ChfrA8
Cy7702A19
Fi3754A12
FiJSC11A14
FimuA17
HapaloA18
Fi9605A13
ChrovaA20
Ca7103A21
CaHK06A22
Ca3363A26
AphflosA36
AnAL93A37
AnWA102A35
AphflosA38
AphflosA23
TriNMC1A39
CylracA60
CylCR12A75
TobouA25
CaparA28
AliatlA27
Syn7509A29
ChrtherA30
Mic7113A31
KamptonA34
Oscy001A57
MicvagA50
OsniviA56
PlaproA54
PlarubA58
PlaagaA59
Pla1120A55
Os30406A47
PlatepA44
PlaserA40
St3757A24
StcyaA48
SpirsubA52
Des1220A46
PhotenA43
LeKIOSTA53
ArtmaxA71
WP_006616416.1
ScyHK05A62
TocamA63
MasrepA66
AphflosA67
OscyMTPA68
Le2104A61
Le3755A73
LeborHP1
ChamminuC3
Consensus
PGPDRMVVFQ NYSLLPWLTV RENIALAVDE VYQNLPKGER RGIIEEHIDM VGLRHAANKR PGELSGGMKQ RVAIARALAI
Conservation
100%
0%

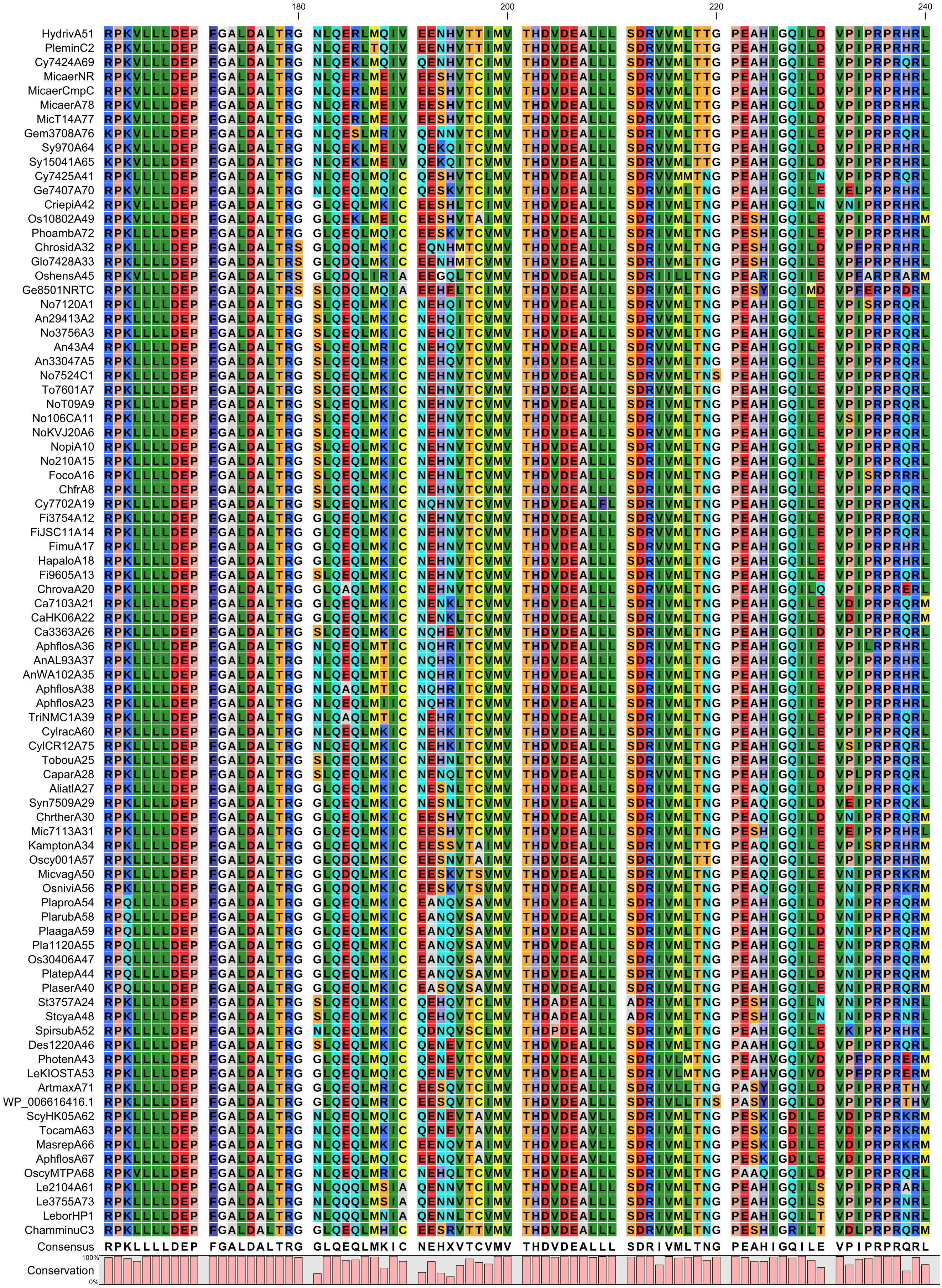
180
200
220
240
HydrivA51
PleminC2
Cy7424A69
MicaerNR
MicaerCmpC
MicaerA78
MicT14A77
Gem3708A76
Sy970A64
Sy15041A65
Cy7425A41
Ge7407A70
CriepiA42
Os10802A49
PhoambA72
ChrosidA32
Glo7428A33
OshensA45
Ge8501NRTC
No7120A1
An29413A2
No3756A3
An43A4
An33047A5
No7524C1
To7601A7
NoT09A9
No106CA11
NoKVJ20A6
NopiA10
No210A15
FocoA16
ChfrA8
Cy7702A19
Fi3754A12
FiJSC11A14
FimuA17
HapaloA18
Fi9605A13
ChrovaA20
Ca7103A21
CaHK06A22
Ca3363A26
AphflosA36
AnAL93A37
AnWA102A35
AphflosA38
AphflosA23
TriNMC1A39
CylracA60
CylCR12A75
TobouA25
CaparA28
AliatlA27
Syn7509A29
ChrtherA30
Mic7113A31
KamptonA34
Oscy001A57
MicvagA50
OsniviA56
PlaproA54
PlarubA58
PlaagaA59
Pla1120A55
Os30406A47
PlatepA44
PlaserA40
St3757A24
StcyaA48
SpirsubA52
Des1220A46
PhotenA43
LeKIOSTA53
ArtmaxA71
WP_006616416.1
ScyHK05A62
TocamA63
MasrepA66
AphflosA67
OscyMTPA68
Le2104A61
Le3755A73
LeborHP1
ChamminuC3
Consensus RPKLLLLDEP FGALDALTRG GLQEQLMKIC NEHXVTCVMV THDVDEALLL SDRIVMLTNG PEAHIGQILE VPIPRPRQRL
Conservation
100%
0%

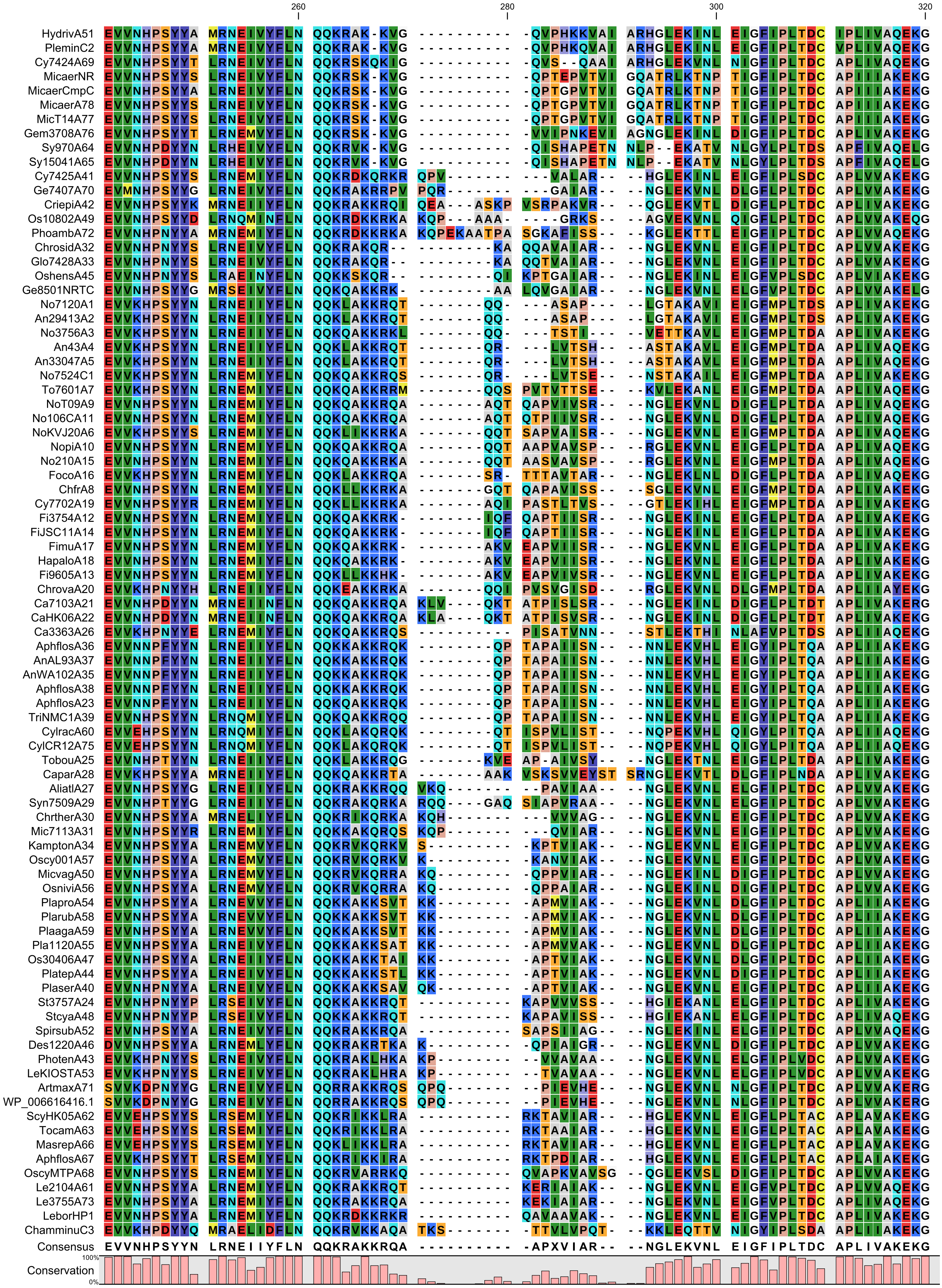
260
280
300
320
HydrivA51
PleminC2
Cy7424A69
MicaerNR
MicaerCmpC
MicaerA78
MicT14A77
Gem3708A76
Sy970A64
Sy15041A65
Cy7425A41
Ge7407A70
CriepiA42
Os10802A49
PhoambA72
ChrosidA32
Glo7428A33
OshensA45
Ge8501NRTC
No7120A1
An29413A2
No3756A3
An43A4
An33047A5
No7524C1
To7601A7
NoT09A9
No106CA11
NoKVJ20A6
NopiA10
No210A15
FocoA16
ChfrA8
Cy7702A19
Fi3754A12
FiJSC11A14
FimuA17
HapaloA18
Fi9605A13
ChrovaA20
Ca7103A21
CaHK06A22
Ca3363A26
AphflosA36
AnAL93A37
AnWA102A35
AphflosA38
AphflosA23
TriNMC1A39
CylracA60
CylCR12A75
TobouA25
CaparA28
AliatlA27
Syn7509A29
ChrtherA30
Mic7113A31
KamptonA34
Oscy001A57
MicvagA50
OsniviA56
PlaproA54
PlarubA58
PlaagaA59
Pla1120A55
Os30406A47
PlatepA44
PlaserA40
St3757A24
StcyaA48
SpirsubA52
Des1220A46
PhotenA43
LeKIOSTA53
ArtmaxA71
WP_006616416.1
ScyHK05A62
TocamA63
MasrepA66
AphflosA67
OscyMTPA68
Le2104A61
Le3755A73
LeborHP1
ChamminuC3
Consensus EVVNHPSYYN LRNEIIYFLN QQKRAKKRQA ---------- -APXVIAR-- --NGLEKVNL EIGFIPLTDC APLIVAKEKG
100%
Conservation
0%

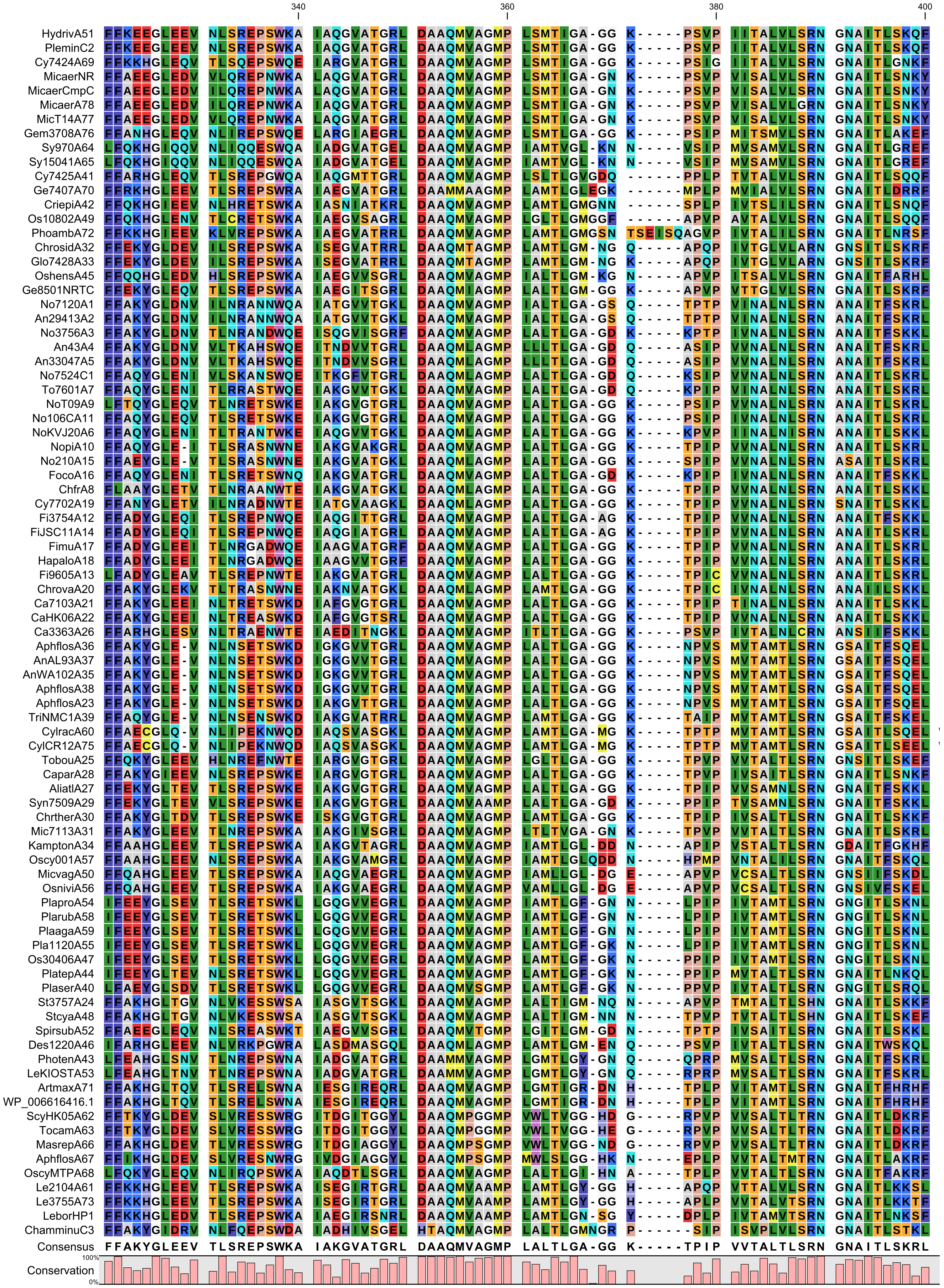
340
360
380
400
HydrivA51
PleminC2
Cy7424A69
MicaerNR
MicaerCmpC
MicaerA78
MicT14A77
Gem3708A76
Sy970A64
Sy15041A65
Cy7425A41
Ge7407A70
CriepiA42
Os10802A49
PhoambA72
ChrosidA32
Glo7428A33
OshensA45
Ge8501NRTC
No7120A1
An29413A2
No3756A3
An43A4
An33047A5
No7524C1
To7601A7
NoT09A9
No106CA11
NoKVJ20A6
NopiA10
No210A15
FocoA16
ChfrA8
Cy7702A19
Fi3754A12
FiJSC11A14
FimuA17
HapaloA18
Fi9605A13
ChrovaA20
Ca7103A21
CaHK06A22
Ca3363A26
AphflosA36
AnAL93A37
AnWA102A35
AphflosA38
AphflosA23
TriNMC1A39
CylracA60
CylCR12A75
TobouA25
CaparA28
AliatlA27
Syn7509A29
ChrtherA30
Mic7113A31
KamptonA34
Oscy001A57
MicvagA50
OsniviA56
PlaproA54
PlarubA58
PlaagaA59
Pla1120A55
Os30406A47
PlatepA44
PlaserA40
St3757A24
StcyaA48
SpirsubA52
Des1220A46
PhotenA43
LeKIOSTA53
ArtmaxA71
WP_006616416.1
ScyHK05A62
TocamA63
MasrepA66
AphflosA67
OscyMTPA68
Le2104A61
Le3755A73
LeborHP1
ChamminuC3
Consensus
FFAKYGLEEV TLSREPSWKA IAKGVATGRL DAAQMVAGMP LALTLGA-GG K-----TPIP VVTALTLSRN GNAITLSKRL
Conservation
100%
0%

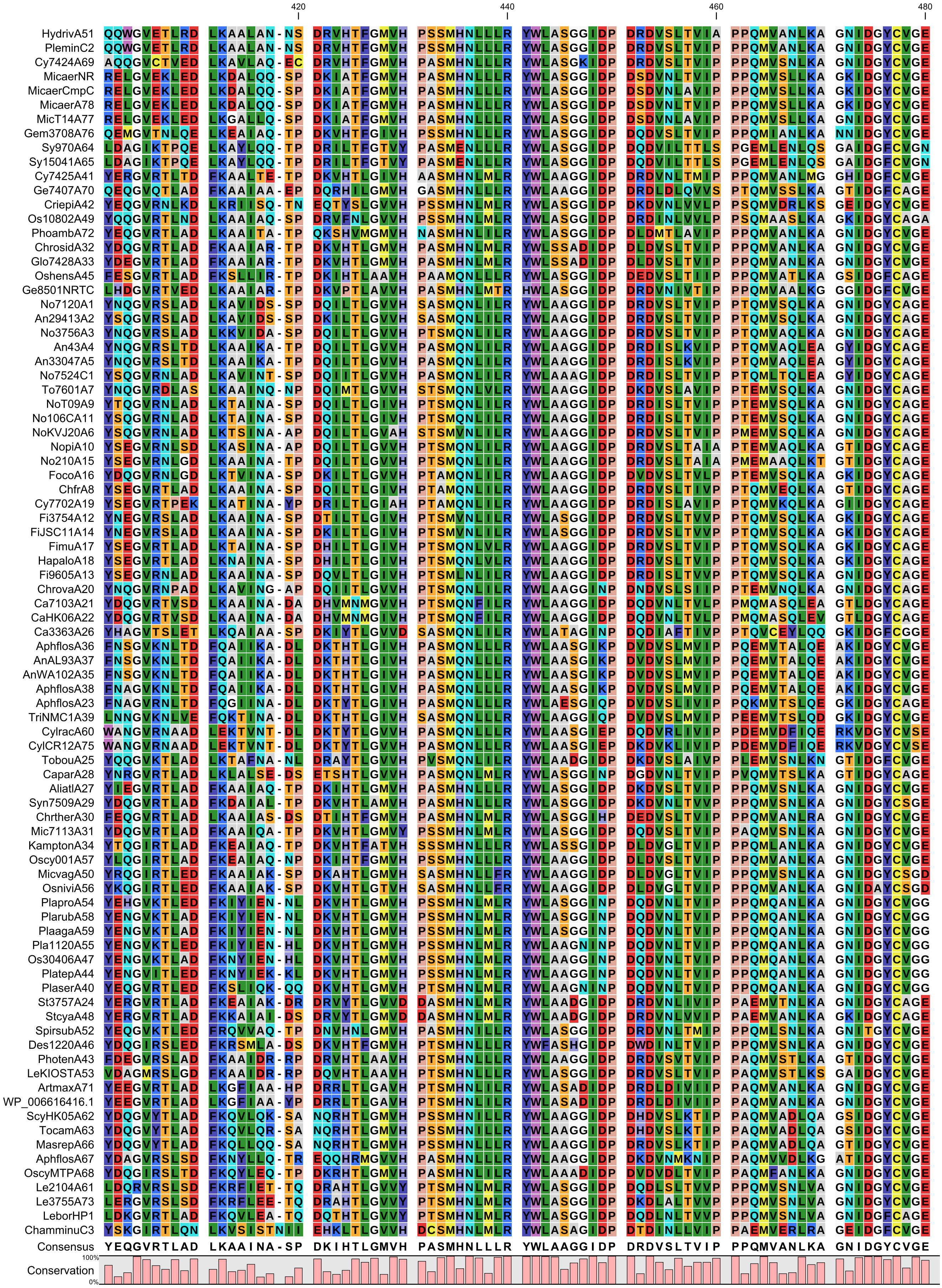

420
440
460
480
HydrivA51 QQWGVETLRD LKAALAN-NS DRVHTFGMVH PSSMHNLLLR YWLASGGIDP DRDVSLTVIA PPQMVANLKA GNIDGYCVGE
PleminC2 QQWGVETLRD LKAALAN-NS DRVHTFGMVH PSSMHNLLLR YWLASGGIDP DRDVSLTVIP PPQMVANLKA GNIDGYCVGE
Cy7424A69 AQQGVCTVED LKAVLAQ-EC DRVHTFGMVH PASMHNLLLR YWLASGKIDP DRDVSLTVIP PPQMVSNLKA GNIDGYCVGE
MicaerNR RELGVEKLED LKDALQQ-SP DKIATFGMVH PASMHNLLLR YWLASGGIDP DSDVNLTVIP PPQMVSLLKA GKIDGYCVGE
MicaerCmpC RELGVEKLED LKDALQQ-SP DKIATFGMVH PASMHNLLLR YWLASGGIDP DSDVNLAVIP PPQMVSLLKA GKIDGYCVGE
MicaerA78 RELGVEKLED LKDALQQ-SP DKIATFGMVH PASMHNLLLR YWLASGGIDP DSDVNLTVIP PPQMVSLLKA GKIDGYCVGE
MicT14A77 RELGVEKLED LKGALLQ-SP DKIATFGMVH PASMHNLLLR YWLASGGIDP DSDVNLAVIP PPQMVSLLKA GNIDGYCVGE
Gem3708A76 QEMGVTNLQE LKEAIAQ-TP DKVHTFGIVH PSSMHNLLLR YWLASGGIDP DQDVSLTVIP PPQMIANLKA NNIDGYCVGE
Sy970A64 LDAGIKTPQE LKAYLQQ-TP DRILTFGTVY PASMENLLLR YWLASGGIDP DQDVILTTLS PGEMLENLQS GAIDGFCVGN
Sy15041A65 LDAGIKTPQE LKAYLQQ-TP DRILTFGTVY PASMENLLLR YWLASGGIDP DQDVILTTLS PGEMLENLQS GAIDGFCVGN
Cy7425A41 YERGVRTLTD FKAALTE-TP DKVHTLGIVH AASMHNLMLR YWLAAGGIDP DRDVNLTMIP PPQMVANLMG GHIDGFCVGE
Ge7407A70 QEQGVQTLAD FKAAIAA-EP DQRHILGMVH GASMHNLLLR YWLASGGIDP DRDLDLQVVS PTQMVSSLKA GTIDGFCAGE
CriepiA42 YEQGVRNLAD LKRIISQ-TN EQTYSLGVVH PSSMHNLMLR YWLASGGIDP DKDVNLVVLP PSQMVDRLKS GEIDGYCVGE
Os10802A49 YQQGVRTLND LKAAIAQ-SP DRVFNLGVVH PSSMHNLLLR YWLASGGIDP DRDINLVVLP PSQMAASLKA GKIDGYCAGA
PhoambA72 YEQGVRTLAD LKAAITA-TP QKSHVMGMVH NASMHNLILR YWLASGGIDP DLDMTLAVIP PPQMVANLKA GNIDGYCVGE
ChrosidA32 YDQGVRTLAD FKAAIAR-TP DKVHTLGMVH PASMHNLMLR YWLSSADIDP DLDVSLTVIP PPQMVANLKA GNIDGYCVGE
Glo7428A33 YDEGVRTLAD FKAAIAR-TP DKVHTLGMVH PASMHNLMLR YWLSSADIDP DLDVSLTVIP PPQMVANLKA GNIDGYCVGE
OshensA45 FESGVRTLAD FKSLLIR-TP DKIHTLAAVH PAAMQNLLLR YWLASGGIDP DEDVSLTIIP PPQMVATLKA GSIDGFCAGE
Ge8501NRTC LHDGVRTVED LKAAIAR-TP DKVPTLAVVH PASMHNLMTR HWLASGGIDP DRDVNIVTIP PPQMVAALKG GGIDGFCVGE
No7120A1 YNQGVRSLAD LKAVIDS-SP DQILTLGVVH SASMQNLILR YWLAAGGIDP DRDVSLTVIP PTQMVSQLKA GNIDGYCAGE
An29413A2 YSQGVRSLAD LKAVIDS-SP DKILTLGVVH SASMQNLILR YWLAAGGIDP DRDVSLTVIP PTQMVSQLKA GNIDGYCAGE
No3756A3 YNQGVRSLAD LKKVIDA-SP DQILTLGVVH PTSMQNLILR YWLAAGGIDP DRDVSLTVIP PTQMVAQLKA GNIDGYCAGE
An43A4 YNQGVRSLTD LKAAIKA-TP DQILTLGVVH PASMQNLILR YWLAAGGIDP DRDISLKVIP PTQMVAQLEA GYIDGYCAGE
An33047A5 YNQGVRSLTD LKAAIKA-TP DQILTLGVVH PASMQNLILR YWLAAGGIDP DRDISLKVIP PTQMVAQLEA GYIDGYCAGE
No7524C1 YSQGVRNLAD LKAVINT-SP DQIITLGVVH PASMQNLILR YWLAAAGIDP DRDISLTVIP PTQMLTQLEA GYIDGYCAGE
To7601A7 YNQGVRDLAS LKAAINQ-NP DQIMTLGVVH STSMQNLVLR YWLAAGGIDP DKDVSLAVIP PTEMVSQLKA GNIDGYCAGE
NoT09A9 YTQGVRNLAD LKTAINA-SP DQILTLGIVH PTSMQNLILR YWLAAGGIDP DRDISLTVIP PTEMVSQLKA GNIDGYCAGE
No106CA11 YSQGVRNLAD LKTAINA-SP DQILTLGIVH PTSMQNLILR YWLAAGGIDP DRDISLTVIP PTEMVSQLKA GNIDGYCAGE
NoKVJ20A6 YSQGVRNLAD LKTSINA-AP DQILTLGVAH STSMVNLILR YWLAAGGIDP DRDVSLTVIP PMEMVSQLKA GNIDGYCAGE
NopiA10 YSEGVRNLSD LKASINA-AP DQILTLGVVH PTSMQNLILR YWLAAGGIDP DRDVSLTAIA PTEMVAQLKA GTIDGYCAGE
No210A15 YSEGVRNLGD LKAAINA-TP DQILTLGVVH PTSMQNLILR YWLAAGGIDP DRDVSLTAIA PMEMAAQLKT GTIDGYCAGE
FocoA16 YDQGVRNLGD LKTVINA-SP DKILTLGVVH PTAMQNLILR YWLAAGGIDP DVDVSLTVLP PTEMVSQLKA GKIDGYCAGE
ChfrA8 YSEGVRTLAD LKAAINA-SP DQILTLGIVH PTAMQNLILR YWLAAGGIDP DRDVSLTIVP PTQMVEQLKA GTIDGYCAGE
Cy7702A19 YSEGVRTPEK LKATINA-YP DRILTLGIAH PTAMQNLILR YWLAAGGIDP DRDISLAVIP PTQMVKQLKA GIIDGYCAGE
Fi3754A12 YNEGVRSLAD LKAAINA-SP DTILTLGIVH PTSMVNLILR YWLASGGIDP DRDVSLTVVP PTQMVSQLKA GKIDGYCAGE
FiJSC11A14 YNEGVRSLAD LKAAINA-SP DKILTLGIVH PTSMVNLILR YWLASGGIDP DRDVSLTVVP PTQMVSQLKA GKIDGYCAGE
FimuA17 YSEGVRTLAD LKTAINA-SP DHILTLGIVH PTSMQNLVLR YWLAAGGIDP DRDVSLTVIP PTQMVSQLKA GKIDGYCAGE
HapaloA18 YSEGVRTLAD LKNAINA-SP DHILTLGIVH PTSMQNLVLR YWLAAGGIDP DRDVSLTVIP PTQMVSQLKA GKIDGYCAGE
Fi9605A13 YSEGVRNLAD LKAAINA-SP DQVLTLGIVH PTSMLNLILR YWLAAGGIDP DRDISLTVVP PTQMVSQLKA GNIDGYCAGE
ChrovaA20 YNQGVRNPAD LKAVING-AP DQIITLGVVH PTSMQNLILR YWLAAGGINP DNDVSLSIIP PTEMVNQLKA GNIDGYCAGE
Ca7103A21 YDQGVRTVSD LKAAINA-DA DHVMNMGVVH PTSMQNFILR YWLAAGGIDP DQDVNLTVLP PMQMASQLEA GTLDGYCAGE
CaHK06A22 YDQGVRTVSD LKAAINA-DA DHVMNMGIVH PTSMQNFILR YWLAAGGIDP DQDVNLTVLP PMQMASQLEV GTLDGYCAGE
Ca3363A26 YHAGVTSLET LKQAIAA-SP DKIYTLGVVD SASMQNLILR YWLATAGINP DQDIAFTIVP PTQVCEYLQQ GKIDGFCGGE
AphflosA36 FNSGVKNLTD FQAIIKA-DL DKTHTLGIVH PASMQNLLLR YWLAASGIKP DVDVSLMVIP PQEMVTALQE AKIDGYCVGE
AnAL93A37 FNSGVKNLTD FQAIIKA-DL DKTHTLGIVH PASMQNLLLR YWLAASGIKP DVDVSLMVIP PQEMVTALQE AKIDGYCVGE
AnWA102A35 FNSGVKNLTD FQAIIKA-DL DKTHTLGIVH PASMQNLLLR YWLAASGIKP DVDVSLMVIP PQEMVTALQE AKIDGYCVGE
AphflosA38 FNAGVKNLTD FQAIIKA-DL DKTHTLGIVH PASMQNLLLR YWLAASGIKP DVDVSLMVIP PQEMVTALQE AKIDGYCVGE
AphflosA23 FNAGVRNLTD FQGIINA-DL DKTYTLGIVH PASMQNLLLR YWLAESGIQP DVDVSLIVIP PQKMVTSLQE GKIDGYCAGE
TriNMC1A39 LNNGVKNLVE FQKTINA-DL DKTHTLGIVH SASMQNLLLR YWLAAGGIQP DVDVSLMVIP PEEMVTSLQD GKIDGYCVGE
CylracA60 WANGVRNAAD LEKTVNT-DL DKTYTLGVVH PASMQNLLLR YWLAASGIEP DQDVRLIVIP PDEMVDFIQE RKVDGYCVSE
CylCR12A75 WANGVRNAAD LEKTVNT-DL DKTYTLGVVH PASMQNLLLR YWLAASGIEP DKDVKLIVIP PDEMVDFIQE RKVDGYCVSE
TobouA25 YQQGVKTLAD LKTAFNA-NL DRAYTLGVVH PVSMQNLILR YWLAADGIDP DKDVSLAIVP PLEMVSNLKN GTIDGFCVGE
CaparA28 YNRGVRTLAD LKLALSE-DS ETSHTLGVVH PASMQNLMLR YWLASGGINP DGDVNLTVIP PVQMVTSLKA GTIDGYCAGE
AliatlA27 YIEGVRTLAD FKAAIAQ-TP DKIHTLGMVH PASMHNLLLR YWLASGGIDP DKDVSLTVIP PPQMVSNLKA GNIDGYCVGE
Syn7509A29 YDQGVRTLAD FKDAIAL-TP DKVHTLAMVH PASMHNLLLR YWLASGGIDP DKDVNLTVVP PPQMVSNLKA GNIDGYCSGE
ChrtherA30 FEQGVRTLAD LKAAIAS-DS DTIHTFGMVH PASMHNLMLR YWLASGGIHP DEDVSLTVIP PPQMVANLRA GNIDGYCVGE
Mic7113A31 YDQGVRTLAD FKAAIQA-TP DKVHTLGMVY PSSMHNLLLR YWLASGGIDP DQDVSLTVIP PAQMVSNLKA GNIDGYCVGE
KamptonA34 YTQGIRTLAD FKEAIAQ-TP DKVHTFATVH SSSMHNLLLR YWLASSGIDP DLDVGLTVIP PPQMLANLKA GNIDGYCVGE
Oscy001A57 YLQGIRTLAD FKEAIAQ-NP DKIHTFGMVH PSSMHNLLLR YWLAAGGIDP DLDVSLTVIP PPQMVANLKA GNIDGYCVGE
MicvagA50 YRQGIRTLED FKAAIAK-SP DKAHTLGMVH SASMHNLLFR YWLAAGGIDP DLDVGLTVIP PPQMVSNLKA GNIDGYCSGD
OsniviA56 YKQGIRTLED FKAAIAK-SP DKVHTLGTVH SASMHNLLFR YWLAAGGIDP DLDVGLTVIP PPQMVANLKA GNIDAYCSGD
PlaproA54 YEHGVKTLED FKIYIEN-NL DKVHTLGMVH PSSMHNLMLR YWLASGGINP DQDVNLTVIP PPQMQANLKA GNIDGYCVGG
PlarubA58 YENGVKTLAD FKIYIEN-NL DKVHTLGMVH PSSMHNLMLR YWLASGGINP DQDVNLTVIP PPQMQANLKA GNIDGYCVGG
PlaagaA59 YENGVKTLAD FKIYIEN-NL DKVHTLGMVH PSSMHNLMLR YWLASGGINP DQDVNLTVIP PPQMQANLKA GNIDGYCVGG
Pla1120A55 YENGVKTLED FKIYIEN-HL DKVHTLGMVH PSSMHNLMLR YWLAAGNINP DQDVNLTVIP PPQMQANLKA GNIDGYCVGG
Os30406A47 YENGVKTLAD FKNYIEN-HL DKVHTLGMVH PSSMHNLMLR YWLAAGGINP DQDVNLTVIP PPQMQANLKA GNIDGYCVGG
PlatepA44 YENGVITLED FKNYIEK-KL DKVHTLGMVH PSSMHNLMLR YWLAAGGINP DQDVNLTVIP PPQMQANLKA GNIDGYCVGG
PlaserA40 YEQGVRTLED FKSLIQK-QQ DKVHTLGMVH PTSMHNLMLR YWLAAGNINP DQDVNLTVIP PPQMQANLKA GNIDGYCVGG
St3757A24 YERGVRTLAD FKEAIAK-DR DRVYTLGVVD DASMHNLMLR YWLAADGIDP DRDVNLVVIP PAEMVTNLKA GNIDGYCAGE
StcyaA48 YERGVRTLAD FKKAIAI-DS DRVYTLGMVD DASMHNLMLR YWLAADGIDP DRDVNLVVIP PAEMVANLKA GKIDGYCAGE
SpirsubA52 YEQGVKTLED FRQVVAQ-TP DNVHNLGMVH PASMHNILLR YWLASGGIDP DRDVNLTMIP PPQMLSNLKA GNITGYCVGE
Des1220A46 YDQGIRSLED FKRSMLA-DS DKVHTFGMVH PTSMHNLLLR YWFASHGIDP DWDINLTVIP PPQMVSNLKA GNIDGYCVGE
PhotenA43 FDEGVRSLAD FKAAIDR-RP DRVHTLAAVH PTSMHNLLLR YWLASGGIDP DRDVSVTVIP PAQMVSTLKA GTIDGYCVGE
LeKIOSTA53 VDAGMRSLGD FKAAIDR-RP DQVHTLAAVH PTSMHNLLLR YWLASGGIDP DRDVSLTVIP PAQMVSTLKS GAIDGYCVGE
ArtmaxA71 YEEGVRTLAD LKGFIAA-HP DRRLTLGAVH PTSMHNLILR YWLASADIDP DRDLDIVIIP PAQMVANLKA GNIDGYCVGE
WP_006616416.1 YEEGVRTLAD LKGFIAA-YP DRRLTLGAVH PTSMHNLILR YWLASADIDP DRDLDIVIIP PAQMVANLKA GNIDGYCVGE
ScyHK05A62 YDQGVYTLAD FKQVLQK-SA NQRHTLGMVH PSSMHNILLR YWLAAGGIDP DHDVSLKTIP PAQMVADLQA GSIDGYCVGE
TocamA63 YDQGVYTLAD FKQVLQR-SA NQRHTLGMVH PSSMHNILLR YWLAAGGIDP DHDVSLKTIP PAQMVADLQA GSIDGYCVGE
MasrepA66 YDQGVYTLAD FKQLLQQ-SA NQRHTLGMVH PSSMHNLLLR YWLAAGGIDP DRDVSLKTIP PAQMVADLQA GTIDGYCVGE
AphflosA67 YDAGVRSLSD FKNYLLQ-TR EQQHRMGVVH PASMHNLLLR YWLAAGGIDP DKDVNMKNIP PAQMVVDLKG ATIDGYCVGE
OscyMTPA68 YDQGIRSLTD FKQYLEQ-TP DKRHTLGMVH PASMHNLILR YWLAAADIDP DVDVDLTVIP PAQMFANLKA GNIDGYCVGE
Le2104A61 LDQRVRSLSD FKRFIET-TQ DRAHTLGVVY PTSMHNLMLR YWLASGGIDP DQDLSLTVVP PPQMVSNLVA GNIDGYCVGE
Le3755A73 LERGVRSLSD FKRFLEE-TQ DRAHTLGVVY PTSMHNLMLR YWLASGGIDP DKDLALTVVP PPQMVSNLVA GNIDGYCVGE
LeborHP1 LDKGVRTLAD FKQVLEA-TQ DQTHTLGVVY PTSMHNLMLR YWLASGGIDP DQDLNLTVVP PPQMVSNLVA GNIDGFCAGE
ChamminuC3 YSKGIRTLQN LKVSISTNII EHKLTLGVVH DCSMHNLMLR YWLASAGIDP DTDINLLVIP PAEMVERLRA GEIDGFCVGE
Consensus YEQGVRTLAD LKAAINA-SP DKIHTLGMVH PASMHNLLLR YWLAAGGIDP DRDVSLTVIP PPQMVANLKA GNIDGYCVGE
100%
Conservation
0%

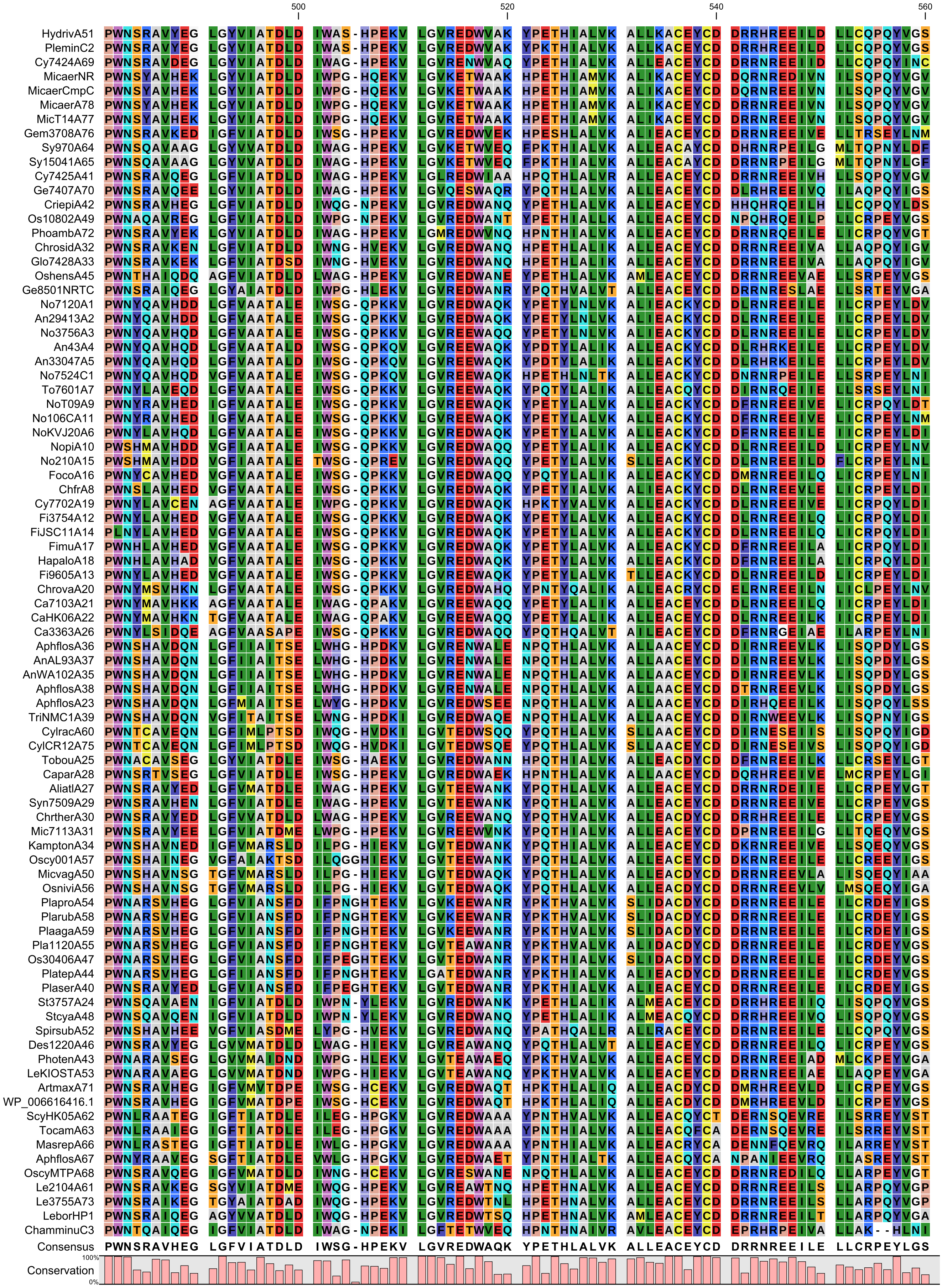
500
520
540
560
HydrivA51 PWNSRAVYEG LGYVIATDLD IWAS-HPEKV LGVREDWVAK YPETHIALVK ALLKACEYCD DRRHREEILD LLCQPQYVGS
PleminC2 PWNSRAVYEG LGYVIATDLD IWAS-HPEKV LGVREDWVAK YPKTHIALVK ALLKACEYCD DRRHREEILD LLCQPQYVGS
Cy7424A69 PWNSRAVDEG LGYVIATDLD IWAG-HPEKV LGVRENWVAQ YPETHIALVK ALLEACEYCD DRRNREEIID LLCQPQYINC
MicaerNR PWNSYAVHEK LGYVIATDLD IWPG-HQEKV LGVKETWAAK HPETHIAMVK ALIKACEYCD DQRNREDIVN ILSQPQYVGV
MicaerCmpC PWNSYAVHEK LGYVIATDLD IWPG-HQEKV LGVKETWAAK HPETHIAMVK ALIKACEYCD DQRNREEIVN ILSQPQYVGV
MicaerA78 PWNSYAVHEK LGYVIATDLD IWPG-HQEKV LGVKETWAAK HPETHIAMVK ALIKACEYCD DRRNREEIVN ILSQPQYVGV
MicT14A77 PWNSYAVHEK LGYVIATDLD IWPG-HQEKV LGVRETWAAK HPETHIAMVK ALIKACEYCD DRRNREEIVN ILSQPQYVGV
Gem3708A76 PWNSRAVKED IGFVIATDLD IWSG-HPEKV LGVREDWVEK HPESHLALVK ALIEACEYCD DRRNREEIVE LLTRSEYLNM
Sy970A64 PWNSQAVAAG LGYVVATDLD IWAG-HPEKV LGVKETWVEQ FPKTHIALVK ALLEACAYCD DHRNRPEILG MLTQPNYLDF
Sy15041A65 PWNSQAVAAG LGYVVATDLD IWAG-HPEKV LGVKETWVEQ FPKTHIALVK ALLEACAYCD DRRNRPEILG MLTQPNYLGF
Cy7425A41 PWNSRAVQEG LGFVIATDLD IWAG-HPEKV LGLREDWIAA HPQTHLALVR ALLEACEYCD DRRNREEIVH LLSQPQYVGV
Ge7407A70 PWNSRAVQEE LGYVIATDLD IWAG-HPEKV LGVQESWAQR YPQTHLALVK ALLEACEYCD DLRHREEIVQ ILAQPQYIGS
CriepiA42 PWNSRAVHEG LGFVIATDLD IWQG-NPEKV LGVREDWANQ YPETHLALVK ALLEACEYCD HHQHRQEILH LLCQPQYLDS
Os10802A49 PWNAQAVREG LGFVIATDLD IWPG-NPEKV LGVREDWANT YPETHIALLK ALLEACEYCD NPQHRQEILP LLCRPEYVGS
PhoambA72 PWNSRAVYEK LGYVIATDLD IWAG-HPEKV LGMREDWVNQ HPNTHIALVK ALLEACEYCD DRRNRQEILE LICRPQYVGT
ChrosidA32 PWNSRAVKEN LGFVIATDLD IWNG-HVEKV LGVREDWANQ HPETHLALIK ALLEACEYCD DRRNREEIVA LLAQPQYIGV
Glo7428A33 PWNSRAVKEK LGFVIATDSD IWNG-HVEKV LGVREDWANQ HPETHLALIK ALLEACEYCD DRRNREEIVA LLAQPQYIGV
OshensA45 PWNTHAIQDQ AGFVIATDLD LWAG-HPEKV LGVREDWANE YPETHLALVK AMLEACEYCD DRRNREEVAE LLSRPEYVGS
Ge8501NRTC PWNSRAIQEG LGYAIATDLD IWPG-HLEKV LGVREDWANR YPQTHVALVT ALLEACEYCD DRRNRESLAE LLSRTEYVGA
No7120A1 PWNYQAVHDD LGFVAATALE IWSG-QPKKV LGVREDWAQK YPETYLNLVK ALIEACKYCD DLRNREEILE ILCRPEYLDV
An29413A2 PWNYQAVHDD LGFVAATALE IWSG-QPKKV LGVREDWAQK YPETYLNLVK ALIEACKYCD DLRNREEILE LICRPEYLDV
No3756A3 PWNYQAVHQD LGFVAATALE IWSG-QPKKV LGVREEWAQQ YPETYLNLVK ALIEACKYCD DLRNREEILE LLCRPEYLDV
An43A4 PWNYQAVHQD LGFVAATALD IWSG-QPKQV LGVREEWAQK YPDTYLALIK ALLEACKYCD DLRHRKEILE LLCRPEYLDV
An33047A5 PWNYQAVHQD LGFVAATALD IWSG-QPKQV LGVREEWAQK YPDTYLALIK ALLEACKYCD DLRHRKEILE LLCRPEYLDV
No7524C1 PWNYQAVHQD VGFVAATALE IWSG-QPKQV LGVREEWAQK HPETHLNLTK ALLEACKYCD DNRNRPEILE LLSRPEYLNI
To7601A7 PWNYLAVEQD LGFVAATALE IWSG-QPKKV LGVREEWAQK YPQTYLALIK ALLEACQYCD DIRNRQEIIE LLSRSEYLNI
NoT09A9 PWNYRAVHED IGFVAATALE IWSG-QPKKV LGVREEWAQK YPETYLALVK ALLEACKYCD DFRNREEIVE LICRPQYLDT
No106CA11 PWNYRAVHED IGFVAATALE IWSG-QPKKV LGVREEWAQK YPETYLALVK ALLEACKYCD DFRNREEIVE LICRPEYLNM
NoKVJ20A6 PWNYLAVHQD LGFVAATALE IWSG-QPKKV LGVREDWAQK YPETYLALVK ALLEACKYCD DFRNREEILE IICRPEYLDI
NopiA10 PWSHMAVHDD VGFIAATALE IWSG-QPKKV LGVREDWAQQ YPETYLALIK ALLEACKYCD DLRNREEILD LICRPEYLNV
No210A15 PWSHMAVHDD VGFIAATALE TWSG-QPREV LGVREDWAQQ YPETYLALVK SLLEACKYCD DLRNREEILD FLCRPEYLNL
FocoA16 PWNYCAVHED VGFVAATALE IWSG-QPKKV LGVREDWAQQ YPQTYLALIK ALLEACKYCD DMRNREEILQ LICRPEYLNI
ChfrA8 PWNSLAVHED VGFVAATALE IWSG-QPKKV LGVREDWAQK YPETYIALVK ALIEACKYCD DLRNREEVLE LICRPEYLDI
Cy7702A19 PWNYLAVCEN AGFVAATALE IWPG-QPKKV LGVREDWAQK HPKTYVALVK ALLEACKYCD DLRNREEIVE LICRPQYLDI
Fi3754A12 PWNYLAVHED VGFVAATALE IWSG-QPKKV LGVREDWAQK YPETYLALVK ALLEACKYCD DLRNREEILQ LICRPQYLDI
FiJSC11A14 PLNYLAVHED VGFVAATALE IWSG-QPKKV LGVREDWAQK YPETYLALVK ALLEACKYCD DLRNREEILQ LICRPQYLDI
FimuA17 PWNHLAVHED VGFVAATALE IWSG-QPKKV LGVREDWAQK YPETYLALVK ALLEACKYCD DFRNREEILA LICRPQYLDI
HapaloA18 PWNHLAVHAD VGFVAATALE IWSG-QPKKV LGVREDWAQK YPETYLALVK ALLEACKYCD DFRNREEILA LICRPQYLDI
Fi9605A13 PWNYLAVHED VGFVAATALE IWSG-QPKKV LGVREEWAQK YPETYLALVK TLLEACKYCD DLRNREEILD LICRPEYLDI
ChrovaA20 PWNYMSVHKN LGFVAATALE IWSG-QPKKV LGVREDWAHQ YPNTYQALIK ALLEACRYCD ELRNREEILN LICLPEYLNV
Ca7103A21 PWNYMAVHKK AGFVAATALE IWAG-QPAKV LGVREEWAQQ YPETYLALIK ALLEACEYCD DLRNREEILQ IICRPEYLDI
CaHK06A22 PWNYMAVHKN TGFVAATALE IWAG-QPAKV LGVREEWAQQ YPETYLALIK ALLEACEYCD DLRNREEILK IICRPEYLDI
Ca3363A26 PWNYLSIDQE AGFVAASAPE IWSG-QPKKV LGVREDWAQQ YPQTHQALVT AILEACEYCD DFRNRGEIAE ILARPEYLNI
AphflosA36 PWNSHAVDQN LGFIIAITSE LWHG-HPDKV LGVRENWALE NPQTHLALVK ALLAACEYCD DIRNREEVLK LISQPDYLGS
AnAL93A37 PWNSHAVDQN LGFIIAITSE LWHG-HPDKV LGVRENWALE NPQTHLALVK ALLAACEYCD DIRNREEVLK LISQPDYLGS
AnWA102A35 PWNSHAVDQN LGFIIAITSE LWHG-HPDKV LGVRENWALE NPQTHLALVK ALLAACEYCD DIRNREEVLK LISQPDYLGS
AphflosA38 PWNSHAVDQN LGFIIAITSE LWHG-HPDKV LGVRENWALE NPQTHLALVK ALLAACEYCD DTRNREEVLK LISQPDYLGS
AphflosA23 PWNSHAVDQN LGFMIAITSE LWYG-HPDKV LGVREDWSEE NPQTHLALVK ALLAACEYCD DIRHQEEILK LISQPQYLSS
TriNMC1A39 PWNSHAVDQN VGFITAITSE LWNG-HPDKI LGVREDWAQE NPQTHLALVK ALLAACEYCD DIRNWEEVLK LISQPNYIGS
CylracA60 PWNTCAVEQN LGFIMLPTSD IWQG-HVDKI LGVTEDWSQQ YPQTHLALVK SLLAACEYCD DIRNESEIIS LISQPQYIGD
CylCR12A75 PWNTCAVEQN LGFIMLPTSD IWQG-HVDKI LGVTEDWSQE YPQTHLALVK SLLAACEYCD DIRNESEIVS LISQPQYIGD
ToboulA25 PWNACAVSEG LGYVIATDLE IWSG-HAEKV LGVREDWANN HPQTHIALVK ALLEACDYCD DFRNREEILK LLCRSEYLGT
CaparA28 PWNSRTVSEG LGFVIATDLE IWSG-HPEKV LGVREDWAEK HPNTHLALVK ALLAACEYCD DQRHREEIVE LMCRPEYLGI
AliatlA27 PWNSRAVYED LGFVMATDLD IWAG-HPEKV LGVTEEWANK YPQTHLALVK ALLEACEYCD DRRNRDEIIE LLCRPEYVGT
Syn7509A29 PWNSRAVHEN LGFVIATDLE IWAG-HPEKV LGVTEEWANK YPQTHLALVK ALLEACEYCD DRRNREEIVE LLCRPEYVGS
ChrtherA30 PWNSRAVYED LGFVVATDLD LWAG-HPEKV LGVREEWANQ YPQTHVALVK ALLEACDYCD DRRHREEILE LLCRPEYVGS
Mic7113A31 PWNSRAVYEE LGFVIATDME LWPG-HPEKV LGVREEWVNK YPQTHIALVK ALLEACEYCD DPRNREEILG LLTQEQYVGS
KamptonA34 PWNSHAVNED IGFVMARSLD ILPG-HIEKV LGVTEDWANK YPQTHIALVK ALLEACEYCD DKRNREEIVE LLSQEQYVGS
Oscy001A57 PWNSHAINEG VGFAIAKTSD ILQGGHIEKV LGVTEEWANK YPQTHLALVK ALLEACEYCD DKRNREEILE LLCREEYIGS
MicvagA50 PWNSHAVNSG TGFVMARSLD ILPG-HIEKV LGVTEDWAQK YPQTHLALVK ALLEACDYCD DRRNREEVLA LISQEQYIAA
OsniviA56 PWNSHAVNSG TGFVMARSLD ILPG-HIEKV LGVTEDWANQ YPQTHLALVK ALLEACDYCD DRRNREEVLV LMSQEQYIGA
PlaproA54 PWNARSVHEG LGFVIANSFD IFPNGHTEKV LGVKEEWANR YPKTHVALVK SLIDACDYCD DRRNREEILE ILCRDEYIGS
PlarubA58 PWNARSVHEG LGFVIANSFD IFPNGHTEKV LGVKEEWANR YPKTHVALVK SLIDACDYCD DRRNREEILE ILCRDEYIGS
PlaagaA59 PWNARSVHEG LGFVIANSFD IFPNGHTEKV LGVKEEWANR YPKTHVALVK SLIDACDYCD DRRNREEILE ILCRDEYIGS
Pla1120A55 PWNARSVHEG LGFVIANSFD IFPNGHTEKV LGVTEAWANR YPKTHIALVK ALIDACDYCD DRRNREEILE ILCRDEYVGS
Os30406A47 PWNARSVHEG LGFVIANSFD IFPEGHTEKV LGVTEDWANR YPKTHIALVK SLIDACDYCD DRRNREEILE ILCRDEYVGS
PlatepA44 PWNARSVHEG LGFIIANSFD IFPNGHTEKV LGATEDWANR YPKTHIALVK ALIEACDYCD DRRNREEILE ILCRDEYVGS
PlaserA40 PWNSRAVYED LGFVIANSFD IFPEGHTEKV LGVTEDWANR YPKTHIALVK ALLEACEYCD DRRNREEILE ILCRDEYIGS
St3757A24 PWNSQAVAEN IGFVIATDLD IWPN-YLEKV LGVREDWANK YPETHLALIK ALMEACEYCD DRRHREEIIQ LISQPQYVGS
StcyaA48 PWNSQAVQEN IGFVIATDLD IWPN-YLEKV LGVREDWANQ YPETHLALIK ALMEACQYCD DRRHREEIVQ LISQPQYVGS
SpirsubA52 PWNSHAVHEE VGFVIASDME LYPG-HVEKV LGVREAWANQ YPATHQALLR ALLRACEYCD DRRNREEILE LLCQPQYVGS
Des1220A46 PWNSRAVYEG LGVVMATDLD LWAG-HIEKV LGVREAWANQ YPQTHLALVT ALLEACEYCD DRRNREEILQ LLCRPEYVGS
PhotenA43 PWNARAVSEG LGVVMAIDND IWPG-HLEKV LGVTEAWAEQ YPKTHVALVK ALLEACEYCD DRRNREEIAD MLCKPEYVGA
LeKIOSTA53 PWNARAVAEG LGVVMATDND IWPG-HIEKV LGVTEAWANQ YPKTHVALVK ALLEACEYCD DRRNREEIAE LLAQPEYVGA
ArtmaxA71 PWNSRAVHEG IGFVMVTDPE IWSG-HCEKV LGVREDWAQT HPKTHLALIQ ALLEACDYCD DMRHREEVLD LICRPEYVGS
WP_006616416.1 PWNSRAVHEG IGFVMATDPE IWSG-HCEKV LGVREDWAQT HPKTHLALIQ ALLEACDYCD DMRHREEVLD LICRPEYVGS
ScyHK05A62 PWNLRAATEG IGFTIATDLE ILEG-HPGKV LGVREDWAAA YPNTHVALVK ALLEACQYCT DERNSQEVRE ILSRREYVST
TocamA63 PWNLRAAIEG IGFTIATDLE ILEG-HPGKV LGVREDWAAA YPNTHIALVK ALLEACQFCA DERNSQEVRE ILSRREYVST
MasrepA66 PWNLRASTEG IGFTIATDLE IWLG-HPGKV LGVREDWAAA YPNTHIALVK ALLEACRYCA DENNFQEVRQ ILARREYVST
AphflosA67 PWNYRAAVEG SGFTIATDLE VWLG-HPGKV LGVREDWAET YPNTHIALTK ALLEACQYCA NPANIEEVRQ ILASREYVST
OscyMTPA68 PWNSRAVQEG IGFVMATDLD IWNG-HCEKV LGVRESWANE NPQTHLALVK ALLEACEYCD DRRNREDILQ LLARPEYVGT
Le2104A61 PWNSRAVKEG SGYVIATDME IWQG-HPEKV LGVREAWTNQ HPETHNALVK ALLEACEYCD DRRNREEILS LLARPQYVGP
Le3755A73 PWNSRAIKEG TGYAIATDAD IWQG-HPEKV LGVREDWTNL HPETHNALVK ALLEACEYCD DRRNREEILS LLARPQYVGP
LeborHP1 PWNSRAIQEG AGYVVATDLD IWQG-HPEKV LGVREDWTSQ HPETHNALVK AMLEACEYCD DRRNREEILT LLARPQYVGA
ChamminuC3 PWNTQAIQEG IGFVIATDLD IWAG-NPEKI LGFTETWVEQ HPNTHNAIVR AVLEACEYCD EPRHRPEIVA LLAK--HLNI
Consensus PWNSRAVHEG LGFVIATDLD IWSG-HPEKV LGVREDWAQK YPETHLALVK ALLEACEYCD DRRNREEILE LLCRPEYLGS
100%
Conservation
0%

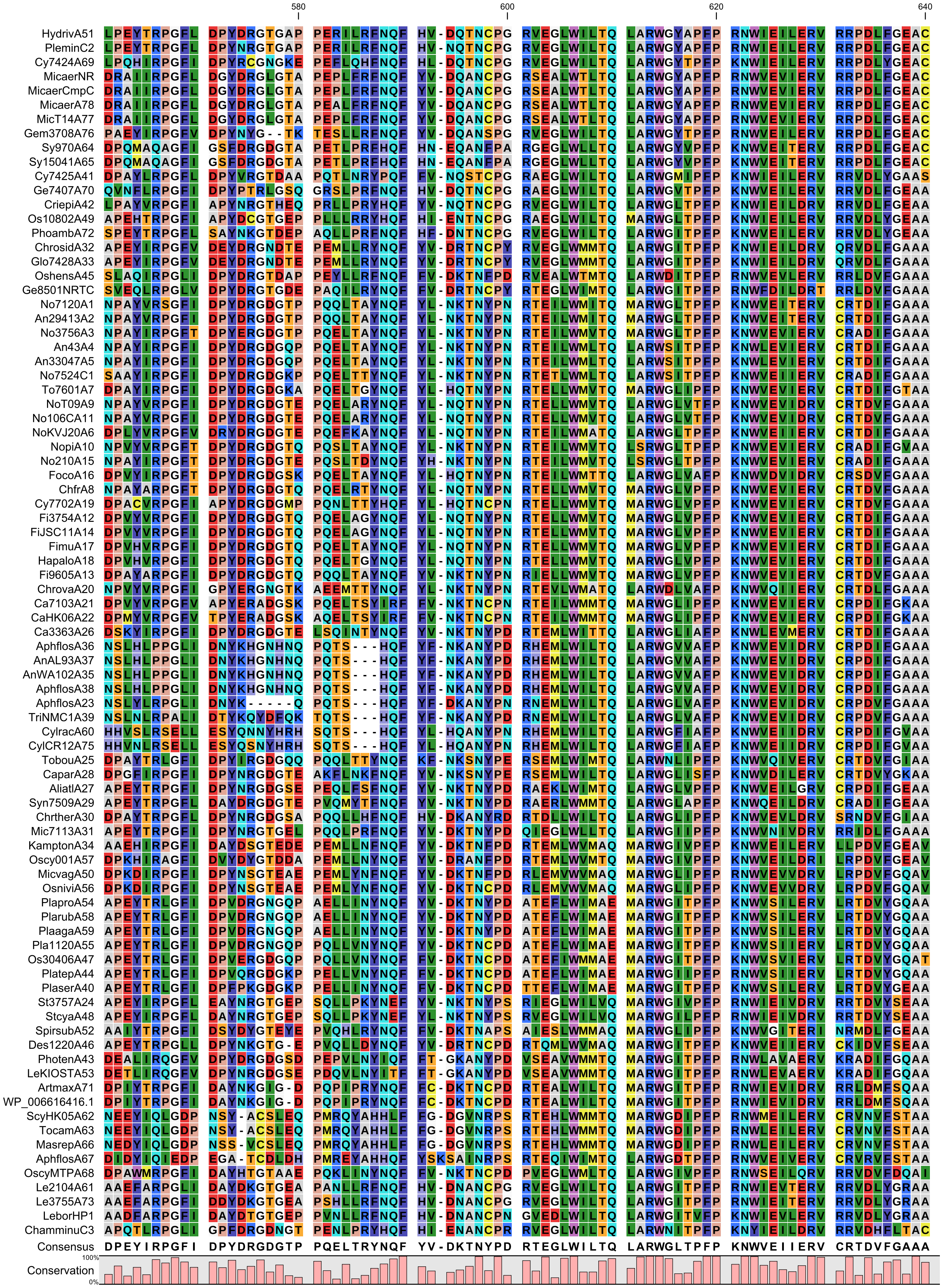

580
600
620
640
HydrivA51
PleminC2
Cy7424A69
MicaerNR
MicaerCmpC
MicaerA78
MicT14A77
Gem3708A76
Sy970A64
Sy15041A65
Cy7425A41
Ge7407A70
CriepiA42
Os10802A49
PhoambA72
ChrosidA32
Glo7428A33
OshensA45
Ge8501NRTC
No7120A1
An29413A2
No3756A3
An43A4
An33047A5
No7524C1
To7601A7
NoT09A9
No106CA11
NoKVJ20A6
NopiA10
No210A15
FocoA16
ChfrA8
Cy7702A19
Fi3754A12
FiJSC11A14
FimuA17
HapaloA18
Fi9605A13
ChrovaA20
Ca7103A21
CaHK06A22
Ca3363A26
AphflosA36
AnAL93A37
AnWA102A35
AphflosA38
AphflosA23
TriNMC1A39
CylracA60
CylCR12A75
ToboudA25
CaparA28
AliatlA27
Syn7509A29
ChrtherA30
Mic7113A31
KamptonA34
Oscy001A57
MicvagA50
OsniviA56
PlaproA54
PlarubA58
PlaagaA59
Pla1120A55
Os30406A47
PlatepA44
PlaserA40
St3757A24
StcyaA48
SpirsubA52
Des1220A46
PhotenA43
LeKIOSTA53
ArtmaxA71
WP_006616416.1
ScyHK05A62
TocamA63
MasrepA66
AphflosA67
OscyMTPA68
Le2104A61
Le3755A73
LeborHP1
ChamminuC3
Consensus
DPEYIRPGFI DPYDRGDGTP PQELTRYNQF YV-DKTNYPD RTEGLWILTQ LARWGLTPFP KNWVEIIERV CRTDVFGAAA
Conservation
100%
0%

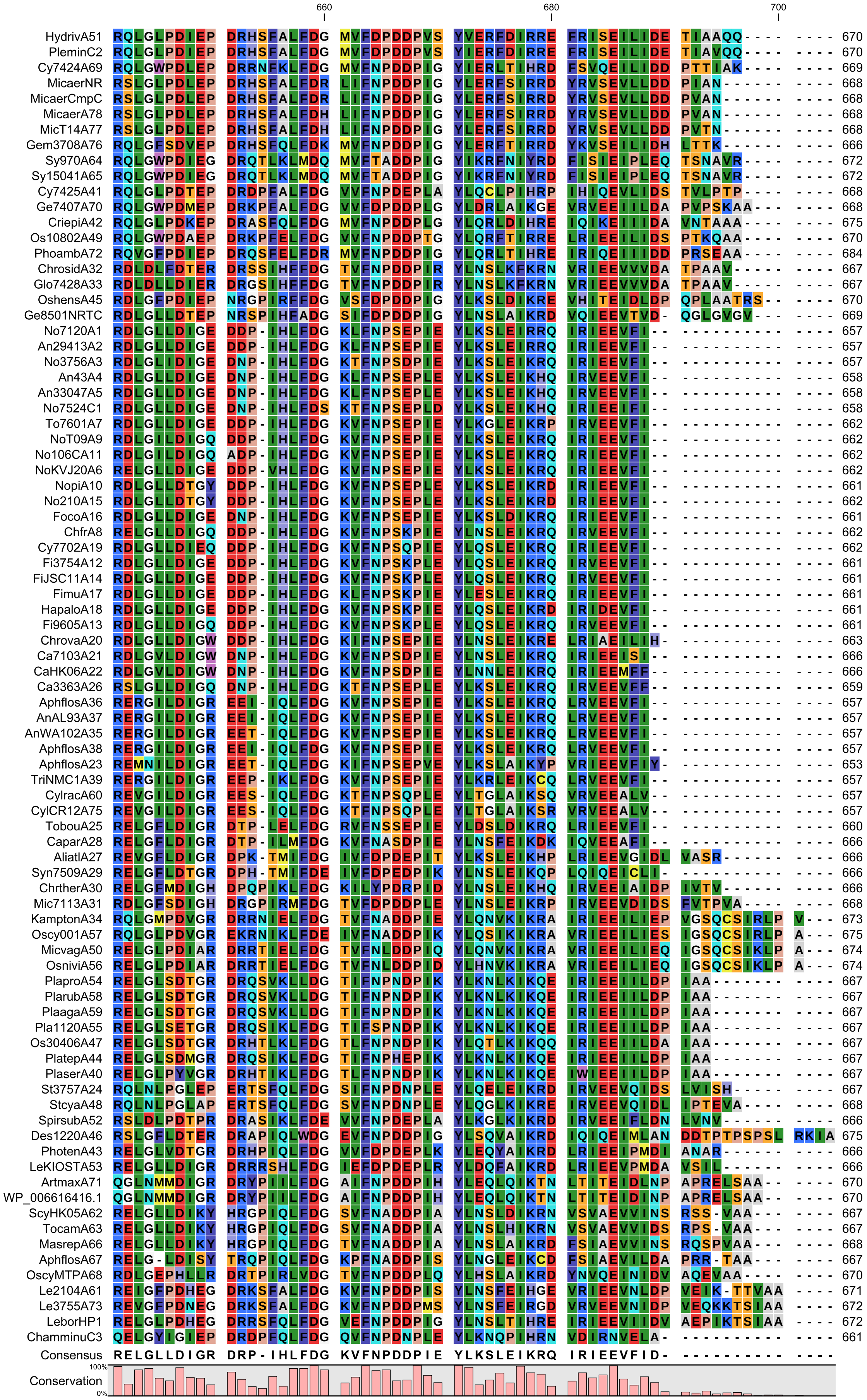
660
680
700
HydrivA51 670
PleminC2 670
Cy7424A69 669
MicaerNR 668
MicaerCmpC 668
MicaerA78 668
MicT14A77 668
Gem3708A76 666
Sy970A64 672
Sy15041A65 672
Cy7425A41 668
Ge7407A70 668
CriepiA42 675
Os10802A49 670
PhoambA72 684
ChrosidA32 667
Glo7428A33 667
OshensA45 670
Ge8501NRTC 669
No7120A1 657
An29413A2 657
No3756A3 657
An43A4 658
An33047A5 658
No7524C1 658
To7601A7 662
NoT09A9 662
No106CA11 662
NoKVJ20A6 662
NopiA10 661
No210A15 662
FocoA16 661
ChfrA8 662
Cy7702A19 662
Fi3754A12 661
FiJSC11A14 661
FimuA17 661
HapaloA18 661
Fi9605A13 661
ChrovaA20 663
Ca7103A21 666
CaHK06A22 666
Ca3363A26 659
AphflosA36 657
AnAL93A37 657
AnWA102A35 657
AphflosA38 657
AphflosA23 653
TriNMC1A39 657
CylracA60 657
CylCR12A75 657
TobouA25 660
CaparA28 666
AliatlA27 666
Syn7509A29 666
ChrtherA30 666
Mic7113A31 668
KamptonA34 673
Oscy001A57 675
MicvagA50 674
OsniviA56 674
PlaproA54 667
PlarubA58 667
PlaagaA59 667
Pla1120A55 667
Os30406A47 667
PlatepA44 667
PlaserA40 667
St3757A24 667
StcyaA48 668
SpirsubA52 666
Des1220A46 675
PhotenA43 666
LeKIOSTA53 666
ArtmaxA71 670
WP_006616416.1 670
ScyHK05A62 667
TocamA63 667
MasrepA66 668
AphflosA67 667
OscyMTPA68 670
Le2104A61 671
Le3755A73 672
LeborHP1 672
ChamminuC3 661
Consensus RELGLLDIGR DRP-IHLFDG KVFNPDDPIE YLKSLEIKRQ IRIEEVFID- ---------- ----
100%
Conservation
0%
