# Supplementary material for: Molecular interaction of nitrate transporter proteins with recombinant glycinebetaine results in efficient nitrate uptake in the cyanobacterium Anabaena PCC 7120
Source: PLoS One. 2021 Nov 18;16(11):e0257870. doi: 10.1371/journal.pone.0257870 (PMC8601584; doi:10.1371/journal.pone.0257870)
Supplement: S6 Fig — (PDF) [file pone.0257870.s006.pdf]

```
                                20                    40                    60                    80
                                 |                     |                     |                     |
MiaerNR     --TTQPFLLV SNVAKEYPSP -EGVYTVLDG VDLQVNEGEF VCIIGHSGCG KSTLLNMVAG FSSPSRGQVL LQDKEVKEPG
MiaerBIT    --TTQPFLLV SNVAKEYPSP -EGVYTVLDG VELQVNEGEF VCIIGHSGCG KSTLLNMVAG FSSPSRGQVL LQDKEVKEPG
PrhoA25     --TSPPLLTI DSVSKVYDTP -QGVYPVLDQ ISLTVESGEF ISVIGHSGCG KSTLLNMVAG FSKPSSGSVY LEGQTVKGPG
Cy1200A71   --EDQAFLNI SGVSKVYPTE -KGEYVVLDG VDLQVKKGEF ICLIGHSGCG KSTLLNMVAG FHGPSSGTVT LHDKAITEPG
No7120A1    PQKTDNFLVV EGVSKIYPTP -EGPYTVLDG IDLKVREGEF VCLIGHSGCG KSTLLNMISG FNTPSEGVVL LQDKPITEPG
No3756A2    TQK-ENFLVV EGVSKIYPTP -EGPYTVLDG IDLKIGEGEF VCLIGHSGCG KSTLLNMISG FNTPSEGVVL LQDQPITEPG
No7524C1    TRKADNFLVV EGVSKIYPTP -EGPYTVLDG IDLKVREGEF VCLIGHSGCG KSTLLNMISG FNTPTDGVVL LQDQPITEPG
NopiA3      T-KADNFLVI EGVSKIYPTP -EGPYTVLDG IDLKVREGEF VCLIGHSGCG KSTLLNMISG FNTPTDGVVM LQDQPITEPG
An33047A4   TPKADNFLVI EGVTKIYPTP -EGPYTVLDG IDLRVREGEF ICLIGHSGCG KSTLLNMISG FNTPTDGVVM LQDQPITEPG
An43A11     TPKADNFLVI EGVTKIYPTP -EGPYTVLDG IDLRVREGEF ICLIGHSGCG KSTLLNMISG FNTPTDGVVM LQDQPITEPG
CaHK06A7    THPSDNFLVV EGVSKIYPTV -DGPYTVLDG IDLKVREGEF VCLIGHSGCG KSTLLNMISG FNTPTEGIVL LQDTPITEPG
Ca7103A9    APKADNFLVV EGVSKIYPTV -DGPYTVLDG IDLKVREGEF VCLIGHSGCG KSTLLNIISG FNTPTEGVVL LQDTPITEPG
Ca336A17    PQKSDNFLVI EGVNKVYPTA -DGPYTVLDG VNLTVREGEF VCLIGHSGCG KSTLLNMVSG FNKPTDGVVL LQGQPITEPG
NoKVJ20A5   TKKAEDFLVI EGVSKIYPTS -EGPYTVLDG VDLKVREGEF VCIIGHSGCG KSTLLNMVSG FNTPTDGIVL LQDKPITEPG
To7601A6    KQKADDFLVI EGVSKIYPTP -EGPYTVLDG VDLKVREGEF VCIIGHSGCG KSTLLNMVAG FNTPSEGIVL LQDQPITEPG
ChfrA10     IQKAGNFLEI EGVTKIYPTP -DGPHTVLDG VNLKVSEGEF ICIIGHSGCG KSTLLNMVSG FNTPTEGVVC LQGQPIAEPG
Cy7702A22   TQKAENFLEI KGVSKIYPTS -DGPYTVLEE VNLQVREGEF VCIIGHSGCG KSTLLNMVSG LNKPTEGVIC LQGQPIAEPG
Fi9339A13   TQKAENFLVI EDVNKIYPTA -DGPYTVLDG VNLKVREGEF VCIIGHSGCG KSTLLNMVSG FNTPTDGVVL LQNQAITEPG
MalaA29     TQKAENFLVI EDVNKIYPTA -DGPYTVLDG VNLKVREGEF VCIIGHSGCG KSTLLNMVSG FNTPTDGVVL LQNQAITEPG
FiJSC11A14  THKAEDFLVI EGVNKIYPTA -DGPYTVLDG VNLKVRESEF VCIIGHSGCG KSTLLNMVSG FNTPTDGVVM LQNKPITEPG
Fi3754A15   THKAEDFLVI EGVNKIYPTA -DGPYTVLDG VNLKVREGEF VCIIGHSGCG KSTLLNMVSG FNTPTDGVVM LQNNPITEPG
Fi9605A12   TQKAEDFLVI EGVNKIYPTP -DGPHTVLDG VNLKVREGEF VCIIGHSGCG KSTLLNMVSG FNTPTDGVVL LQNNPITEPG
FocoA8      ATKAENFLVI EGVSKVYATP -DGPYTVLDG VDLQVREGEF ICIIGHSGCG KSTLLNMVSG FNTPTDGVVI LQDQPITEPG
AbWA102A18  TQKKEDFLVI DGVNKIYPTD -KGSYTVLDD INLKVSEGEF ICLIGHSGCG KSTLLNMVSG FNKPTNGVVW LQGQPITEPG
AphflosA19  TQKKEDFLVI DGVNKIYPTD -KGSYTVLDD INLKVSEGEF ICLIGHSGCG KSTLLNMVSG FNKPTNGVVW LQGQPITEPG
TriNMC1A16  TQRKEDFLVI DGVNKIYPTA -EGPYTVLDD INLKVHEGEF ICLIGHSGCG KSTLLNMVSG FNKPTNGVVW LQGQPITEPG
CyraA24     HQKSRDFLFI DGVNKIYPTA -EGPYTVLDN ISLKVQEGEF ICFIGHSGCG KSTLLNMISG FNQPTNGMVW LQGQPITEPG
RabrA33     HQKSRDFLFI DGVNKIYPTA -EGPYTVLDN ISLKVQEGEF ICFIGHSGCG KSTLLNMISG FNQPTNGMVW LQGEPVTEPG
TobouA21    TQKPEPFLVI EGVTKIYPTA -NGPYTVLDG IDFTVNEGEF ICIIGHSGCG KSTLLNMISG FNQPTDGVVL LQNQPITEPG
CapaA38     N---EPFLVI ENLTKIYPSA -KGPNVVLDG INLSVKEGEF ICVIGHSGCG KSTLLNMVSG FNTPTEGEVR LQNQQITEPG
ChaminC7    ----MPFLAI DRVTKVYPTA -NGPATILDG IDLEVNEGEF ICLIGHSGCG KSTLLNMVSG FNTPTSGTVT LQDRIISEPG
Sy6714A61   TQETTAFLAI DNVTKVYPTP -QGPYTVLDG VNLEVKEGEF ICIIGHSGCG KSTLLNMVSG FNQPTQGTVK LKNKVITAPG
Ge3709A73   TLEKTDFLTI QKVSKVYSTA -NGDYTVLDN VNLTVKEGEF ICVIGHSGCG KSTLLNMISG FNQPTTGTVK LKDKIIAEPG
AlatA20     TYKNDPFLVI ENVSKIYPTP -TGPYTVLDG VELTVREGEF VCLIGHSGCG KSTLLNMVAG FNTPSDGEVR LQSKAVKEPG
Sy7509A23   TTKNEHFLVI ENVSKIYPTP -TGPYTVLDG VELSVGEGEF VCLIGHSGCG KSTLLNMVAG FNTPSDGEVR LNSKAITEPG
CrepC8      ALKRDPFLVI DNVSKVFPTP -NGSFIALED VNLTVYEGEF VCLIGHSGCG KSTLLNMVAG FNKPTSGEIT LQAKRITEPG
KampA81     SLKSEPFLTI EDVSKVYETP -KGPYTVLDG VNLTINEGEF ICLIGHSGCG KSTLLNMVAG FNQPTTGKLK LKGKWIEEPG
Cy7822A34   ---KAF-VTV ENVSKVYPTP -KGPYTVLEN VNLTVAEGEF ICVIGHSGCG KSTLLNMISG FATPTTGSVR VNGKPITKPG
Cy7424A36   ---EAF-VAI ENVSKVYPTP -KGPYTVLEN VNLTVAQGEF ICVIGHSGCG KSTLLNMVSG FATPSTGSVR VNGKPVTKPG
Cy0110A35   ---NNLSVVI ENVSKVYPTP -KGPYTVLQD VNLTVQEGEF LCVIGHSGCG KSTLLNMVSG FATPSQGSVL VNGKPVKKPG
HyriA41     ---DKAFLVI EGVSKVYPTP -TGPYTVLEN VNLTVNEGEF ICVIGHSGCG KSTLLNMVSG FASPTTGSVR VNGKPIKKPG
PlemiC3     ---DKAFLVI EGVSKVYPTP -AGPYTVLEN VNLTVNEGEF ICVIGHSGCG KSTLLNMVSG FASPTTGSVR VNGKPIKKPG
StcyC5      ---NASFLNI EGVSKVYPTP -TGPYTVLEN VNLTVNEGEF ICVIGHSGCG KSTLLNMVSG FAHPTTGSVT VGGKIITEPG
MyGI1A70    ---NSSFVEI KNVSKVYPTL -TGSYTVLEN VNLTVDEGEF ICVIGHSGCG KSTLLNMVSG FTRPTTGMVT VAGKTITEPG
Ge3709A47   ---QDSFLTI ENVSKVYPTP -TGSYTVLQD VNLNVNQGEF ICVIGHSGCG KSTLLNMVSG FAQPTSGSVN LKGKPITKPG
GeherA54    ---QDSFLTI ENVSKIYPTP -KGDYTVLKD VNLTVNQGEF ICVIGHSGCG KSTLLNMVSG FSQPTFGSVK LKGKPITKPG
Cy7425A46   ---TQDFLVI KDVSKVYPTP -KGPYTVLED VNLTIAEGEF ICLIGHSGCG KSTLLNMVSG FNQPTAGEVK LQGKPILKPG
PsbicA85    -----AFLRI EKVSKIYPTP -QGEYVVLKD VNLAIAEGEF ICLIGHSGCG KSTLLNMVGG FSKPSIGEVK VNGKLITKPG
PsRCrA88    -----AFLRI EKVSKIYPTP -QGEYVVLKD VNLAIAEGEF ICLIGHSGCG KSTLLNMVGG FSKPSIGEVK VNGKLITKPG
Ps6802A69   ---RNAFLSI EQVAKVYPTS -TGPYVVLEG VSLTVAQGEF ICVIGHSGCG KSTLLNMVSG FAKPTRGEVR LRGHLITKPG
CygrC2      ----QPLLRF DALGKVYPTP -TGPYPVLEN VNLQVEQGEF ICVIGHSGCG KSTLLNMVSG FSTPTAGQVL LNGRPIEKPG
LeboA83     SRAANPFLVI DQVSKVYPTK -NGDYTVLKD VNLTVNQGEF ICVIGHSGCG KSTLLNMVSG FSKPTTGSVL LQGKPIEKPG
LeO77A58    ---DMPLLSL ENISKVYPTP -NGPYTVLED VNLSIQAGEF ICVIGHSGCG KSTLLNMVSG FSTPTTGSVL LKGVPIVKPG
ChsiA39     ---REPFLVI EDVSKVYPTS -KGPYTVLDG VNLTVAEGEF ICVIGHSGCG KSTLLNMVAG FNHPTSGEVR LGSKPITKPG
Gl7428A40   ---REPFLVM EDISKVYPTS -KGPYTVLDG VNLTVAEGEF ICVIGHSGCG KSTLLNMVAG FNHPTSGEVR LGSKPITKPG
Sy7509C4    ---TSPFLLI EDVSKVYPTA NKGTYTVLEG VNLTVNEGEF ICVIGHSGCG KSTLLNMVSG FTTPSSGRVE LNSVPITQPG
Mi7113C10   ---PEAFLTI EDVSKVYPTK -KGSFTVLDG VNLTVEEGEF ICVIGHSGCG KSTLLNMVSG FAFPTHGEVR VQGKPITKPG
AncyC9      --SHQPFLAI KDVCKVYPTK -NGPFTVLDG VNLNVEQGEF LCVIGHSGCG KSTLLNMVSG FNFPTTGQVL LEGEPITKPG
CystC12     --SRQPFLEI KDVCKVYPTK -KGPFTVLDG VNLNVEKGEF LCVIGHSGCG KSTLLNMVSG FNFPTTGQVL LEGEPITKPG
An90A65     --SRQPFLEI KDVCKVYPTK -NGPFTVLDG VNLNVEKGEF LCVIGHSGCG KSTLLNMVSG FNFPTTGQVL LEGEPITKPG
DociA48     --NRQPFLEI KEVCKVYPTK -KGPFTVLDG VNLNVEKGEF ICVIGHSGCG KSTLLNMVSG FNFPTTGQVL LEGQPITKPG
Ca7507A67   --NRQPFLEI KDVSKVYPTK -KGPFTVLDG VNLNVNQGEF ICVIGHSGCG KSTLLNMVSG FNFPTSGQVL LEGQPITQPG
No7107C11   --NSRHFLEI RDVSKVYPTK -KGPFTVLDG VNLNVNQGEF ICVIGHSGCG KSTLLNMVSG FNFPTTGQVL LEGQPITKPG
NopunA49    --SRRPFLEI KDVTKVYPTK -KGPFTVLDG VNLNVEQGEF ICVIGHSGCG KSTLLNMVSG FNFPTSGEVL LEGEPITKPG
NocaA53     --TRHPFLQI KDVTKVYPTK -KGPFTVLDG VNLNVEQGEF ICVIGHSGCG KSTLLNMVSG FNFPTSGQVL LEGQPITKPG
No7524A56   --SR-PFLEI KDVTKVYPTK -KGPFTVLDG VNLNVEQGEF ICVIGHSGCG KSTLLNMVSG FNTPTTGQVL LEGQPITKPG
Ab33047A55  --GHRSFLEI QDVTKVYPTK -KGPFTVLDG VNLNVEQGEF ICVIGHSGCG KSTLLNMVSG FATPTTGQVL LEGQPITKPG
HapA62      --NRQPYLEI KEVSKVYPTK -NGPFTVLDG VNLNVGEGEF ICVIGHSGCG KSTLLNMVSG FNNPTDGQVL LEGKPITKPG
ChfrA66     --NRKPYLQI QDVTKVYPTK -NGPFTVLDG VNLNVGEGEF ICVIGHSGCG KSTLLNMVSG FNSPTSGQVL LEGKPITKPG
Sc1581A64   D-VRKPFLEI KDVSKVYPTK -KGPFTVLDG VNLNVGQGEF ICVIGHSGCG KSTLLNMVSG FNFPTSGQVL LEGKPITKPG
AphflosA59  --NHQSFLKI ENVTKIYPTK -KGPFTVLDG INLDVQKGEF ICVIGHSGCG KSTLLNMVSG FNTPTTGQVL LEGQPIVKPG
ChrthA50    --NRQAFVQI KDVCKVYPTK -NGPFTVLDG VNLSVYEGEF ICVIGHSGCG KSTLLNMVSG FAHPTSGDVL VDGSPVTKPG
Os10802A37  ---RDEFLVM ENVTKVYKTP -KGPFTVLDG VNLTVQQGEF ICVIGHSGCG KSTLLNMVSG FAQPTTGRVD LQGAPITKPG
Pl001A63    --AKKPFVVF DNVSKTYKTS -RGPYTVLKD VNLTINRGEF ICVIGHSGCG KSTLLNMVSG FNTPSTGSVT VGGEPVTKPG
NodnodA72   ---DQGFLTF DQVSKSFPTP -KGPYPVLEN VNLTIQEGEF VCFIGHSGCG KSTLLSLVSG FQQATGGHVT LKGQPILKPG
Ly8106A74   ---HQAFLNF ENVSKIYSTP -QGPYTVLEN VNLTVEQGEF ICVIGHSGCG KSTLLSMVSG FNQPTDGNVL LQGKAIQQPG
MivagA79    ---RQPFLVM DSVYKEYPN- --G-YRALEN VNLNVAEGEF ITLIGHSGCG KSTLLNMVSG FSHPSSGTVT INGQRITKPG
PlseA87     ---NQPYLVF DHVSKVYPTP -KGPYTVLKD VNLTINEGEF ICVIGHSGCG KSTLLNMVSG FNIPSDGKVT LRSKPILKPG
ScHK05A42   S-RSNPFLLI EEVSKIYPTP -KGPFTVLQG VNLTVHEGEF ICVIGHSGCG KSTLLNMVAG FTKPTHGNVL LGSKPITQPG
TocaA43     S-RSNPFLLI EEVSKIYPTP -KGPFTVLQG VNLTVNEGEF ICVIGHSGCG KSTLLNMVAG FTKPTHGNVL LGSKPITQPG
MareA44     SKRSNPFLVI EDASKVYPTP -KGPYTVLDG VNLNVHEGEF ICVIGHSGCG KSTLLNMVAG FTKPTSGGVL LNSKPITQPG
SchofA45    PTRPQPFLVM ENVSKVYPTP -KGPFVVLED VNLTVNQGEF ICVIGHSGCG KSTLLNMVAG FTKPTGGSVL LNSKPITQPG
SctoA60     ATRQNPFLLI ENVSKIYPTS -KGPYTVLQD VNLTVNEGEF ICVIGHSGCG KSTLLNMVSG FATPTHGSVL LNSKPITQPG
NesphA68    ---PEPFLNI ENVSKIYPTP -KGDYVVLEQ VNLDVRQGEF VCVIGHSGCG KSTLLNMVAG FSEPTAGLVT LQGKRITEPG
ChaminC13   ---KAPFLLI ENVAKIYPTA -KGNYTVLEN VDLSVNKGEF ICVIGHSGCG KSTLLNMVGG FSQPSVGRVE LKGEPILKPG
Sy33AbA26   PVAGLPLLCL QNVGKTYFTS -AGPFVALEN INLEVAAGEF VCIIGHSGCG KSTLINMVAG FSTPTSGSIA LKGQPITSPG
ThelA27     -----QHLRI DGVSKVYPTA -NGDHVVLED IHLEVSSGEF ICFIGHSGCG KTTLLNMVAG FTRPTRGEIT LNGVPVRHPG
TheloA80    ----ESFLRL EQVSKVYPTR -NGPFTVLTD ITLDVQAGEF ICVIGHSGCG KTTLLNMISG FVRPTTGEVR LKGQPIKEPG
ThNK55aA89  ----ESFLRL EQVSKVYPTR -SGPFTVLTD ITLDVQAGEF ICVIGHSGCG KTTLLNMISG FVRPTTGEVR LKGQPIKEPG
Sy23BaA28   ----EPYLSL ENVSKTYSTP -KGDYPVLRD INLSVNEGEF VCLVGHSGCG KTTLLNMVSG FAQPTSGSVK LKGQPITAPG
GlviA76     ---LEPLLAI RNVSKSFPTP -NGPYTVLED VNLAVRQGEF VCIIGHSGCG KTTLLNMVAG FATPTTGEVL LRSEPIARPG
```

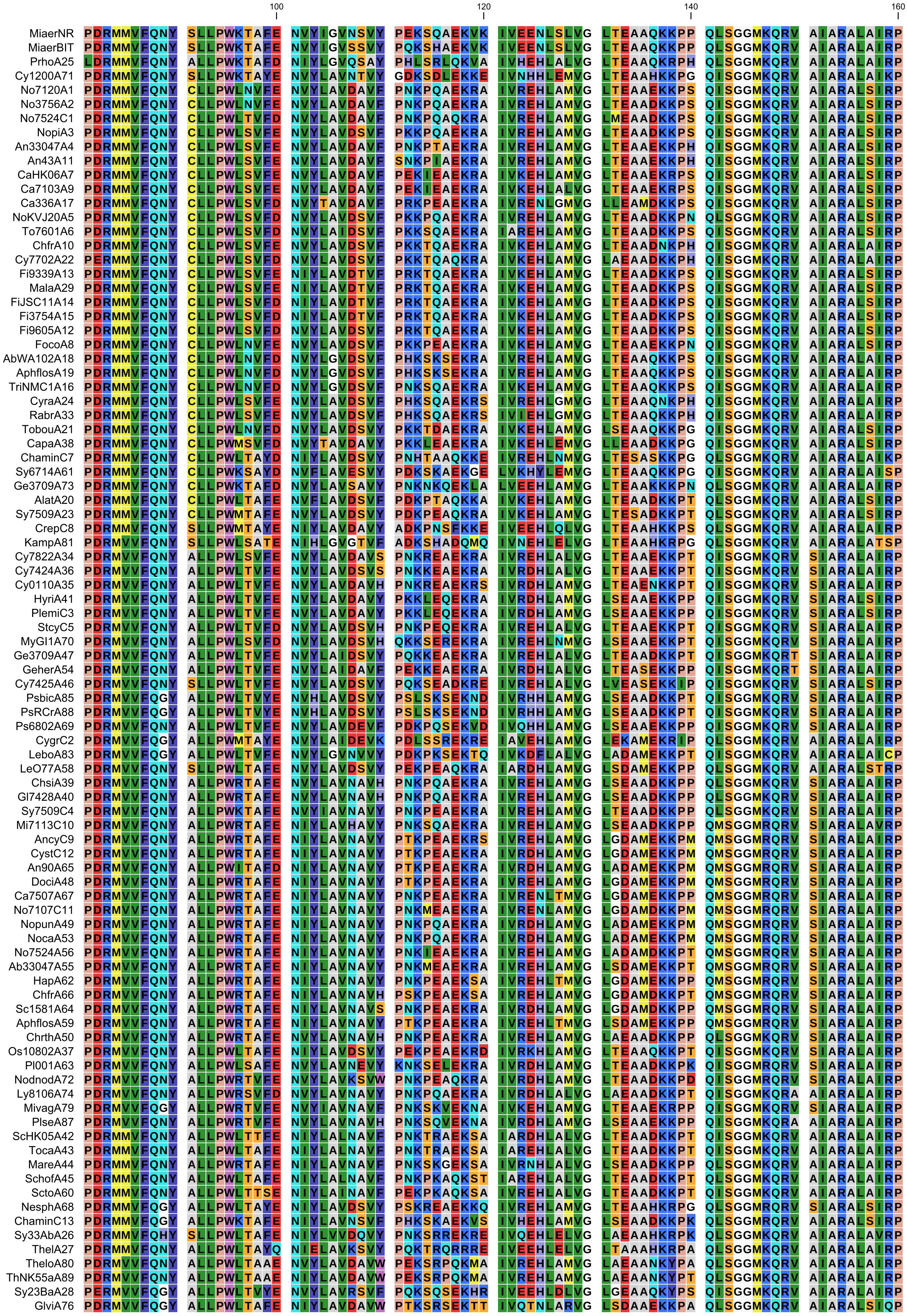
100
120
140
160
MiaerNR
MiaerBIT
PrhoA25
Cy1200A71
No7120A1
No3756A2
No7524C1
NopiA3
An33047A4
An43A11
CaHK06A7
Ca7103A9
Ca336A17
NoKVJ20A5
To7601A6
ChfrA10
Cy7702A22
Fi9339A13
MalaA29
FiJSC11A14
Fi3754A15
Fi9605A12
FocoA8
AbWA102A18
AphflosA19
TriNMC1A16
CyraA24
RabrA33
TobouA21
CapaA38
ChaminC7
Sy6714A61
Ge3709A73
AlatA20
Sy7509A23
CrepC8
KampA81
Cy7822A34
Cy7424A36
Cy0110A35
HyriA41
PlemiC3
StcyC5
MyGI1A70
Ge3709A47
GeherA54
Cy7425A46
PsbicA85
PsRCrA88
Ps6802A69
CygrC2
LeboA83
LeO77A58
ChsiA39
Gl7428A40
Sy7509C4
Mi7113C10
AncyC9
CystC12
An90A65
DociA48
Ca7507A67
No7107C11
NopunA49
NocaA53
No7524A56
Ab33047A55
HapA62
ChfrA66
Sc1581A64
AphflosA59
ChrthA50
Os10802A37
Pl001A63
NodnodA72
Ly8106A74
MivagA79
PlseA87
ScHK05A42
TocaA43
MareA44
SchofA45
SctoA60
NesphA68
ChaminC13
Sy33AbA26
TheIA27
TheloA80
ThNK55aA89
Sy23BaA28
GlviA76

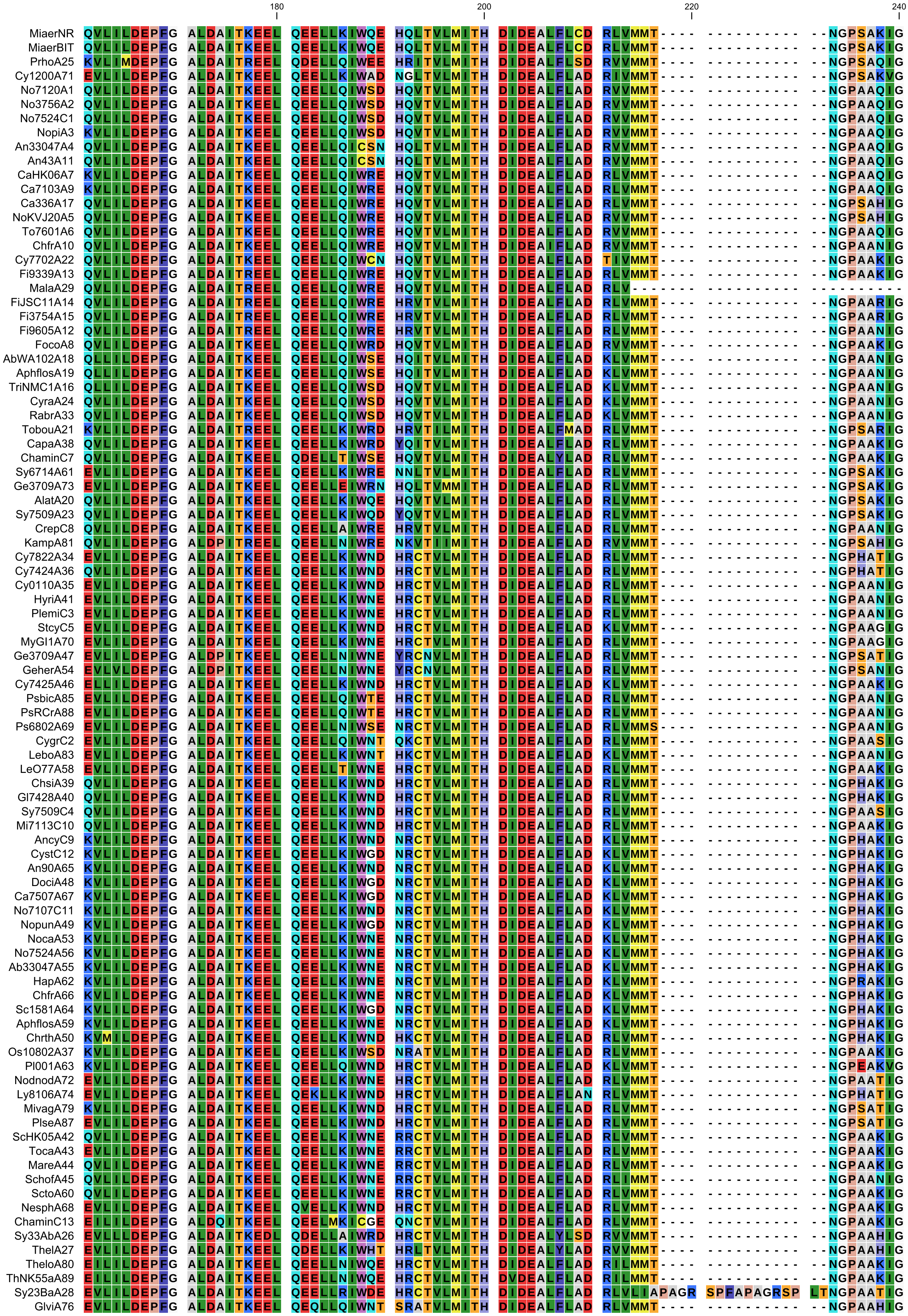
180
200
220
240
MiaerNR
MiaerBIT
PrhoA25
Cy1200A71
No7120A1
No3756A2
No7524C1
NopiA3
An33047A4
An43A11
CaHK06A7
Ca7103A9
Ca336A17
NoKVJ20A5
To7601A6
ChfrA10
Cy7702A22
Fi9339A13
MalaA29
FiJSC11A14
Fi3754A15
Fi9605A12
FocoA8
AbWA102A18
AphflosA19
TriNMC1A16
CyraA24
RabrA33
TobouA21
CapaA38
ChaminC7
Sy6714A61
Ge3709A73
AlatA20
Sy7509A23
CrepC8
KampA81
Cy7822A34
Cy7424A36
Cy0110A35
HyriA41
PlemiC3
StcyC5
MyGl1A70
Ge3709A47
GeherA54
Cy7425A46
PsbicA85
PsRCrA88
Ps6802A69
CygrC2
LeboA83
LeO77A58
ChsiA39
Gl7428A40
Sy7509C4
Mi7113C10
AncyC9
CystC12
An90A65
DociA48
Ca7507A67
No7107C11
NopunA49
NocaA53
No7524A56
Ab33047A55
HapA62
ChfrA66
Sc1581A64
AphflosA59
ChrthA50
Os10802A37
Pl001A63
NodnodA72
Ly8106A74
MivagA79
PlseA87
ScHK05A42
TocaA43
MareA44
SchofA45
SctoA60
NesphA68
ChaminC13
Sy33AbA26
ThelA27
TheloA80
ThNK55aA89
Sy23BaA28
GlviA76

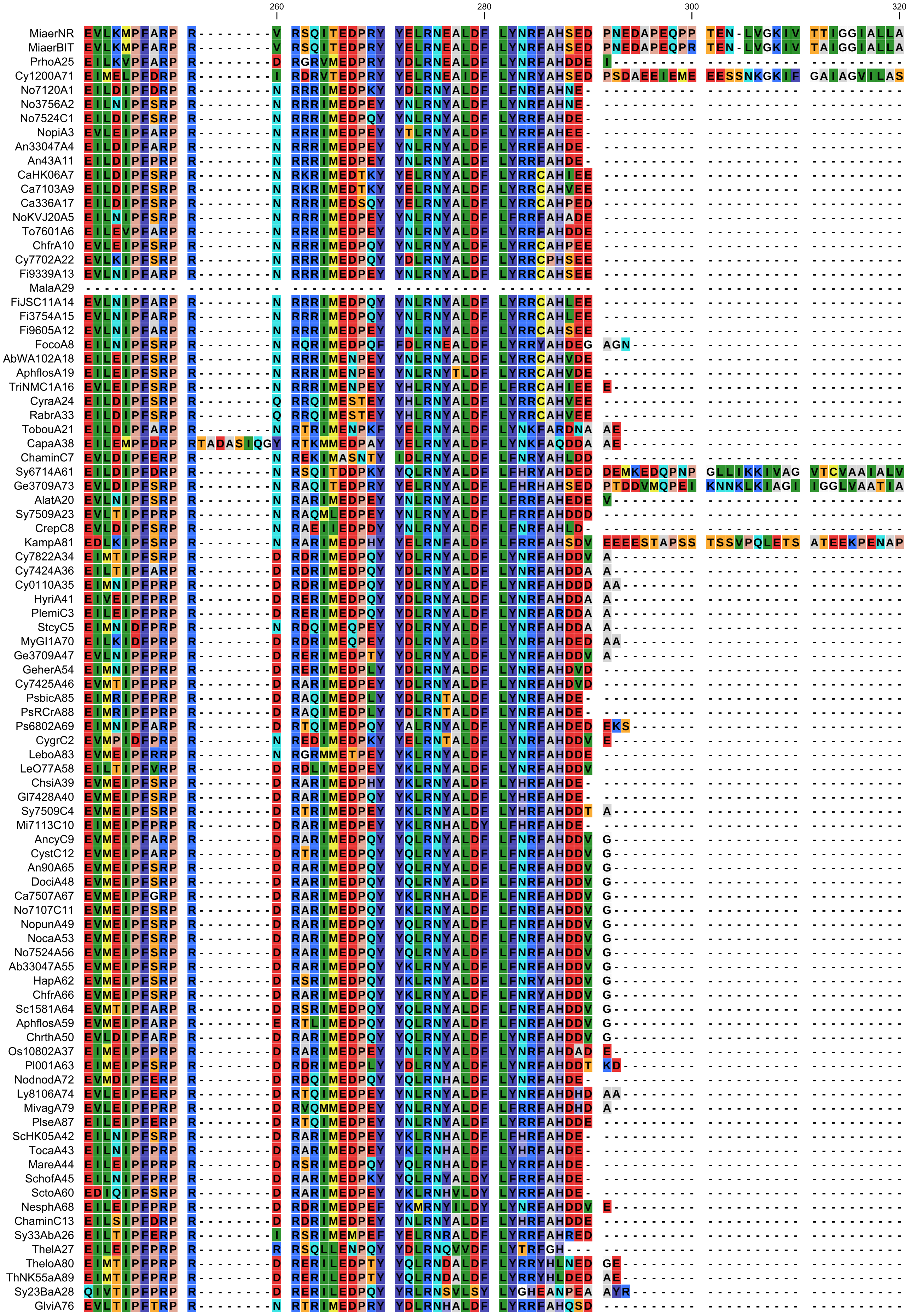
260
280
300
320
MiaerNR
MiaerBIT
PrhoA25
Cy1200A71
No7120A1
No3756A2
No7524C1
NopiA3
An33047A4
An43A11
CaHK06A7
Ca7103A9
Ca336A17
NoKVJ20A5
To7601A6
ChfrA10
Cy7702A22
Fi9339A13
MalaA29
FiJSC11A14
Fi3754A15
Fi9605A12
FocoA8
AbWA102A18
AphflosA19
TriNMC1A16
CyraA24
RabrA33
TobouA21
CapaA38
ChaminC7
Sy6714A61
Ge3709A73
AlatA20
Sy7509A23
CrepC8
KampA81
Cy7822A34
Cy7424A36
Cy0110A35
HyriA41
PlemiC3
StcyC5
MyGl1A70
Ge3709A47
GeherA54
Cy7425A46
PsbicA85
PsRCrA88
Ps6802A69
CygrC2
LeboA83
LeO77A58
ChsiA39
Gl7428A40
Sy7509C4
Mi7113C10
AncyC9
CystC12
An90A65
DociA48
Ca7507A67
No7107C11
NopunA49
NocaA53
No7524A56
Ab33047A55
HapA62
ChfrA66
Sc1581A64
AphflosA59
ChrthA50
Os10802A37
Pl001A63
NodnodA72
Ly8106A74
MivagA79
PlseA87
ScHK05A42
TocaA43
MareA44
SchofA45
SctoA60
NesphA68
ChaminC13
Sy33AbA26
ThelA27
TheloA80
ThNK55aA89
Sy23BaA28
GlviA76

```
                                   340                 360                 380                 400
                                     |                   |                   |                   |
MiaerNR      FVGFSLIQMF TSKTANTTNP ATIEAPR--- ---------- ---------- ---------- ---------- ----------
MiaerBIT     FVGFSLIQMF TSKTANTTNP ATIEAPR--- ---------- ---------- ---------- ---------- ----------
PrhoA25      ---------- ---------- ---------- ---------- ---------- ---------- ---------- ----------
Cy1200A71    IVGITMWQSF SNRPTATPN- -SVESVN--- ---------- ---------- ---------- ---------- ----------
No7120A1     ---------- ---------- ---------- ---------- ---------- ---------- ---------- ----------
No3756A2     ---------- ---------- ---------- ---------- ---------- ---------- ---------- ----------
No7524C1     ---------- ---------- ---------- ---------- ---------- ---------- ---------- ----------
NopiA3       ---------- ---------- ---------- ---------- ---------- ---------- ---------- ----------
An33047A4    ---------- ---------- ---------- ---------- ---------- ---------- ---------- ----------
An43A11      ---------- ---------- ---------- ---------- ---------- ---------- ---------- ----------
CaHK06A7     ---------- ---------- ---------- ---------- ---------- ---------- ---------- ----------
Ca7103A9     ---------- ---------- ---------- ---------- ---------- ---------- ---------- ----------
Ca336A17     ---------- ---------- ---------- ---------- ---------- ---------- ---------- ----------
NoKVJ20A5    ---------- ---------- ---------- ---------- ---------- ---------- ---------- ----------
To7601A6     ---------- ---------- ---------- ---------- ---------- ---------- ---------- ----------
ChfrA10      ---------- ---------- ---------- ---------- ---------- ---------- ---------- ----------
Cy7702A22    ---------- ---------- ---------- ---------- ---------- ---------- ---------- ----------
Fi9339A13    ---------- ---------- ---------- ---------- ---------- ---------- ---------- ----------
MalaA29      ---------- ---------- ---------- ---------- ---------- ---------- ---------- ----------
FiJSC11A14   ---------- ---------- ---------- ---------- ---------- ---------- ---------- ----------
Fi3754A15    ---------- ---------- ---------- ---------- ---------- ---------- ---------- ----------
Fi9605A12    ---------- ---------- ---------- ---------- ---------- ---------- ---------- ----------
FocoA8       ---------- ---------- ---------- ---------- ---------- ---------- ---------- ----------
AbWA102A18   ---------- ---------- ---------- ---------- ---------- ---------- ---------- ----------
AphflosA19   ---------- ---------- ---------- ---------- ---------- ---------- ---------- ----------
TriNMC1A16   ---------- ---------- ---------- ---------- ---------- ---------- ---------- ----------
CyraA24      ---------- ---------- ---------- ---------- ---------- ---------- ---------- ----------
RabrA33      ---------- ---------- ---------- ---------- ---------- ---------- ---------- ----------
TobouA21     ---------- ---------- ---------- ---------- ---------- ---------- ---------- ----------
CapaA38      ---------- ---------- ---------- ---------- ---------- ---------- ---------- ----------
ChaminC7     ---------- ---------- ---------- ---------- ---------- ---------- ---------- ----------
Sy6714A61    GIGLVQTFSS KGDSGPAPAV ESMEGY---- ---------- ---------- ---------- ---------- ----------
Ge3709A73    GFTLFQSFNG NNGQSPTTNT EQISN----- ---------- ---------- ---------- ---------- ----------
AlatA20      ---------- ---------- ---------- ---------- ---------- ---------- ---------- ----------
Sy7509A23    ---------- ---------- ---------- ---------- ---------- ---------- ---------- ----------
CrepC8       ---------- ---------- ---------- ---------- ---------- ---------- ---------- ----------
KampA81      VTKAEVESAA SATKPFATIP FNASKDTKEA NMSSELNSHS SKSPESSPGK IMAFGAIWLA TTIGLTIVSV GSVANLQKET
Cy7822A34    ---------- ---------- ---------- ---------- ---------- ---------- ---------- ----------
Cy7424A36    ---------- ---------- ---------- ---------- ---------- ---------- ---------- ----------
Cy0110A35    ---------- ---------- ---------- ---------- ---------- ---------- ---------- ----------
HyriA41      ---------- ---------- ---------- ---------- ---------- ---------- ---------- ----------
PlemiC3      ---------- ---------- ---------- ---------- ---------- ---------- ---------- ----------
StcyC5       ---------- ---------- ---------- ---------- ---------- ---------- ---------- ----------
MyGI1A70     ---------- ---------- ---------- ---------- ---------- ---------- ---------- ----------
Ge3709A47    ---------- ---------- ---------- ---------- ---------- ---------- ---------- ----------
GeherA54     ---------- ---------- ---------- ---------- ---------- ---------- ---------- ----------
Cy7425A46    ---------- ---------- ---------- ---------- ---------- ---------- ---------- ----------
PsbicA85     ---------- ---------- ---------- ---------- ---------- ---------- ---------- ----------
PsRCrA88     ---------- ---------- ---------- ---------- ---------- ---------- ---------- ----------
Ps6802A69    ---------- ---------- ---------- ---------- ---------- ---------- ---------- ----------
CygrC2       ---------- ---------- ---------- ---------- ---------- ---------- ---------- ----------
LeboA83      ---------- ---------- ---------- ---------- ---------- ---------- ---------- ----------
LeO77A58     ---------- ---------- ---------- ---------- ---------- ---------- ---------- ----------
ChsiA39      ---------- ---------- ---------- ---------- ---------- ---------- ---------- ----------
Gl7428A40    ---------- ---------- ---------- ---------- ---------- ---------- ---------- ----------
Sy7509C4     ---------- ---------- ---------- ---------- ---------- ---------- ---------- ----------
Mi7113C10    ---------- ---------- ---------- ---------- ---------- ---------- ---------- ----------
AncyC9       ---------- ---------- ---------- ---------- ---------- ---------- ---------- ----------
CystC12      ---------- ---------- ---------- ---------- ---------- ---------- ---------- ----------
An90A65      ---------- ---------- ---------- ---------- ---------- ---------- ---------- ----------
DociA48      ---------- ---------- ---------- ---------- ---------- ---------- ---------- ----------
Ca7507A67    ---------- ---------- ---------- ---------- ---------- ---------- ---------- ----------
No7107C11    ---------- ---------- ---------- ---------- ---------- ---------- ---------- ----------
NopunA49     ---------- ---------- ---------- ---------- ---------- ---------- ---------- ----------
NocaA53      ---------- ---------- ---------- ---------- ---------- ---------- ---------- ----------
No7524A56    ---------- ---------- ---------- ---------- ---------- ---------- ---------- ----------
Ab33047A55   ---------- ---------- ---------- ---------- ---------- ---------- ---------- ----------
HapA62       ---------- ---------- ---------- ---------- ---------- ---------- ---------- ----------
ChfrA66      ---------- ---------- ---------- ---------- ---------- ---------- ---------- ----------
Sc1581A64    ---------- ---------- ---------- ---------- ---------- ---------- ---------- ----------
AphflosA59   ---------- ---------- ---------- ---------- ---------- ---------- ---------- ----------
ChrthA50     ---------- ---------- ---------- ---------- ---------- ---------- ---------- ----------
Os10802A37   ---------- ---------- ---------- ---------- ---------- ---------- ---------- ----------
Pl001A63     ---------- ---------- ---------- ---------- ---------- ---------- ---------- ----------
NodnodA72    ---------- ---------- ---------- ---------- ---------- ---------- ---------- ----------
Ly8106A74    ---------- ---------- ---------- ---------- ---------- ---------- ---------- ----------
MivagA79     ---------- ---------- ---------- ---------- ---------- ---------- ---------- ----------
PlseA87      ---------- ---------- ---------- ---------- ---------- ---------- ---------- ----------
ScHK05A42    ---------- ---------- ---------- ---------- ---------- ---------- ---------- ----------
TocaA43      ---------- ---------- ---------- ---------- ---------- ---------- ---------- ----------
MareA44      ---------- ---------- ---------- ---------- ---------- ---------- ---------- ----------
SchofA45     ---------- ---------- ---------- ---------- ---------- ---------- ---------- ----------
SctoA60      ---------- ---------- ---------- ---------- ---------- ---------- ---------- ----------
NesphA68     ---------- ---------- ---------- ---------- ---------- ---------- ---------- ----------
ChaminC13    ---------- ---------- ---------- ---------- ---------- ---------- ---------- ----------
Sy33AbA26    ---------- ---------- ---------- ---------- ---------- ---------- ---------- ----------
ThelA27      ---------- ---------- ---------- ---------- ---------- ---------- ---------- ----------
TheloA80     ---------- ---------- ---------- ---------- ---------- ---------- ---------- ----------
ThNK55aA89   ---------- ---------- ---------- ---------- ---------- ---------- ---------- ----------
Sy23BaA28    ---------- ---------- ---------- ---------- ---------- ---------- ---------- ----------
GlviA76      ---------- ---------- ---------- ---------- ---------- ---------- ---------- ----------
```

```
                          420                 440                 460                 480
                            |                   |                   |                   |
MiaerNR       ---------- ---------- ---------- ---------- ---------- ---------- ---------- ----------
MiaerBIT      ---------- ---------- ---------- ---------- ---------- ---------- ---------- ----------
PrhoA25       ---------- ---------- ---------- ---------- ---------- ---------- ---------- ----------
Cy1200A71     ---------- ---------- ---------- ---------- ---------- ---------- ---------- ----------
No7120A1      ---------- ---------- ---------- ---------- ---------- ---------- ---------- ----------
No3756A2      ---------- ---------- ---------- ---------- ---------- ---------- ---------- ----------
No7524C1      ---------- ---------- ---------- ---------- ---------- ---------- ---------- ----------
NopiA3        ---------- ---------- ---------- ---------- ---------- ---------- ---------- ----------
An33047A4     ---------- ---------- ---------- ---------- ---------- ---------- ---------- ----------
An43A11       ---------- ---------- ---------- ---------- ---------- ---------- ---------- ----------
CaHK06A7      ---------- ---------- ---------- ---------- ---------- ---------- ---------- ----------
Ca7103A9      ---------- ---------- ---------- ---------- ---------- ---------- ---------- ----------
Ca336A17      ---------- ---------- ---------- ---------- ---------- ---------- ---------- ----------
NoKVJ20A5     ---------- ---------- ---------- ---------- ---------- ---------- ---------- ----------
To7601A6      ---------- ---------- ---------- ---------- ---------- ---------- ---------- ----------
ChfrA10       ---------- ---------- ---------- ---------- ---------- ---------- ---------- ----------
Cy7702A22     ---------- ---------- ---------- ---------- ---------- ---------- ---------- ----------
Fi9339A13     ---------- ---------- ---------- ---------- ---------- ---------- ---------- ----------
MalaA29       ---------- ---------- ---------- ---------- ---------- ---------- ---------- ----------
FiJSC11A14    ---------- ---------- ---------- ---------- ---------- ---------- ---------- ----------
Fi3754A15     ---------- ---------- ---------- ---------- ---------- ---------- ---------- ----------
Fi9605A12     ---------- ---------- ---------- ---------- ---------- ---------- ---------- ----------
FocoA8        ---------- ---------- ---------- ---------- ---------- ---------- ---------- ----------
AbWA102A18    ---------- ---------- ---------- ---------- ---------- ---------- ---------- ----------
AphflosA19    ---------- ---------- ---------- ---------- ---------- ---------- ---------- ----------
TriNMC1A16    ---------- ---------- ---------- ---------- ---------- ---------- ---------- ----------
CyraA24       ---------- ---------- ---------- ---------- ---------- ---------- ---------- ----------
RabrA33       ---------- ---------- ---------- ---------- ---------- ---------- ---------- ----------
TobouA21      ---------- ---------- ---------- ---------- ---------- ---------- ---------- ----------
CapaA38       ---------- ---------- ---------- ---------- ---------- ---------- ---------- ----------
ChaminC7      ---------- ---------- ---------- ---------- ---------- ---------- ---------- ----------
Sy6714A61     ---------- ---------- ---------- ---------- ---------- ---------- ---------- ----------
Ge3709A73     ---------- ---------- ---------- ---------- ---------- ---------- ---------- ----------
AlatA20       ---------- ---------- ---------- ---------- ---------- ---------- ---------- ----------
Sy7509A23     ---------- ---------- ---------- ---------- ---------- ---------- ---------- ----------
CrepC8        ---------- ---------- ---------- ---------- ---------- ---------- ---------- ----------
KampA81       AKTETTTISA SPVPIASPPP PSAMSAAPMS VPSSMSAAPA IAPSSVSAID AAPASATAST NSAPATTDAT SAPRITDKAE
Cy7822A34     ---------- ---------- ---------- ---------- ---------- ---------- ---------- ----------
Cy7424A36     ---------- ---------- ---------- ---------- ---------- ---------- ---------- ----------
Cy0110A35     ---------- ---------- ---------- ---------- ---------- ---------- ---------- ----------
HyriA41       ---------- ---------- ---------- ---------- ---------- ---------- ---------- ----------
PlemiC3       ---------- ---------- ---------- ---------- ---------- ---------- ---------- ----------
StcyC5        ---------- ---------- ---------- ---------- ---------- ---------- ---------- ----------
MyGl1A70      ---------- ---------- ---------- ---------- ---------- ---------- ---------- ----------
Ge3709A47     ---------- ---------- ---------- ---------- ---------- ---------- ---------- ----------
GeherA54      ---------- ---------- ---------- ---------- ---------- ---------- ---------- ----------
Cy7425A46     ---------- ---------- ---------- ---------- ---------- ---------- ---------- ----------
PsbicA85      ---------- ---------- ---------- ---------- ---------- ---------- ---------- ----------
PsRCrA88      ---------- ---------- ---------- ---------- ---------- ---------- ---------- ----------
Ps6802A69     ---------- ---------- ---------- ---------- ---------- ---------- ---------- ----------
CygrC2        ---------- ---------- ---------- ---------- ---------- ---------- ---------- ----------
LeboA83       ---------- ---------- ---------- ---------- ---------- ---------- ---------- ----------
LeO77A58      ---------- ---------- ---------- ---------- ---------- ---------- ---------- ----------
ChsiA39       ---------- ---------- ---------- ---------- ---------- ---------- ---------- ----------
Gl7428A40     ---------- ---------- ---------- ---------- ---------- ---------- ---------- ----------
Sy7509C4      ---------- ---------- ---------- ---------- ---------- ---------- ---------- ----------
Mi7113C10     ---------- ---------- ---------- ---------- ---------- ---------- ---------- ----------
AncyC9        ---------- ---------- ---------- ---------- ---------- ---------- ---------- ----------
CystC12       ---------- ---------- ---------- ---------- ---------- ---------- ---------- ----------
An90A65       ---------- ---------- ---------- ---------- ---------- ---------- ---------- ----------
DociA48       ---------- ---------- ---------- ---------- ---------- ---------- ---------- ----------
Ca7507A67     ---------- ---------- ---------- ---------- ---------- ---------- ---------- ----------
No7107C11     ---------- ---------- ---------- ---------- ---------- ---------- ---------- ----------
NopunA49      ---------- ---------- ---------- ---------- ---------- ---------- ---------- ----------
NocaA53       ---------- ---------- ---------- ---------- ---------- ---------- ---------- ----------
No7524A56     ---------- ---------- ---------- ---------- ---------- ---------- ---------- ----------
Ab33047A55    ---------- ---------- ---------- ---------- ---------- ---------- ---------- ----------
HapA62        ---------- ---------- ---------- ---------- ---------- ---------- ---------- ----------
ChfrA66       ---------- ---------- ---------- ---------- ---------- ---------- ---------- ----------
Sc1581A64     ---------- ---------- ---------- ---------- ---------- ---------- ---------- ----------
AphflosA59    ---------- ---------- ---------- ---------- ---------- ---------- ---------- ----------
ChrthA50      ---------- ---------- ---------- ---------- ---------- ---------- ---------- ----------
Os10802A37    ---------- ---------- ---------- ---------- ---------- ---------- ---------- ----------
Pl001A63      ---------- ---------- ---------- ---------- ---------- ---------- ---------- ----------
NodnodA72     ---------- ---------- ---------- ---------- ---------- ---------- ---------- ----------
Ly8106A74     ---------- ---------- ---------- ---------- ---------- ---------- ---------- ----------
MivagA79      ---------- ---------- ---------- ---------- ---------- ---------- ---------- ----------
PlseA87       ---------- ---------- ---------- ---------- ---------- ---------- ---------- ----------
ScHK05A42     ---------- ---------- ---------- ---------- ---------- ---------- ---------- ----------
TocaA43       ---------- ---------- ---------- ---------- ---------- ---------- ---------- ----------
MareA44       ---------- ---------- ---------- ---------- ---------- ---------- ---------- ----------
SchofA45      ---------- ---------- ---------- ---------- ---------- ---------- ---------- ----------
SctoA60       ---------- ---------- ---------- ---------- ---------- ---------- ---------- ----------
NesphA68      ---------- ---------- ---------- ---------- ---------- ---------- ---------- ----------
ChaminC13     ---------- ---------- ---------- ---------- ---------- ---------- ---------- ----------
Sy33AbA26     ---------- ---------- ---------- ---------- ---------- ---------- ---------- ----------
ThelA27       ---------- ---------- ---------- ---------- ---------- ---------- ---------- ----------
TheloA80      ---------- ---------- ---------- ---------- ---------- ---------- ---------- ----------
ThNK55aA89    ---------- ---------- ---------- ---------- ---------- ---------- ---------- ----------
Sy23BaA28     ---------- ---------- ---------- ---------- ---------- ---------- ---------- ----------
GlviA76       ---------- ---------- ---------- ---------- ---------- ---------- ---------- ----------
```

```
                                                 500        520        540        560
                                                   |          |          |          |
    MiaerNR ---------- ---------- ---------- ---------- ---------- ---------- ---------- ----------
   MiaerBIT ---------- ---------- ---------- ---------- ---------- ---------- ---------- ----------
   PrhoA25 ---------- ---------- ---------- ---------- ---------- ---------- ---------- ----------
  Cy1200A71 ---------- ---------- ---------- ---------- ---------- ---------- ---------- ----------
   No7120A1 ---------- ---------- ---------- ---------- ---------- ---------- ---------- ----------
   No3756A2 ---------- ---------- ---------- ---------- ---------- ---------- ---------- ----------
   No7524C1 ---------- ---------- ---------- ---------- ---------- ---------- ---------- ----------
     NopiA3 ---------- ---------- ---------- ---------- ---------- ---------- ---------- ----------
  An33047A4 ---------- ---------- ---------- ---------- ---------- ---------- ---------- ----------
    An43A11 ---------- ---------- ---------- ---------- ---------- ---------- ---------- ----------
   CaHK06A7 ---------- ---------- ---------- ---------- ---------- ---------- ---------- ----------
   Ca7103A9 ---------- ---------- ---------- ---------- ---------- ---------- ---------- ----------
   Ca336A17 ---------- ---------- ---------- ---------- ---------- ---------- ---------- ----------
 NoKVJ20A5 ---------- ---------- ---------- ---------- ---------- ---------- ---------- ----------
   To7601A6 ---------- ---------- ---------- ---------- ---------- ---------- ---------- ----------
    ChfrA10 ---------- ---------- ---------- ---------- ---------- ---------- ---------- ----------
  Cy7702A22 ---------- ---------- ---------- ---------- ---------- ---------- ---------- ----------
  Fi9339A13 ---------- ---------- ---------- ---------- ---------- ---------- ---------- ----------
    MalaA29 ---------- ---------- ---------- ---------- ---------- ---------- ---------- ----------
FiJSC11A14 ---------- ---------- ---------- ---------- ---------- ---------- ---------- ----------
  Fi3754A15 ---------- ---------- ---------- ---------- ---------- ---------- ---------- ----------
  Fi9605A12 ---------- ---------- ---------- ---------- ---------- ---------- ---------- ----------
    FocoA8 ---------- ---------- ---------- ---------- ---------- ---------- ---------- ----------
AbWA102A18 ---------- ---------- ---------- ---------- ---------- ---------- ---------- ----------
AphflosA19 ---------- ---------- ---------- ---------- ---------- ---------- ---------- ----------
TriNMC1A16 ---------- ---------- ---------- ---------- ---------- ---------- ---------- ----------
    CyraA24 ---------- ---------- ---------- ---------- ---------- ---------- ---------- ----------
    RabrA33 ---------- ---------- ---------- ---------- ---------- ---------- ---------- ----------
   TobouA21 ---------- ---------- ---------- ---------- ---------- ---------- ---------- ----------
    CapaA38 ---------- ---------- ---------- ---------- ---------- ---------- ---------- ----------
   ChaminC7 ---------- ---------- ---------- ---------- ---------- ---------- ---------- ----------
  Sy6714A61 ---------- ---------- ---------- ---------- ---------- ---------- ---------- ----------
  Ge3709A73 ---------- ---------- ---------- ---------- ---------- ---------- ---------- ----------
    AlatA20 ---------- ---------- ---------- ---------- ---------- ---------- ---------- ----------
  Sy7509A23 ---------- ---------- ---------- ---------- ---------- ---------- ---------- ----------
    CrepC8 ---------- ---------- ---------- ---------- ---------- ---------- ---------- ----------
   KampA81 IDALKQKLYT QIDTTWKTTP TFTEDLVYKV KVKQDGTILG YEATNQVAKN FVQETPLASI QNSASSASAT AEKTAEFRVT
  Cy7822A34 ---------- ---------- ---------- ---------- ---------- ---------- ---------- ----------
  Cy7424A36 ---------- ---------- ---------- ---------- ---------- ---------- ---------- ----------
  Cy0110A35 ---------- ---------- ---------- ---------- ---------- ---------- ---------- ----------
    HyriA41 ---------- ---------- ---------- ---------- ---------- ---------- ---------- ----------
    PlemiC3 ---------- ---------- ---------- ---------- ---------- ---------- ---------- ----------
    StcyC5 ---------- ---------- ---------- ---------- ---------- ---------- ---------- ----------
  MyGl1A70 ---------- ---------- ---------- ---------- ---------- ---------- ---------- ----------
  Ge3709A47 ---------- ---------- ---------- ---------- ---------- ---------- ---------- ----------
  GeherA54 ---------- ---------- ---------- ---------- ---------- ---------- ---------- ----------
  Cy7425A46 ---------- ---------- ---------- ---------- ---------- ---------- ---------- ----------
   PsbicA85 ---------- ---------- ---------- ---------- ---------- ---------- ---------- ----------
  PsRCrA88 ---------- ---------- ---------- ---------- ---------- ---------- ---------- ----------
  Ps6802A69 ---------- ---------- ---------- ---------- ---------- ---------- ---------- ----------
    CygrC2 ---------- ---------- ---------- ---------- ---------- ---------- ---------- ----------
    LeboA83 ---------- ---------- ---------- ---------- ---------- ---------- ---------- ----------
  LeO77A58 ---------- ---------- ---------- ---------- ---------- ---------- ---------- ----------
    ChsiA39 ---------- ---------- ---------- ---------- ---------- ---------- ---------- ----------
  Gl7428A40 ---------- ---------- ---------- ---------- ---------- ---------- ---------- ----------
   Sy7509C4 ---------- ---------- ---------- ---------- ---------- ---------- ---------- ----------
  Mi7113C10 ---------- ---------- ---------- ---------- ---------- ---------- ---------- ----------
   AncyC9 ---------- ---------- ---------- ---------- ---------- ---------- ---------- ----------
   CystC12 ---------- ---------- ---------- ---------- ---------- ---------- ---------- ----------
   An90A65 ---------- ---------- ---------- ---------- ---------- ---------- ---------- ----------
   DociA48 ---------- ---------- ---------- ---------- ---------- ---------- ---------- ----------
  Ca7507A67 ---------- ---------- ---------- ---------- ---------- ---------- ---------- ----------
  No7107C11 ---------- ---------- ---------- ---------- ---------- ---------- ---------- ----------
  NopunA49 ---------- ---------- ---------- ---------- ---------- ---------- ---------- ----------
   NocaA53 ---------- ---------- ---------- ---------- ---------- ---------- ---------- ----------
  No7524A56 ---------- ---------- ---------- ---------- ---------- ---------- ---------- ----------
Ab33047A55 ---------- ---------- ---------- ---------- ---------- ---------- ---------- ----------
    HapA62 ---------- ---------- ---------- ---------- ---------- ---------- ---------- ----------
   ChfrA66 ---------- ---------- ---------- ---------- ---------- ---------- ---------- ----------
  Sc1581A64 ---------- ---------- ---------- ---------- ---------- ---------- ---------- ----------
AphflosA59 ---------- ---------- ---------- ---------- ---------- ---------- ---------- ----------
  ChrthA50 ---------- ---------- ---------- ---------- ---------- ---------- ---------- ----------
Os10802A37 ---------- ---------- ---------- ---------- ---------- ---------- ---------- ----------
   Pl001A63 ---------- ---------- ---------- ---------- ---------- ---------- ---------- ----------
NodnodA72 ---------- ---------- ---------- ---------- ---------- ---------- ---------- ----------
  Ly8106A74 ---------- ---------- ---------- ---------- ---------- ---------- ---------- ----------
  MivagA79 ---------- ---------- ---------- ---------- ---------- ---------- ---------- ----------
   PlseA87 ---------- ---------- ---------- ---------- ---------- ---------- ---------- ----------
 ScHK05A42 ---------- ---------- ---------- ---------- ---------- ---------- ---------- ----------
   TocaA43 ---------- ---------- ---------- ---------- ---------- ---------- ---------- ----------
   MareA44 ---------- ---------- ---------- ---------- ---------- ---------- ---------- ----------
  SchofA45 ---------- ---------- ---------- ---------- ---------- ---------- ---------- ----------
   SctoA60 ---------- ---------- ---------- ---------- ---------- ---------- ---------- ----------
 NesphA68 ---------- ---------- ---------- ---------- ---------- ---------- ---------- ----------
 ChaminC13 ---------- ---------- ---------- ---------- ---------- ---------- ---------- ----------
Sy33AbA26 ---------- ---------- ---------- ---------- ---------- ---------- ---------- ----------
   ThelA27 ---------- ---------- ---------- ---------- ---------- ---------- ---------- ----------
   TheloA80 ---------- ---------- ---------- ---------- ---------- ---------- ---------- ----------
ThNK55aA89 ---------- ---------- ---------- ---------- ---------- ---------- ---------- ----------
 Sy23BaA28 ---------- ---------- ---------- ---------- ---------- ---------- ---------- ----------
   GlviA76 ---------- ---------- ---------- ---------- ---------- ---------- ---------- ----------
```

	580 600 620 640
MiaerNR	---------- ---------- ---------- ---------- ---------- ---------- ---------- ----------
MiaerBIT	---------- ---------- ---------- ---------- ---------- ---------- ---------- ----------
PrhoA25	---------- ---------- ---------- ---------- ---------- ---------- ---------- ----------
Cy1200A71	---------- ---------- ---------- ---------- ---------- ---------- ---------- ----------
No7120A1	---------- ---------- ---------- ---------- ---------- ---------- ---------- ----------
No3756A2	---------- ---------- ---------- ---------- ---------- ---------- ---------- ----------
No7524C1	---------- ---------- ---------- ---------- ---------- ---------- ---------- ----------
NopiA3	---------- ---------- ---------- ---------- ---------- ---------- ---------- ----------
An33047A4	---------- ---------- ---------- ---------- ---------- ---------- ---------- ----------
An43A11	---------- ---------- ---------- ---------- ---------- ---------- ---------- ----------
CaHK06A7	---------- ---------- ---------- ---------- ---------- ---------- ---------- ----------
Ca7103A9	---------- ---------- ---------- ---------- ---------- ---------- ---------- ----------
Ca336A17	---------- ---------- ---------- ---------- ---------- ---------- ---------- ----------
NoKVJ20A5	---------- ---------- ---------- ---------- ---------- ---------- ---------- ----------
To7601A6	---------- ---------- ---------- ---------- ---------- ---------- ---------- ----------
ChfrA10	---------- ---------- ---------- ---------- ---------- ---------- ---------- ----------
Cy7702A22	---------- ---------- ---------- ---------- ---------- ---------- ---------- ----------
Fi9339A13	---------- ---------- ---------- ---------- ---------- ---------- ---------- ----------
MalaA29	---------- ---------- ---------- ---------- ---------- ---------- ---------- ----------
FiJSC11A14	---------- ---------- ---------- ---------- ---------- ---------- ---------- ----------
Fi3754A15	---------- ---------- ---------- ---------- ---------- ---------- ---------- ----------
Fi9605A12	---------- ---------- ---------- ---------- ---------- ---------- ---------- ----------
FocoA8	---------- ---------- ---------- ---------- ---------- ---------- ---------- ----------
AbWA102A18	---------- ---------- ---------- ---------- ---------- ---------- ---------- ----------
AphflosA19	---------- ---------- ---------- ---------- ---------- ---------- ---------- ----------
TriNMC1A16	---------- ---------- ---------- ---------- ---------- ---------- ---------- ----------
CyraA24	---------- ---------- ---------- ---------- ---------- ---------- ---------- ----------
RabrA33	---------- ---------- ---------- ---------- ---------- ---------- ---------- ----------
TobouA21	---------- ---------- ---------- ---------- ---------- ---------- ---------- ----------
CapaA38	---------- ---------- ---------- ---------- ---------- ---------- ---------- ----------
ChaminC7	---------- ---------- ---------- ---------- ---------- ---------- ---------- ----------
Sy6714A61	---------- ---------- ---------- ---------- ---------- ---------- ---------- ----------
Ge3709A73	---------- ---------- ---------- ---------- ---------- ---------- ---------- ----------
AlatA20	---------- ---------- ---------- ---------- ---------- ---------- ---------- ----------
Sy7509A23	---------- ---------- ---------- ---------- ---------- ---------- ---------- ----------
CrepC8	---------- ---------- ---------- ---------- ---------- ---------- ---------- ----------
KampA81	FSTANGGALD VKPWE----- ---------- ---------- ---------- ---------- ---------- ----------
Cy7822A34	---------- ---------- ---------- ---------- ---------- ---------- ---------- ----------
Cy7424A36	---------- ---------- ---------- ---------- ---------- ---------- ---------- ----------
Cy0110A35	---------- ---------- ---------- ---------- ---------- ---------- ---------- ----------
HyriA41	---------- ---------- ---------- ---------- ---------- ---------- ---------- ----------
PlemiC3	---------- ---------- ---------- ---------- ---------- ---------- ---------- ----------
StcyC5	---------- ---------- ---------- ---------- ---------- ---------- ---------- ----------
MyGI1A70	---------- ---------- ---------- ---------- ---------- ---------- ---------- ----------
Ge3709A47	---------- ---------- ---------- ---------- ---------- ---------- ---------- ----------
GeherA54	---------- ---------- ---------- ---------- ---------- ---------- ---------- ----------
Cy7425A46	---------- ---------- ---------- ---------- ---------- ---------- ---------- ----------
PsbicA85	---------- ---------- ---------- ---------- ---------- ---------- ---------- ----------
PsRCrA88	---------- ---------- ---------- ---------- ---------- ---------- ---------- ----------
Ps6802A69	---------- ---------- ---------- ---------- ---------- ---------- ---------- ----------
CygrC2	---------- ---------- ---------- ---------- ---------- ---------- ---------- ----------
LeboA83	---------- ---------- ---------- ---------- ---------- ---------- ---------- ----------
LeO77A58	---------- ---------- ---------- ---------- ---------- ---------- ---------- ----------
ChsiA39	---------- ---------- ---------- ---------- ---------- ---------- ---------- ----------
Gl7428A40	---------- ---------- ---------- ---------- ---------- ---------- ---------- ----------
Sy7509C4	---------- ---------- ---------- ---------- ---------- ---------- ---------- ----------
Mi7113C10	---------- ---------- ---------- ---------- ---------- ---------- ---------- ----------
AncyC9	---------- ---------- ---------- ---------- ---------- ---------- ---------- ----------
CystC12	---------- ---------- ---------- ---------- ---------- ---------- ---------- ----------
An90A65	---------- ---------- ---------- ---------- ---------- ---------- ---------- ----------
DociA48	---------- ---------- ---------- ---------- ---------- ---------- ---------- ----------
Ca7507A67	---------- ---------- ---------- ---------- ---------- ---------- ---------- ----------
No7107C11	---------- ---------- ---------- ---------- ---------- ---------- ---------- ----------
NopunA49	---------- ---------- ---------- ---------- ---------- ---------- ---------- ----------
NocaA53	---------- ---------- ---------- ---------- ---------- ---------- ---------- ----------
No7524A56	---------- ---------- ---------- ---------- ---------- ---------- ---------- ----------
Ab33047A55	---------- ---------- ---------- ---------- ---------- ---------- ---------- ----------
HapA62	---------- ---------- ---------- ---------- ---------- ---------- ---------- ----------
ChfrA66	---------- ---------- ---------- ---------- ---------- ---------- ---------- ----------
Sc1581A64	---------- ---------- ---------- ---------- ---------- ---------- ---------- ----------
AphflosA59	---------- ---------- ---------- ---------- ---------- ---------- ---------- ----------
ChrthA50	---------- ---------- ---------- ---------- ---------- ---------- ---------- ----------
Os10802A37	---------- ---------- ---------- ---------- ---------- ---------- ---------- ----------
Pl001A63	---------- ---------- ---------- ---------- ---------- ---------- ---------- ----------
NodnodA72	---------- ---------- ---------- ---------- ---------- ---------- ---------- ----------
Ly8106A74	---------- ---------- ---------- ---------- ---------- ---------- ---------- ----------
MivagA79	---------- ---------- ---------- ---------- ---------- ---------- ---------- ----------
PlseA87	---------- ---------- ---------- ---------- ---------- ---------- ---------- ----------
ScHK05A42	---------- ---------- ---------- ---------- ---------- ---------- ---------- ----------
TocaA43	---------- ---------- ---------- ---------- ---------- ---------- ---------- ----------
MareA44	---------- ---------- ---------- ---------- ---------- ---------- ---------- ----------
SchofA45	---------- ---------- ---------- ---------- ---------- ---------- ---------- ----------
SctoA60	---------- ---------- ---------- ---------- ---------- ---------- ---------- ----------
NesphA68	---------- ---------- ---------- ---------- ---------- ---------- ---------- ----------
ChaminC13	---------- ---------- ---------- ---------- ---------- ---------- ---------- ----------
Sy33AbA26	---------- ---------- ---------- ---------- ---------- ---------- ---------- ----------
ThelA27	---------- ---------- ---------- ---------- ---------- ---------- ---------- ----------
TheloA80	---------- ---------- ---------- ---------- ---------- ---------- ---------- ----------
ThNK55aA89	---------- ---------- ---------- ---------- ---------- ---------- ---------- ----------
Sy23BaA28	---------- ---------- ---------- ---------- ---------- ---------- ---------- ----------
GlviA76	---------- ---------- ---------- ---------- ---------- ---------- ---------- ----------

MiaerNR	- - - - - - - - -	319
MiaerBIT	- - - - - - - - -	319
PrhoA25	- - - - - - - - -	264
Cy1200A71	- - - - - - - - -	318
No7120A1	- - - - - - - - -	264
No3756A2	- - - - - - - - -	263
No7524C1	- - - - - - - - -	264
NopiA3	- - - - - - - - -	263
An33047A4	- - - - - - - - -	264
An43A11	- - - - - - - - -	264
CaHK06A7	- - - - - - - - -	265
Ca7103A9	- - - - - - - - -	265
Ca336A17	- - - - - - - - -	265
NoKVJ20A5	- - - - - - - - -	265
To7601A6	- - - - - - - - -	265
ChfrA10	- - - - - - - - -	265
Cy7702A22	- - - - - - - - -	265
Fi9339A13	- - - - - - - - -	265
MalaA29	- - - - - - - - -	212
FiJSC11A14	- - - - - - - - -	265
Fi3754A15	- - - - - - - - -	265
Fi9605A12	- - - - - - - - -	265
FocoA8	- - - - - - - - -	268
AbWA102A18	- - - - - - - - -	265
AphflosA19	- - - - - - - - -	265
TriNMC1A16	- - - - - - - - -	266
CyraA24	- - - - - - - - -	265
RabrA33	- - - - - - - - -	265
TobouA21	- - - - - - - - -	267
CapaA38	- - - - - - - - -	272
ChaminC7	- - - - - - - - -	261
Sy6714A61	- - - - - - - - -	321
Ge3709A73	- - - - - - - - -	320
AlatA20	- - - - - - - - -	266
Sy7509A23	- - - - - - - - -	265
CrepC8	- - - - - - - - -	264
KampA81	- - - - - - - - -	550
Cy7822A34	- - - - - - - - -	262
Cy7424A36	- - - - - - - - -	262
Cy0110A35	- - - - - - - - -	264
HyriA41	- - - - - - - - -	263
PlemiC3	- - - - - - - - -	263
StcyC5	- - - - - - - - -	263
MyGl1A70	- - - - - - - - -	264
Ge3709A47	- - - - - - - - -	263
GeherA54	- - - - - - - - -	262
Cy7425A46	- - - - - - - - -	262
PsbicA85	- - - - - - - - -	259
PsRCrA88	- - - - - - - - -	259
Ps6802A69	- - - - - - - - -	265
CygrC2	- - - - - - - - -	262
LeboA83	- - - - - - - - -	265
LeO77A58	- - - - - - - - -	262
ChsiA39	- - - - - - - - -	261
Gl7428A40	- - - - - - - - -	261
Sy7509C4	- - - - - - - - -	264
Mi7113C10	- - - - - - - - -	261
AncyC9	- - - - - - - - -	264
CystC12	- - - - - - - - -	264
An90A65	- - - - - - - - -	264
DociA48	- - - - - - - - -	264
Ca7507A67	- - - - - - - - -	264
No7107C11	- - - - - - - - -	264
NopunA49	- - - - - - - - -	264
NocaA53	- - - - - - - - -	264
No7524A56	- - - - - - - - -	263
Ab33047A55	- - - - - - - - -	264
HapA62	- - - - - - - - -	264
ChfrA66	- - - - - - - - -	264
Sc1581A64	- - - - - - - - -	265
AphflosA59	- - - - - - - - -	264
ChrthA50	- - - - - - - - -	264
Os10802A37	- - - - - - - - -	263
Pl001A63	- - - - - - - - -	265
NodnodA72	- - - - - - - - -	261
Ly8106A74	- - - - - - - - -	264
MivagA79	- - - - - - - - -	260
PlseA87	- - - - - - - - -	262
ScHK05A42	- - - - - - - - -	263
TocaA43	- - - - - - - - -	263
MareA44	- - - - - - - - -	264
SchofA45	- - - - - - - - -	264
SctoA60	- - - - - - - - -	264
NesphA68	- - - - - - - - -	263
ChaminC13	- - - - - - - - -	262
Sy33AbA26	- - - - - - - - -	265
ThelA27	- - - - - - - - -	257
TheloA80	- - - - - - - - -	263
ThNK55aA89	- - - - - - - - -	263
Sy23BaA28	- - - - - - - - -	280
GlviA76	- - - - - - - - -	262

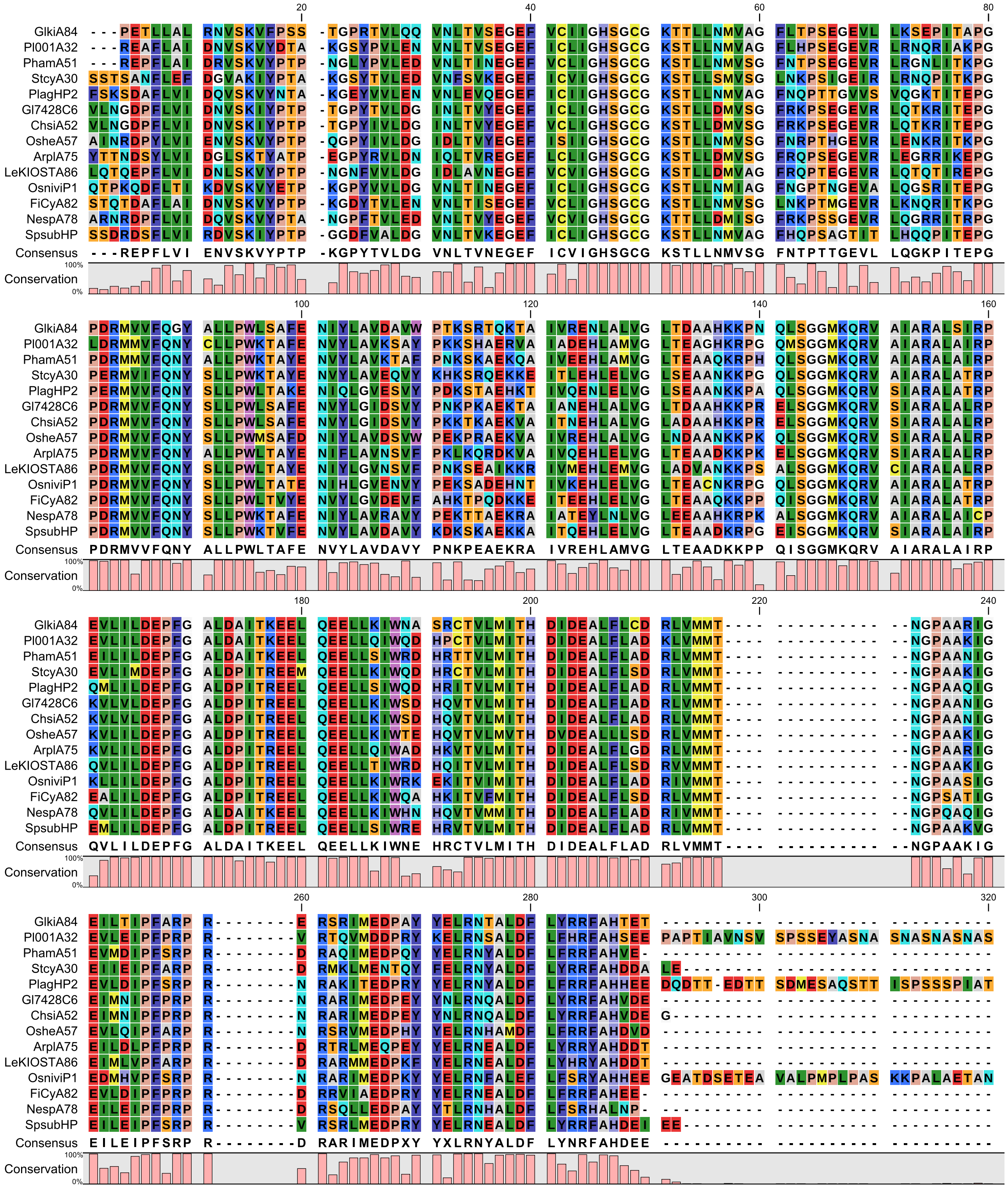
20 40 60 80
GlkiA84 ---PETLLAL RNVSKVFPSS -TGPRTVLQQ VNLTVSEGEF VCIIGHSGCG KTTLLNMVAG FLTPSEGEVL LKSEPITAPG
PI001A32 ---REAFLAI DNVSKVYDTA -KGSYPVLEN VNLTVSEGEF ICLIGHSGCG KSTLLNMVAG FLHPSEGEVR LRNQRIAKPG
PhamA51 ---REPFLAI DRVSKVYPTP -NGLYPVLED VNLTINEGEF VCLIGHSGCG KSTLLNMVSG FNTPSEGEVR LRGNLITKPG
StcyA30 SSTSANFLEF DGVAKIYPTA -KGSYTVLED VNFSVKEGEF ICVIGHSGCG KSTLLSMVSG LNKPSIGEIR LRNQPITKPG
PlagHP2 FSKSDAFLVI DQVSKVYNTA -KGEYVVLEN VNLEVQEGEF ICIIGHSGCG KSTLLNMVAG FNQPTTGVVS VQGKTITEPG
GI7428C6 VLNGDPFLVI DNVSKIYPTP -TGPYTVLDG INLTVYEGEF ICLIGHSGCG KSTLLDMVSG FRKPSEGEVR LQTKRITEPG
ChsiA52 VLNGDPFLVI DNVSKIYPTP -TGPYIVLDG INLTVYEGEF VCLIGHSGCG KSTLLDMVSG FRKPSEGEVR LQTKRITEPG
OsheA57 AINRDPYLVI ENVSKVYPTP -QGPYIVLDG IDLTVYEGEF ISIIGHSGCG KSTLLNMVSG FNRPTHGEVR LENKRITKPG
ArplA75 YTTNDSYLVI DGLSKTYATP -EGPYRVLDN IQLTVREGEF LCLIGHSGCG KSTLLDMVSG FRQPSEGEVR LEGRRIKEPG
LeKIOSTA86 LQTQEPFLVI DNLSKVYPTP -NGNFVVLDG IDLAVNEGEF VCVIGHSGCG KSTLLDMVAG FRQPTEGEVR LQTQTIREPG
OsniviP1 QTPKQDFLTI KDVSKVYETP -KGPYVVLDG VNLTINEGEF ICLIGHSGCG KSTLLNMIAG FNGPTNGEVA LQGSRITEPG
FiCyA82 STQTDAFLAI DNVSKVYPTP -KGDYTVLEN VNLTISEGEF VCLIGHSGCG KSTLLNMVSG LNKPTMGEVR LKNQRITKPG
NespA78 ARNRDPFLVI DQVSKVYPTA -NGPFTVLED VNLTVYEGEF VCVIGHSGCG KTTLLDMISG FRKPSSGEVR LQGRRITRPG
SpsubHP SSDRDSFLVI RDVSKIYPTP -GGDFVALDG VNLTVKEGEF ICLIGHSGCG KSTLLNMVAG FHQPSAGTIT LHQQPITEPG
Consensus ---REPFLVI ENVSKVYPTP -KGPYTVLDG VNLTVNEGEF ICVIGHSGCG KSTLLNMVSG FNTPTTGEVL LQGKPITEPG
Conservation 100% 0%
100 120 140 160
GlkiA84 PDRMVVFQGY ALLPWLSAFE NIYLAVDAVW PTKSRTQKTA IVRENLALVG LTDAAHKKPN QLSGGMKQRV AIARALSIRP
PI001A32 LDRMMVFQNY CLLPWKTAFE NVYLAVKSAY PKKSHAERVA IADEHLAMVG LTEAGHKRPG QMSGGMKQRV AIARALAIRP
PhamA51 PDRMMVFQNY ALLPWKTAYE NVYLAVKTAF PNKSKAEKQA IVEEHLAMVG LTEAAQKRPH QLSGGMKQRV AIARALAIRP
StcyA30 PERMVIFQNY SLLPWKTAYE NVYLAVEQVY KHKSRQEKKE ITLEHLELVG LSEAANKKPG QLSGGMKQRV AIARALATRP
PlagHP2 PERMVVFQNY SLLPWLTAKE NIQLGVESVY PDKSTAEHKT IVQENLELVG LSEAANKKPA QLSGGMKQRV SIARALATRP
GI7428C6 PDRMVVFQNY SLLPWLSAFE NVYLGIDSVY PNKPKAEKTA IANEHLALVG LTDAAHKKPR ELSGGMKQRV SIARALALRP
ChsiA52 PDRMVVFQNY SLLPWLSAFE NVYLGIDSVY PKKTKAEKVA ITNEHLALVG LADAAHKKPR ELSGGMKQRV SIARALALRP
OsheA57 PDRMVVFQNY SLLPWMSAFD NIYLAVDSVW PEKPRAEKVA IVREHLALVG LNDAANKKPK QLSGGMKQRV SIARALALRP
ArplA75 PDRMVVFQNY ALLPWLTAYE NIFLAVNSVF PKLKQRDKVA IVQEHLELVG LTEAADKKPK ELSGGMKQRV SIARALALRP
LeKIOSTA86 PDRMVVFQNY SLLPWLTAYE NIYLGVNSVF PNKSEAIKKR IVMEHLEMVG LADVANKKPS ALSGGMKQRV CIARALALRP
OsniviP1 PDRMVVFQNY SLLPWLTATE NIHLGVENVY PEKSADEHNT IVKEHLELVG LTEACNKRPG QLSGGMKQRV AIARALATRP
FiCyA82 PDRMVVFQNY SLLPWLTVYE NVYLGVDEVF AHKTPQDKKE ITEEHLELVG LTEAAQKKPP QISGGMKQRV AIARALATRP
NespA78 PDRMVVFQNY SLLPWKTAFE NIYLAVRAVY PEKTTAEKRA IATEYLNLVG LEEAAHKRPK ALSGGMKQRV AIARALAICP
SpsubHP PDRMVVFQNY SLLPWKTVFE NVYLAVDAVY KDKSKAEKKA ITQEHLELVG LTEAADKRPG EISGGMKQRV SIARALAIRP
Consensus PDRMVVFQNY ALLPWLTAFE NVYLAVDAVY PNKPEAEKRA IVREHLAMVG LTEAADKKPP QISGGMKQRV AIARALAIRP
Conservation 100% 0%
180 200 220 240
GlkiA84 EVLILDEPFG ALDAITKEEL QEELLKIWNA SRCTVLMITH DIDEALFLCD RLVMMT---- ---------- --NGPAARIG
PI001A32 EVLILDEPFG ALDAITKEEL QEELLQIWQD HPCTVLMITH DIDEALFLAD KLVMMT---- ---------- --NGPAAKIG
PhamA51 EILILDEPFG ALDAITKEEL QEELLSIWRD HRTTVLMITH DIDEALFLSD RLVMMT---- ---------- --NGPAANIG
StcyA30 EVLIMDEPFG ALDPITREEM QEELLSIWQD HRCTVLMITH DIDEALFLSD RLVMMT---- ---------- --NGPAAKIG
PlagHP2 QMLILDEPFG ALDPITREEL QEELLSIWQD HRITVLMITH DIDEALFLAD RLVMMT---- ---------- --NGPAAQIG
GI7428C6 KVLVLDEPFG ALDPITREEL QEELLKIWSD HQVTVLMITH DIDEALFLAD RLVMMT---- ---------- --NGPAANIG
ChsiA52 KVLVLDEPFG ALDPITREEL QEELLKIWSD HQVTVLMITH DIDEALFLAD RLVMMT---- ---------- --NGPAANIG
OsheA57 KVLILDEPFG ALDPITREEL QEELLKIWTE HQVTVLMVTH DVDEALLLSD RLVMMT---- ---------- --NGPAAKIG
ArplA75 KVLILDEPFG ALDPITREEL QEELLQIWAD HKVTVLMITH DIDEALFLGD RLVMMT---- ---------- --NGPAARIG
LeKIOSTA86 QVLILDEPFG ALDPITREEL QEELLTIWRD HQITVLMITH DIDEALFLSD RVVMMT---- ---------- --NGPAAKIG
OsniviP1 KLLILDEPFG ALDPITREEL QEELLKIWRK EKITVIMITH DIDEALFLAD RIVMMT---- ---------- --NGPAASIG
FiCyA82 EALILDEPFG ALDPITREEL QEELLKIWQA HKITVFMITH DIDEALFLSD RLVMMT---- ---------- --NGPSATIG
NespA78 QVLILDEPFG ALDAITKEEL QEELLKIWHN HQVTVMMITH DIDEALFLAD RIVMMT---- ---------- --NGPQAQIG
SpsubHP EMLILDEPFG ALDPITREEL QEELLSIWRE HRVTVLMITH DIDEALFLAD RLVMMT---- ---------- --NGPAAKVG
Consensus QVLILDEPFG ALDAITKEEL QEELLKIWNE HRCTVLMITH DIDEALFLAD RLVMMT---- ---------- --NGPAAKIG
Conservation 100% 0%
260 280 300 320
GlkiA84 EILTIPFARP R--------E RSRIMEDPAY YELRNTALDF LYRRFAHTET ---------- ---------- ----------
PI001A32 EVLEIPFPRP R--------V RTQVMDDPRY KELRNSALDF LFHRFAHSEE PAPTIAVNSV SPSSEYASNA SNASNASNAS
PhamA51 EVMDIPFSRP R--------D RAQIMEDPQY YELRNYALDF LFRRFAHVE- ---------- ---------- ----------
StcyA30 EIIEIPFARP R--------D RMKLMENTQY FELRNYALDF LYRRFAHDDA LE-------- ---------- ----------
PlagHP2 EVLDIPFSRP R--------N RAKITEDPRY YELRNYALDF LFRRFAHHEE DQDTT-EDTT SDMESAQSTT ISPSSSPIAT
GI7428C6 EIMNIPFPRP R--------N RARIMEDPEY YNLRNQALDF LYRRFAHVDE G--------- ---------- ----------
ChsiA52 EIMNIPFPRP R--------N RARIMEDPEY YNLRNQALDF LYRRFAHVDE G--------- ---------- ----------
OsheA57 EVLQIPFARP R--------N RSRVMEDPHY YELRNHAMDF LFRRFAHDVD ---------- ---------- ----------
ArplA75 EILDLPFPRP R--------D RTRLMEQPEY YELRNEALDF LYRRYAHDDT ---------- ---------- ----------
LeKIOSTA86 EIMLVPFARP R--------D RARMMEDPKF YELRNEALDF LYHRYAHDDT ---------- ---------- ----------
OsniviP1 EDMHVPFSRP R--------N RARIMEDPKY YELRNFALEF LFSRYAHHEE GEATDSETEA VALPMPLPAS KKPALAETAN
FiCyA82 EVLDIPFPRP R--------D RRVIAEDPRY YELRNEALDF LFRRFAHEE- ---------- ---------- ----------
NespA78 EILEIPFPRP R--------D RSQLLEDPAY YTLRNHALDF LFSRHALNP- ---------- ---------- ----------
SpsubHP EILEIPFSRP R--------V RSRLMEDPRY YELRNEALDF LYNRFAHDEI EE-------- ---------- ----------
Consensus EILEIPFSRP R--------D RARIMEDPXY YXLRNYALDF LYNRFAHDEE ---------- ---------- ----------
Conservation 100% 0%

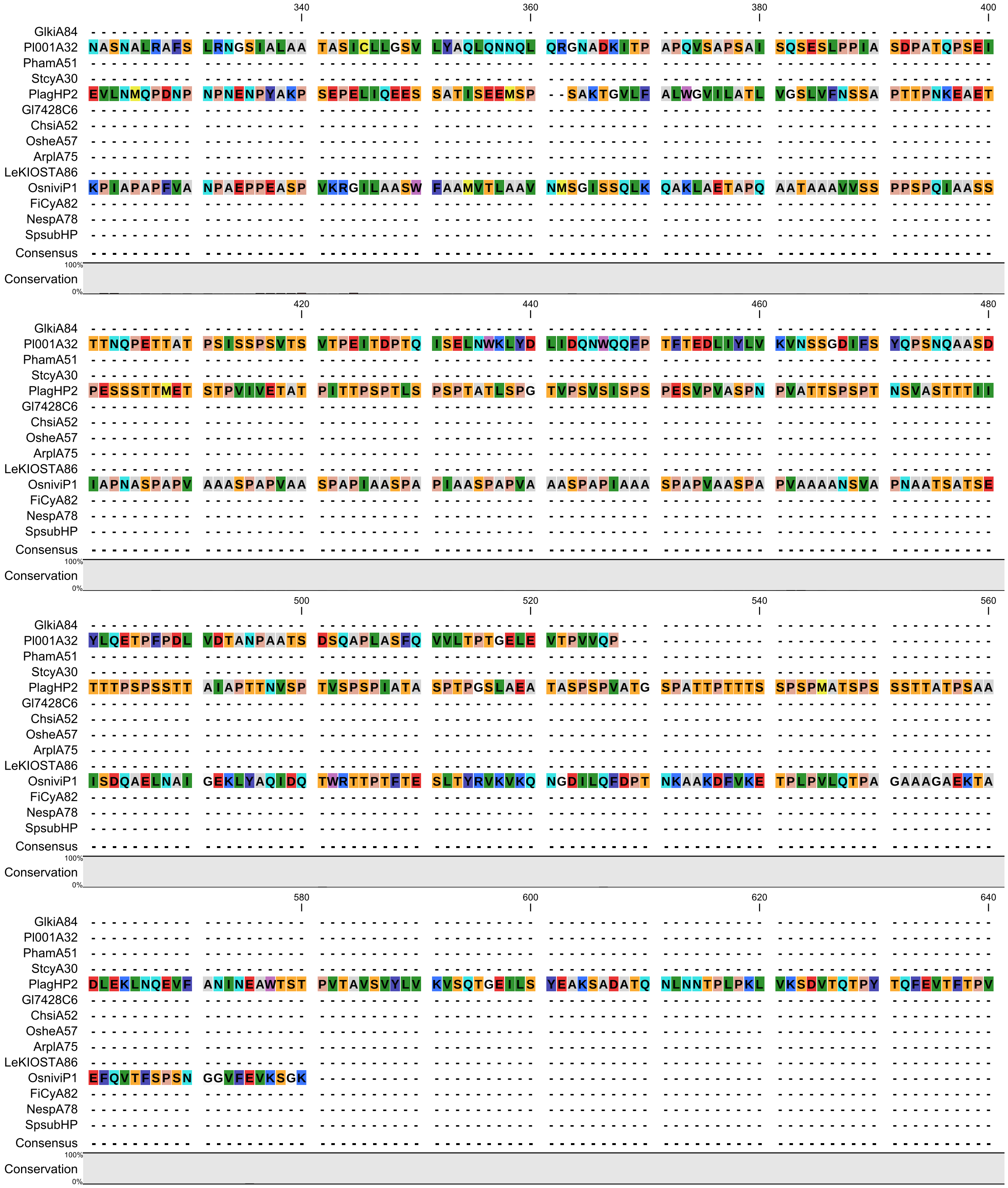
340 360 380 400
GlkiA84 ---------- ---------- ---------- ---------- ---------- ---------- ---------- ----------
Pl001A32 NASNALRAFS LRNGSIALAA TASICLLGSV LYAQLQNNQL QRGNADKITP APQVSAPSAI SQSESLPPIA SDPATQPSEI
PhamA51 ---------- ---------- ---------- ---------- ---------- ---------- ---------- ----------
StcyA30 ---------- ---------- ---------- ---------- ---------- ---------- ---------- ----------
PlagHP2 EVLNMQPDNP NPNENPYAKP SEPELIQEES SATISEEMSP --SAKTGVLF ALWGVILATL VGSLVFNSSA PTTPNKEAET
Gl7428C6 ---------- ---------- ---------- ---------- ---------- ---------- ---------- ----------
ChsiA52 ---------- ---------- ---------- ---------- ---------- ---------- ---------- ----------
OsheA57 ---------- ---------- ---------- ---------- ---------- ---------- ---------- ----------
ArplA75 ---------- ---------- ---------- ---------- ---------- ---------- ---------- ----------
LeKIOSTA86 ---------- ---------- ---------- ---------- ---------- ---------- ---------- ----------
OsniviP1 KPIAPAPFVA NPAEPPEASP VKRGILAASW FAAMVTLAAV NMSGISSQLK QAKLAETAPQ AATAAAVVSS PPSPQIAASS
FiCyA82 ---------- ---------- ---------- ---------- ---------- ---------- ---------- ----------
NespA78 ---------- ---------- ---------- ---------- ---------- ---------- ---------- ----------
SpsubHP ---------- ---------- ---------- ---------- ---------- ---------- ---------- ----------
Consensus ---------- ---------- ---------- ---------- ---------- ---------- ---------- ----------
100%
Conservation
0%
420 440 460 480
GlkiA84 ---------- ---------- ---------- ---------- ---------- ---------- ---------- ----------
Pl001A32 TTNQPETTAT PSISSPSVTS VTPEITDPTQ ISELNWKLYD LIDQNWQQFP TFTEDLIYLV KVNSSGDIFS YQPSNQAASD
PhamA51 ---------- ---------- ---------- ---------- ---------- ---------- ---------- ----------
StcyA30 ---------- ---------- ---------- ---------- ---------- ---------- ---------- ----------
PlagHP2 PESSSTTMET STPVIVETAT PITTPSPTLS PSPTATLSPG TVPSVSISPS PESVPVASPN PVATTSPSPT NSVASTTTII
Gl7428C6 ---------- ---------- ---------- ---------- ---------- ---------- ---------- ----------
ChsiA52 ---------- ---------- ---------- ---------- ---------- ---------- ---------- ----------
OsheA57 ---------- ---------- ---------- ---------- ---------- ---------- ---------- ----------
ArplA75 ---------- ---------- ---------- ---------- ---------- ---------- ---------- ----------
LeKIOSTA86 ---------- ---------- ---------- ---------- ---------- ---------- ---------- ----------
OsniviP1 IAPNASPAPV AAASPAPVAA SPAPIAASPA PIAASPAPVA AASPAPIAAA SPAPVAASPA PVAAAANSVA PNAATSATSE
FiCyA82 ---------- ---------- ---------- ---------- ---------- ---------- ---------- ----------
NespA78 ---------- ---------- ---------- ---------- ---------- ---------- ---------- ----------
SpsubHP ---------- ---------- ---------- ---------- ---------- ---------- ---------- ----------
Consensus ---------- ---------- ---------- ---------- ---------- ---------- ---------- ----------
100%
Conservation
0%
500 520 540 560
GlkiA84 ---------- ---------- ---------- ---------- ---------- ---------- ---------- ----------
Pl001A32 YLQETPFPDL VDTANPAATS DSQAPLASFQ VVLTPTGELE VTPVVQP--- ---------- ---------- ----------
PhamA51 ---------- ---------- ---------- ---------- ---------- ---------- ---------- ----------
StcyA30 ---------- ---------- ---------- ---------- ---------- ---------- ---------- ----------
PlagHP2 TTTPSPSSTT AIAPTTNVSP TVSPSPIATA SPTPGSLAEA TASPSPVATG SPATTPTTTS SPSPMATSPS SSTTATPSAA
Gl7428C6 ---------- ---------- ---------- ---------- ---------- ---------- ---------- ----------
ChsiA52 ---------- ---------- ---------- ---------- ---------- ---------- ---------- ----------
OsheA57 ---------- ---------- ---------- ---------- ---------- ---------- ---------- ----------
ArplA75 ---------- ---------- ---------- ---------- ---------- ---------- ---------- ----------
LeKIOSTA86 ---------- ---------- ---------- ---------- ---------- ---------- ---------- ----------
OsniviP1 ISDQAELNAI GEKLYAQIDQ TWRTTPTFTE SLTYRVKVKQ NGDILQFDPT NKAAKDFVKE TPLPVLQTPA GAAAGAEKTA
FiCyA82 ---------- ---------- ---------- ---------- ---------- ---------- ---------- ----------
NespA78 ---------- ---------- ---------- ---------- ---------- ---------- ---------- ----------
SpsubHP ---------- ---------- ---------- ---------- ---------- ---------- ---------- ----------
Consensus ---------- ---------- ---------- ---------- ---------- ---------- ---------- ----------
100%
Conservation
0%
580 600 620 640
GlkiA84 ---------- ---------- ---------- ---------- ---------- ---------- ---------- ----------
Pl001A32 ---------- ---------- ---------- ---------- ---------- ---------- ---------- ----------
PhamA51 ---------- ---------- ---------- ---------- ---------- ---------- ---------- ----------
StcyA30 ---------- ---------- ---------- ---------- ---------- ---------- ---------- ----------
PlagHP2 DLEKLNQEVF ANINEAWTST PVTAVSVYLV KVSQTGEILS YEAKSADATQ NLNNTPLPKL VKSDVTQTPY TQFEVTFTPV
Gl7428C6 ---------- ---------- ---------- ---------- ---------- ---------- ---------- ----------
ChsiA52 ---------- ---------- ---------- ---------- ---------- ---------- ---------- ----------
OsheA57 ---------- ---------- ---------- ---------- ---------- ---------- ---------- ----------
ArplA75 ---------- ---------- ---------- ---------- ---------- ---------- ---------- ----------
LeKIOSTA86 ---------- ---------- ---------- ---------- ---------- ---------- ---------- ----------
OsniviP1 EFQVTFSPSN GGVFEVKSGK ---------- ---------- ---------- ---------- ---------- ----------
FiCyA82 ---------- ---------- ---------- ---------- ---------- ---------- ---------- ----------
NespA78 ---------- ---------- ---------- ---------- ---------- ---------- ---------- ----------
SpsubHP ---------- ---------- ---------- ---------- ---------- ---------- ---------- ----------
Consensus ---------- ---------- ---------- ---------- ---------- ---------- ---------- ----------
100%
Conservation
0%

GlkiA84	- - - - - - - - -	262
Pl001A32	- - - - - - - - -	499
PhamA51	- - - - - - - - -	261
StcyA30	- - - - - - - - -	267
PlagHP2	G T L D L K P V P	621
Gl7428C6	- - - - - - - - -	265
ChsiA52	- - - - - - - - -	266
OsheA57	- - - - - - - - -	265
ArplA75	- - - - - - - - -	265
LeKIOSTA86	- - - - - - - - -	265
OsniviP1	- - - - - - - - -	555
FiCyA82	- - - - - - - - -	264
NespA78	- - - - - - - - -	264
SpsubHP	- - - - - - - - -	267
Consensus	- - - - - - - - -	
Conservation (100% / 0%)		
