# Supplementary material for: Molecular interaction of nitrate transporter proteins with recombinant glycinebetaine results in efficient nitrate uptake in the cyanobacterium Anabaena PCC 7120
Source: PLoS One. 2021 Nov 18;16(11):e0257870. doi: 10.1371/journal.pone.0257870 (PMC8601584; doi:10.1371/journal.pone.0257870)
Supplement: S1 Table — (DOC) [file pone.0257870.s007.doc]

**Table S1. Sequence retrieval details of nrtA protein for multiple sequence alignment and phylogenetic tree construction showing accession number, proteins, organisms and name proposed.**

| **S. No.** | **Accession No.** | **Name of Protein** | **Organism** | **Name Proposed** |
| --- | --- | --- | --- | --- |
| 1 | BAB75032.1 | Nitrate-binding protein | *Nostoc* sp. PCC 7120 | No7120A1 |
| 2 | WP_067768543.1 | Bicarbonate-binding protein | *Nostoc* sp. NIES-3756 | No3756BT1 |
| 3 | WP_015141053.1 | Nitrate/sulfonate/bicarbonate ABC transporter substrate-binding protein | *Nostoc* sp. PCC 7524 | No7524SB1 |
| 4 | WP_017651187.1 | Bicarbonate-binding protein CmpA | *Fortiea contorta* | FocoBT2 |
| 5 | WP_015197497.1 | Twin-arginine translocation pathway signal | *Calothrix parietina* | CapaTA1 |
| 6 | WP_009453919.1 | Twin-arginine translocation pathway signal | *Fischerella* | FiTA2 |
| 7 | WP_062246501.1 | Bicarbonate-binding protein | *Fischerella* sp. NIES-3754 | Fi3754BT3 |
| 8 | WP_016860187.1 | Bicarbonate-binding protein CmpA | *Fischerella muscicola* | FimuBT4 |
| 9 | WP_066424585.1 | Bicarbonate-binding protein | *Anabaena* sp. 4-3 | An43BP1 |
| 10 | WP_066382327.1 | Bicarbonate-binding protein | *Anabaena* sp. CA = ATCC 33047 | An33047BP2 |
| 11 | WP_044450237.1 | Bicarbonate-binding protein | *Mastigocladus laminosus* | MalaBP3 |
| 12 | WP_016870987.1 | Bicarbonate-binding protein CmpA | *Fischerella thermalis* | FithBP4 |
| 13 | WP_053455523.1 | Bicarbonate-binding protein, partial | *Hapalosiphon* sp. MRB220 | Ha220BP5 |
| 14 | WP_026723799.1 | Bicarbonate-binding protein | *Fischerella* sp. PCC 9431 | Fi9431BP6 |
| 15 | WP_073620108.1 | Bicarbonate-binding protein | *Calothrix* sp. HK-06 | CaHK06BP7 |
| 16 | WP_019490882.1 | Bicarbonate-binding protein CmpA | *Calothrix* sp. PCC 7103 | Ca7103BP8 |
| 17 | WP_012629925.1 | Nitrate transporter | *Cyanothece* sp. PCC 7425 | Cy7425NT1 |
| 18 | WP_075599019.1 | Bicarbonate-binding protein | Oscillatoriales cyanobacterium 'hensonii' | OsheBP9 |
| 19 | WP_011429511.1 | Nitrate-binding protein | *Synechococcus* sp. JA-3-3Ab | Sy33AbNB1 |
| 20 | WP_011431707.1 | Nitrate ABC transporter substrate-binding protein | *Synechococcus* sp. JA-2-3B'a(2-13) | Sy23BSB2 |
| 21 | WP_017711817.1 | Hypothetical protein | *Prochlorothrix hollandica* | PrhoHP1 |
| 22 | WP_015180234.1 | Nitrate/sulfonate/bicarbonate ABC transporter substrate-binding protein | *Microcoleus* sp. PCC 7113 | Mi7113SB3 |
| 23 | WP_011057190.1 | Nitrate/nitrite transport system substrate-binding protein | *Thermosynechococcus elongates* | ThelSB4 |
| 24 | WP_035998664.1 | Bicarbonate-binding protein | *Leptolyngbya* sp. JSC-1 | LeJ1BP10 |
| 25 | WP_068514201.1 | Bicarbonate-binding protein | *Leptolyngbya* sp. O-77 | LeO77BP11 |
| 26 | WP_015166927.1 | Nitrate/sulfonate/bicarbonate ABC transporter substrate-binding protein | *Synechococcus* sp. PCC 7502 | Sy7502SB5 |
| 27 | WP_066121067.1 | Bicarbonate-binding protein | *Geminocystis* sp. NIES-3709 | Ge3709BP12 |
| 28 | WP_073608069.1 | Bicarbonate-binding protein | *Phormidium tenue* | PhteBP13 |
| 29 | WP_009554637.1 | Nitrate ABC transporter substrate-binding protein | Oscillatoriales cyanobacterium JSC-12 | Os12SB6 |
| 30 | WP_002740621.1 | Nitrate transporter | *Microcystis aeruginosa* | MiaeNT2 |
| 31 | WP_002792634.1 | Nitrate-binding protein | *Microcystis aeruginosa* | MiaeNB2 |
| 32 | WP_008313485.1 | Nitrate ABC transporter substrate-binding protein | *Leptolyngbya* sp. PCC 6406 | Le6406SB7 |
| 33 | WP_006616041.1 | Bicarbonate-binding protein | *Arthrospira* | ArNT3 |
| 34 | WP_055077330.1 | Bicarbonate-binding protein | *PseudAnabaena* sp. 'Roaring Creek' | PsRCBP14 |
| 35 | WP_011662703.1 | Nitrate transporter | *Rhodopseudomonas palustris* | RhpaSB8 |
| 36 | WP_017298507.1 | Bicarbonate-binding protein CmpA | *Nodosilinea nodulosa* | NonoBP15 |
| 37 | WP_014497015.1 | Nitrate-binding protein | *Bradyrhizobium japonicum* | BrjaNB3 |
| 38 | WP_024124218.1 | ABC nitrate/nitrite uptake system substrate-binding protein NrtA | *Thermosynechococcus* sp. NK55a | ThNK55aSB9 |
| 39 | WP_008203233.1 | Nitrate-binding protein | *Microcystis* sp. T1-4 | MiT14NB4 |
| 40 | WP_015188382.1 | Nitrate transporter | *Gloeocapsa* sp. PCC 7428 | Gl7428NT4 |
| 41 | WP_073550528.1 | Bicarbonate-binding protein | *Chroogloeocystis siderophila* | ChsiBP16 |
| 42 | WP_046660735.1 | Bicarbonate-binding protein | *Microcystis aeruginosa* | MiaeBP17 |
| 43 | WP_063196323.1 | Bicarbonate-binding protein | *Bradyrhizobium* sp. AT1 | BrAT1BP18 |
| 44 | WP_017294427.1 | Bicarbonate-binding protein CmpA | *Geminocystis herdmanii* | GeheBP19 |
| 45 | WP_027525548.1 | Bicarbonate-binding protein | *Bradyrhizobium* sp. Ec3.3 | BrEc33BP20 |
| 46 | WP_009626421.1 | Nitrate ABC transporter substrate-binding protein | *PseudAnabaena biceps* | PsbiSB10 |
| 47 | WP_026102964.1 | Bicarbonate-binding protein | *PseudAnabaena* sp. PCC 6802 | Ps6802BP21 |
| 48 | WP_045053370.1 | Bicarbonate-binding protein | *Aliterella atlantica* | AlatBP22 |
| 49 | WP_054464192.1 | Bicarbonate-binding protein | *Planktothricoides* sp. SR001 | Pl001BP23 |
| 50 | WP_063705620.1 | Bicarbonate-binding protein | *Bradyrhizobium* sp. BR 10245 | Br1024BP24 |
| 51 | WP_017720835.1 | Bicarbonate-binding protein CmpA | *Oscillatoria* sp. PCC 10802 | Os1080BP25 |
| 52 | WP_048434603.1 | Bicarbonate-binding protein | *Methylobacterium platani* | MeplBP26 |
| 53 | WP_028347335.1 | Bicarbonate-binding protein | *Bradyrhizobium elkanii* | BrelBP27 |
| 54 | WP_085352121.1 | Bicarbonate-binding protein | *Bradyrhizobium canariense* | BrcaBP28 |
| 55 | WP_062221849.1 | Bicarbonate-binding protein | *Aureimonas* sp. D3 | AuD3BP29 |
| 56 | WP_063680912.1 | Bicarbonate-binding protein | *Bradyrhizobium neotropicale* | BrneBP30 |
| 57 | WP_041393411.1 | Bicarbonate-binding protein | *Pleurocapsa minor* | PlmiBP31 |
| 58 | WP_073598534.1 | Bicarbonate-binding protein | *Hydrococcus rivularis* | HyriBP32 |
| 59 | WP_057843169.1 | Bicarbonate-binding protein | *Bradyrhizobium retamae* | BrreBP33 |
| 60 | WP_058598585.1 | Bicarbonate-binding protein | *Aureimonas ureilytica* | AuurBP34 |
| 61 | WP_060845660.1 | Bicarbonate-binding protein | *Methylobacterium aquaticum* | MeaqBP35 |
| 62 | WP_073597103.1 | Bicarbonate-binding protein | *Phormidium ambiguum* | PhamBP36 |
| 63 | WP_029005330.1 | Bicarbonate-binding protein | *Azorhizobium doebereinerae* | AzdoBP37 |
| 64 | WP_036004553.1 | Bicarbonate-binding protein | *Bradyrhizobium yuanmingense* | BryuBP38 |
| 65 | WP_015952673.1 | Twin-arginine translocation pathway signal | *Methylobacterium extorquens* | MeexTA3 |
| 66 | WP_028177814.1 | Bicarbonate-binding protein | *Bradyrhizobium japonicum* | BrjaBP39 |
| 67 | WP_079586607.1 | Bicarbonate-binding protein | *Bradyrhizobium lablabi* | BrlaBP40 |
| 68 | WP_011440656.1 | Twin-arginine translocation pathway signal | *Rhodopseudomonas palustris* | RhpaTA4 |
| 69 | WP_012456706.1 | Twin-arginine translocation pathway signal | *Methylobacterium populi* | MepoTA5 |
| 70 | WP_055454261.1 | Bicarbonate-binding protein | *Pannonibacter indicus* | PainBP41 |
| 71 | WP_042002401.1 | Bicarbonate-binding protein | *Streptomyces* sp. AcH 505 | St505BP42 |
| 72 | WP_015170816.1 | Nitrate ABC transporter substrate-binding protein | *Geitlerinema* sp. PCC 7407 | Ge7407SB11 |
| 73 | WP_036294439.1 | Bicarbonate-binding protein | *Methylosinus* sp. PW1 | MePW1BP43 |
| 74 | WP_022962231.1 | Bicarbonate-binding protein CmpA | *Pseudomonas pelagia* | PspeBP44 |
| 75 | WP_015219206.1 | Nitrate transport 45kD protein | *Cyanobacterium aponinum* | CyapNT4 |
| 76 | WP_007816837.1 | Nitrate ABC transporter substrate-binding protein | *Rhizobium* sp. CF142 | Rh142SB12 |
| 77 | WP_029010539.1 | Bicarbonate-binding protein | *Azospirillum halopraeferens* | AzhaBP45 |
| 78 | WP_050473102.1 | Bicarbonate-binding protein | *Pannonibacter phragmitetus* | PaphBP46 |
| 79 | WP_054167764.1 | Bicarbonate-binding protein | *alpha proteobacterium* AAP38 | Alpr38BP47 |
| 80 | WP_026290005.1 | Bicarbonate-binding protein | *Thioalkalivibrio sulfidiphilus* | TlsuBP48 |
| 81 | WP_002715797.1 | Hypothetical protein | *Afipia felis* | AffeHP3 |
| 82 | WP_024580851.1 | Bicarbonate-binding protein | *Bradyrhizobium* | BrBP50 |
| 83 | WP_015785372.1 | Nitrate ABC transporter substrate-binding protein | *Cyanothece* | CySB13 |
| 84 | WP_037098641.1 | Bicarbonate-binding protein | *Rhizobium alamii* | RhalBP51 |
| 85 | WP_009632003.1 | Nitrate ABC transporter substrate-binding protein | *Synechocystis* sp. PCC 7509 | Sy7509NT5 |
| 86 | WP_020812591.1 | Bicarbonate-binding protein CmpA | *Agrobacterium tumefaciens* | AgtuBP52 |
| 87 | WP_080857579.1 | Bicarbonate-binding protein | *Agrobacterium genomo* sp. 7 | Agge7BP53 |
| 88 | WP_010629446.1 | Nitrate-binding protein,periplasmic | *Halomonas* sp. KM-1 | HaKMNT6 |
| 89 | WP_015887598.1 | Nitrate ABC transporter substrate-binding protein | *Sinorhizobium fredii* | SifrSB14 |
| 90 | WP_024277052.1 | Bicarbonate-binding protein | *Xanthobacter* sp. 126 | Xan126BP54 |
| 91 | WP_028743134.1 | Bicarbonate-binding protein | *Rhizobium leguminosarum* | RhilgBP55 |
| 92 | WP_065117158.1 | Bicarbonate-binding protein | *Agrobacterium rhizogenes* | AgrhiBP56 |
| 93 | WP_014761948.1 | Nitrate transporter | *Sinorhizobium fredii* | SifrNT7 |
| 94 | WP_078341062.1 | Bicarbonate-binding protein | *Ochrobactrum* sp. P6BS-III | OcP6BSBP57 |
| 95 | WP_042555239.1 | Bicarbonate-binding protein | *Pseudomonas fulva* | PsfuBP58 |
| 96 | WP_064992988.1 | Bicarbonate-binding protein | *Mesorhizobium* sp. WSM1497 | Ms1497BP59 |
| 97 | WP_086088978.1 | Bicarbonate-binding protein | *Pseudorhodoplanes sinuspersici* | Pssibp60 |
| 98 | WP_075893906.1 | Bicarbonate-binding protein | *Limnothrix rosea* | LiroBP61 |
| 99 | WP_002711764.1 | Hypothetical protein | *Afipia clevelandensis* | AfclHP5 |
| 100 | WP_065781836.1 | Bicarbonate-binding protein | *Ensifer* | EnBP62 |
| 101 | WP_016915167.1 | Bicarbonate-binding protein | *Halomonas* | HaloBP63 |
| 102 | WP_046599953.1 | Bicarbonate-binding protein | *Neorhizobium galegae* | NegaBP64 |
| 103 | WP_018238580.1 | Bicarbonate-binding protein CmpA | *Ensifer* sp. BR816 | En816BP65 |
