# Supplementary material for: Molecular interaction of nitrate transporter proteins with recombinant glycinebetaine results in efficient nitrate uptake in the cyanobacterium Anabaena PCC 7120
Source: PLoS One. 2021 Nov 18;16(11):e0257870. doi: 10.1371/journal.pone.0257870 (PMC8601584; doi:10.1371/journal.pone.0257870)
Supplement: S2 Table — (DOC) [file pone.0257870.s008.doc]

**Table S2. Sequence retrieval details of nrtC protein for multiple sequence alignment and phylogenetic tree construction showing accession number, proteins, organisms and name proposed.**

| **S. No.** | **Accession No.** | **Name of Protein** | **Organism** | **Name Proposed** |
| --- | --- | --- | --- | --- |
| 1 | WP_010994786.1 | Bacitracin ABC transporter ATP-binding protein | *Nostoc* sp. PCC 7120 | No7120A1 |
| 2 | ABA24140.1 | Nitrate transport ATP-binding subunits C and D | *Anabaena variabilis* ATCC 29413 | An29413A2 |
| 3 | WP_067763412.1 | Bacitracin ABC transporter ATP-binding protein | *Nostoc* sp. NIES-3756 | No3756A3 |
| 4 | WP_066425022.1 | Bacitracin ABC transporter ATP-binding protein | *Anabaena* sp. 4-3 | An43A4 |
| 5 | WP_066379927.1 | Bacitracin ABC transporter ATP-binding protein | *Anabaena* sp. CA = ATCC 33047 | An33047A5 |
| 6 | WP_069070193.1 | Bacitracin ABC transporter ATP-binding protein | *Nostoc* sp. KVJ20 | NoKVJ20A6 |
| 7 | WP_015140511.1 | Nitrate transport ATP-binding subunits C and D | *Nostoc* sp. PCC 7524 | No7524C1 |
| 8 | WP_045872526.1 | Bacitracin ABC transporter ATP-binding protein | *Tolypothrix* sp. PCC 7601 | To7601A7 |
| 9 | WP_016877344.1 | Bacitracin ABC transporter ATP-binding protein | *Chlorogloeopsis fritschii* | ChfrA8 |
| 10 | OUL37688.1 | Bacitracin ABC transporter ATP-binding protein | *Nostoc* sp. T09 | NoT09A9 |
| 11 | WP_062289971.1 | Bacitracin ABC transporter ATP-binding protein | *Nostoc piscinale* | NopiA10 |
| 12 | OUL34525.1 | bacitracin ABC transporter ATP-binding protein | *Nostoc* sp. 106C | No106CA11 |
| 13 | WP_062247085.1 | Bacitracin ABC transporter ATP-binding protein | *Fischerella* sp. NIES-3754 | Fi3754A12 |
| 14 | WP_026732017.1 | Bacitracin ABC transporter ATP-binding protein | *Fischerella* sp. PCC 9605 | Fi9605A13 |
| 15 | WP_009455133.1 | Bacitracin ABC transporter ATP-binding protein | *Fischerella* sp. JSC-11 | FiJSC11A14 |
| 16 | OCQ94753.1 | Bacitracin ABC transporter ATP-binding protein | *Nostoc* sp. MBR 210 | No210A15 |
| 17 | WP_017653156.1 | Bacitracin ABC transporter ATP-binding protein | *Fortiea contorta* | FocoA16 |
| 18 | WP_016868533.1 | Bacitracin ABC transporter ATP-binding protein | *Fischerella muscicola* | FimuA17 |
| 19 | WP_017310281.1 | Bacitracin ABC transporter ATP-binding protein | *Hapalosiphonaceae* | HapaloA18 |
| 20 | WP_017321042.1 | Bacitracin ABC transporter ATP-binding protein | *Cyanobacterium* PCC 7702 | Cy7702A19 |
| 21 | CEJ43998.1 | Nitrate ABC transporter, ATPase subunits C and D | *Chrysosporum ovalisporum* | ChrovaA20 |
| 22 | WP_019494228.1 | Bacitracin ABC transporter ATP-binding protein | *Calothrix* sp. PCC 7103 | Ca7103A21 |
| 23 | WP_073617563.1 | Bacitracin ABC transporter ATP-binding protein | *Calothrix* sp. HK-06 | CaHK06A22 |
| 24 | OBQ24284.1 | Bacitracin ABC transporter ATP-binding protein | *Aphanizomenon flos-aquae* LD13 | AphflosA23 |
| 25 | BAU66173.1 | Nitrate ABC transporter, ATPase subunits C and D | *Stanieria* sp. NIES-3757 | St3757A24 |
| 26 | WP_038081182.1 | Bacitracin ABC transporter ATP-binding protein | *Tolypothrix bouteillei* | TobouA25 |
| 27 | WP_035155288.1 | Bacitracin ABC transporter ATP-binding protein | *Calothrix* sp. 336/3 | Ca3363A26 |
| 28 | WP_045053372.1 | Bacitracin ABC transporter ATP-binding protein | *Aliterella atlantica* | AliatlA27 |
| 29 | WP_015196746.1 | Nitrate ABC transporter ATPase C and D | *Calothrix parietina* | CaparA28 |
| 30 | WP_009632005.1 | Nitrate transport ATP-binding subunits C and D | *Synechocystis* sp. PCC 7509 | Syn7509A29 |
| 31 | WP_015155571.1 | Nitrate ABC transporter ATPase C and D | *Chroococcidiopsis thermalis* | ChrtherA30 |
| 32 | WP_015180232.1 | Nitrate transport ATP-binding subunits C and D | *Microcoleus* sp. PCC 7113 | Mic7113A31 |
| 33 | WP_073550530.1 | Bacitracin ABC transporter ATP-binding protein | *Chroogloeocystis siderophila* | ChrosidA32 |
| 34 | WP_015188384.1 | Nitrate ABC transporter ATPases C and D | *Gloeocapsa* sp. PCC 7428 | Glo7428A33 |
| 35 | WP_007355412.1 | Bacitracin ABC transporter ATP-binding protein | *Kamptonema* | KamptonA34 |
| 36 | WP_053539149.1 | Bacitracin ABC transporter ATP-binding protein | *Anabaena* sp. WA102 | AnWA102A35 |
| 37 | WP_039201141.1 | Bacitracin ABC transporter ATP-binding protein | *Aphanizomenon flos-aquae* | AphflosA36 |
| 38 | OBQ15895.1 | Bacitracin ABC transporter ATP-binding protein | *Anabaena* sp. AL93 | AnAL93A37 |
| 39 | WP_027400666.1 | Bacitracin ABC transporter ATP-binding protein | *Aphanizomenon flos-aquae* | AphflosA38 |
| 40 | WP_071189519.1 | Bacitracin ABC transporter ATP-binding protein | *Trichormus* sp. NMC-1 | TriNMC1A39 |
| 41 | WP_083616795.1 | Bacitracin ABC transporter ATP-binding protein | *Planktothrix serta* | PlaserA40 |
| 42 | WP_012629923.1 | Bacitracin ABC transporter ATP-binding protein | *Cyanothece* sp. PCC 7425 | Cy7425A41 |
| 43 | WP_015204596.1 | Nitrate ABC transporter ATPase C and D | *Crinalium epipsammum* | CriepiA42 |
| 44 | WP_073608070.1 | Bacitracin ABC transporter ATP-binding protein | *Phormidium tenue* | PhotenA43 |
| 45 | WP_072718090.1 | Bacitracin ABC transporter ATP-binding protein | *Planktothrix tepida* | PlatepA44 |
| 46 | WP_075599017.1 | Bacitracin ABC transporter ATP-binding protein | Oscillatoriales cyanobacterium 'hensonii' | OshensA45 |
| 47 | WP_069966852.1 | Bacitracin ABC transporter ATP-binding protein | *Desertifilum* sp. IPPAS B-1220 | Des1220A46 |
| 48 | OIP67536.1 | Bacitracin ABC transporter ATP-binding protein | Oscillatoriales cyanobacterium CG2_30_40_61 | Os30406A47 |
| 49 | WP_015192768.1 | Nitrate ABC transporter ATPases C and D | *Stanieria cyanosphaera* | StcyaA48 |
| 50 | WP_017720837.1 | Bacitracin ABC transporter ATP-binding protein | *Oscillatoria* sp. PCC 10802 | Os10802A49 |
| 51 | WP_006632840.1 | Bacitracin ABC transporter ATP-binding protein | *Microcoleus vaginatus* | MicvagA50 |
| 52 | WP_073598503.1 | Bacitracin ABC transporter ATP-binding protein | *Hydrococcus rivularis* | HydrivA51 |
| 53 | WP_017305085.1 | Hypothetical protein | *Spirulina subsalsa* | SpirsubA52 |
| 54 | WP_035991066.1 | Bacitracin ABC transporter ATP-binding protein | *Leptolyngbya* sp. KIOST-1 | LeKIOSTA53 |
| 55 | WP_015144734.1 | Nitrate transport ATP-binding subunits C and D | *Pleurocapsa minor* | PleminC2 |
| 56 | WP_026796599.1 | Nacitracin ABC transporter ATP-binding protein | *Planktothrix prolifica* | PlaproA54 |
| 57 | WP_079678859.1 | Bacitracin ABC transporter ATP-binding protein | *Planktothrix* sp. PCC 11201 | Pla1120A55 |
| 58 | WP_015179105.1 | nitrate ABC transporter ATPases C and D | *Oscillatoria nigro-viridis* | OsniviA56 |
| 59 | OCQ99285.1 | Bacitracin ABC transporter ATP-binding protein | Oscillatoriales cyanobacterium USR001 | Oscy001A57 |
| 60 | WP_026787880.1 | Bacitracin ABC transporter ATP-binding protein | *Planktothrix rubescens* | PlarubA58 |
| 61 | WP_027254352.1 | Bacitracin ABC transporter ATP-binding protein | *Planktothrix agardhii* | PlaagaA59 |
| 62 | WP_006275770.1 | Bacitracin ABC transporter ATP-binding protein | *Cylindrospermopsis raciborskii* | CylracA60 |
| 63 | WP_058998044.1 | Bacitracin ABC transporter ATP-binding protein | *Leptolyngbya* sp. NIES-2104 | Le2104A61 |
| 64 | WP_073633349.1 | Bacitracin ABC transporter ATP-binding protein | *Scytonema* sp. HK-05 | ScyHK05A62 |
| 65 | WP_015161466.1 | Nitrate transport ATP-binding subunits C and D | *Chamaesiphon minutus* | ChamminuC3 |
| 66 | WP_041041552.1 | Bacitracin ABC transporter ATP-binding protein | *Tolypothrix campylonemoides* | TocamA63 |
| 67 | BAW97359.1 | Nitrate ABC transporter, ATPase subunits C and D | *Synechococcus* sp. NIES-970 | Sy970A64 |
| 68 | WP_029681844.1 | Bacitracin ABC transporter ATP-binding protein | *Synechococcus* sp. NKBG15041c | Sy15041A65 |
| 69 | WP_017318772.1 | Bacitracin ABC transporter ATP-binding protein | *Mastigocladopsis repens* | MasrepA66 |
| 70 | OBQ26683.1 | Bacitracin ABC transporter ATP-binding protein | *Aphanizomenon flos-aquae* LD13 | AphflosA67 |
| 71 | WP_058883035.1 | Bacitracin ABC transporter ATP-binding protein | Oscillatoriales cyanobacterium MTP1 | OscyMTPA68 |
| 72 | WP_015955510.1 | Bacitracin ABC transporter ATP-binding protein | *Cyanothece* sp. PCC 7424 | Cy7424A69 |
| 73 | WP_015170818.1 | Nitrate ABC transporter ATPase C and D | *Geitlerinema* sp. PCC 7407 | Ge7407A70 |
| 74 | WP_006669969.1 | Bacitracin ABC transporter ATP-binding protein | *Arthrospira maxima* | ArtmaxA71 |
| 75 | WP_073597101.1 | Bacitracin ABC transporter ATP-binding protein | *Phormidium ambiguum* | PhoambA72 |
| 76 | WP_068378995.1 | Bacitracin ABC transporter ATP-binding protein | *Leptolyngbya* sp. NIES-3755 | Le3755A73 |
| 77 | WP_006616416.1 | Bacitracin ABC transporter ATP-binding protein | *Arthrospira platensis* | ArtplaA74 |
| 78 | WP_017288932.1 | Hypothetical protein | *Leptolyngbya boryana* | LeborHP1 |
| 79 | WP_057177292.1 | Bacitracin ABC transporter ATP-binding protein | *Cylindrospermopsis* sp. CR12 | CylCR12A75 |
| 80 | AAY28871.1 | NrtC | *Geitlerinema* sp. PCC 8501 | Ge8501NRTC |
| 81 | WP_066344220.1 | Bacitracin ABC transporter ATP-binding protein | *Geminocystis* sp. NIES-3708 | Gem3708A76 |
| 82 | WP_008203236.1 | Bacitracin ABC transporter ATP-binding protein | *Microcystis* sp. T1-4 | MicT14A77 |
| 83 | WP_002784029.1 | Nitrate reductase | *Microcystis aeruginosa* | MicaerNR |
| 84 | WP_061431300.1 | bacitracin ABC transporter ATP-binding protein | *Microcystis aeruginosa* | MicaerA78 |
| 85 | WP_002740627.1 | Bicarbonate transport ATP-binding protein CmpC | *Microcystis aeruginosa* | MicaerCmpC |
| 86 | WP_010994786.1 | Bacitracin ABC transporter ATP-binding protein | *Nostoc* sp. PCC 7120 | No7120A1 |
