# Supplementary material for: Molecular interaction of nitrate transporter proteins with recombinant glycinebetaine results in efficient nitrate uptake in the cyanobacterium Anabaena PCC 7120
Source: PLoS One. 2021 Nov 18;16(11):e0257870. doi: 10.1371/journal.pone.0257870 (PMC8601584; doi:10.1371/journal.pone.0257870)
Supplement: S3 Table — (DOC) [file pone.0257870.s009.doc]

**Table S3.** Sequence retrieval details of nrtD protein for multiple sequence alignment and phylogenetic tree construction showing accession number, proteins, organisms and name proposed.

| **S. No.** | **Accession No.** | **Name of Protein** | **Organism** | **Name Proposed** |
| --- | --- | --- | --- | --- |
| 1 | WP_010994787.1 | Bacitracin ABC transporter ATP-binding protein | *Nostoc* sp. PCC 7120 | No7120A1 |
| 2 | WP_067763414.1 | Bacitracin ABC transporter ATP-binding protein | *Nostoc* sp. NIES-3756 | No3756A2 |
| 3 | WP_015140510.1 | Nitrate transport ATP-binding subunits C and D | *Nostoc* sp. PCC 7524 | No7524C1 |
| 4 | WP_062289969.1 | Bacitracin ABC transporter ATP-binding protein | *Nostoc* *piscinale* | NopiA3 |
| 5 | WP_066379923.1 | Bacitracin ABC transporter ATP-binding protein | *Anabaena* sp. CA = ATCC 33047 | An33047A4 |
| 6 | WP_069070194.1 | Bacitracin ABC transporter ATP-binding protein | *Nostoc* sp. KVJ20 | NoKVJ20A5 |
| 7 | WP_045872525.1 | Bacitracin ABC transporter ATP-binding protein | *Tolypothrix* sp. PCC 7601 | To7601A6 |
| 8 | WP_073617562.1 | Bacitracin ABC transporter ATP-binding protein | *Calothrix* sp. HK-06 | CaHK06A7 |
| 9 | WP_017653155.1 | Bacitracin ABC transporter ATP-binding protein | *Fortiea contorta* | FocoA8 |
| 10 | WP_019494227.1 | Bacitracin ABC transporter ATP-binding protein | *Calothrix* sp. PCC 7103 | Ca7103A9 |
| 11 | WP_016877343.1 | Bacitracin ABC transporter ATP-binding protein | *Chlorogloeopsis fritschii* | ChfrA10 |
| 12 | WP_066425024.1 | Bacitracin ABC transporter ATP-binding protein | *Anabaena* sp. 4-3 | An43A11 |
| 13 | WP_026732016.1 | Bacitracin ABC transporter ATP-binding protein | *Fischerella* sp. PCC 9605 | Fi9605A12 |
| 14 | WP_017310280.1 | Bacitracin ABC transporter ATP-binding protein | *Fischerella* sp. PCC 9339 | Fi9339A13 |
| 15 | WP_009455136.1 | Bacitracin ABC transporter ATP-binding protein | *Fischerella* sp. JSC-11 | FiJSC11A14 |
| 16 | WP_062247083.1 | Bacitracin ABC transporter ATP-binding protein | *Fischerella* sp. NIES-3754 | Fi3754A15 |
| 17 | WP_071189520.1 | Bacitracin ABC transporter ATP-binding protein | *Trichormus* sp. NMC-1 | TriNMC1A16 |
| 18 | WP_035155286.1 | Bacitracin ABC transporter ATP-binding protein | *Calothrix* sp. 336/3 | Ca336A17 |
| 19 | WP_053539148.1 | Bacitracin ABC transporter ATP-binding protein | *Anabaena* sp. WA102 | AbWA102A18 |
| 20 | WP_027400667.1 | Bacitracin ABC transporter ATP-binding protein | *Aphanizomenon flosaquae* | AphflosA19 |
| 21 | WP_045053373.1 | Bacitracin ABC transporter ATP-binding protein | *Aliterella atlantica* | AlatA20 |
| 22 | WP_038087528.1 | Bacitracin ABC transporter ATP-binding protein | *Tolypothrix bouteillei* | TobouA21 |
| 23 | WP_017321041.1 | Bacitracin ABC transporter ATP-binding protein | Cyanobacterium PCC 7702 | Cy7702A22 |
| 24 | WP_009632006.1 | Nitrate transport ATP-binding subunits C and D | *Synechocystis* sp. PCC 7509 | Sy7509A23 |
| 25 | WP_071250916.1 | Bacitracin ABC transporter ATP-binding protein | *Cylindrospermopsis raciborskii* | CyraA24 |
| 26 | WP_026099376.1 | Bacitracin ABC transporter ATP-binding protein | *Prochlorothrix hollandica* | PrhoA25 |
| 27 | WP_011429514.1 | Bacitracin ABC transporter ATP-binding protein | *Synechococcus* sp. JA-3-3Ab | Sy33AbA26 |
| 28 | WP_015108593.1 | Nitrate transport ATP-binding subunits C and D | *Cyanobium gracile* | CygrC2 |
| 29 | WP_011057194.1 | Bacitracin ABC transporter ATP-binding protein | *Thermosynechococcus elongatus* | ThelA27 |
| 30 | WP_011432250.1 | Bacitracin ABC transporter ATP-binding protein | *Synechococcus* sp. JA-2-3B'a(2-13) | Sy23BaA28 |
| 31 | WP_044449967.1 | Bacitracin ABC transporter ATP-binding protein | *Mastigocladus laminosus* | MalaA29 |
| 32 | WP_041619233.1 | Bacitracin ABC transporter ATP-binding protein | *Stanieria cyanosphaera* | StcyA30 |
| 33 | WP_015179104.1 | Nitrate ABC transporter ATPases C and D | *Oscillatoria nigro-viridis* | OsniviP1 |
| 34 | WP_054464195.1 | Nitrate ABC transporter ATP-binding protein | *Planktothricoides* sp. SR001 | Pl001A32 |
| 35 | WP_009344156.1 | Bacitracin ABC transporter ATP-binding protein | *Raphidiopsis brookii* | RabrA33 |
| 36 | WP_013321844.1 | Bacitracin ABC transporter ATP-binding protein | *Cyanothece* sp. PCC 7822 | Cy7822A34 |
| 37 | WP_008278223.1 | Bacitracin ABC transporter ATP-binding protein | *Cyanothece* sp. CCY0110 | Cy0110A35 |
| 38 | WP_015956618.1 | Bacitracin ABC transporter ATP-binding protein | *Cyanothece* sp. PCC 7424 | Cy7424A36 |
| 39 | WP_017719674.1 | Bacitracin ABC transporter ATP-binding protein | *Oscillatoria* sp. PCC 10802 | Os10802A37 |
| 40 | WP_015196747.1 | Nitrate ABC transporter ATPase C and D | *Calothrix parietina* | CapaA38 |
| 41 | WP_073547894.1 | Bacitracin ABC transporter ATP-binding protein | *Chroogloeocystis siderophila* | ChsiA39 |
| 42 | WP_015189781.1 | Nitrate ABC transporter, ATPase subunits C and D | *Gloeocapsa* sp. PCC 7428 | Gl7428A40 |
| 43 | WP_073600723.1 | Bacitracin ABC transporter ATP-binding protein | *Hydrococcus rivularis* | HyriA41 |
| 44 | WP_073633350.1 | Bacitracin ABC transporter ATP-binding protein | *Scytonema* sp. HK-05 | ScHK05A42 |
| 45 | WP_015143223.1 | Nitrate transport ATP-binding subunits C and D | *Pleurocapsa minor* | PlemiC3 |
| 46 | WP_009631067.1 | Nitrate transport ATP-binding subunits C and D | *Synechocystis* sp. PCC 7509 | Sy7509C4 |
| 47 | WP_041041554.1 | Bacitracin ABC transporter ATP-binding protein | *Tolypothrix campylonemoides* | TocaA43 |
| 48 | WP_017318773.1 | Bacitracin ABC transporter ATP-binding protein | *Mastigocladopsis repens* | MareA44 |
| 49 | WP_015194407.1 | Nitrate ABC transporter ATPases C and D | *Stanieria cyanosphaera* | StcyC5 |
| 50 | WP_051077082.1 | Bacitracin ABC transporter ATP-binding protein | *Scytonema hofmannii* | SchofA45 |
| 51 | WP_015188385.1 | Nitrate ABC transporter ATPases C and D | *Gloeocapsa* sp. PCC 7428 | Gl7428C6 |
| 52 | WP_012630258.1 | Bacitracin ABC transporter ATP-binding protein | *Cyanothece* sp. PCC 7425 | Cy7425A46 |
| 53 | WP_015161467.1 | Nitrate transport ATP-binding subunits C and D | *Chamaesiphon minutus* | ChaminC7 |
| 54 | WP_066121665.1 | Bacitracin ABC transporter ATP-binding protein | *Geminocystis* sp. NIES-3709 | Ge3709A47 |
| 55 | WP_028082986.1 | Bacitracin ABC transporter ATP-binding protein | *Dolichospermum circinale* | DociA48 |
| 56 | WP_012408916.1 | Bacitracin ABC transporter ATP-binding protein | *Nostoc punctiforme* | NopunA49 |
| 57 | WP_015152992.1 | Nitrate ABC transporter ATPases C and D | *Chroococcidiopsis thermalis* | ChrthA50 |
| 58 | WP_073597100.1 | Bacitracin ABC transporter ATP-binding protein | *Phormidium ambiguum* | PhamA51 |
| 59 | WP_073550531.1 | Bacitracin ABC transporter ATP-binding protein | *Chroogloeocystis siderophila* | ChsiA52 |
| 60 | WP_073641103.1 | Bacitracin ABC transporter ATP-binding protein | *Nostoc calcicola* | NocaA53 |
| 61 | WP_017292854.1 | Bacitracin ABC transporter ATP-binding protein | *Geminocystis herdmanii* | GeherA54 |
| 62 | WP_066375789.1 | Bacitracin ABC transporter ATP-binding protein | *Anabaena* sp. CA = ATCC 33047 | Ab33047A55 |
| 63 | WP_041555333.1 | Bacitracin ABC transporter ATP-binding protein | *Nostoc* sp. PCC 7524 | No7524A56 |
| 64 | WP_015204595.1 | nitrate ABC transporter ATPase C and D | *Crinalium epipsammum* | CrepC8 |
| 65 | WP_075599163.1 | bacitracin ABC transporter ATP-binding protein | Oscillatoriales cyanobacterium '*hensonii*' | OsheA57 |
| 66 | WP_068509329.1 | Bacitracin ABC transporter ATP-binding protein | *Leptolyngbya* sp. O-77 | LeO77A58 |
| 67 | WP_009341908.1 | Bacitracin ABC transporter ATP-binding protein | Aphanizomenonaceae | AphflosA59 |
| 68 | WP_015216436.1 | Nitrate ABC transporter ATPases C and D | *Anabaena cylindrica* | AncyC9 |
| 69 | WP_015184636.1 | Nitrate transport ATP-binding subunits C and D | *Microcoleus* sp. PCC 7113 | Mi7113C10 |
| 70 | WP_048867897.1 | Bacitracin ABC transporter ATP-binding protein | *Scytonema tolypothrichoides* | SctoA60 |
| 71 | WP_028948332.1 | Bacitracin ABC transporter ATP-binding protein | *Synechocystis* sp. PCC 6714 | Sy6714A61 |
| 72 | WP_015112904.1 | Nitrate ABC transporter ATPase C and D | *Nostoc* sp. PCC 7107 | No7107C11 |
| 73 | WP_026721425.1 | Bacitracin ABC transporter ATP-binding protein | Hapalosiphonaceae | HapA62 |
| 74 | WP_082348937.1 | Bacitracin ABC transporter ATP-binding protein | *Planktothricoides* sp. SR001 | Pl001A63 |
| 75 | WP_029635808.1 | Bacitracin ABC transporter ATP-binding protein | *Scytonema hofmanni* UTEX B 1581 | Sc1581A64 |
| 76 | WP_015083516.1 | Nitrate ABC transporter NrtD | *Anabaena* sp. 90 | An90A65 |
| 77 | WP_015210079.1 | Nitrate transport ATP-binding subunits C and D | *Cylindrospermum stagnale* | CystC12 |
| 78 | WP_085951643.1 | Bacitracin ABC transporter ATP-binding protein | *Chlorogloeopsis fritschii* | ChfrA66 |
| 79 | WP_015129673.1 | Nitrate ABC transporter ATPase C and D | *Calothrix* sp. PCC 7507 | Ca7507A67 |
| 80 | WP_002784026.1 | Nitrate reductase | *Microcystis aeruginosa* | MiaerNR |
| 81 | WP_017305086.1 | Hypothetical protein | *Spirulina subsalsa* | SpsubHP |
| 82 | WP_036535906.1 | Bacitracin ABC transporter ATP-binding protein | *Neosynechococcus sphagnicola* | NesphA68 |
| 83 | WP_019502704.1 | Bacitracin ABC transporter ATP-binding protein | *Pseudanabaena* sp. PCC 6802 | Ps6802A69 |
| 84 | WP_036484539.1 | Bacitracin ABC transporter ATP-binding protein | *Myxosarcina* sp. GI1 | MyGI1A70 |
| 85 | WP_016516147.1 | Bacitracin ABC transporter ATP-binding protein | *Microcystis aeruginosa* | MiaerBIT |
| 86 | WP_069789270.1 | Bacitracin ABC transporter ATP-binding protein | Cyanobacterium sp. IPPAS B-1200 | Cy1200A71 |
| 87 | WP_017302394.1 | Bacitracin ABC transporter ATP-binding protein | *Nodosilinea nodulosa* | NodnodA72 |
| 88 | WP_066121072.1 | Bacitracin ABC transporter ATP-binding protein | *Geminocystis* sp. NIES-3709 | Ge3709A73 |
| 89 | WP_039896114.1 | Bacitracin ABC transporter ATP-binding protein | *Lyngbya* sp. PCC 8106 | Ly8106A74 |
| 90 | WP_006616417.1 | Bacitracin ABC transporter ATP-binding protein | *Arthrospira platensis* | ArplA75 |
| 91 | WP_011142088.1 | Bacitracin ABC transporter ATP-binding protein | *Gloeobacter violaceus* | GlviA76 |
| 92 | WP_036530922.1 | Bacitracin ABC transporter ATP-binding protein | *Neosynechococcus sphagnicola* | NespA78 |
| 93 | WP_006632551.1 | Bacitracin ABC transporter ATP-binding protein | *Microcoleus vaginatus* | MivagA79 |
| 94 | WP_011057838.1 | Bacitracin ABC transporter ATP-binding protein | *Thermosynechococcus elongatus* | TheloA80 |
| 95 | WP_007355413.1 | Nitrate ABC transporter ATP-binding protein | *Kamptonema* | KampA81 |
| 96 | WP_018399284.1 | Bacitracin ABC transporter ATP-binding protein | Filamentous cyanobacterium ESFC-1 | FiCyA82 |
| 97 | WP_081693735.1 | Hypothetical protein | *Planktothrix agardhii* | PlagHP2 |
| 98 | WP_026148817.1 | Bacitracin ABC transporter ATP-binding protein | *Leptolyngbya boryana* | LeboA83 |
| 99 | WP_015159564.1 | Nitrate transport ATP-binding subunits C and D | *Chamaesiphon minutus* | ChaminC13 |
| 100 | WP_041244108.1 | Bacitracin ABC transporter ATP-binding protein | *Gloeobacter kilaueensis* | GlkiA84 |
| 101 | WP_009628543.1 | Nitrate ABC transporter ATPases C and D | *Pseudanabaena biceps* | PsbicA85 |
| 102 | WP_035991064.1 | Bacitracin ABC transporter ATP-binding protein | *Leptolyngbya* sp. KIOST-1 | LeKIOSTA86 |
| 103 | WP_083622630.1 | Bacitracin ABC transporter ATP-binding protein | *Planktothrix serta* | PlseA87 |
| 104 | WP_082429378.1 | Bacitracin ABC transporter ATP-binding protein | *Pseudanabaena* sp. 'Roaring Creek' | PsRCrA88 |
| 105 | WP_051372890.1 | Bacitracin ABC transporter ATP-binding protein | *Thermosynechococcus* sp. NK55a | ThNK55aA89 |
| 106 | WP_010994787.1 | Bacitracin ABC transporter ATP-binding protein | *Nostoc* sp. PCC 7120 | No7120A1 |
